# Supplementary material for: p53 regulates DREAM complex-mediated repression in a p21-independent manner
Source: EMBO J. 2025 Mar 4;44(8):2279–97. doi: 10.1038/s44318-025-00402-7 (PMC12000331; doi:10.1038/s44318-025-00402-7)
Supplement: Supplementary file 1 — Appendix [file 44318_2025_402_MOESM1_ESM.pdf]

Appendix for
p53 regulates DREAM complex-mediated repression in a p21-independent manner

Ritu Agrawal[1], Sagar Sengupta[1,2,3]

[3]Corresponding author: ssg2@nibmg.ac.in

This PDF file includes:

Appendix Figure S1……Page 2-3
Appendix Figure S2……Page 4
Appendix Figure S3……Page 5-6
Appendix Figure S4……Page 7-8
Appendix Figure S5……Page 9
Appendix Figure S6……Page 10-11
Appendix Figure S7……Page 12-13
Appendix Table S1……..(Statistical analysis) Page 14-16
Appendix Table S2……..(PCR primers) Page 17-20

Appendix Figures and Figure Legends

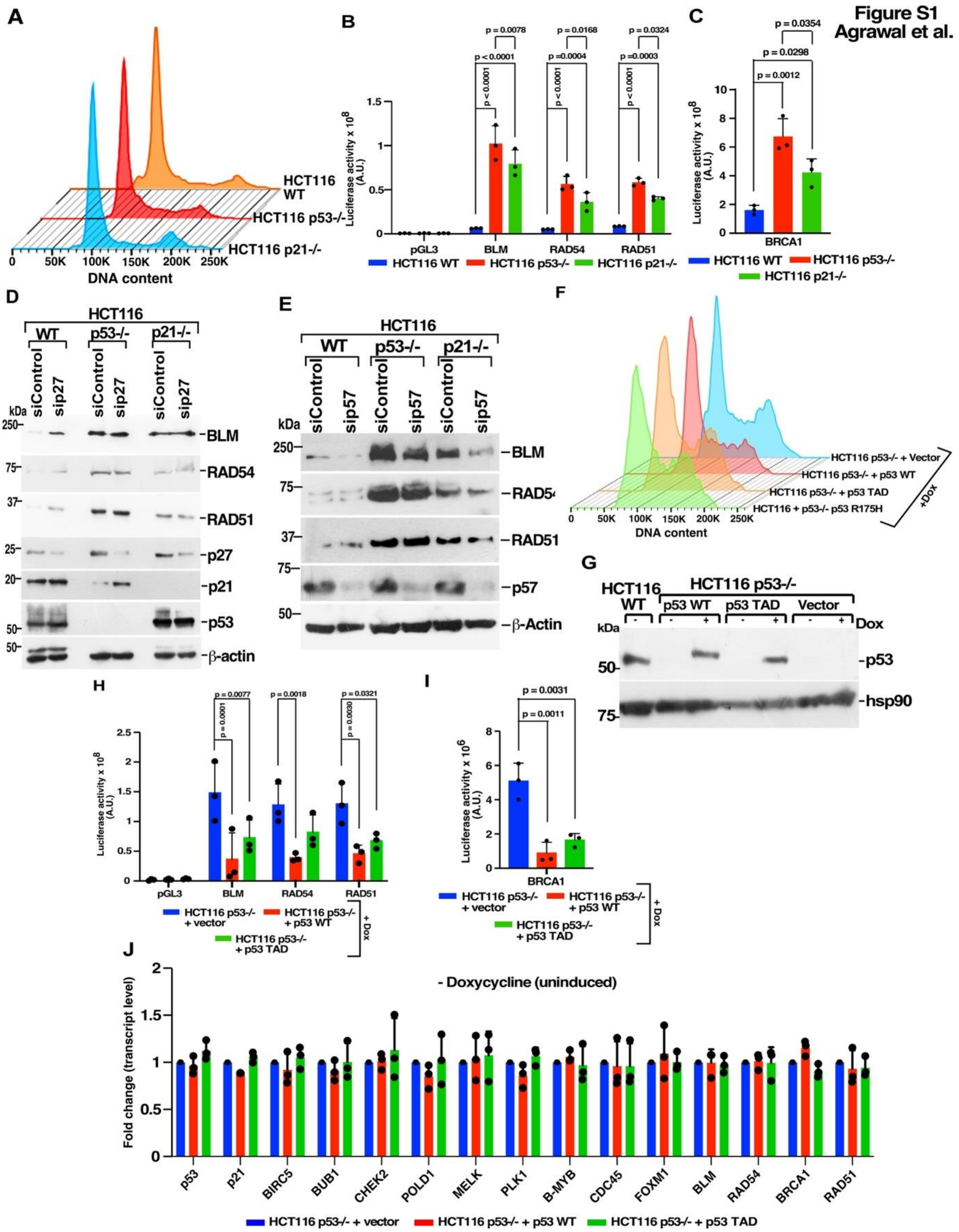

Appendix Figure S1 legend

A, F. Cell cycle profile of asynchronously growing (A) HCT116 p53+/+, HCT116 p53-/-, HCT116 p21-/- cells, (F) HCT116 p53-/- cells expressing either vector, p53 WT, p53 TAD mutant, p53 R175H. Cells were stained with propidium iodide and analysed by flow cytometry. Three biological replicates were carried out, and the same result was obtained in all the experiments.

B, C. p21-independent repression of DREAM complex target promoter activity. Luciferase assay was carried out in asynchronously growing HCT116 WT, HCT116 p53-/-, HCT116 p21-/- cells. The cells were transfected with constructs encoding (B) BLM luciferase, RAD54 luciferase, RAD51 luciferase (C) BRCA1 luciferase. CMV-β-galactosidase was added in all cases. Lysates made were used for luciferase and β-galactosidase assays. Mean ± SD. A.U. is absolute units after normalization with β-galactosidase activity. The data is from three biological replicates.

D, E. Effect of the lack of CDK inhibitors, p27 and p57. HCT116 WT, HCT116 p53-/-, and HCT116 p21-/- cells were transfected with either siControl and (D) sip27 or (E) sip57. Lysates were prepared, and immunoblotting was performed with the indicated antibodies. Three biological replicates were carried out and the same result was obtained.

G. Comparison of the p53 levels expressed in HCT116 WT and Dox-regulated stable lines expressing either Flag-p53 WT or Flag-p53 TAD. Lysates were prepared from asynchronously growing HCT116 cells or Flag-p53 WT or Flag-p53 TAD cells (in both ±Dox conditions). Immunoblotting was performed with the indicated antibodies. Three biological replicates were carried out, and the same result was obtained.

H, I. p53 TAD mutant repress DREAM target promoter activity. Same as (B, C) except luciferase was carried out in HCT116 p53-/- cells expressing either vector, p53 WT or p53 TAD (+Dox). The constructs used were (H) BLM luciferase, RAD54 luciferase, and RAD51 luciferase (I) BRCA1 luciferase. Three biological replicates were carried out, and the same result was obtained.

J. p53 WT and p53 TAD mutant do not repress DREAM complex targets when not expressed. RNA was isolated from stable lines generated in HCT116 p53-/- cells expressing either the vector, p53 WT or p53 TAD mutant and grown in the absence of Doxycycline. RT-qPCR of the indicated genes was performed. The data is from three biological replicates.

Figure S2
Agrawal et al.

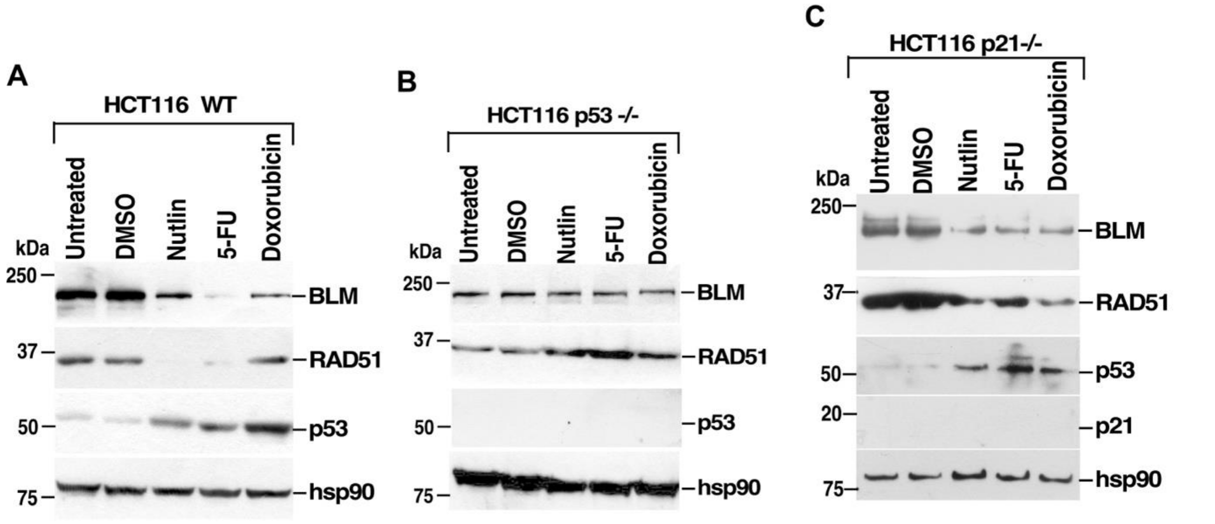

Figure S2 legend

A-C. DREAM complex targets repressed in response to DNA damage in the absence of p21. (A) HCT116 p53+/+ (B) HCT116 p53 -/- (C) HCT116 p21-/- cells were treated with the indicated DNA damaging agents for 24 hours. Whole-cell extracts were made post-treatment, and immunoblotting was carried out with the indicated antibodies. Three biological replicates were carried out and the same result was obtained.

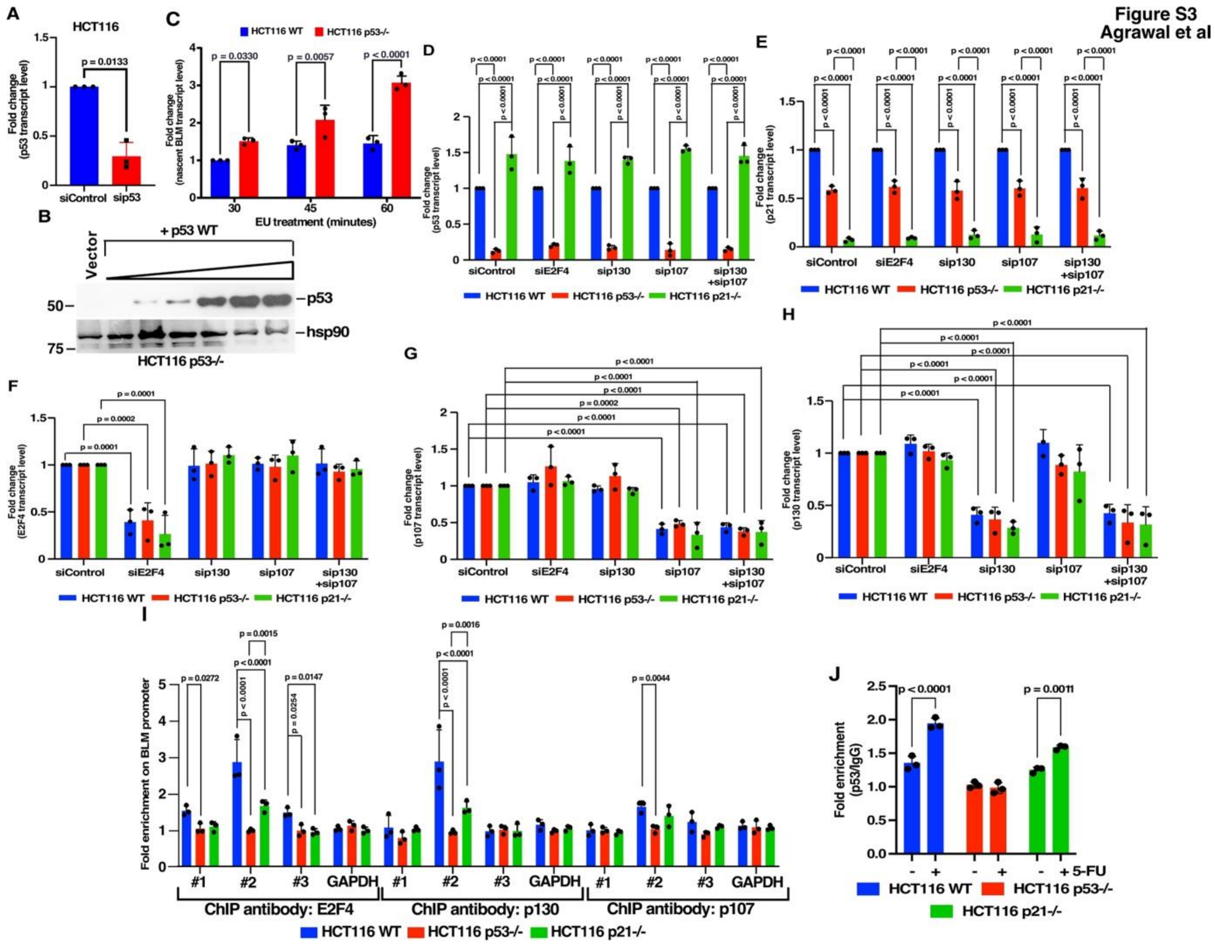

Appendix Figure S3 legend

A. Ablation of p53 in HCT116 WT cells. HCT116 cells were transfected with either siRNA control or siRNA p53. RNA was isolated 24 hours post-transfection and the levels of the p53 transcript were determined by RT-qPCR. Mean ± SD. The data is from three biological replicates.

B. Overexpression of the increasing amount of p53 in HCT116 p53 -/- cells. For luciferase assays, HCT116 p53 -/- cells were transfected with the indicated increasing amount of p53 WT DNA. Immunoblotting with the lysates prepared for luciferase assay was carried out with the indicated antibodies. Three biological replicates were carried out and the same result was obtained.

C. BLM nascent mRNA level increases in the absence of p53. HCT116 p53+/+, HCT 116 p53-/- cells were labelled with 0.5mM EU for 30 minutes, 45 minutes and 60 minutes. Total RNA was collected, EU-labelled RNA enriched and the levels of BLM nascent transcripts were

determined by RT-qPCR. Mean ± SD. Cortactin was used as an internal control. The data is from three biological replicates.

D-H. Ablation of DREAM complex members. In HCT116 p53+/+, HCT116 p53-/-, HCT116 p21-/- cells siRNA mediated ablation of E2F4, p130 and p107 was carried out. RNAs were isolated from each condition and the levels of (D) p53, (E) p21, (F) E2F4, (G) p107 and (H) p130 transcripts were determined by RT-qPCR. Cortactin was used as an internal control. Mean ± SD. The data is from three biological replicates.

I. DREAM complex members bind to the #2 E2F binding site in the BLM promoter. E2F4, p130, p107 ChIP was carried out using HCT116 WT, HCT116 p53-/-, HCT116 p21-/- cells. Recruitment of E2F4, p130 and p107 was determined by ChIP-qPCR analysis using three sets of primers encompassing putative E2F binding sites on BLM promoter. Recruitment to GAPDH promoter was used as a control. Mean ± SD. The data is from three biological replicates.

J. p53 binds to the #2 site on the BLM promoter in response to DNA damage in the absence of p21. Recruitment of p53 to the #2 site on BLM promoter was determined by ChIP-qPCR in HCT116 WT, HCT116 p53-/- and HCT116 p21-/- cells with and without treatment of 5-FU. Mean ± SD. The data is from three biological replicates.

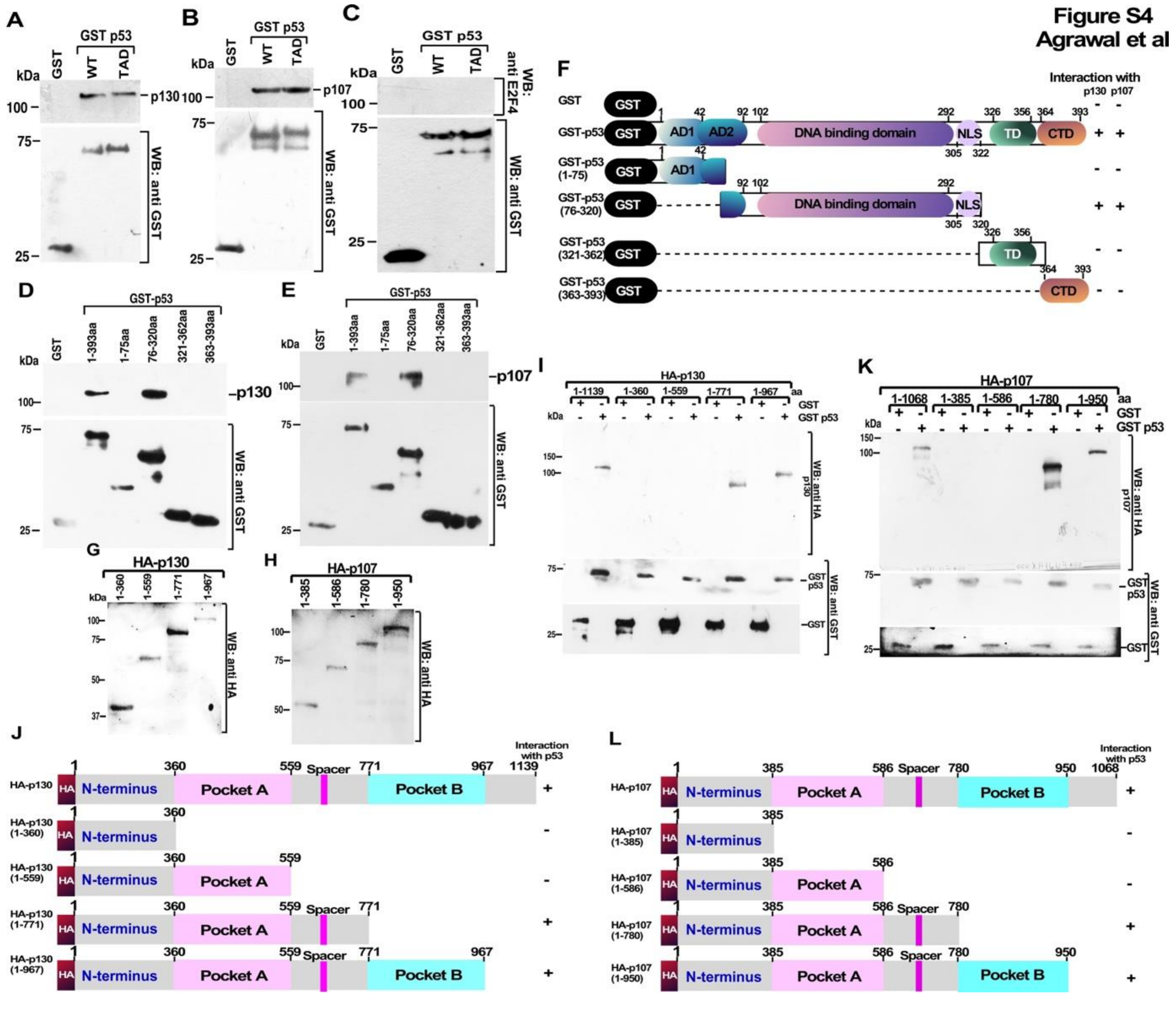

Appendix Figure S4 legend

A-C. p130 and p107 interact with p53 WT and p53 TAD mutant. Interaction assays were carried out using *in vitro*-translated (A) p130, (B) p107, (C) E2F4 proteins and GST-tagged recombinant p53 variants (Bottom). (Top) Interactions were determined by immunoblotting with anti-p130/p107/E2F4 antibodies. Three biological replicates were carried out, and the same result was obtained.

D-E. p130 and p107 interact with p53 (75-320aa) domain. Interaction assays were carried out using *in vitro*-translated (D) p130, (E) p107 and GST-tagged recombinant p53 and its domain mutants (1-75aa, 76-320aa, 321-362aa, 363-393aa) (Bottom). (Top) Interactions were determined by immunoblotting with anti-p130/p107 antibodies. Three biological replicates were carried out and the same result was obtained.

F. Schematic diagram showing the interaction of p130 and p107 with p53 domains. The GST constructs are shown on the left, and their interaction with full-length p130 and p107 is summarized on the right. AD1, activation domain 1; AD2, activation domain 2; NLS, nuclear localization signal; TD, tetramerization domain; + indicates detectable interaction; − indicates undetectable interaction. The numbers indicate the amino acids of human p53.

G, H. Equalization of different fragments of p130 and p107. *In vitro* transcription and translation of different fragments of (G) p130 (1-360aa, 1-559aa, 1-771aa, 1-967aa) and (H) p107 (1-385aa, 1-586aa, 1-780aa, 1-950aa) were carried out and subjected to western blot analysis with anti-HA antibody.

I, K. p53 interacts with p130 (559-771aa) and p107 (586-780aa). Interaction assays were carried out using *in vitro*-transcribed and translated (I) p130, (K) p107 fragments and GST-tagged recombinant p53 (Bottom two blots). (Top) The interactions were determined by immunoblotting with anti-HA antibodies. Three biological replicates were carried out, and the same result was obtained.

J, L. Schematic diagram showing the interaction of GST-p53 with the p130/p107 domains. The HA-tagged constructs of (J) p130 and (L) p107 are shown on the left, and the extent of their interaction with GST-p53 WT is summarized on the right where + indicates detectable interaction; − indicates undetectable interaction. The N-terminus, Pocket A, Pocket B and the Spacer domains in p130/p107 have been indicated.

Figure S5
Agrawal et al.

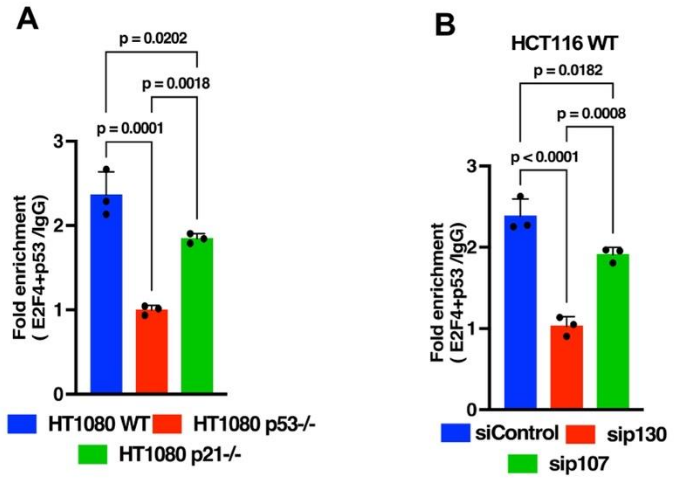

Appendix Figure S5 legend

A. E2F4 and p53 are co-recruited onto the BLM promoter in the absence of p21. Re-ChIP assay was carried out using HT1080 p53 WT, HT1080 p53-/-, HT1080 p21-/- cells using anti-E2F4 as the 1st antibody and anti-p53 as the 2nd antibody. Co-recruitment of E2F4 and p53 on the #2 E2F site on the BLM promoter was determined by ChIP-qPCR analysis. Mean ± SD. The data is from three biological replicates.

B. E2F4 and p53 are co-recruited onto the BLM promoter in the presence of p130. In HCT116 WT cells siRNA-mediated ablation of p130 and p107 was performed. Re-ChIP assay was carried out using anti-E2F4 as the 1st antibody and anti-p53 as the 2nd antibody. Co-recruitment of E2F4 and p53 on the #2 E2F site on the BLM promoter in the presence and absence of p130 / p107 was determined by ChIP-qPCR analysis. Mean ± SD. The data is from three biological replicates.

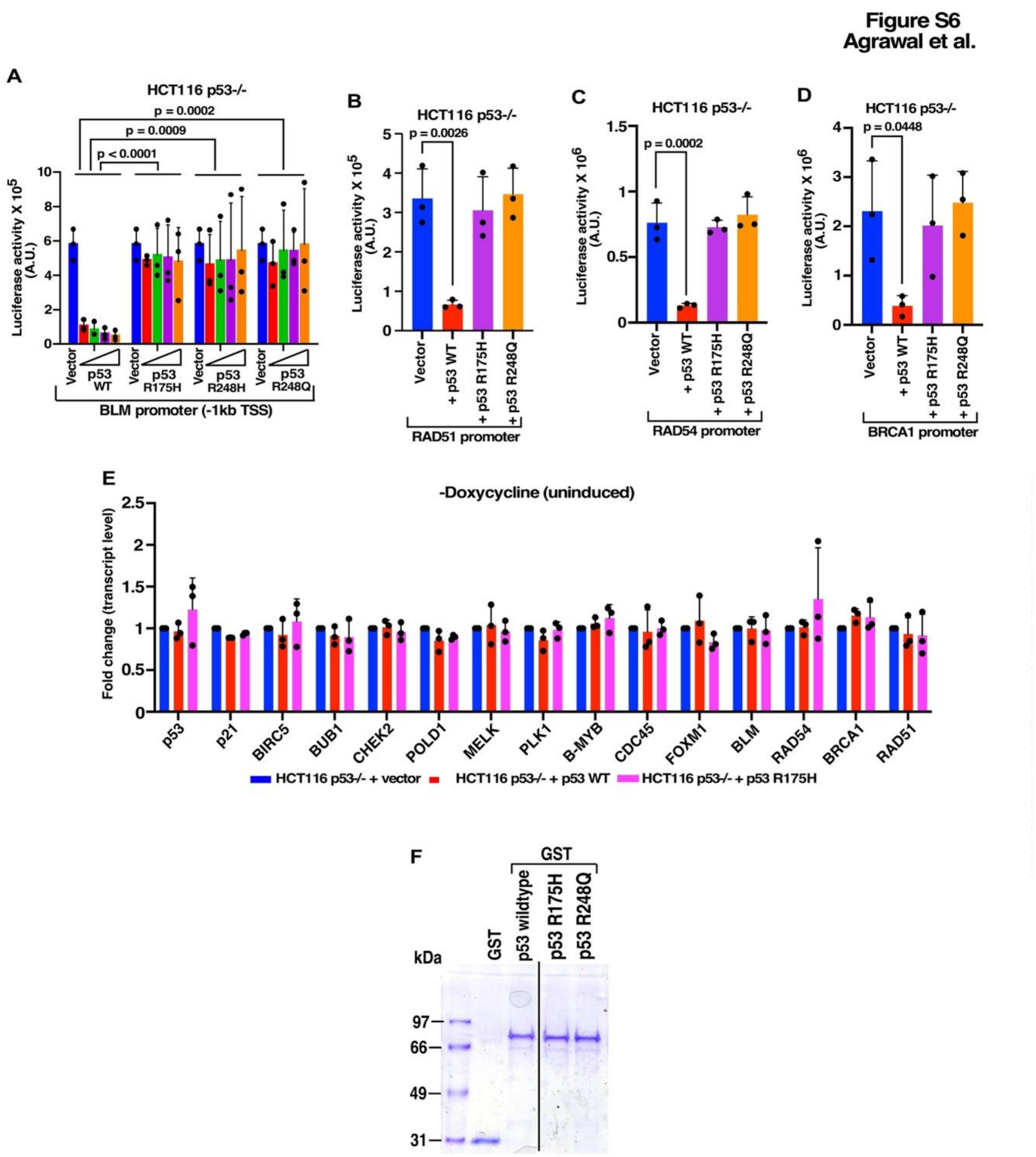

Appendix Figure S6 legend

A. p53 hotspot mutants cannot repress BLM promoter. Increasing amounts (50, 200, 500 and 1000ng) of p53 WT and p53 mutants (R175H, R248H and R248Q) were overexpressed in HCT116 p53-/- cells with 640bp pGL3-BLM minimal promoter and CMV-β-galactosidase. Lysates made were used for luciferase and β-galactosidase assays. Mean ± SD. A.U. is absolute

units after normalization with β-galactosidase activity. The data is from three biological replicates.

B-D. p53 hotspot mutants cannot repress DREAM complex promoters. p53 WT and p53 mutants (R175H, R248Q) were overexpressed in HCT116 p53-/- cells with (B) pGL3-RAD51, (C) pGL3-RAD54, and (D) pGL3-BRCA1 promoter constructs and CMV-β-galactosidase. Lysates made were used for luciferase and β-galactosidase assays. Mean ± SD. A.U. is absolute units after normalization with β-galactosidase activity. The data is from three biological replicates.

E. p53 R175H cannot repress DREAM complex targets. RNA was isolated from stable lines generated in HCT116 p53-/- cells expressing either the Vector, p53 WT or p53 R175H and grown in the absence of Doxycycline. RT-qPCR of the indicated genes was performed. Mean ± SD. The data is from three biological replicates.

F. Purity of recombinant p53 variants used in the in vitro interaction assays. Coomassie gels showing the purity of recombinant GST, p53 WT, p53 R175H, p53 R248Q.

Figure S7
Agrawal et al

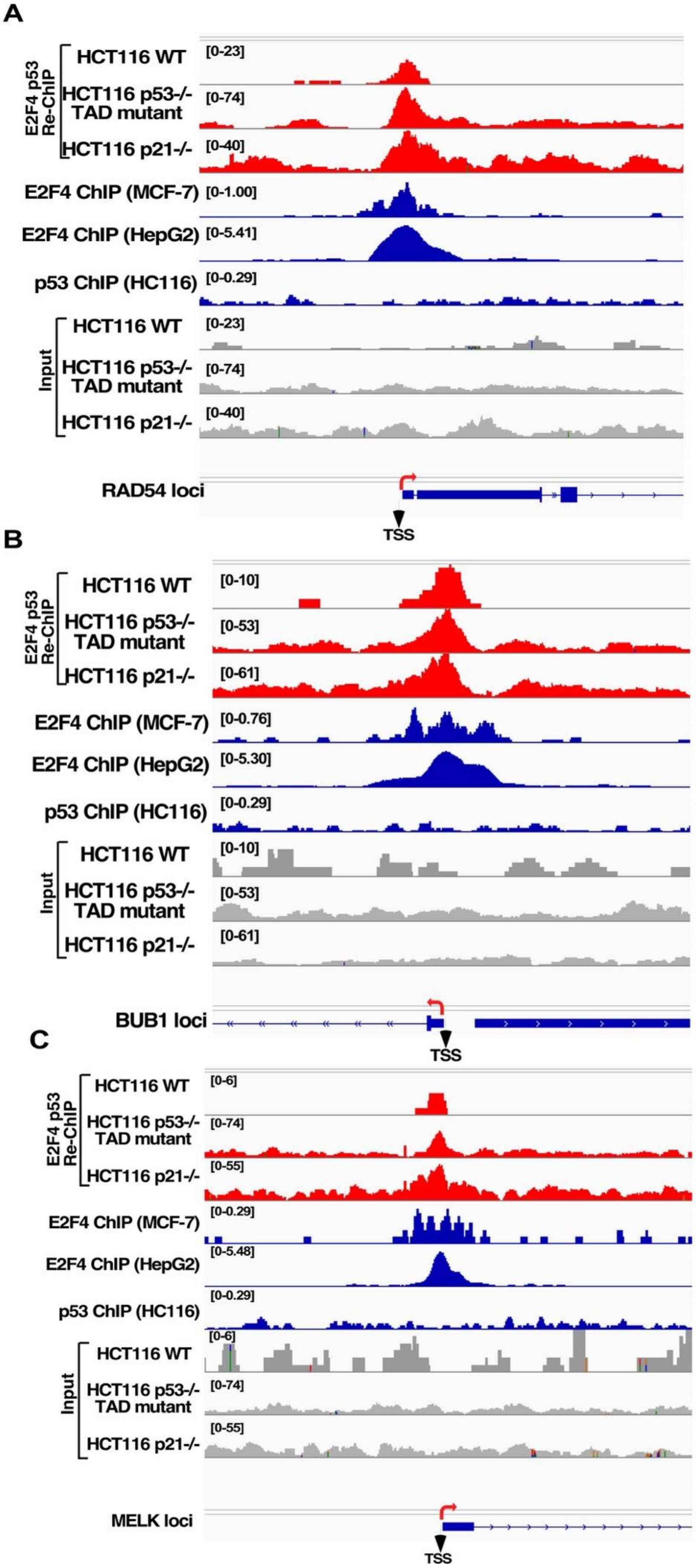

Appendix Figure S7 legend

A-C. E2F4, p53 Re-ChIP data overlap with E2F4 ChIP seq data. IGV browser tracks from E2F4 p53 Re-ChIP seq peaks (HCT116 p53 WT, HCT116 p21-/-, HCT116 p53-/- p53 TAD mutant (+Dox condition), E2F4 ChIP seq peaks (HepG2, MCF7), p53 ChIP peak and respective input signals. Upstream of TSS for (A) RAD54, (B) BUB1, and (C) MELK promoters have been depicted.

Appendix Table

Appendix Table S1: Details of Statistical Analysis

Figure number	Statistical Analysis performed	Type of Test	Software used
Figure 1A	2way ANOVA	Sidak's multiple comparisons test	GraphPad Prism
Figure 1F	2way ANOVA	Sidak's multiple comparisons test	GraphPad Prism
Figure 2A	Paired t-test	N/A	GraphPad Prism
Figure 2E	2way ANOVA	Tukey's multiple comparisons test	GraphPad Prism
Figure 2G	Ordinary one-way ANOVA	Dunnett's multiple comparisons test	GraphPad Prism
Figure 2H	2way ANOVA	Tukey's multiple comparisons test	GraphPad Prism
Figure 2I	Ordinary one-way ANOVA	Tukey's multiple comparisons test	GraphPad Prism
Figure 2J	Ordinary one-way ANOVA	Dunnett's multiple comparisons test	GraphPad Prism
Figure 2K	Ordinary one-way ANOVA	Tukey's multiple comparisons test	GraphPad Prism
Figure 3K	2way ANOVA	Sidak's multiple comparisons test	GraphPad Prism
Figure 3L	Ordinary one-way ANOVA	Tukey's multiple comparisons test	GraphPad Prism
Figure 4B	2way ANOVA	Tukey's multiple comparisons test	GraphPad Prism
Figure 4F	Ordinary one-way ANOVA	Tukey's multiple comparisons test	GraphPad Prism
Figure 4G	Ordinary one-way ANOVA	Tukey's multiple comparisons test	GraphPad Prism

Figure S1B	2way ANOVA	Tukey's multiple comparisons test	GraphPad Prism
Figure S1C	Ordinary one-way ANOVA	Tukey's multiple comparisons test	GraphPad Prism
Figure S1H	2way ANOVA	Sidak's multiple comparisons test	GraphPad Prism
Figure S1I	Ordinary one-way ANOVA	Tukey's multiple comparisons test	GraphPad Prism
Figure S1J	2way ANOVA	Tukey's multiple comparisons test	GraphPad Prism
Figure S3A	Paired t-test	N/A	GraphPad Prism
Figure S3C	2way ANOVA	Sidak's multiple comparisons test	GraphPad Prism
Figure S3D	2way ANOVA	Tukey's multiple comparisons test	GraphPad Prism
Figure S3E	2way ANOVA	Tukey's multiple comparisons test	GraphPad Prism
Figure S3F	2way ANOVA	Tukey's multiple comparisons test	GraphPad Prism
Figure S3G	2way ANOVA	Tukey's multiple comparisons test	GraphPad Prism
Figure S3H	2way ANOVA	Tukey's multiple comparisons test	GraphPad Prism
Figure S3I	2way ANOVA	Tukey's multiple comparisons test	GraphPad Prism
Figure S3J	2way ANOVA	Sidak's multiple comparisons test	GraphPad Prism
Figure S5A	Ordinary one-way ANOVA	Tukey's multiple comparisons test	GraphPad Prism
Figure S5B	Ordinary one-way ANOVA	Tukey's multiple comparisons test	GraphPad Prism
Figure S6A	2way ANOVA	Tukey's multiple comparisons test	GraphPad Prism

Figure S6B	Ordinary one-way ANOVA	Dunnett's multiple comparisons test	GraphPad Prism
Figure S6C	Ordinary one-way ANOVA	Dunnett's multiple comparisons test	GraphPad Prism
Figure S6D	Ordinary one-way ANOVA	Dunnett's multiple comparisons test	GraphPad Prism
Figure S6E	2way ANOVA	Sidak's multiple comparisons test	GraphPad Prism

Appendix Table S2: PCR primers sequence

Primers		
**RT-qPCR primers**		
**Gene**	**Forward Primer Sequence (5'-3')**	**Reverse Primer Sequence (5'-3')**
BLM	AGACAGGATTCTCTGCC ACCAGGA	TGGTGTTTCAGCCCAGTT GCT
RAD54	ATGAGGTTGGGAAATGG C	GAGGACTCCAACATGAA G
BRCA1	GTGAGTCAGTGTGCAGC ATTTGAA	TCTATGCTTGTTTCCCGA CTGTGG
RAD51	TCTCTGGCAGTGATGTCC TGGA	TAAAGGGCGGTGGCACT GTCTA
p107	CTCTTTGCCTATAGCTCA CCTC	GCGGATCACCACCTCAAT AA
p130	TACACGCTGGAGGGAAA T	TTCCACTGTCCCTTTGCT TAC
p21	CTCACATCCTCCTCCTTC TTCAG	CACACACAGAATCTGAC TCCC
Actin (Control)	AGGCACCAGGGCGTGAT	GCCCACATAGGAATCCTT CTGAC
CTTN (Control)	GCCGACCGAGTAGACAA G	GTATTTGCCGCCGAAACC
MDM2	TGTTTGGCGTGCCAAGCT TCTC	CACAGATGTACCTGAGTC CGATG
Cyclin G	CCTTCTGTGTTGGCATTG TCTATC	CAAGCTCTTGCCAGAAG GTCAG
Bax	TCAGGATGCGTCCACCA AGAAG	TGTGTCCACGGCGGCAA TCATC
BIRC5	CCACTGAGAACGAGCCA GACTT	GTATTACAGGCGTAAGCC ACCG
BUB1	GCTCTGTCAGCAGACTTC CTTC	CAGCAGATGTGAAGTCT CCTGG
Cyclin B2	CAACCAGAGCAGCACAA GTAGC	GGAGCCAACTTTTCCATC TGTAC

Cyclin A2	CTCTACACAGTCACGGG ACAAAG	CTGTGGTGCTTTGAGGTA GGTC
Cyclin B1	GACCTGTGTCAGGCTTTC TCTG	GGTATTTTGGTCTGACTG CTTGC
Chk2	GACCAAGAACCTGAGGA GCCTA	GGATCAGATGACAGCAG GAGTTC
POLD1	ACTACACGGGAGCCACT GTCAT	GCGTGGTGTAACACAGG TTGTG
RAD18	GTATGCATGGGACAGGA AGATAA	GAGGAATTGGAACCTGA CAGAG
MELK1	TCCTGTGGACAAGCCAG TGCTA	GGGAGTAGCAGCACCTG TTGAT
PLK1	GCACAGTGTCAATGCCTC CAAG	GCCGTACTTGTCCGAATA GTCC
B MYB	CACCAGAAACGAGCCTG CCTTA	CTCAGGTCACACCAAGC ATCAG
CDC45	TGGATGCTGTCCAAGGA CCTGA	CAGGACACCAACATCAG TCACG
ORC1	CTCAAGCCTAGAACGCC ACGTT	GGAAGAGACTCAGGTAC AGCAG
E2F2	CTCTCTGAGCTTCAAGCA CCTG	CTTGACGGCAATCACTGT CTGC
FOXM1	TCTGCCAATGGCAAGGT CTCCT	CTGGATTCGGTCGTTTCT GCTG
p27	ATAAGGAAGCGACCTGC AACCG	TTCTTGGGCGTCTGCTCC ACAG
HDAC2	TAAATCCAAGGACAACA GTGG	GGTGAGACTGTCAAATTC AGG
p53 (3’UTR**)**	TGCAATAGGTGTGCGTCA GAA	CCCCGGGACAAAGCAAA
p53 (Coding region**)**	GCGAGCACTGCCCAACA A CA	GGATCTGAAGGGTGAAA T ATTCT
E2F4	TGCAGAAGTCCAGGGAA TG	TGAGCTCACCACTGTCCT TG
HDAC1	GGAAATCTATCGCCCTCA CAA	TGCTGTACTCCGACATGT TATC

HDAC6	AGCGGAGGTAAAGAAGAAAGG	CTTCAGCCTCAAGGTTCAGAT
**Cloning primers**		
pGL3 BRCA1 promoter -497 to +274bp with respect to TSS	CTAGCTAGCTAGACACTGTGGCGAAGACCTTT	CCCAAGCTTGGGTTCCCTCGCGACCTACAAAC
pGL3 RAD51 promoter -441 to +267 with respect to TSS	CTAGCTAGCTAGATGCATGCCGGGAGATGTAG	CCCAAGCTTGGGTCACACACTCACCTCGGTCC
pGL3 RAD54 promoter (-557 to +154) with respect to TSS	CTAGCTAGCTAGATGCATGCCGGGAGATGTAG	CCCAAGCTTGGGTCACACACTCACCTCGGTCC
pGL3 BLM promoter (-461bp to +179bp) with respect to TSS (referred as BLM 640bp minimal promoter)	AATCGAGCTCGTGAGGGGTACGGGTGAAACAG	CCGCTCGAGCGGAGGAAACGGAAGAACCCGAG
pcDNA3.1 hygro(+) E2F4 (1-414aa)	CCCAAGCTTGGGGCCACCATGGCGGAGGCCGGGCCACAG	GATCCGCGTCAGAGGTTGAGAACAGGCAC
pLVX-TetOne-Puro-Flag p53 / pLVX-TetOne-Puro-Flag p53 (L22Q, W23S, W53Q, F54S)	CCGGAATTCCGGGCCACCATGGACTACAAAGACCATGACGGTGATTATAAAGATCATGACATCGATTACAAGGATGACGATGACAAGGAGGAGCCGCAGTCAGATCCT	CGCGGATCCGCGTCAGTCTGAGTCAGGCCCTTC
pGL3 BLM promoter -3499 to +64 w.r.t TSS (referred as BLM promoter - 3.5kb)	AATCGAGCTCGTCTGGCAGATCGCCTAAGG	CCGCTCGAGCGGCCTAGCGGACGGAACCAGGATCC
**Primers to generate site-directed mutagenesis**		
BLM promoter WT E2F4 site to mutated E2F4 site (#1)	GAATAGGCAAGCTTCCGGAAGGAAGTGAGCCAGGGCTTG	CAAGCCCTGGCTCACTTCCTTCCGGAAGCTTGCCTATTC
BLM promoter WT E2F4 site to mutated E2F4 site (#2)	GCGGCCGTGGTTGCGGCGAAGGAAGTTTGGATCCTGGTTC	GAACCAGGATCCAAACTTCCTTCGCCGCAACCACGGCCGC

BLM promoter WT E2F4 site to mutated E2F4 site (#3)	CCAGCAGCCTGAGGGGAAGGGAACAGATGTCCGAGTGCG	CGCACTCGGACATCTGTTCCCTTCCCCTCAGGCTGCTGG
BLM promoter WT E2F4 site to mutated E2F4 site (#4)	CCGGACTCTGATTGGGCCC	GGAGGGACGCGTATCTCCGGGGCCCAATCAGAGTCCGG
**ChIP and Re-ChIP primers**		
BLM promoter E2F4 binding site #1	GCCAATCGGAATAGGCAAGC	AGCAGGGCTAGATCAATGCG
BLM promoter E2F4 binding site #2	ACAGTATTGGTCGGCTTCCC	TCGCACGCAGACTCCTA
BLM promoter E2F4 binding site #3	CAAAGACCCAACTAGCTCCG	GAGGGACGCGTATCTCCAAAG
BLM promoter E2F4 binding site #4	GAGATACGCGTCCCTCCCG	ACCAATACTGTCGCACTCGG
GAPDH promoter	GCAGCCCCTTCATACCCTCACGT	GAGCCACACCATCCTAGTTGC
**DNA affinity purification primers**		
BLM promoter (732bp)	GAGGGGTACGGGTGAAAC AG (BLM promoter specific primer)	Biotin – CGCCGGGCCTTTCT TTATGT (Within the pGL3 vector body after the MCS ends)
