# Supplementary figures and images for: p53 regulates DREAM complex-mediated repression in a p21-independent manner (part 1 of 2)
Source: EMBO J. 2025 Mar 4;44(8):2279–97. doi: 10.1038/s44318-025-00402-7 (PMC12000331; doi:10.1038/s44318-025-00402-7)

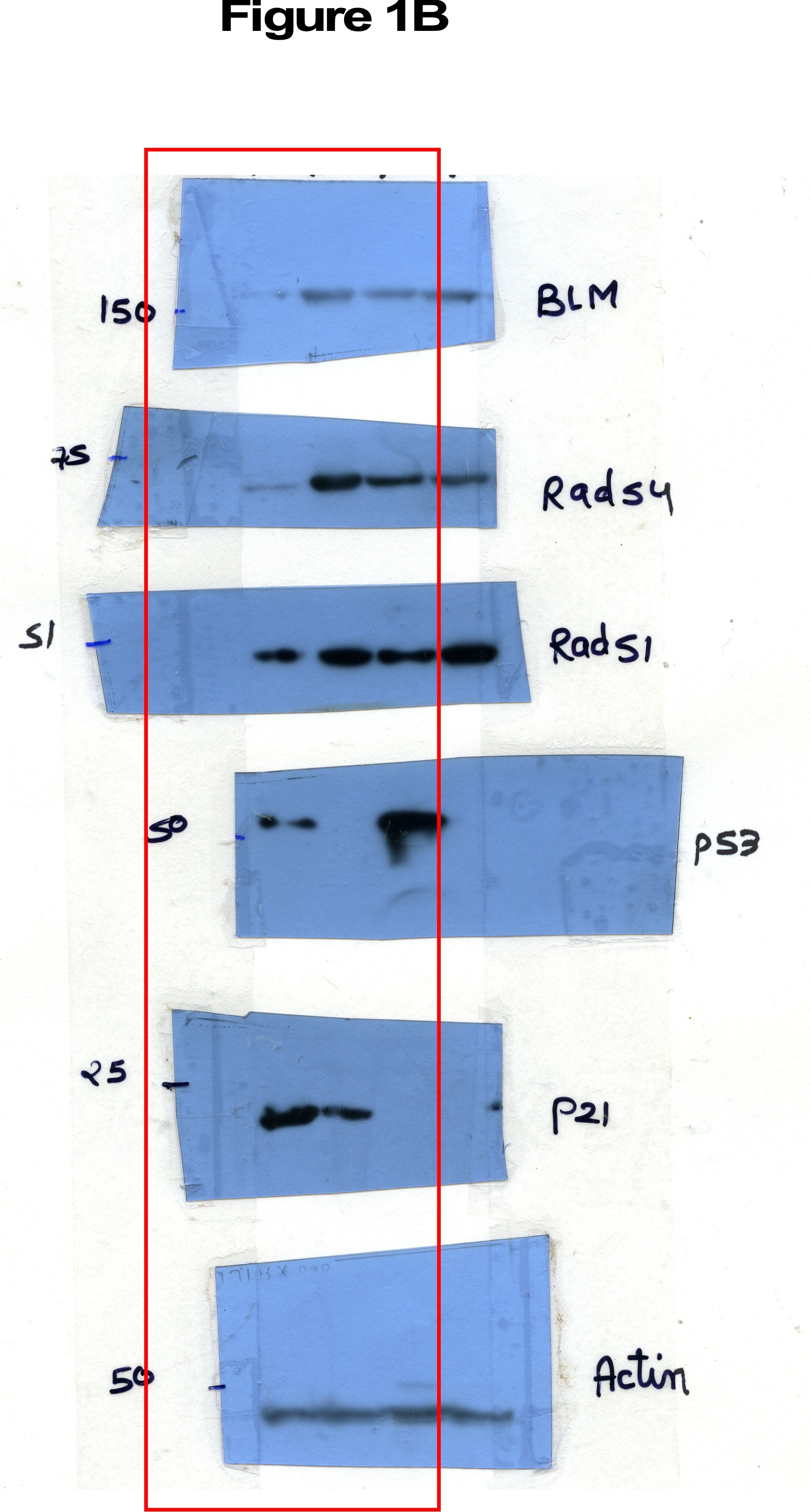

Supplement: Supplementary file 6 — Source data Fig. 1 [file 44318_2025_402_MOESM6_ESM.zip › SD Figure 1/1B/1B Western Replicate#1 (in publication).jpg]

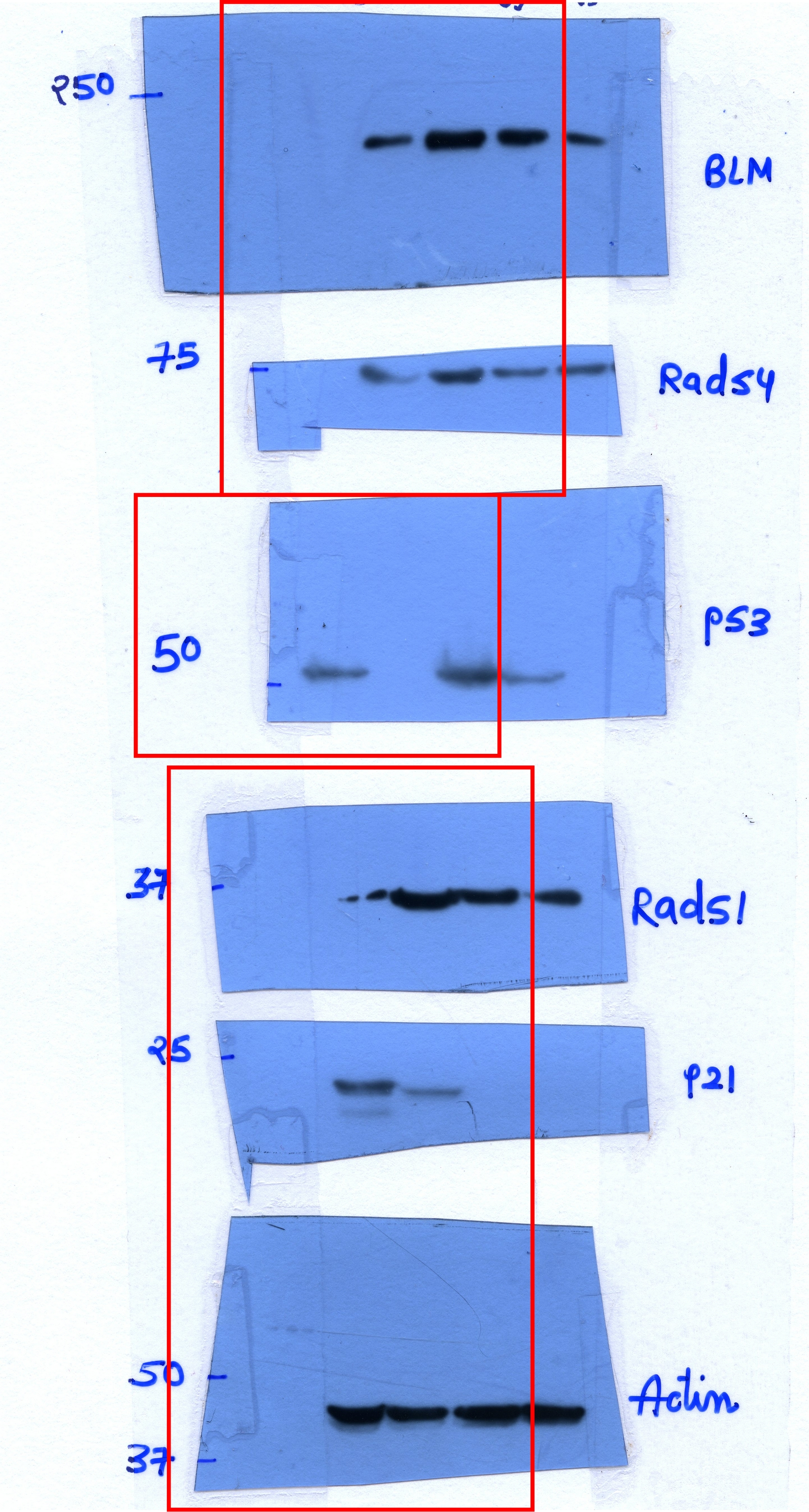

Supplement: Supplementary file 6 — Source data Fig. 1 [file 44318_2025_402_MOESM6_ESM.zip › SD Figure 1/1B/1B Western Replicate#2.jpg]

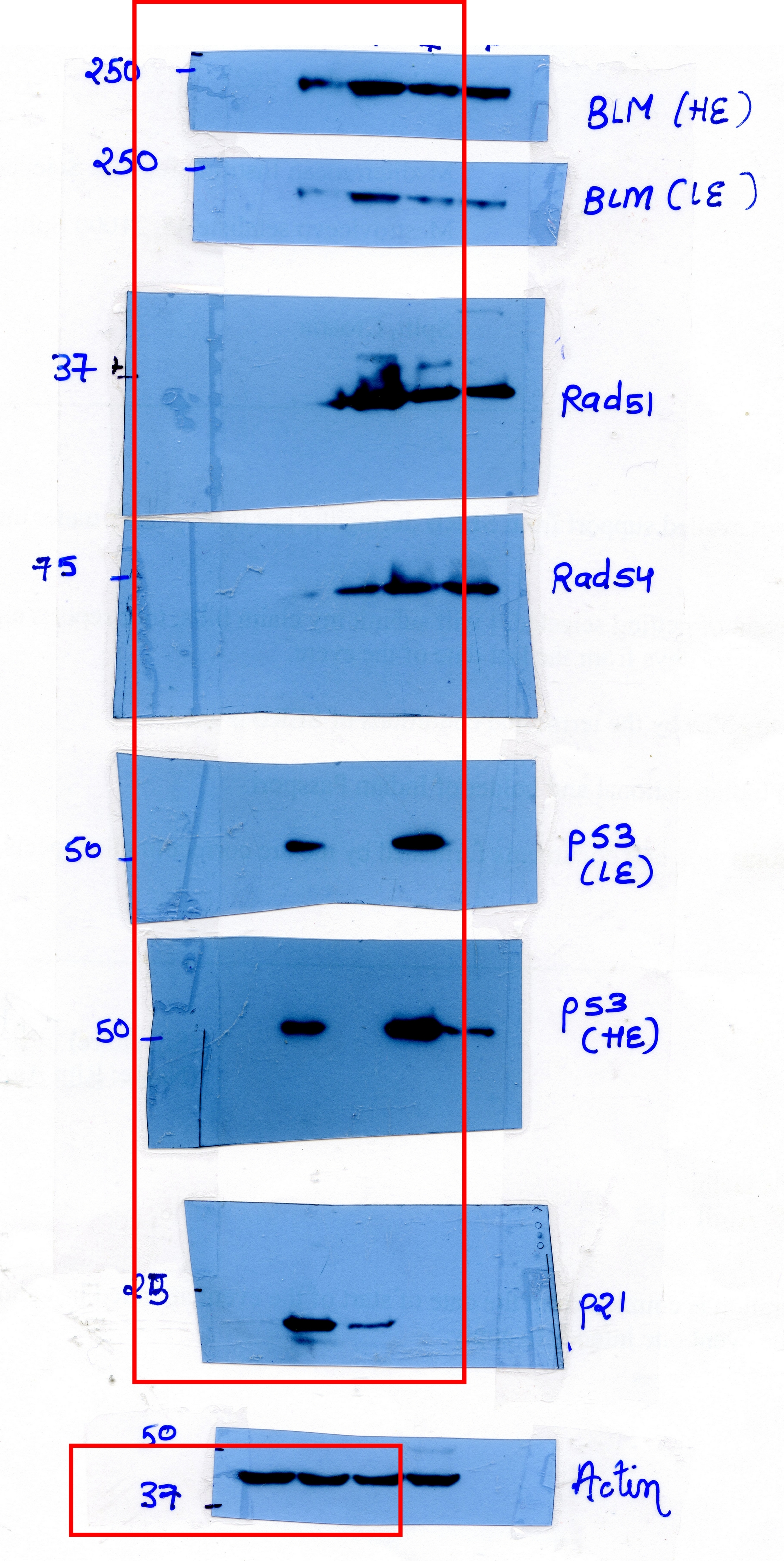

Supplement: Supplementary file 6 — Source data Fig. 1 [file 44318_2025_402_MOESM6_ESM.zip › SD Figure 1/1B/1B Western Replicate#3.jpg]

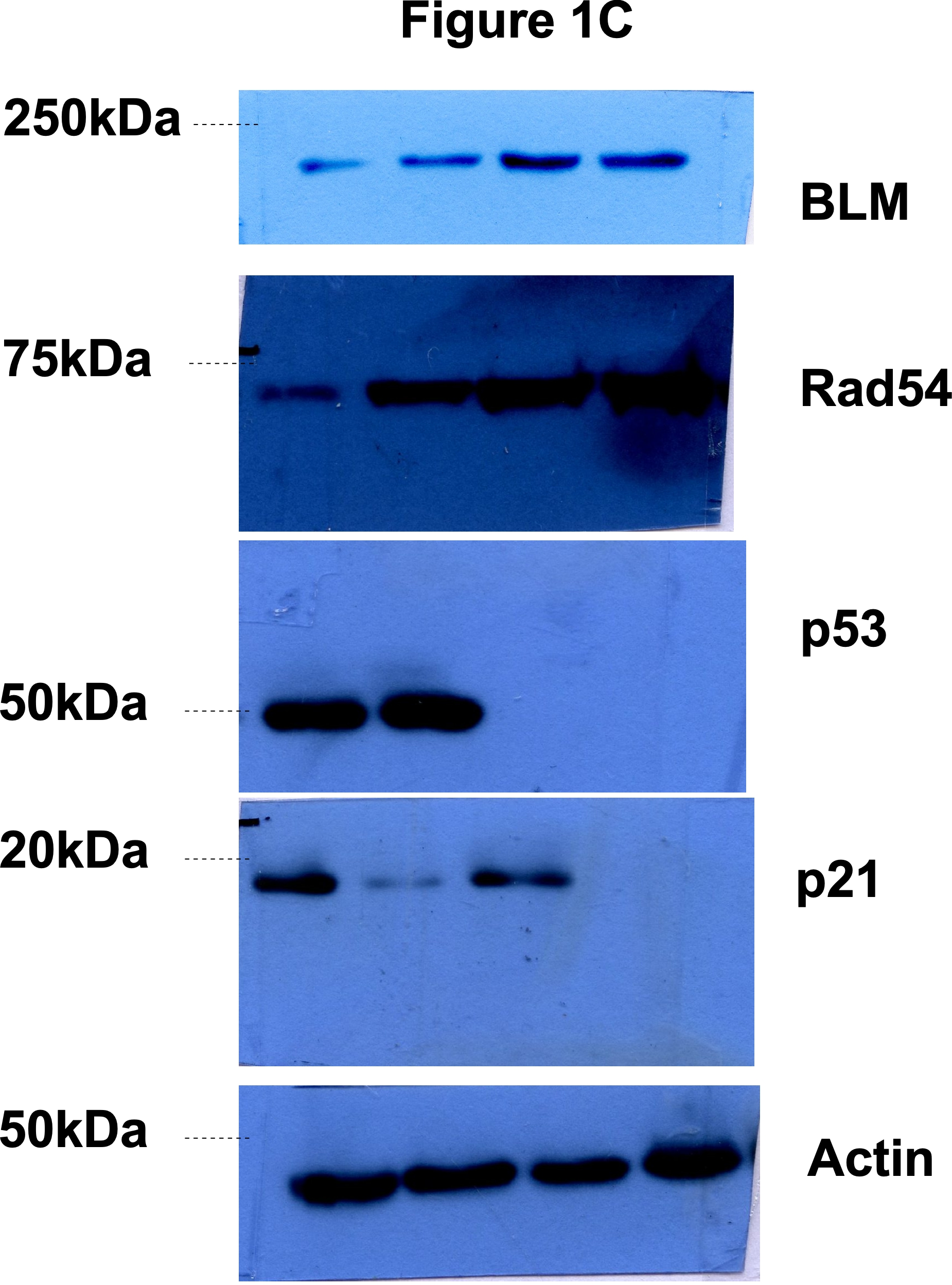

Supplement: Supplementary file 6 — Source data Fig. 1 [file 44318_2025_402_MOESM6_ESM.zip › SD Figure 1/1C/1C Western Replicate#1 (in publication).jpg]

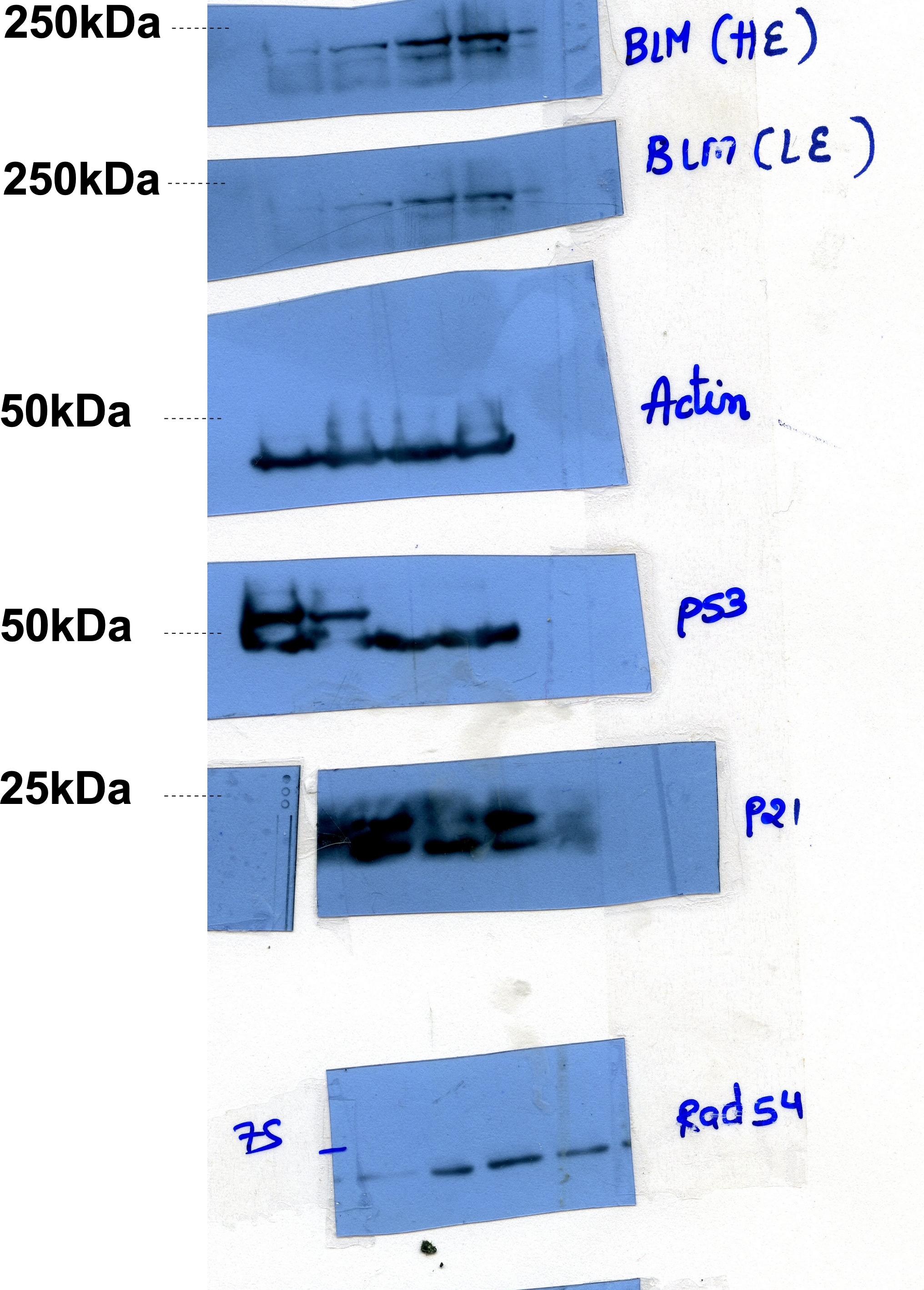

Supplement: Supplementary file 6 — Source data Fig. 1 [file 44318_2025_402_MOESM6_ESM.zip › SD Figure 1/1C/1C Western Replicate#2.jpg]

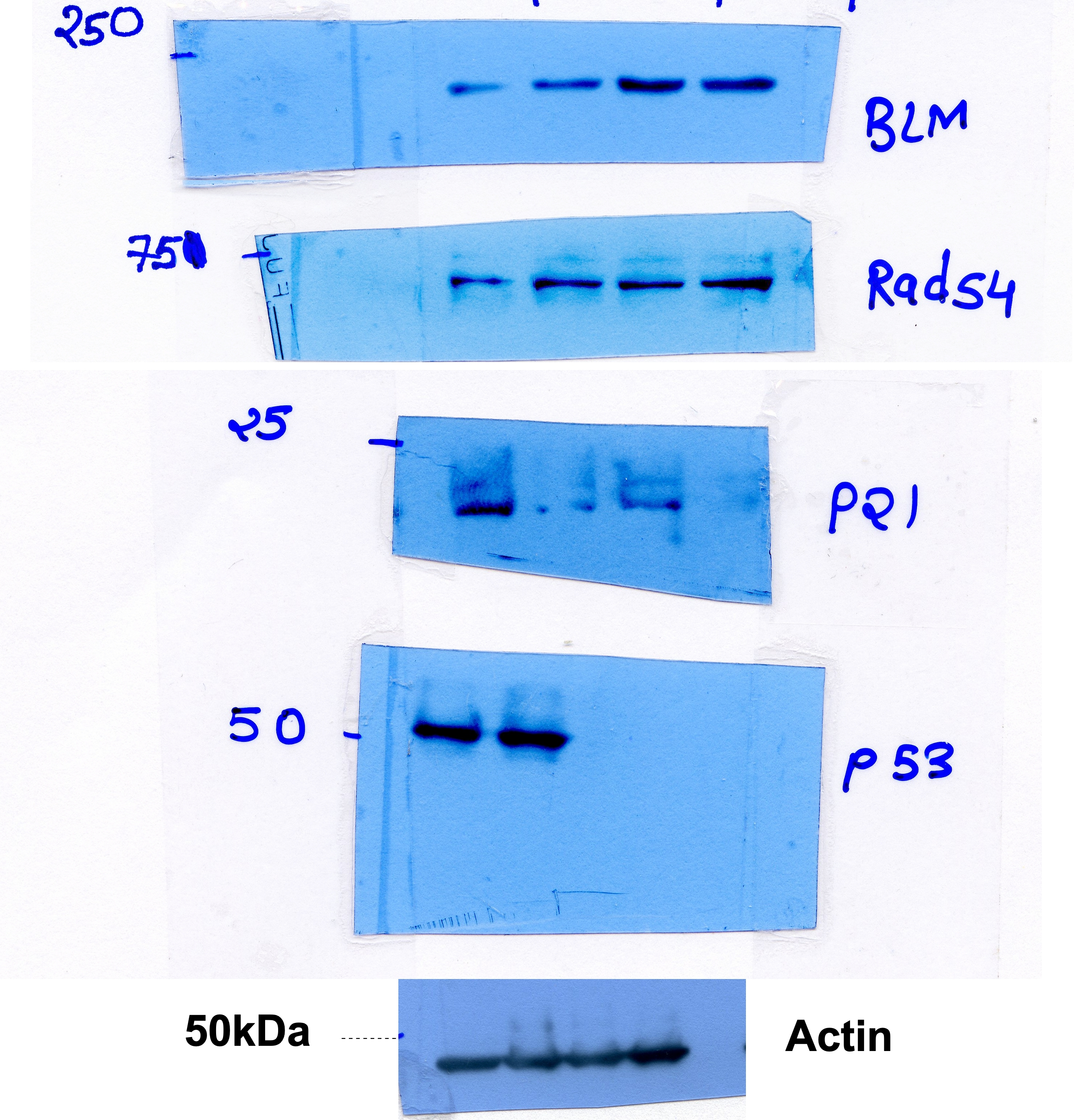

Supplement: Supplementary file 6 — Source data Fig. 1 [file 44318_2025_402_MOESM6_ESM.zip › SD Figure 1/1C/1C Western Replicate#3.jpg]

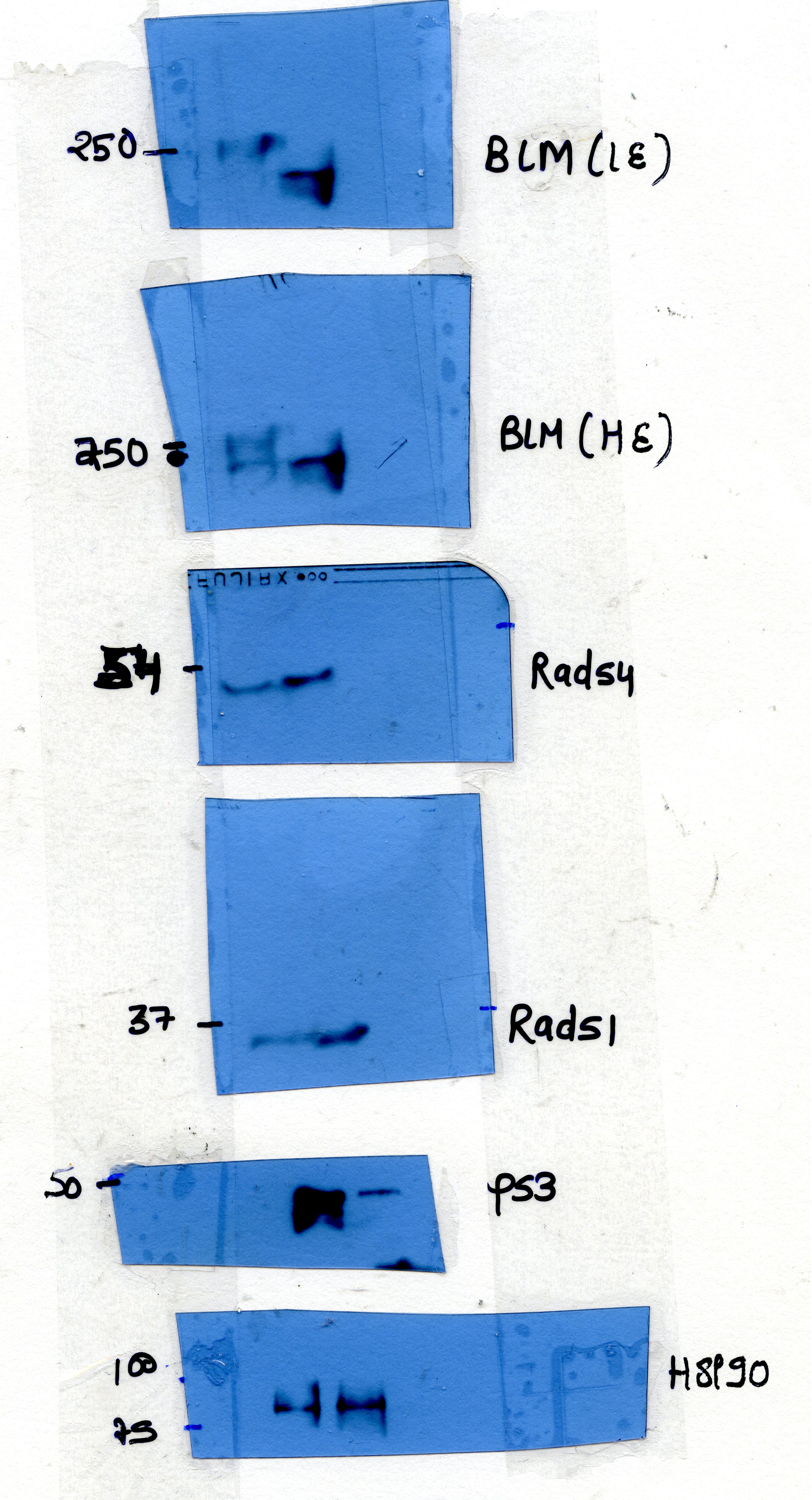

Supplement: Supplementary file 6 — Source data Fig. 1 [file 44318_2025_402_MOESM6_ESM.zip › SD Figure 1/1D/1D Western Replicate#1 .jpg]

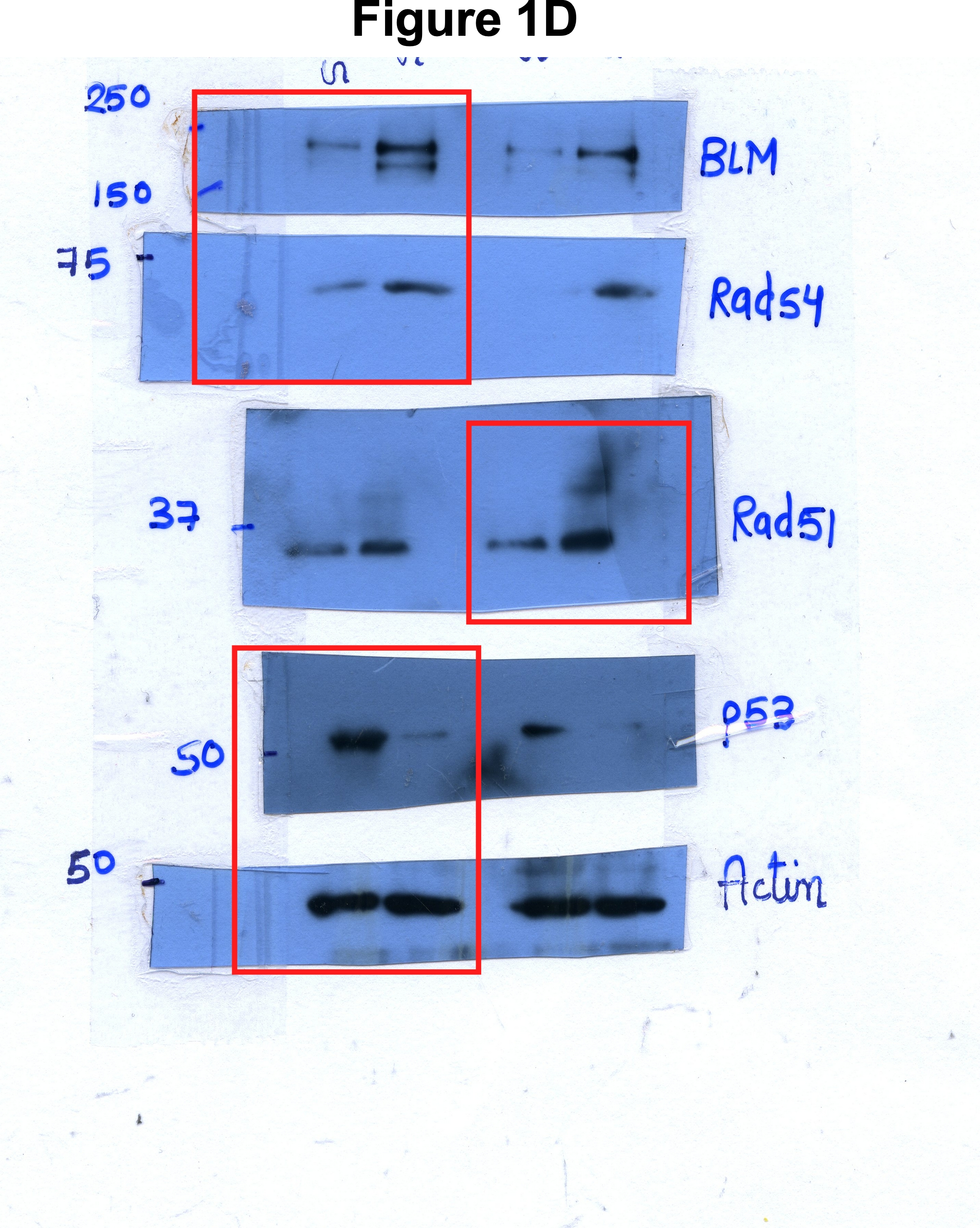

Supplement: Supplementary file 6 — Source data Fig. 1 [file 44318_2025_402_MOESM6_ESM.zip › SD Figure 1/1D/1D Western Replicate#2 (in publication).jpg]

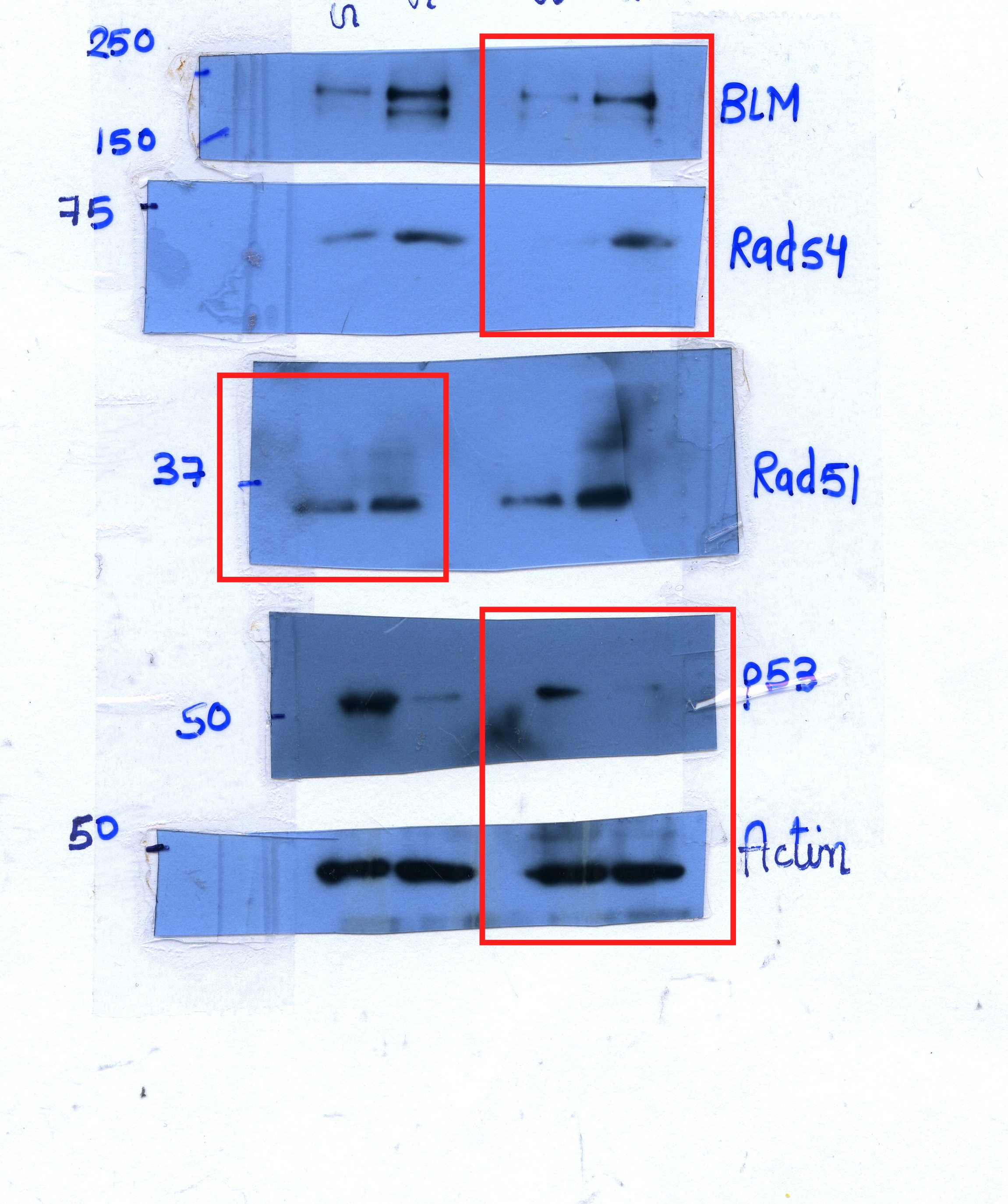

Supplement: Supplementary file 6 — Source data Fig. 1 [file 44318_2025_402_MOESM6_ESM.zip › SD Figure 1/1D/1D Western Replicate#3.jpg]

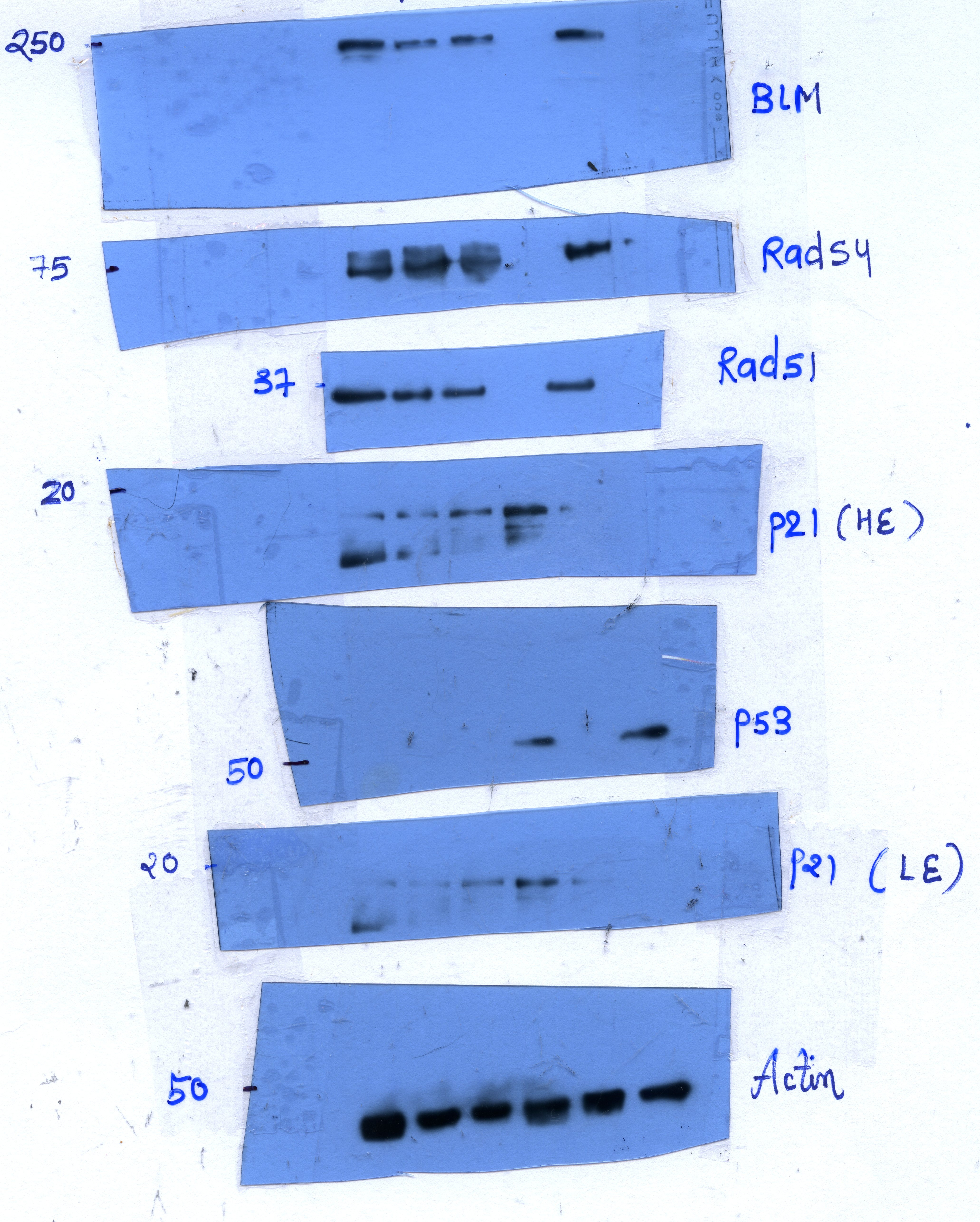

Supplement: Supplementary file 6 — Source data Fig. 1 [file 44318_2025_402_MOESM6_ESM.zip › SD Figure 1/1E/1E Western Replicate#1.jpg]

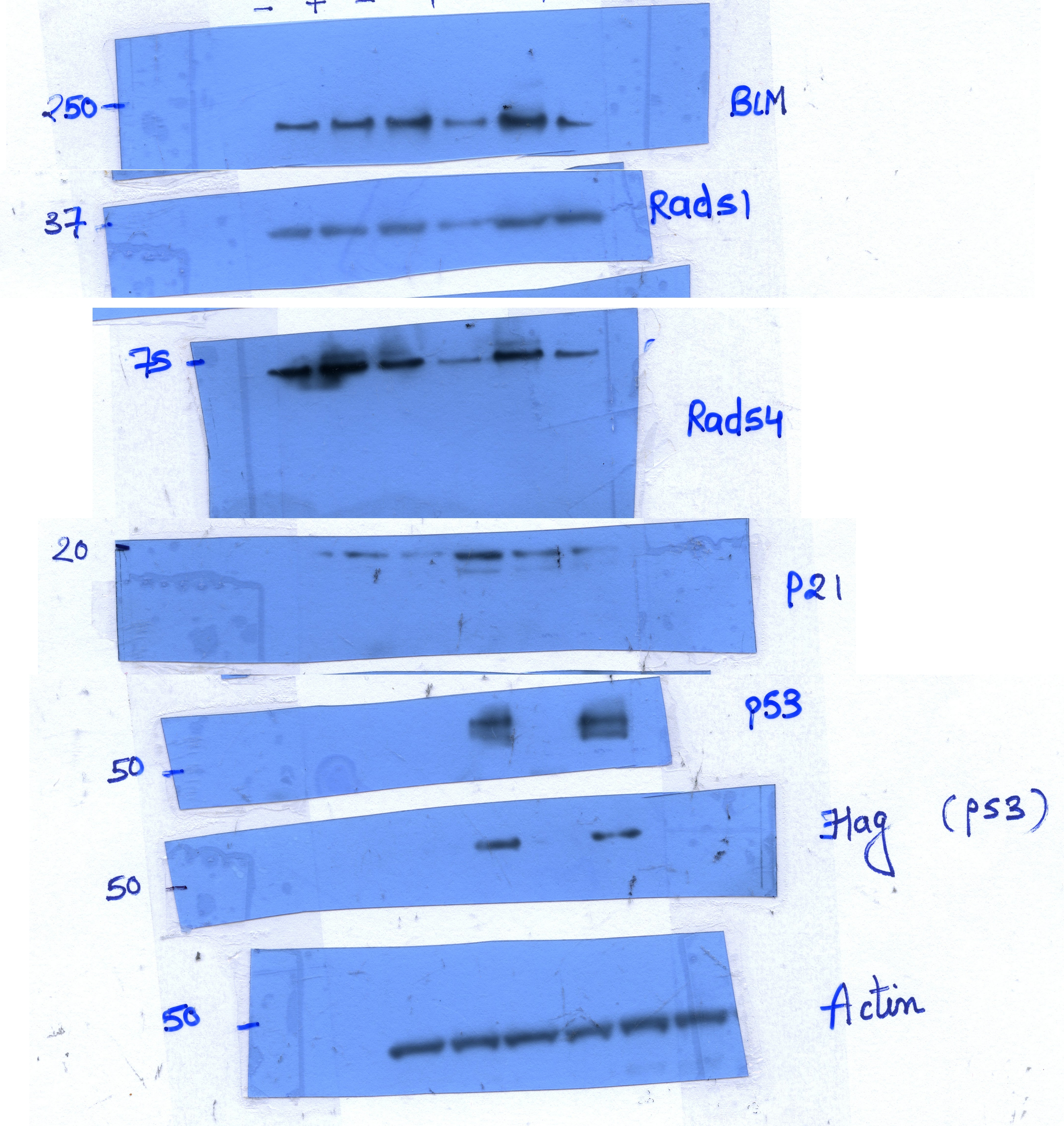

Supplement: Supplementary file 6 — Source data Fig. 1 [file 44318_2025_402_MOESM6_ESM.zip › SD Figure 1/1E/1E Western Replicate#2.jpg]

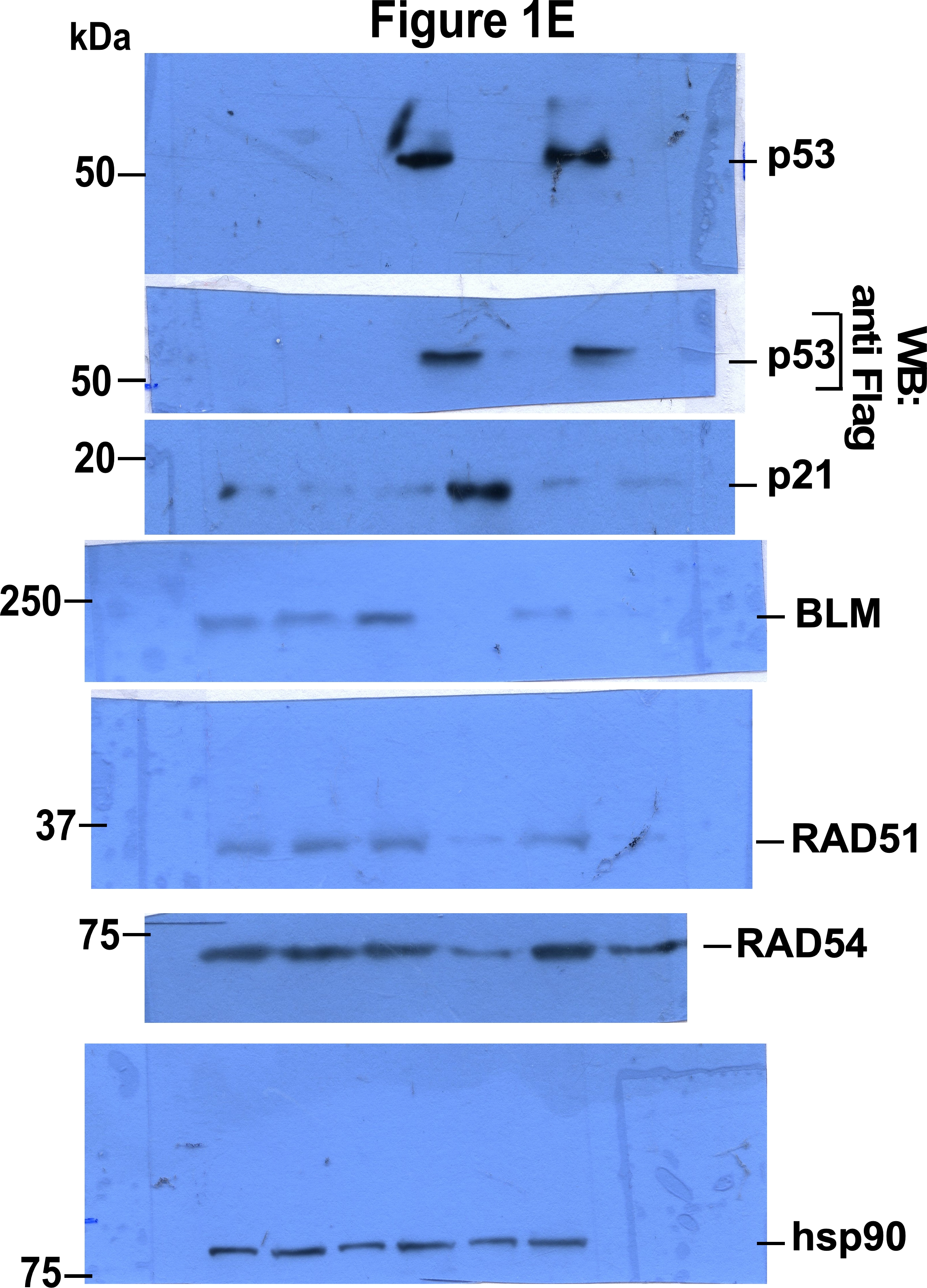

Supplement: Supplementary file 6 — Source data Fig. 1 [file 44318_2025_402_MOESM6_ESM.zip › SD Figure 1/1E/1E Western Replicate#3 (in publication).jpg]

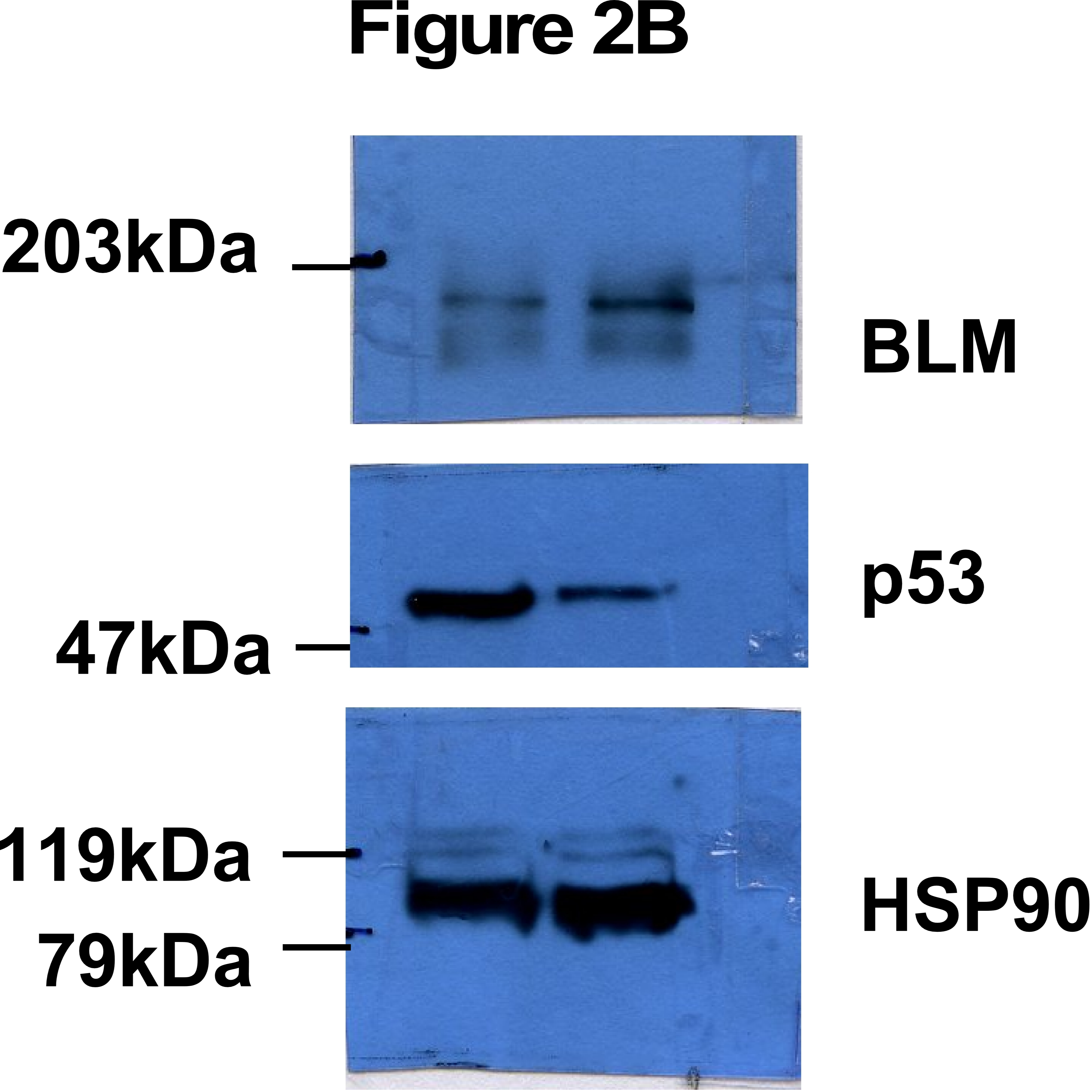

Supplement: Supplementary file 7 — Source data Fig. 2 [file 44318_2025_402_MOESM7_ESM.zip › SD Figure 2/2B/2B Western Replicate#1 (in publication).jpg]

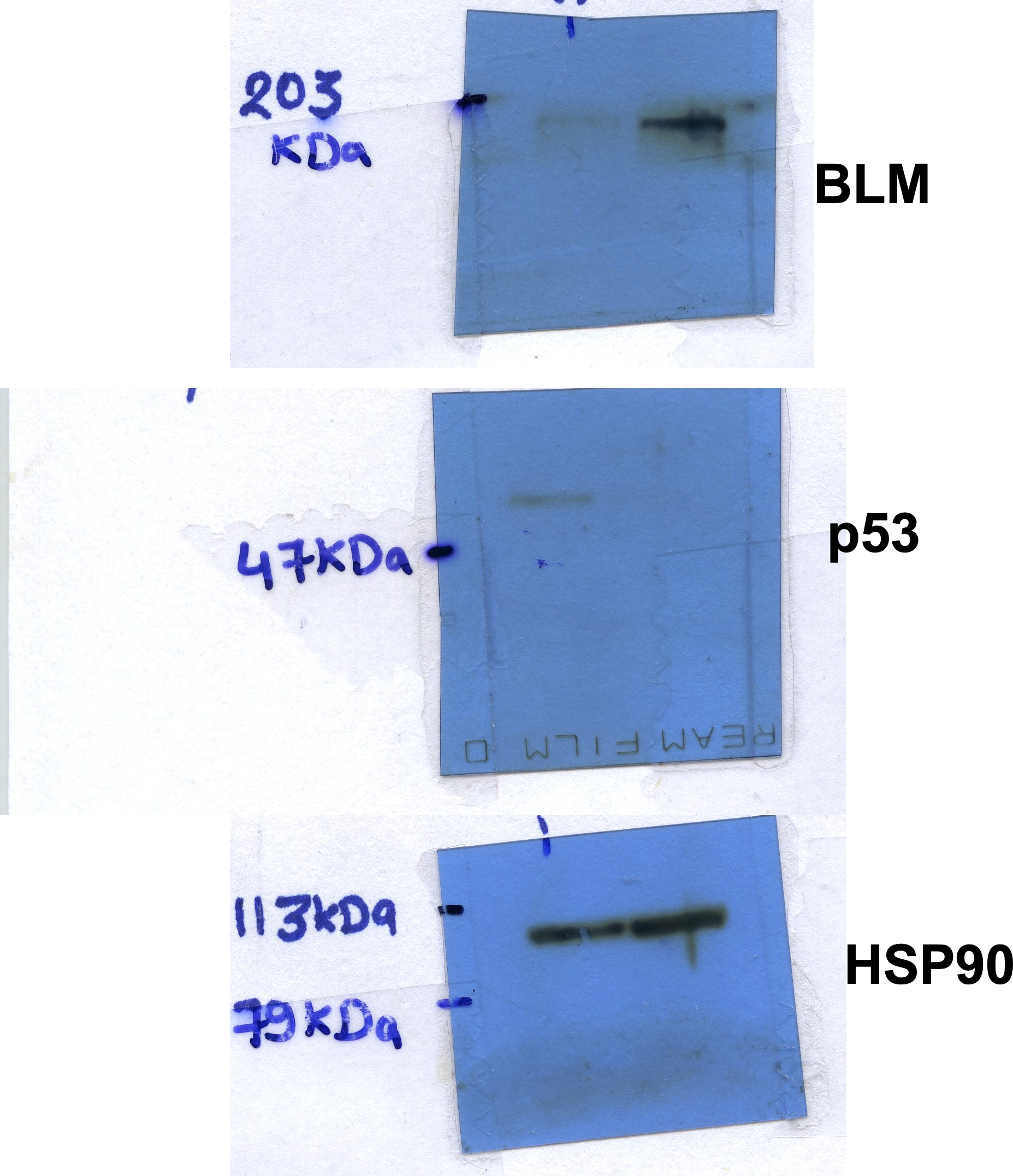

Supplement: Supplementary file 7 — Source data Fig. 2 [file 44318_2025_402_MOESM7_ESM.zip › SD Figure 2/2B/2B Western Replicate#2.jpg]

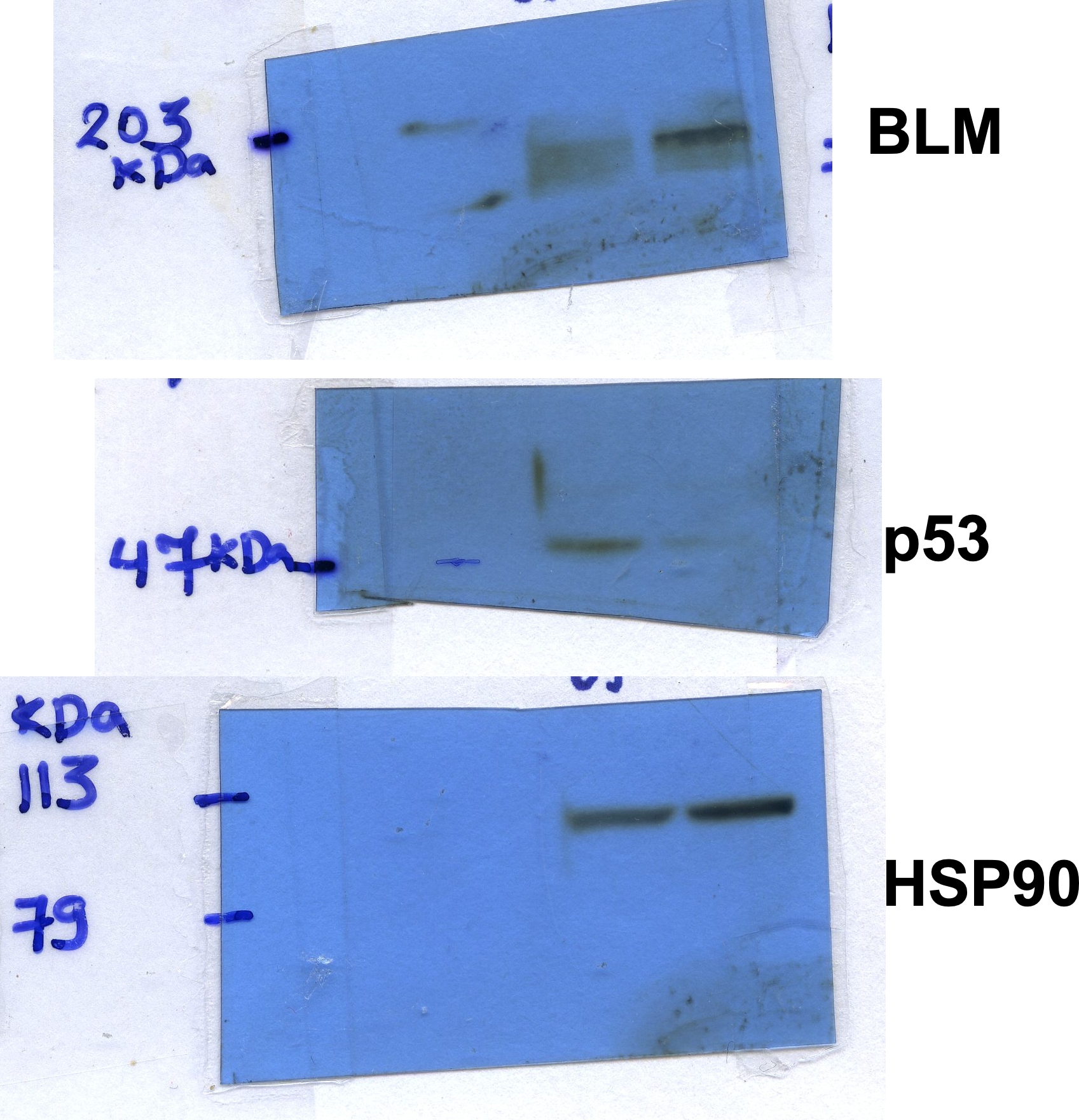

Supplement: Supplementary file 7 — Source data Fig. 2 [file 44318_2025_402_MOESM7_ESM.zip › SD Figure 2/2B/2B Western Replicate#3.jpg]

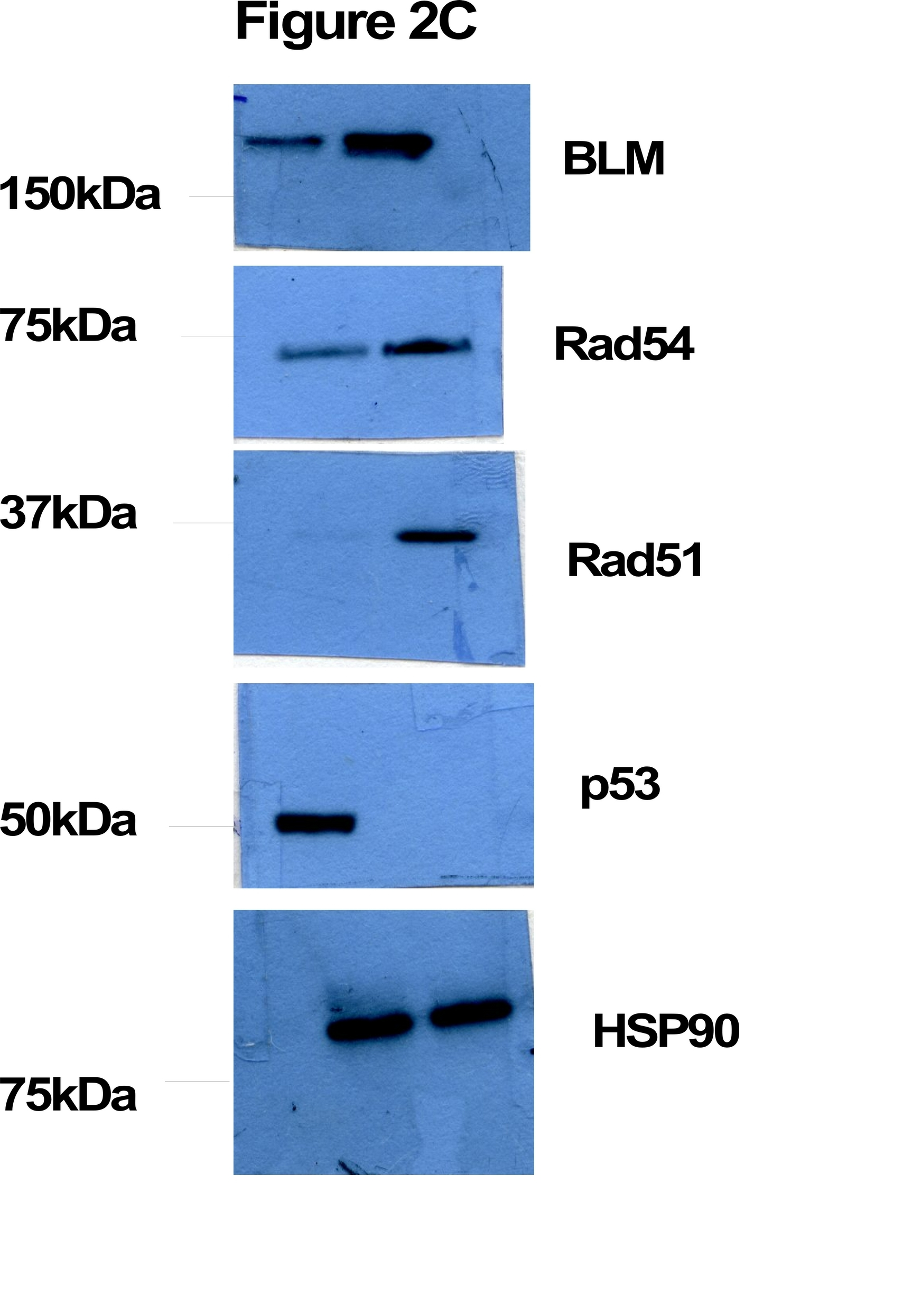

Supplement: Supplementary file 7 — Source data Fig. 2 [file 44318_2025_402_MOESM7_ESM.zip › SD Figure 2/2C/2C Western Replicate#1 (in publication).jpg]

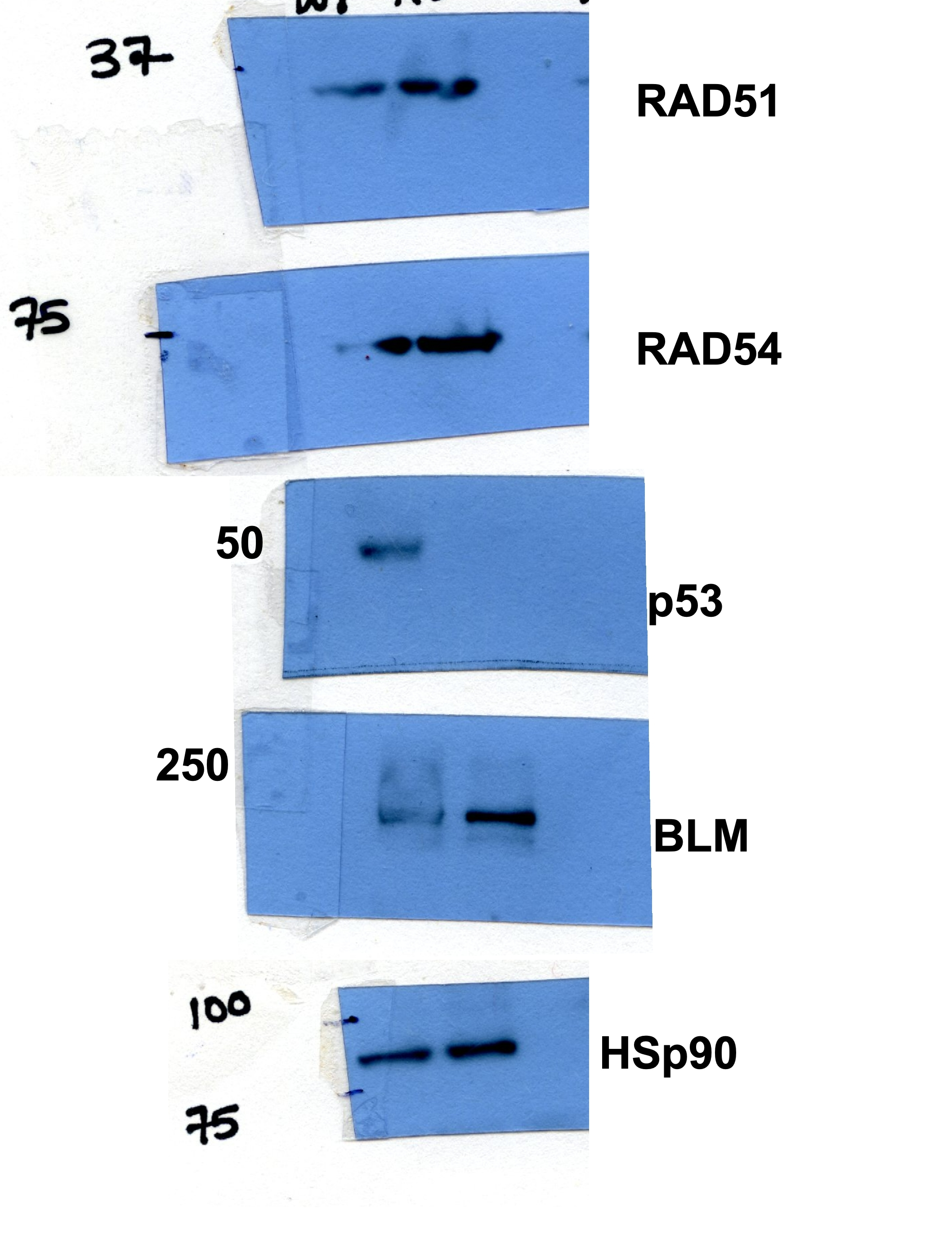

Supplement: Supplementary file 7 — Source data Fig. 2 [file 44318_2025_402_MOESM7_ESM.zip › SD Figure 2/2C/2C Western Replicate#2.jpg]

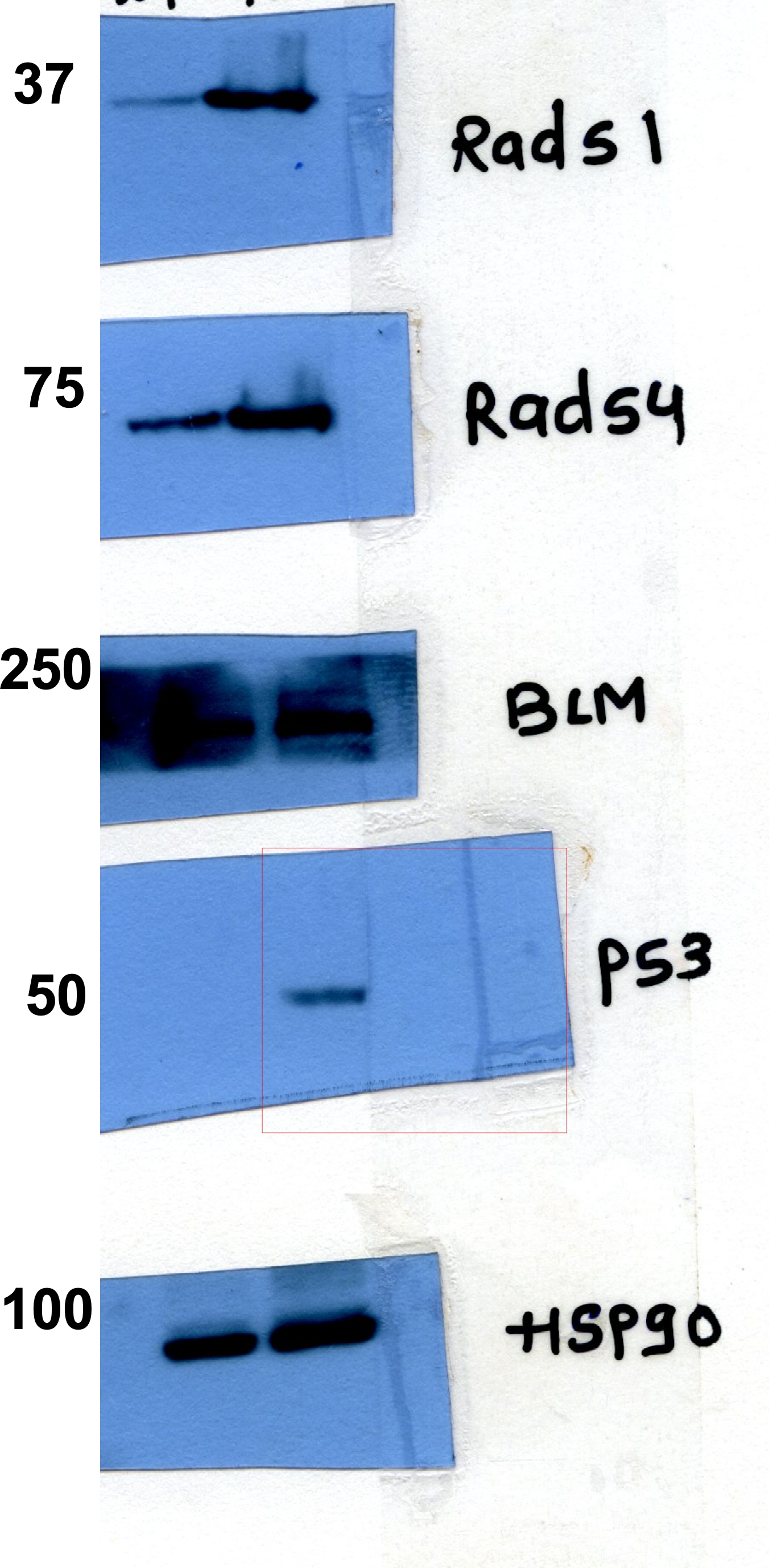

Supplement: Supplementary file 7 — Source data Fig. 2 [file 44318_2025_402_MOESM7_ESM.zip › SD Figure 2/2C/2C Western Replicate#3.jpg]

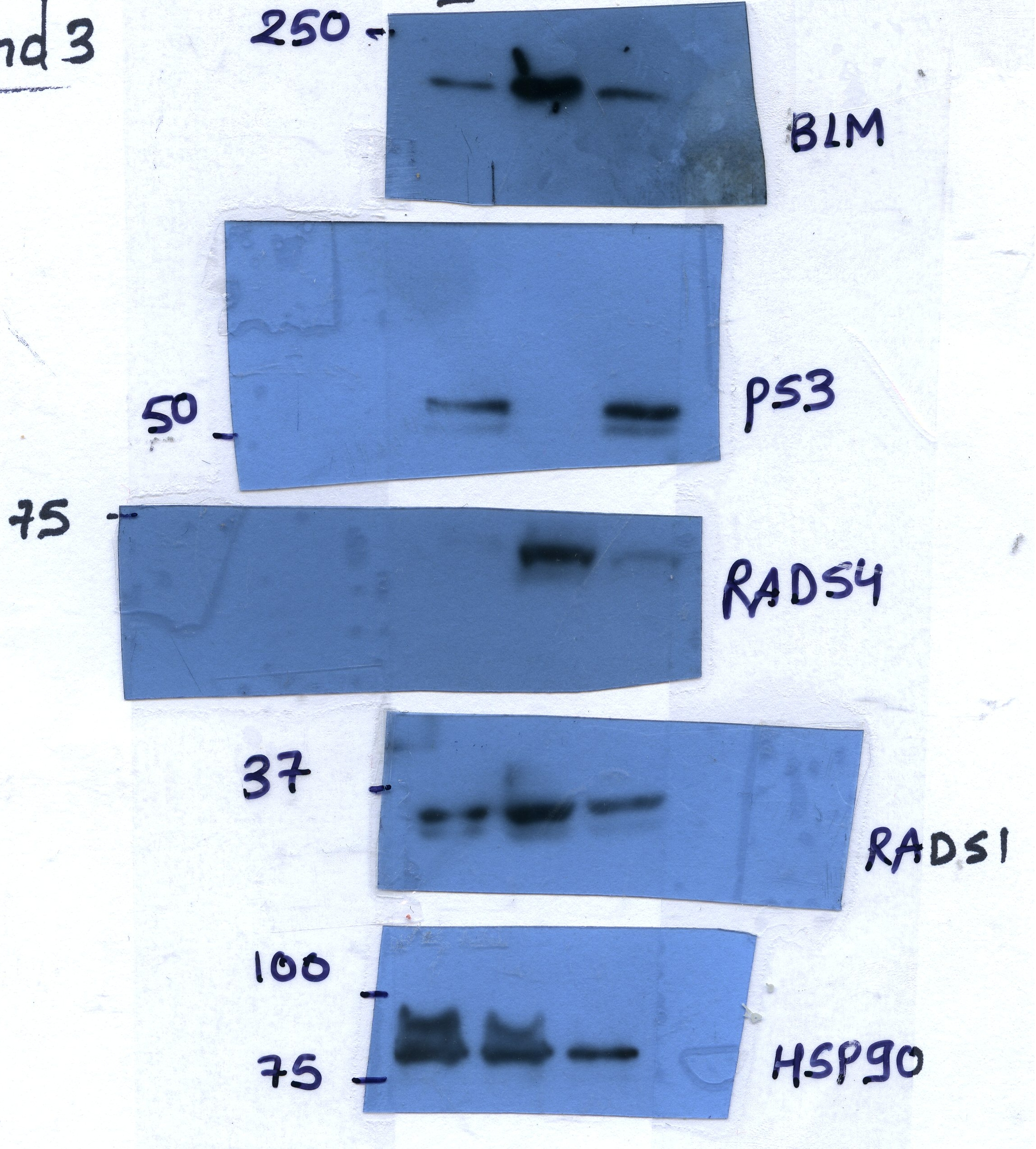

Supplement: Supplementary file 7 — Source data Fig. 2 [file 44318_2025_402_MOESM7_ESM.zip › SD Figure 2/2D/2D Western Replicate#1 .jpg]

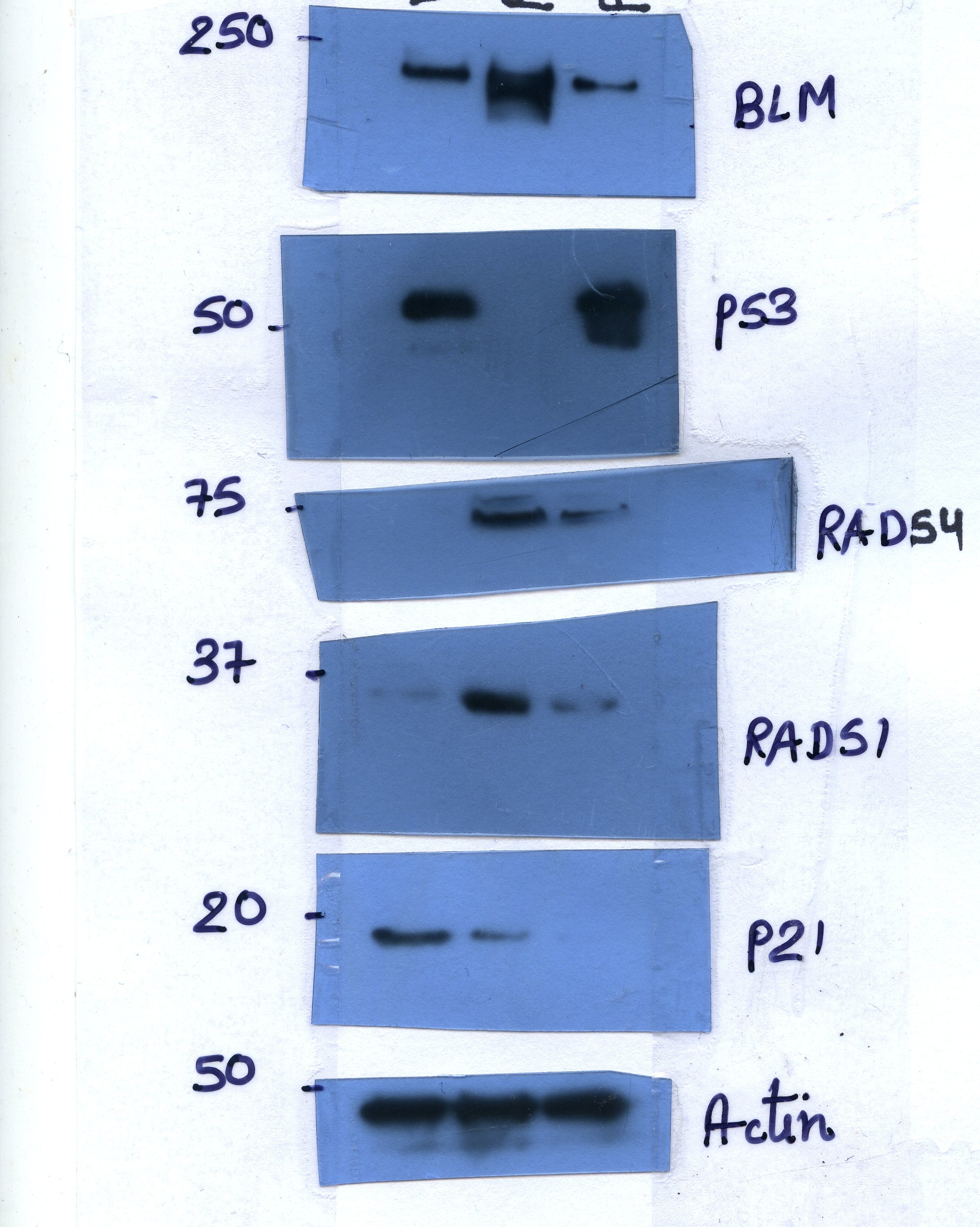

Supplement: Supplementary file 7 — Source data Fig. 2 [file 44318_2025_402_MOESM7_ESM.zip › SD Figure 2/2D/2D Western Replicate#2.jpg]

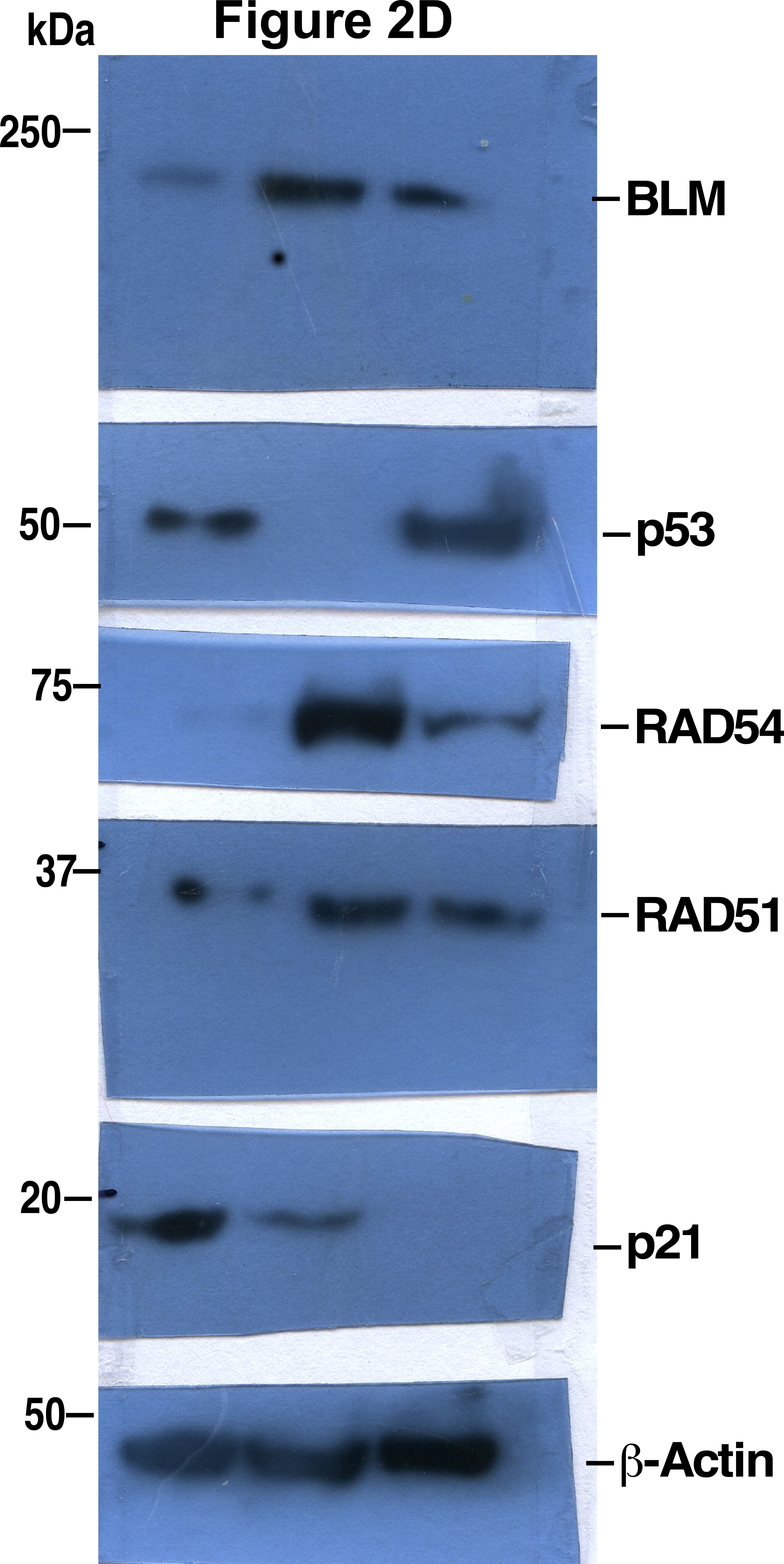

Supplement: Supplementary file 7 — Source data Fig. 2 [file 44318_2025_402_MOESM7_ESM.zip › SD Figure 2/2D/2D Western Replicate#3 (in publication).jpg]

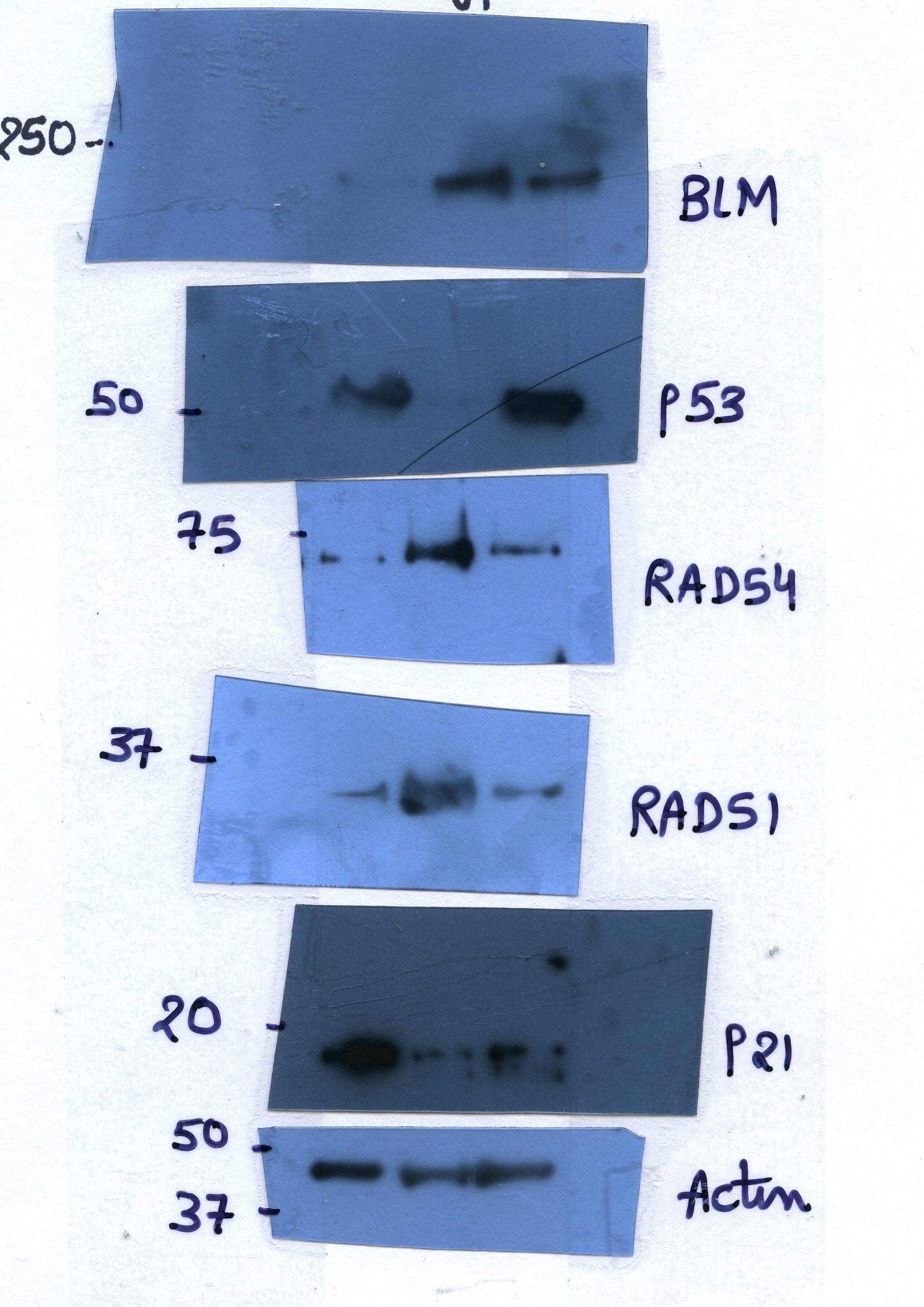

Supplement: Supplementary file 7 — Source data Fig. 2 [file 44318_2025_402_MOESM7_ESM.zip › SD Figure 2/2F/2F Western Replicate#1.jpg]

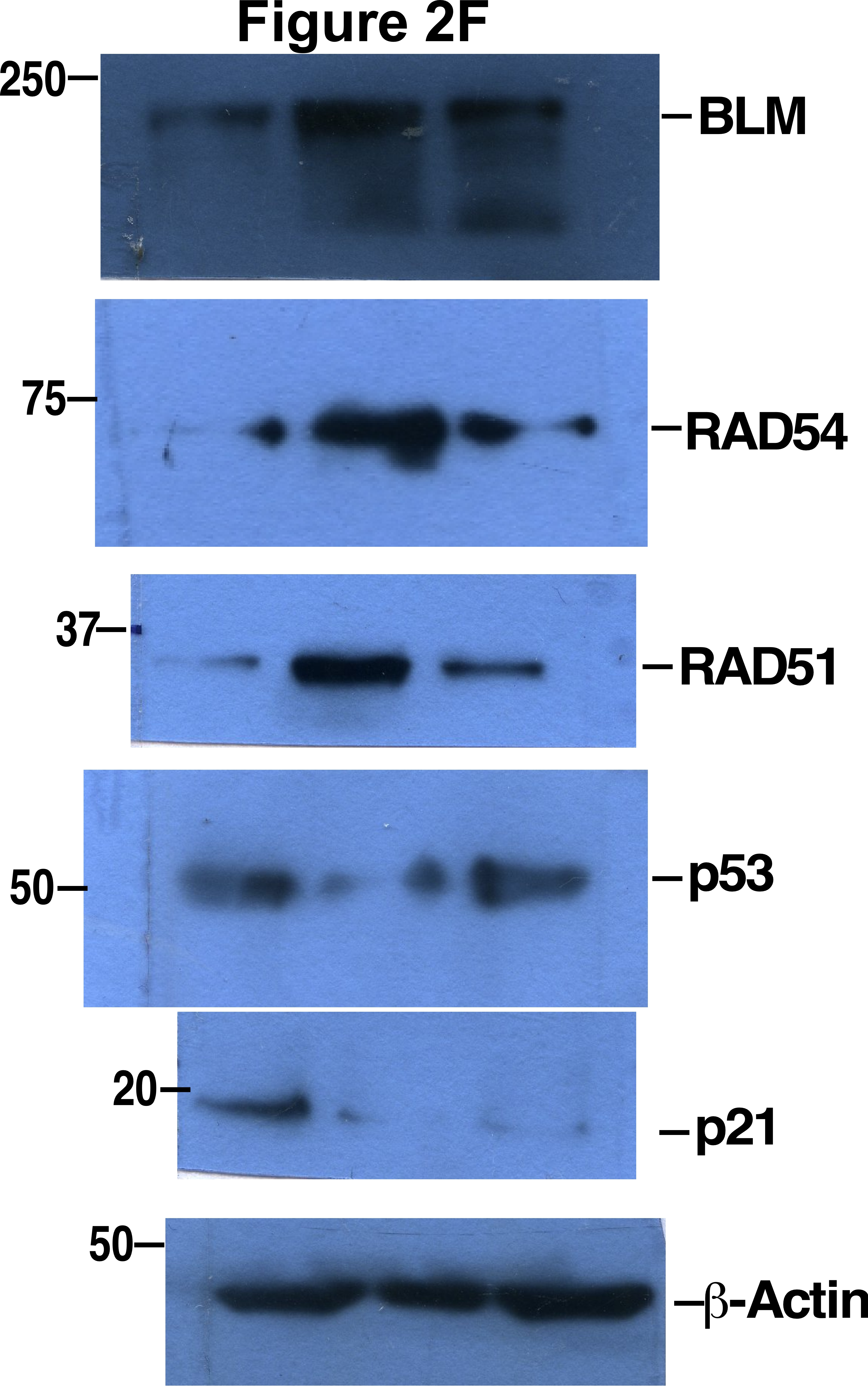

Supplement: Supplementary file 7 — Source data Fig. 2 [file 44318_2025_402_MOESM7_ESM.zip › SD Figure 2/2F/2F Western Replicate#2 (in publication).jpg]

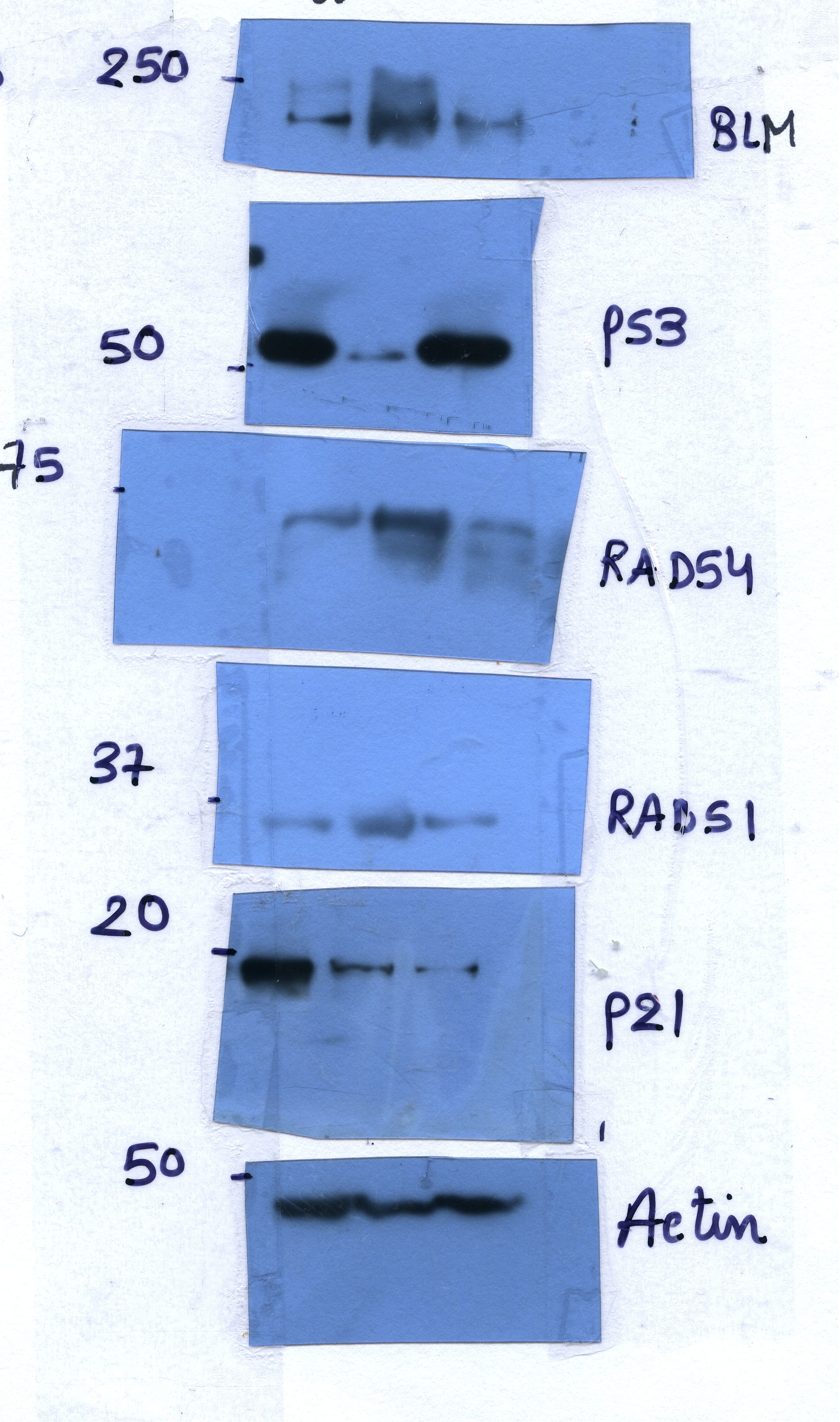

Supplement: Supplementary file 7 — Source data Fig. 2 [file 44318_2025_402_MOESM7_ESM.zip › SD Figure 2/2F/2F Western Replicate#3.jpg]

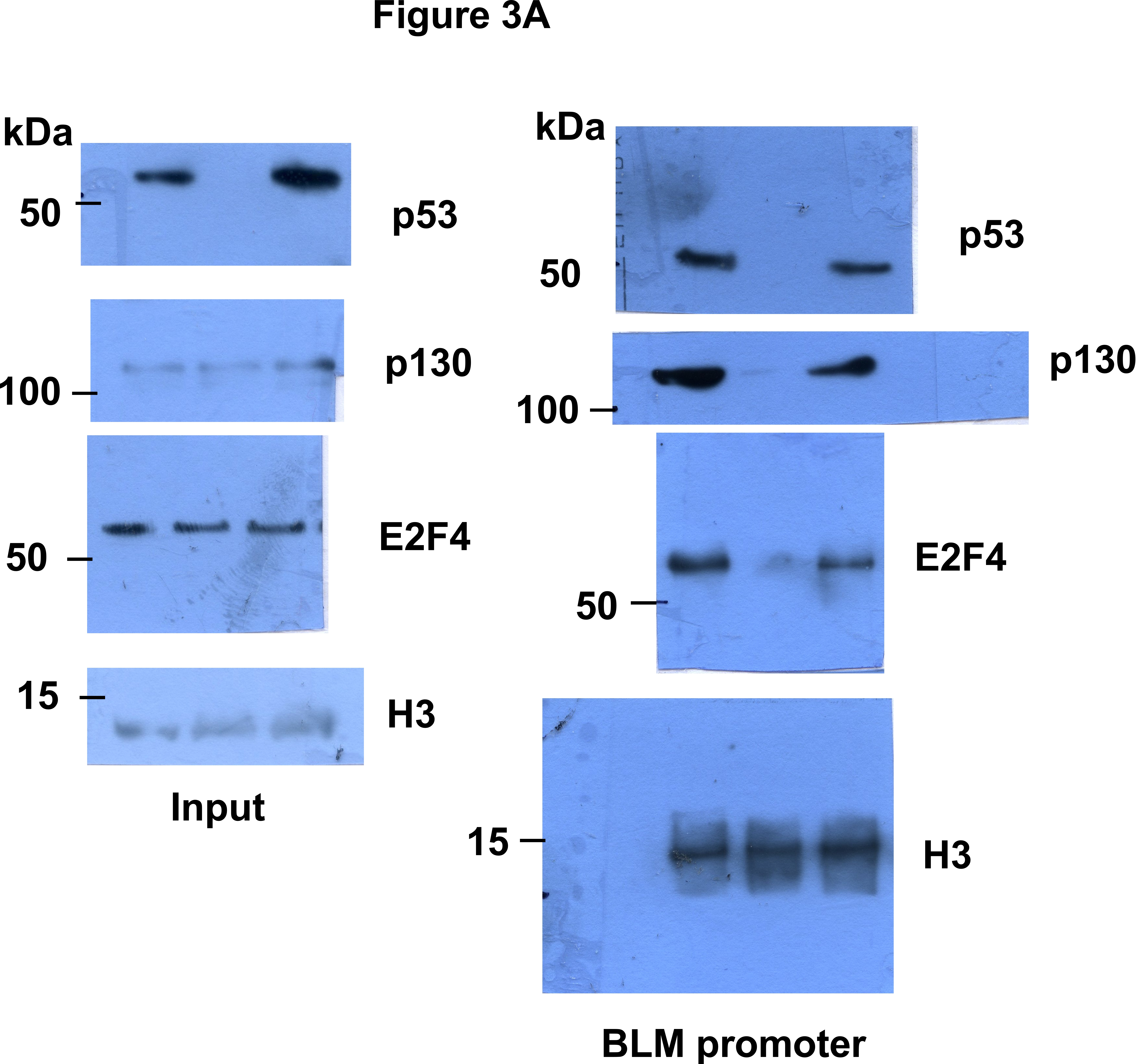

Supplement: Supplementary file 8 — Source data Fig. 3 [file 44318_2025_402_MOESM8_ESM.zip › SD Figure 3/3A/3A Western Replicate#1 (in publication).jpg]

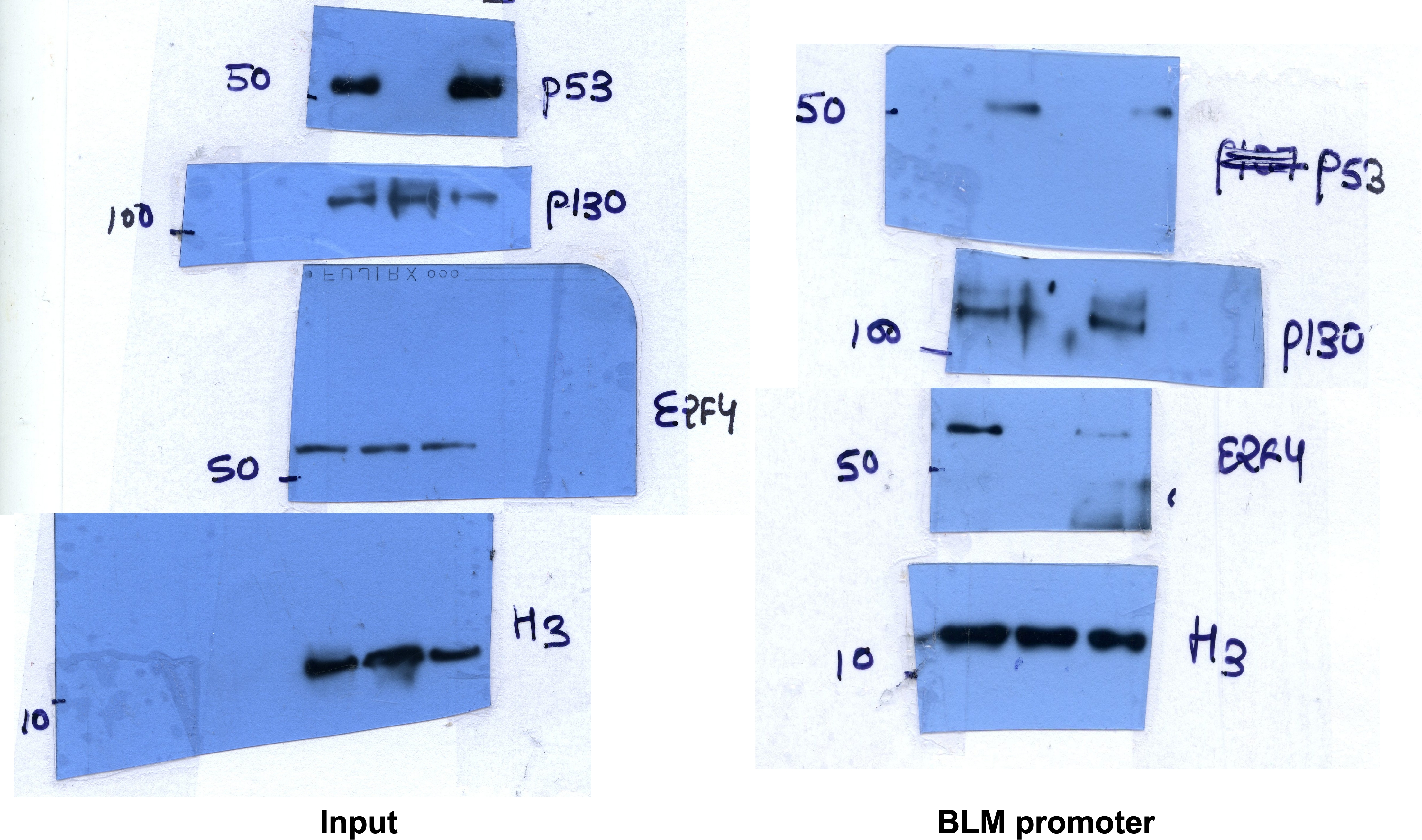

Supplement: Supplementary file 8 — Source data Fig. 3 [file 44318_2025_402_MOESM8_ESM.zip › SD Figure 3/3A/3A Western Replicate#2 .jpg]

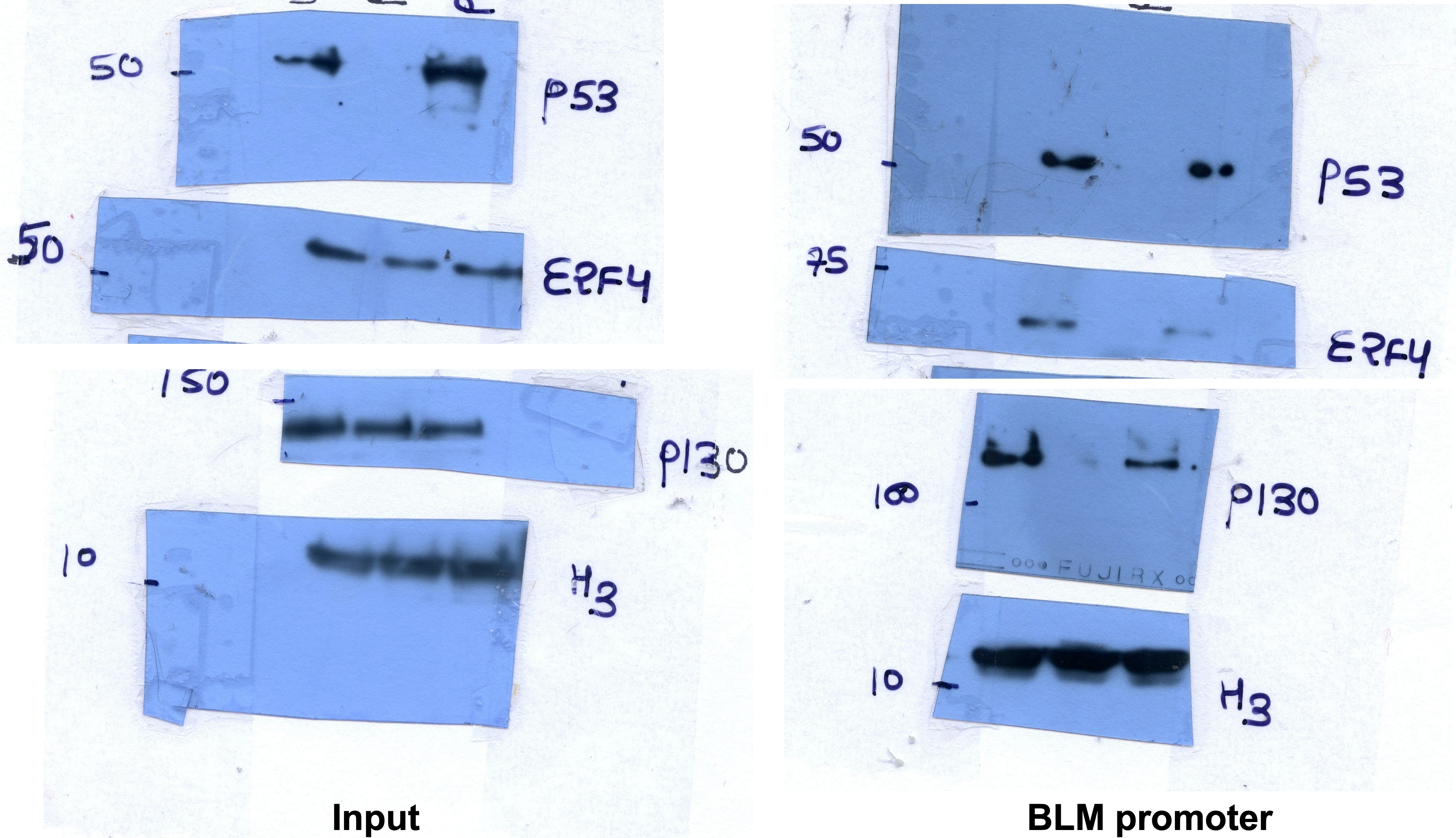

Supplement: Supplementary file 8 — Source data Fig. 3 [file 44318_2025_402_MOESM8_ESM.zip › SD Figure 3/3A/3A Western Replicate#3.jpg]

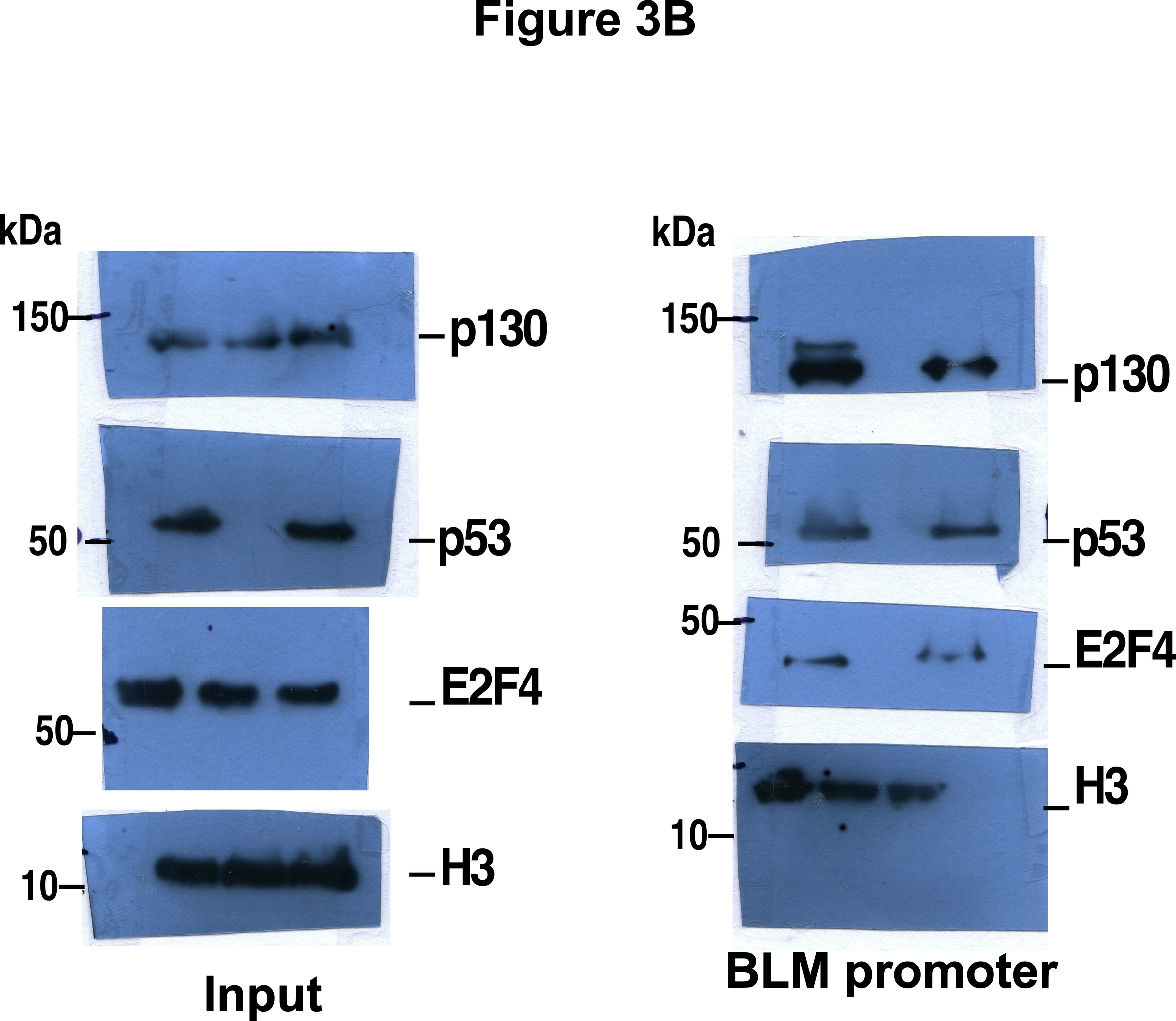

Supplement: Supplementary file 8 — Source data Fig. 3 [file 44318_2025_402_MOESM8_ESM.zip › SD Figure 3/3B/3B Western Replicate#1 (in publication).jpg]

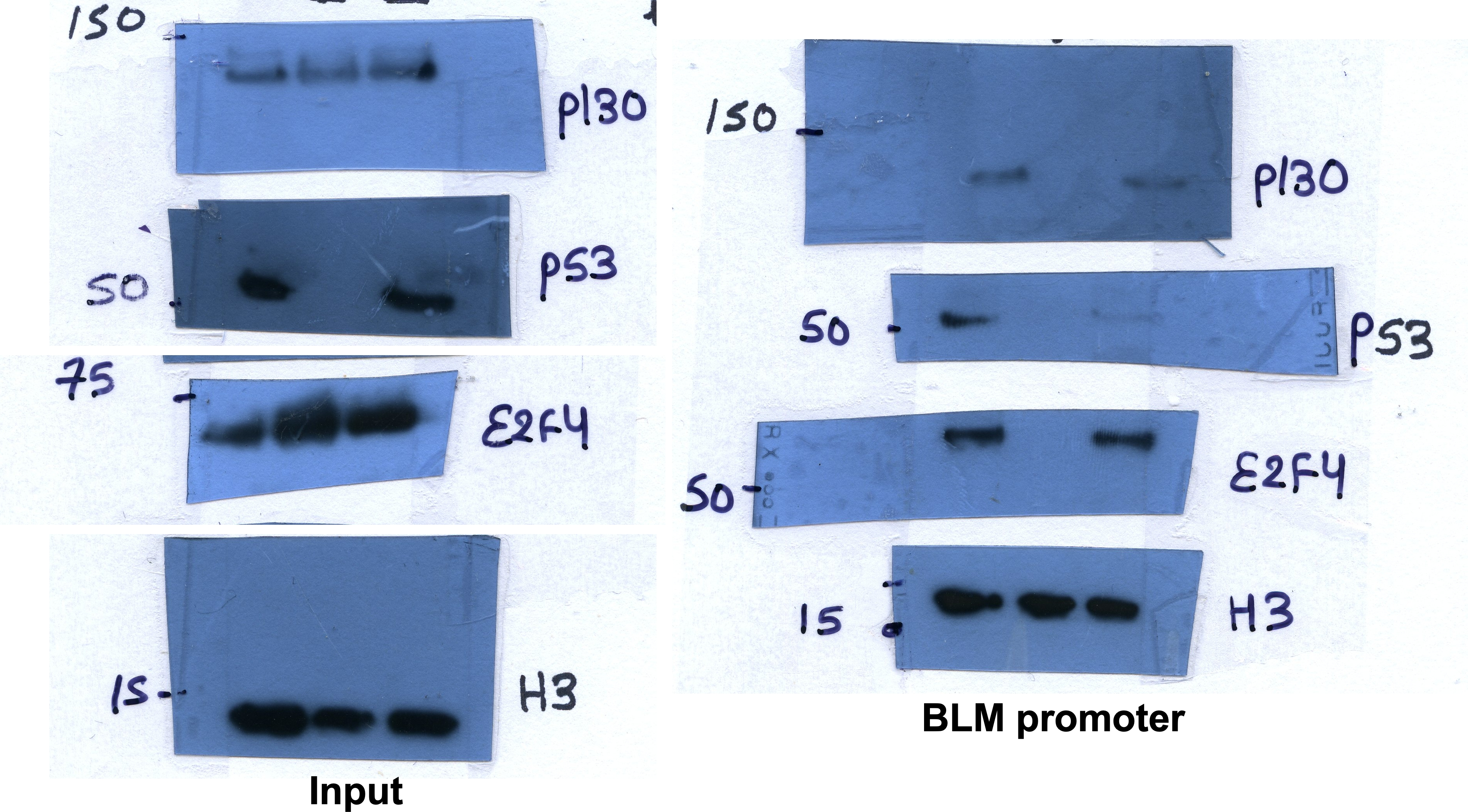

Supplement: Supplementary file 8 — Source data Fig. 3 [file 44318_2025_402_MOESM8_ESM.zip › SD Figure 3/3B/3B Western Replicate#2.jpg]

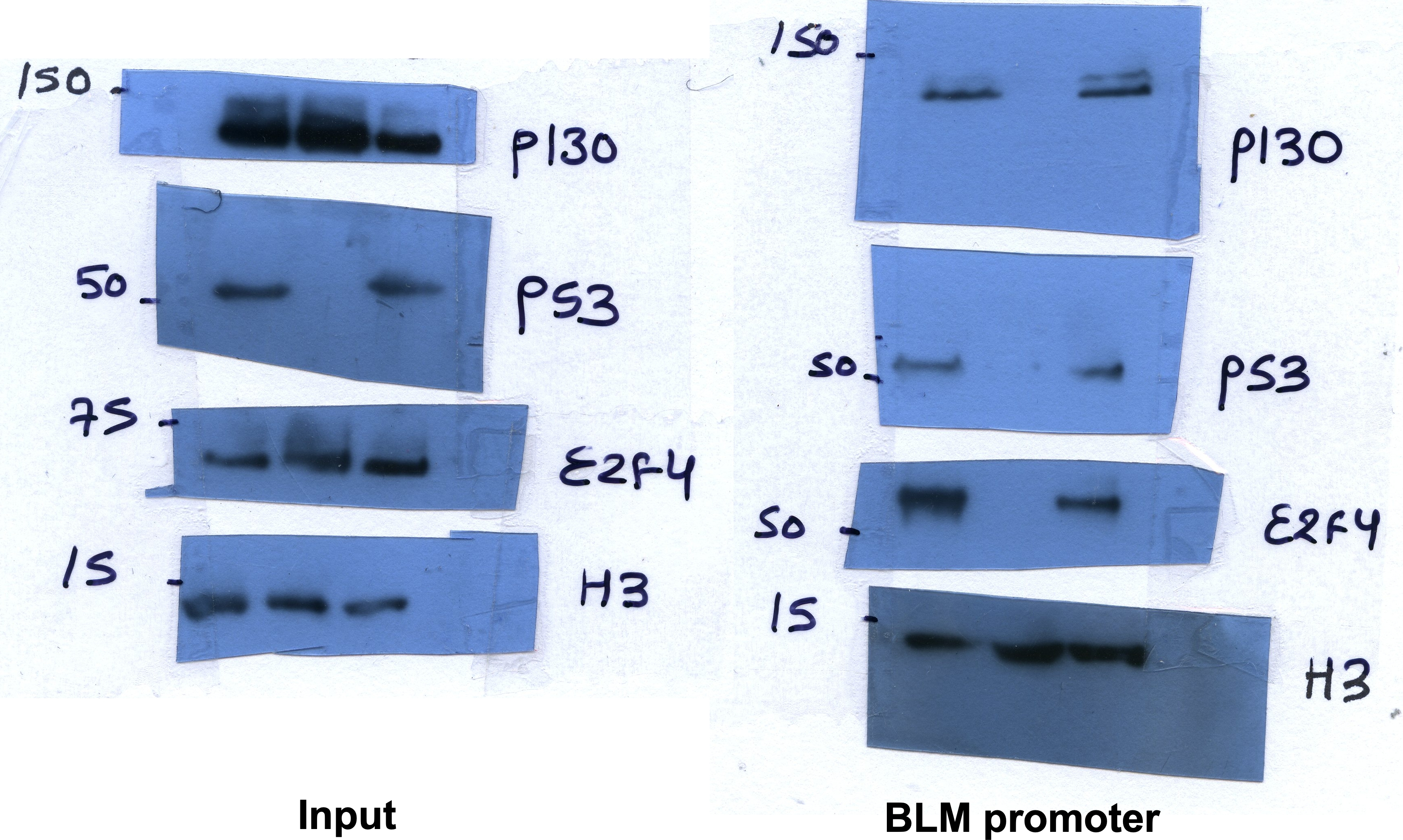

Supplement: Supplementary file 8 — Source data Fig. 3 [file 44318_2025_402_MOESM8_ESM.zip › SD Figure 3/3B/3B Western Replicate#3.jpg]

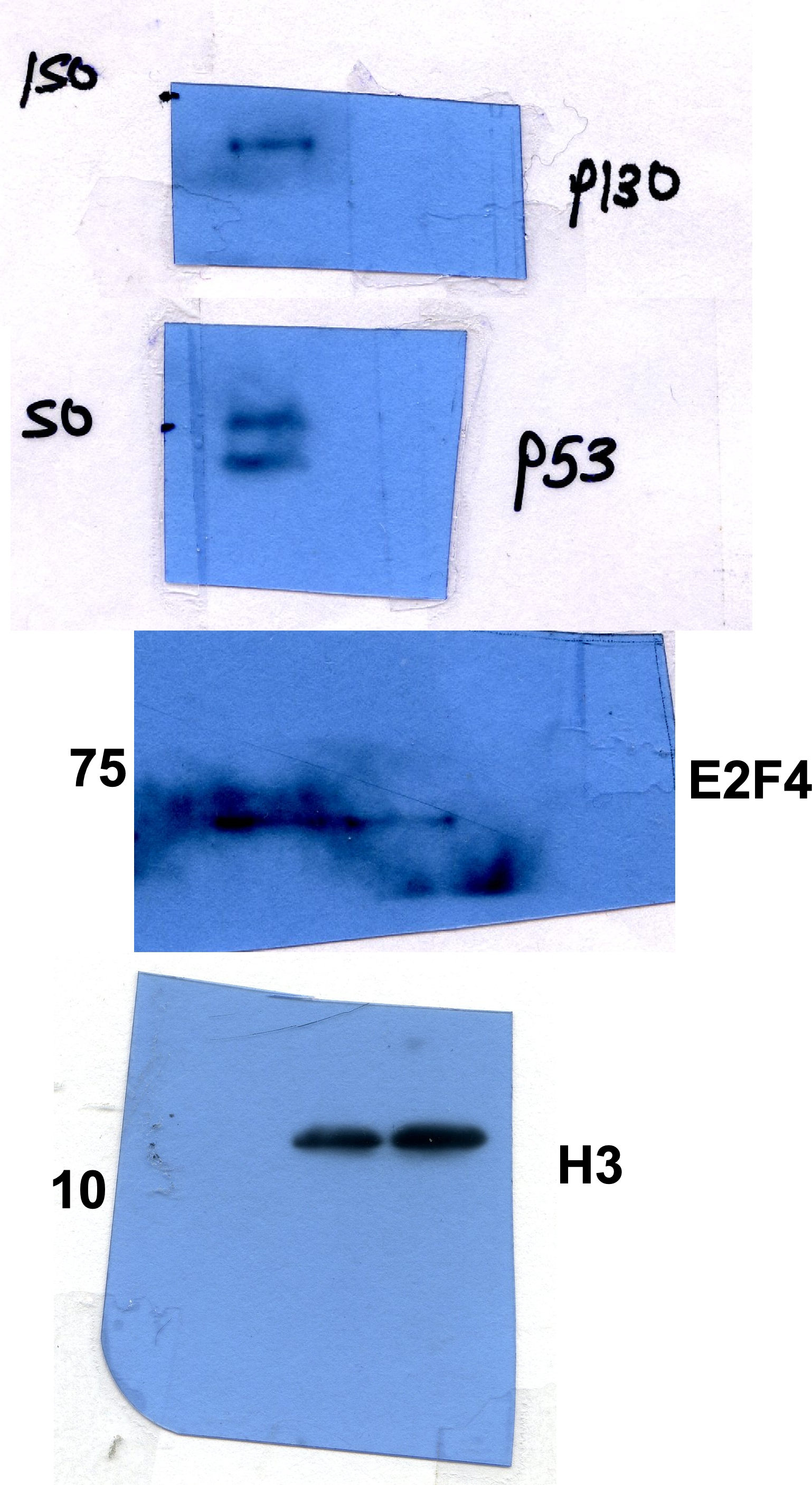

Supplement: Supplementary file 8 — Source data Fig. 3 [file 44318_2025_402_MOESM8_ESM.zip › SD Figure 3/3C/3C Western Replicate#1.jpg]

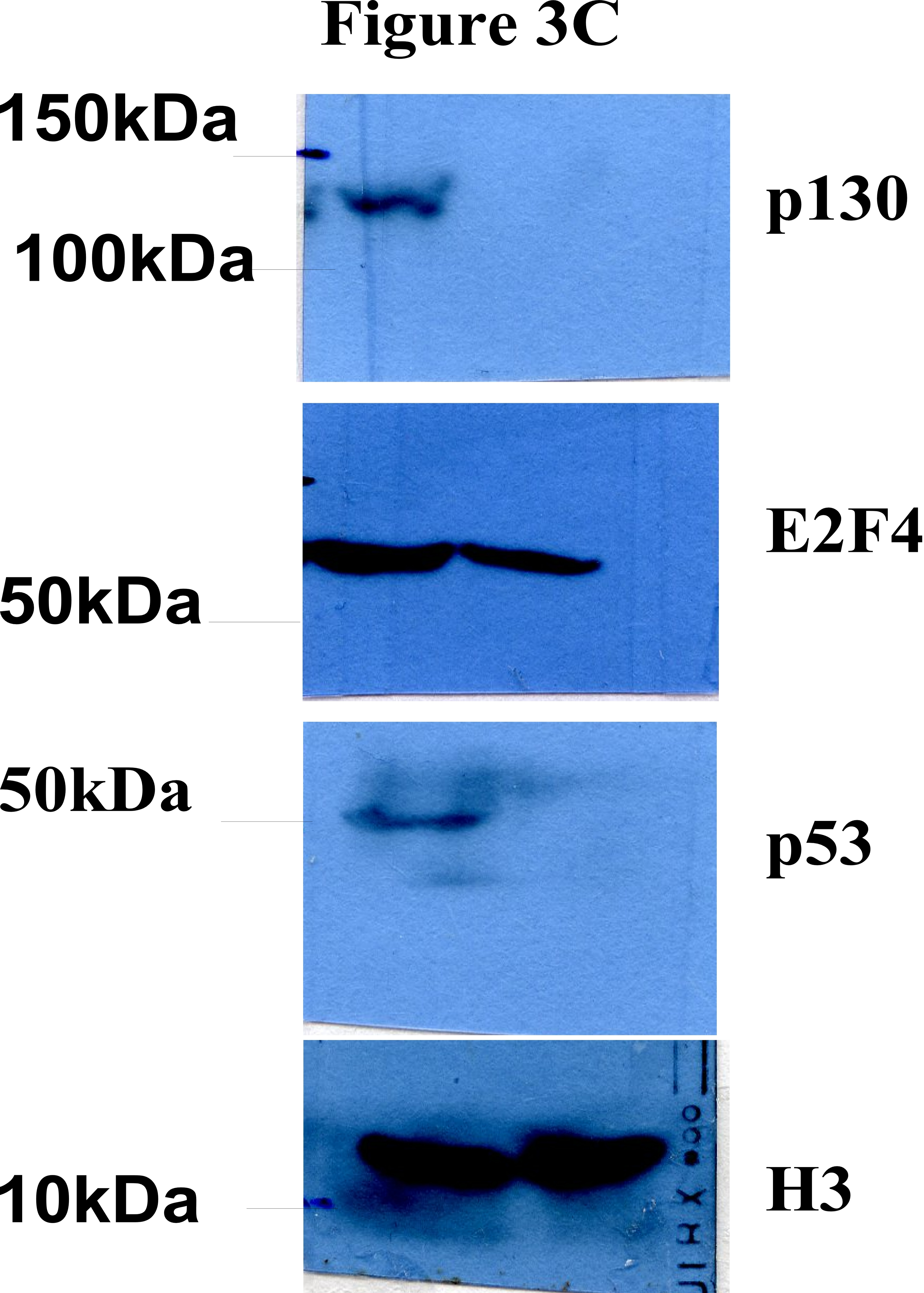

Supplement: Supplementary file 8 — Source data Fig. 3 [file 44318_2025_402_MOESM8_ESM.zip › SD Figure 3/3C/3C Western Replicate#2 (in publication).jpg]

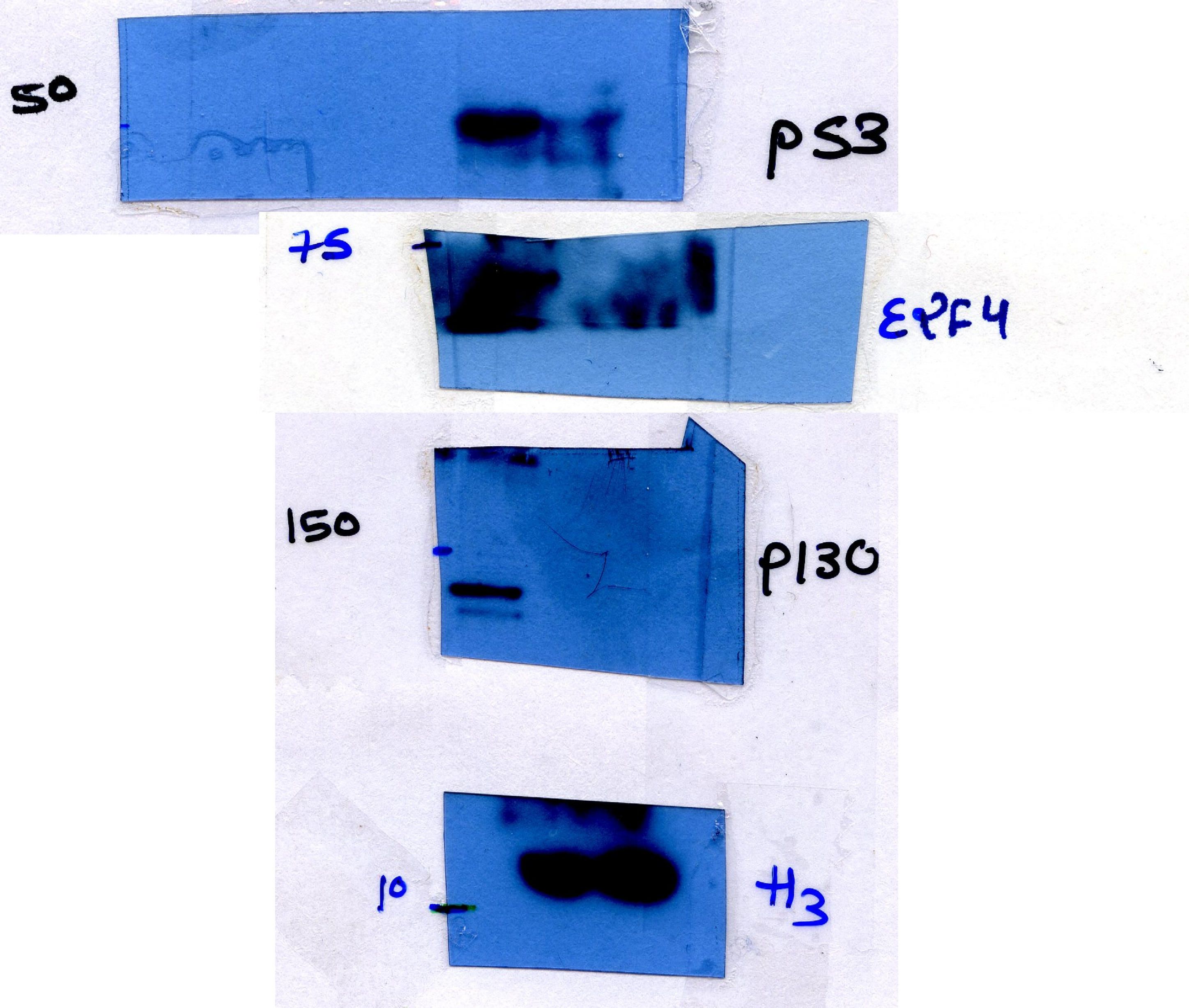

Supplement: Supplementary file 8 — Source data Fig. 3 [file 44318_2025_402_MOESM8_ESM.zip › SD Figure 3/3C/3C Western Replicate#3.jpg]

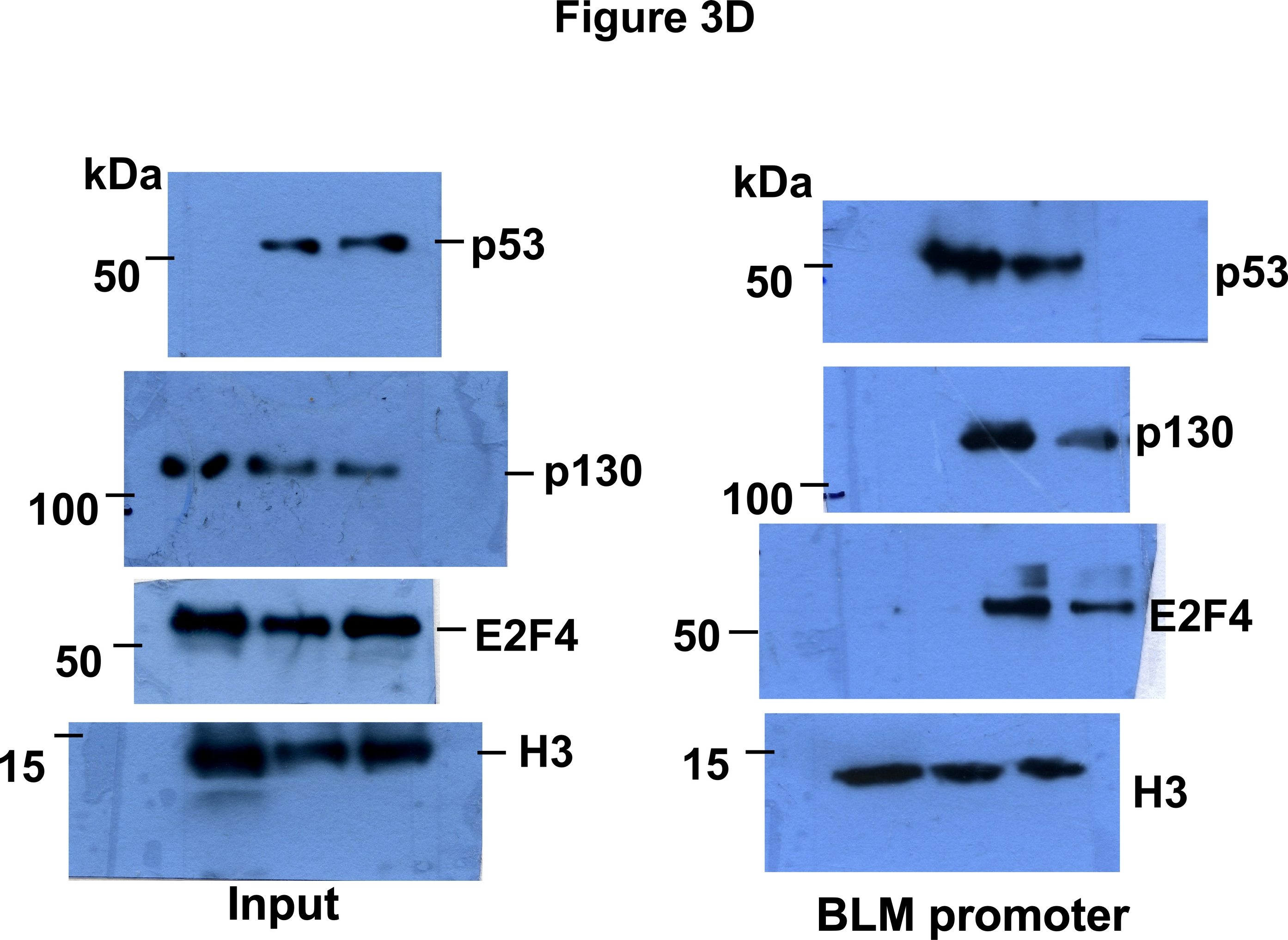

Supplement: Supplementary file 8 — Source data Fig. 3 [file 44318_2025_402_MOESM8_ESM.zip › SD Figure 3/3D/3D Western Replicate#1 (in publication).jpg]

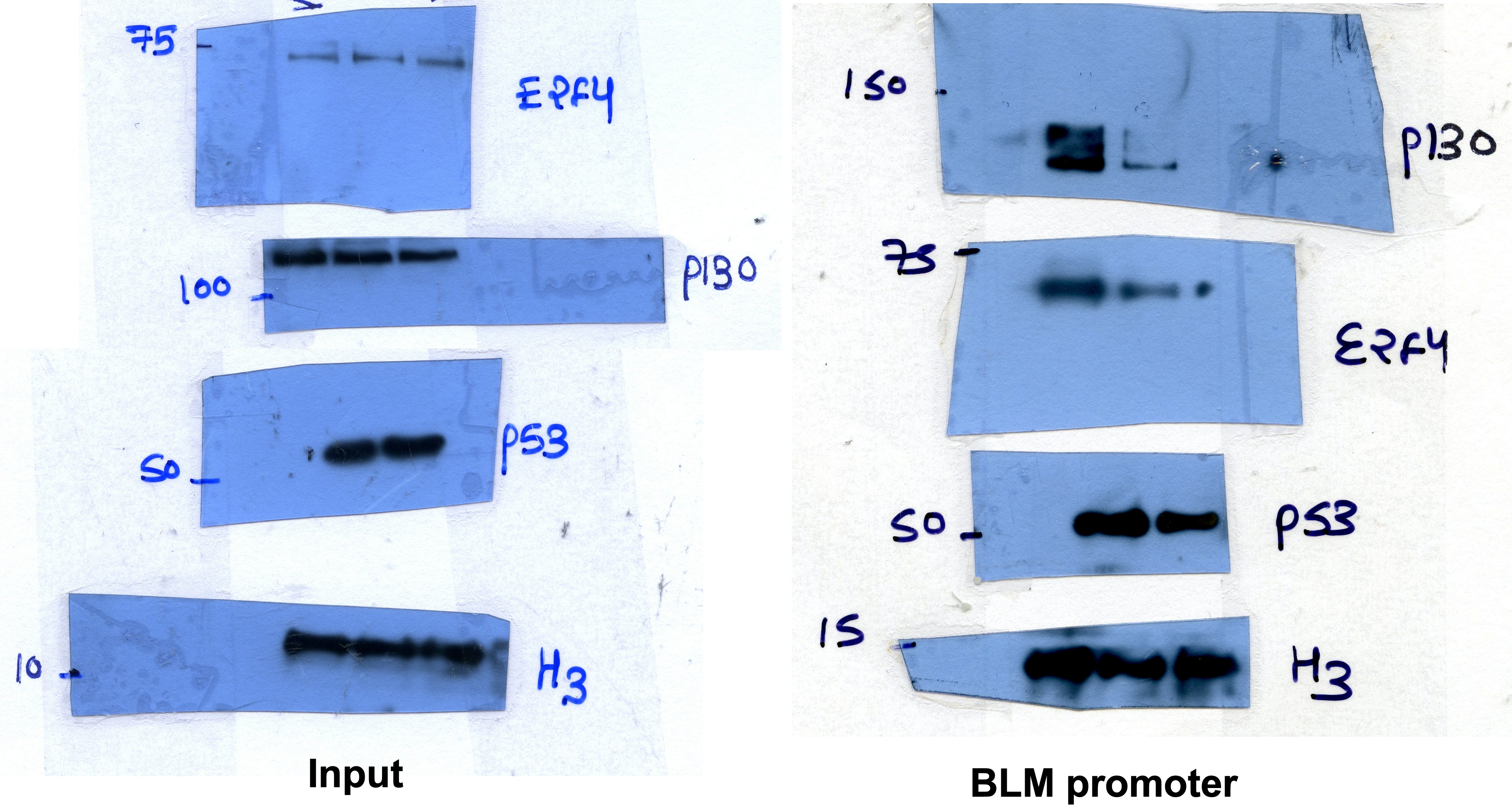

Supplement: Supplementary file 8 — Source data Fig. 3 [file 44318_2025_402_MOESM8_ESM.zip › SD Figure 3/3D/3D Western Replicate#2.jpg]

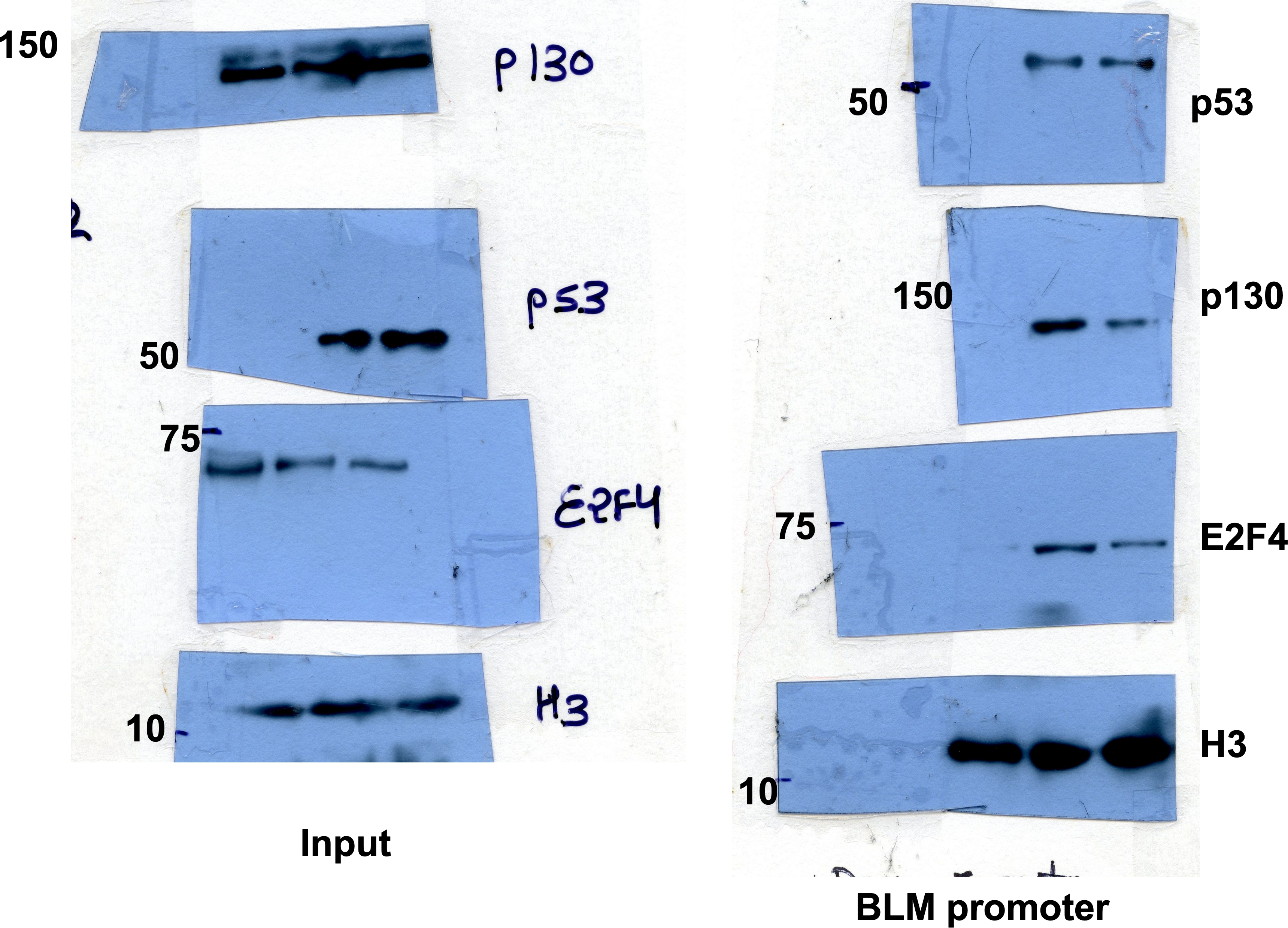

Supplement: Supplementary file 8 — Source data Fig. 3 [file 44318_2025_402_MOESM8_ESM.zip › SD Figure 3/3D/3D Western Replicate#3.jpg]

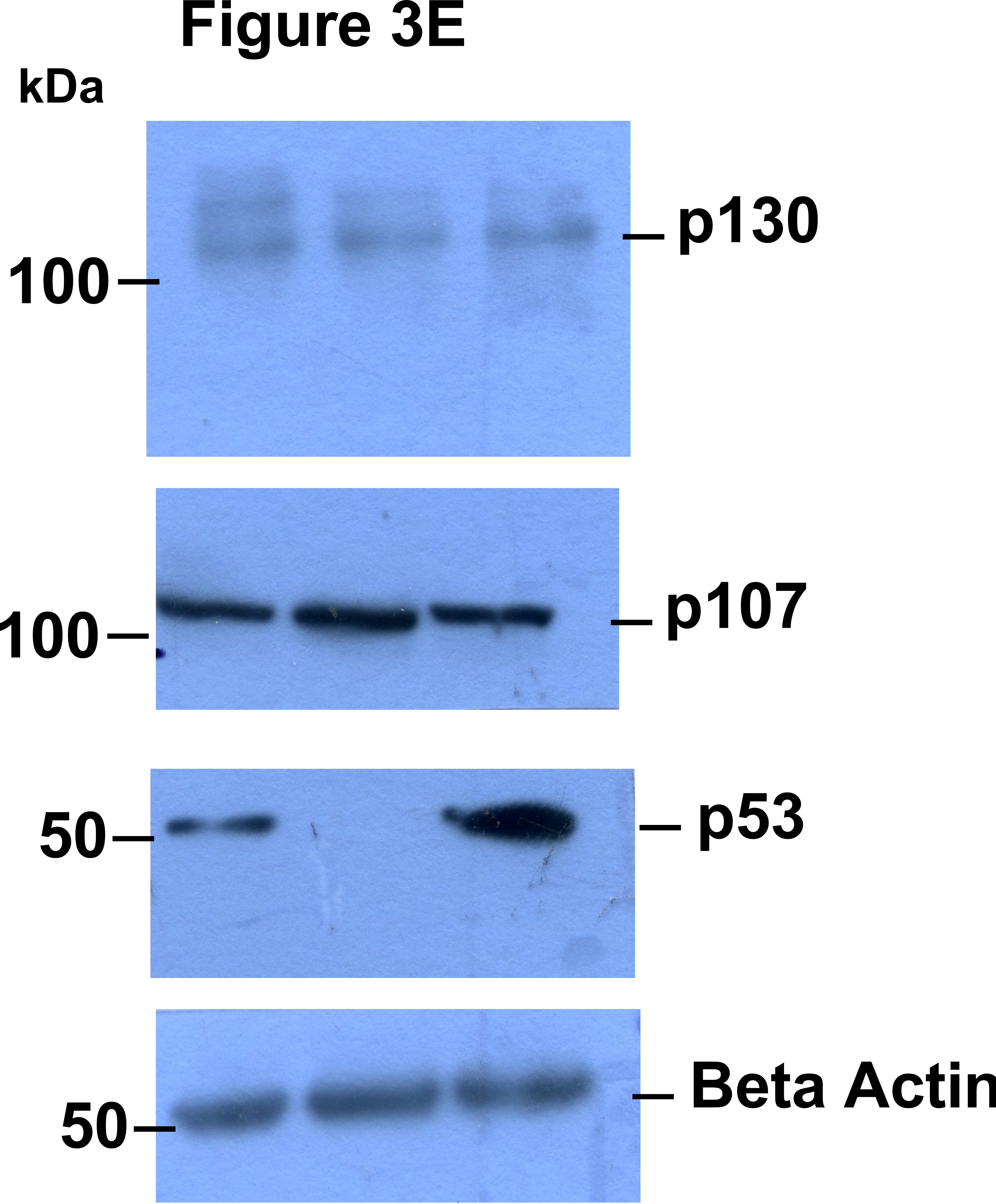

Supplement: Supplementary file 8 — Source data Fig. 3 [file 44318_2025_402_MOESM8_ESM.zip › SD Figure 3/3E/3E Western Replicate#1 (in publication).jpg]

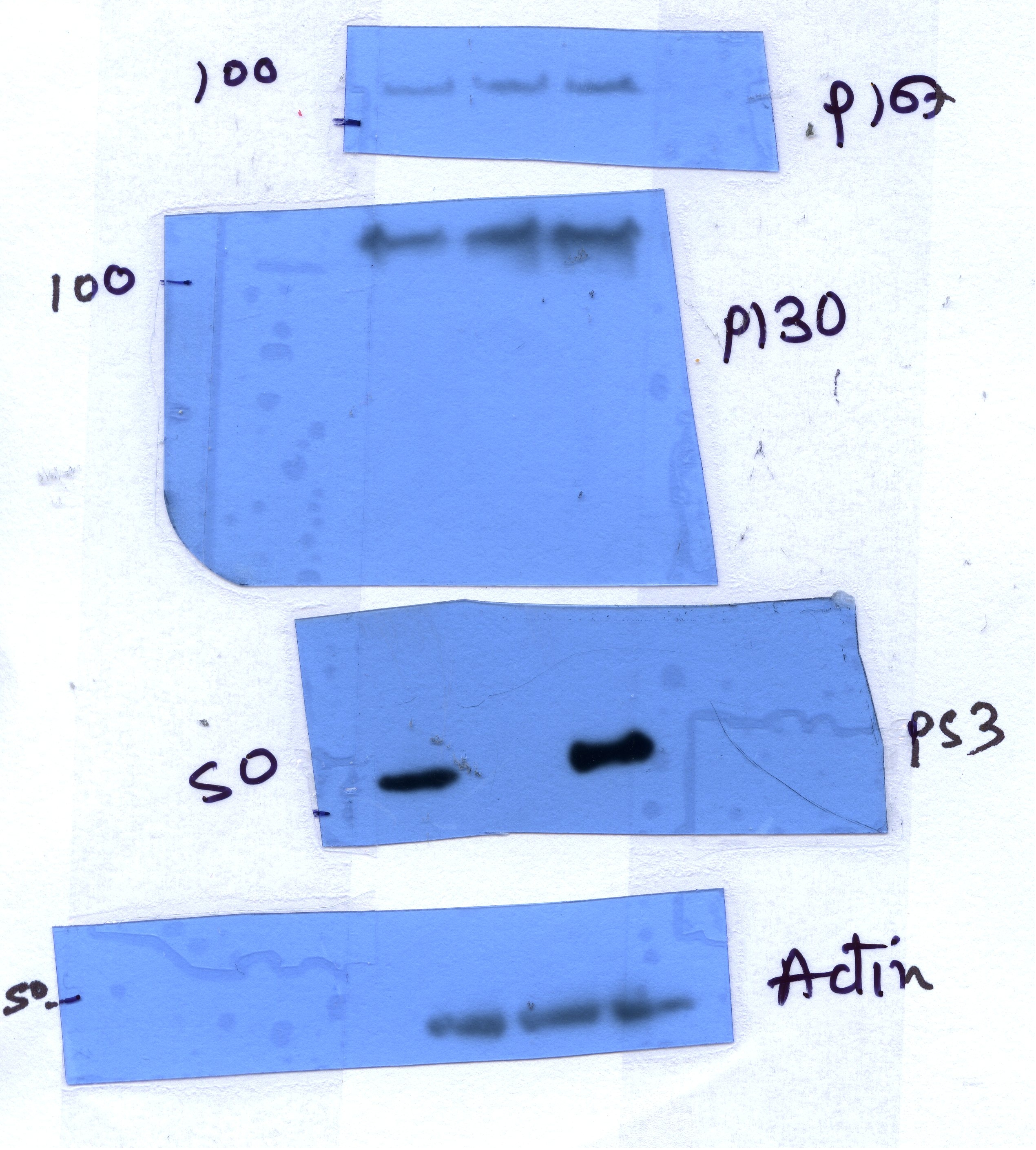

Supplement: Supplementary file 8 — Source data Fig. 3 [file 44318_2025_402_MOESM8_ESM.zip › SD Figure 3/3E/3E Western Replicate#2.jpg]

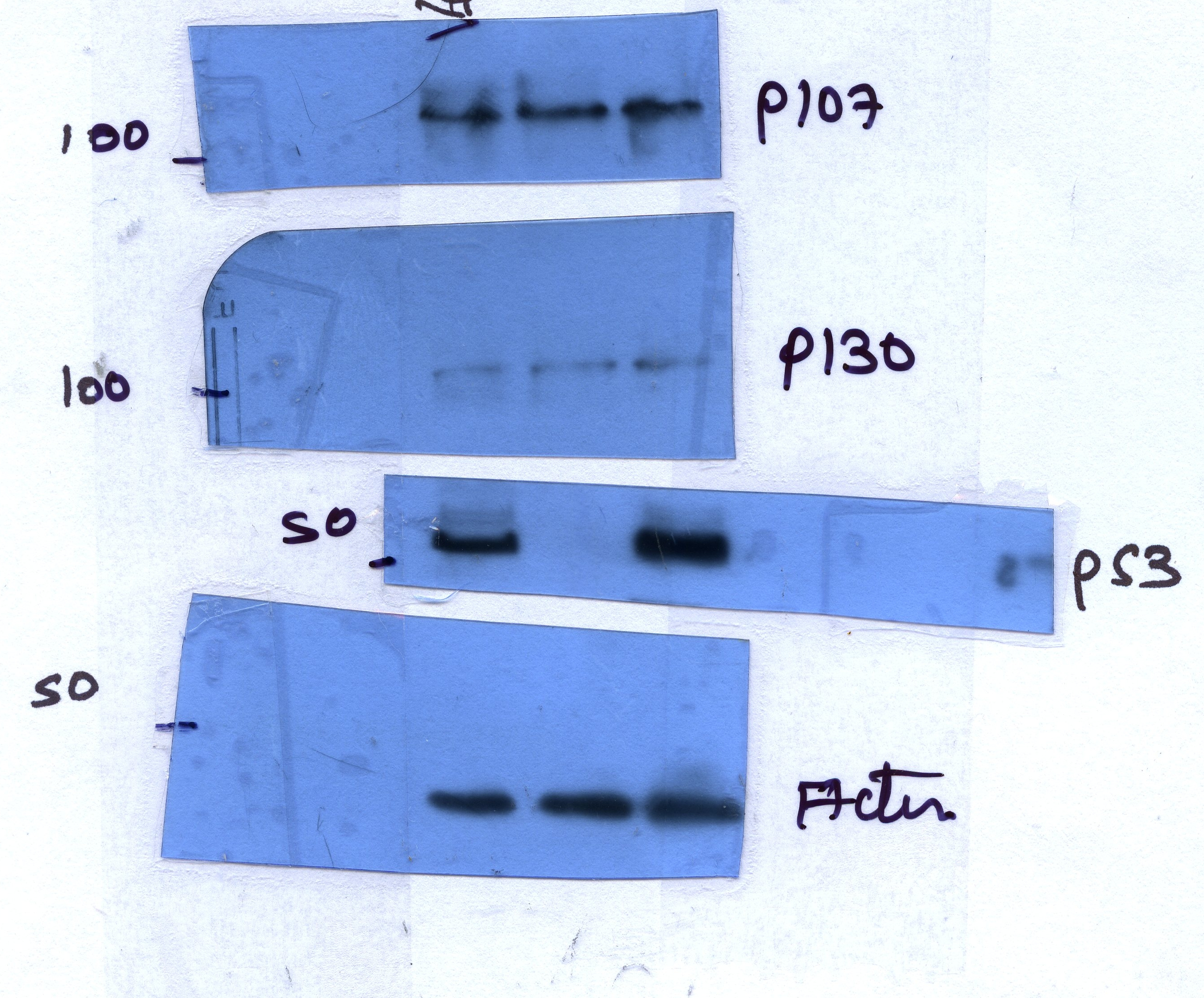

Supplement: Supplementary file 8 — Source data Fig. 3 [file 44318_2025_402_MOESM8_ESM.zip › SD Figure 3/3E/3E Western Replicate#3.jpg]

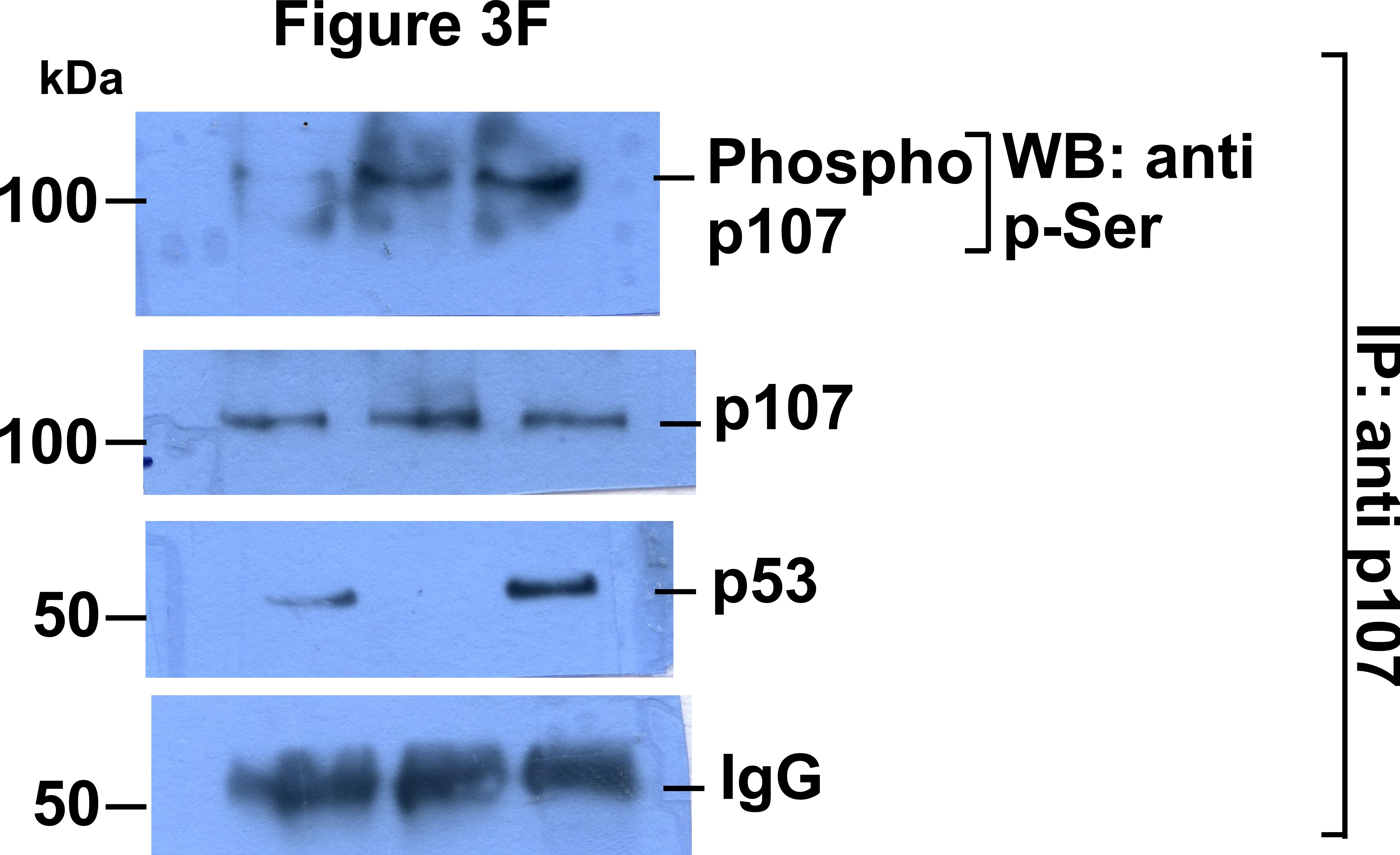

Supplement: Supplementary file 8 — Source data Fig. 3 [file 44318_2025_402_MOESM8_ESM.zip › SD Figure 3/3F/3F Western Replicate#1 (in publication).jpg]

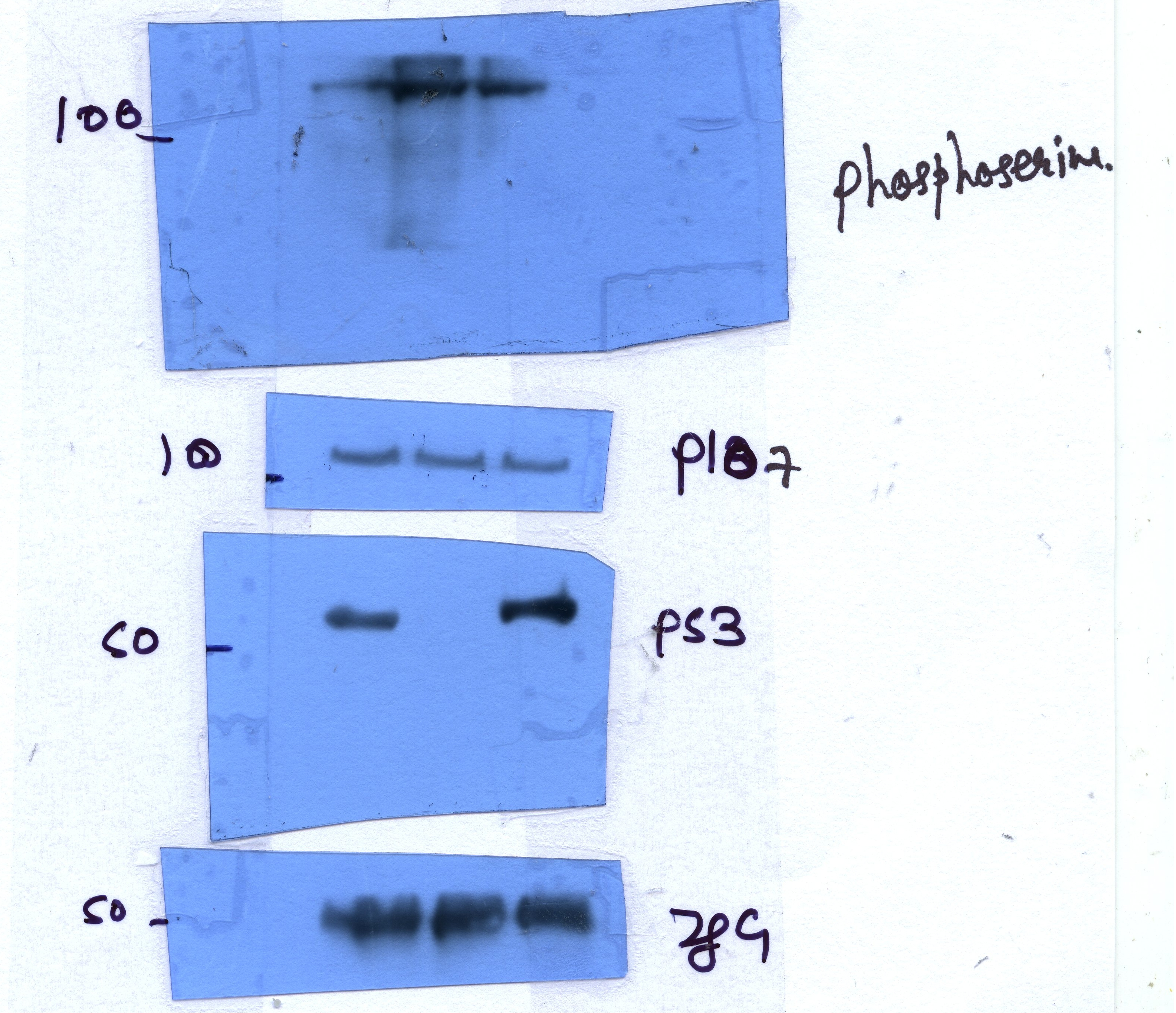

Supplement: Supplementary file 8 — Source data Fig. 3 [file 44318_2025_402_MOESM8_ESM.zip › SD Figure 3/3F/3F Western Replicate#2.jpg]

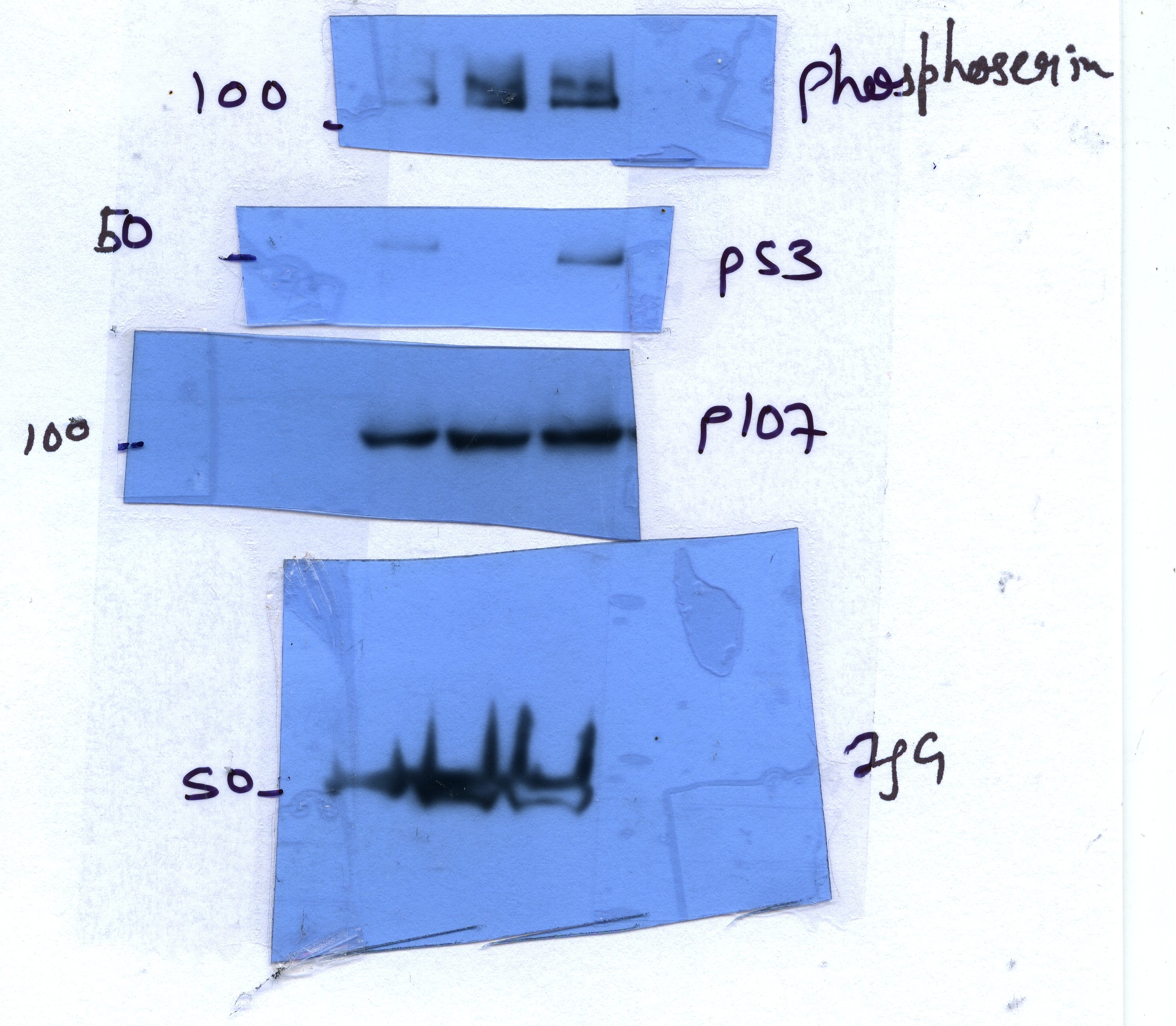

Supplement: Supplementary file 8 — Source data Fig. 3 [file 44318_2025_402_MOESM8_ESM.zip › SD Figure 3/3F/3F Western Replicate#3.jpg]

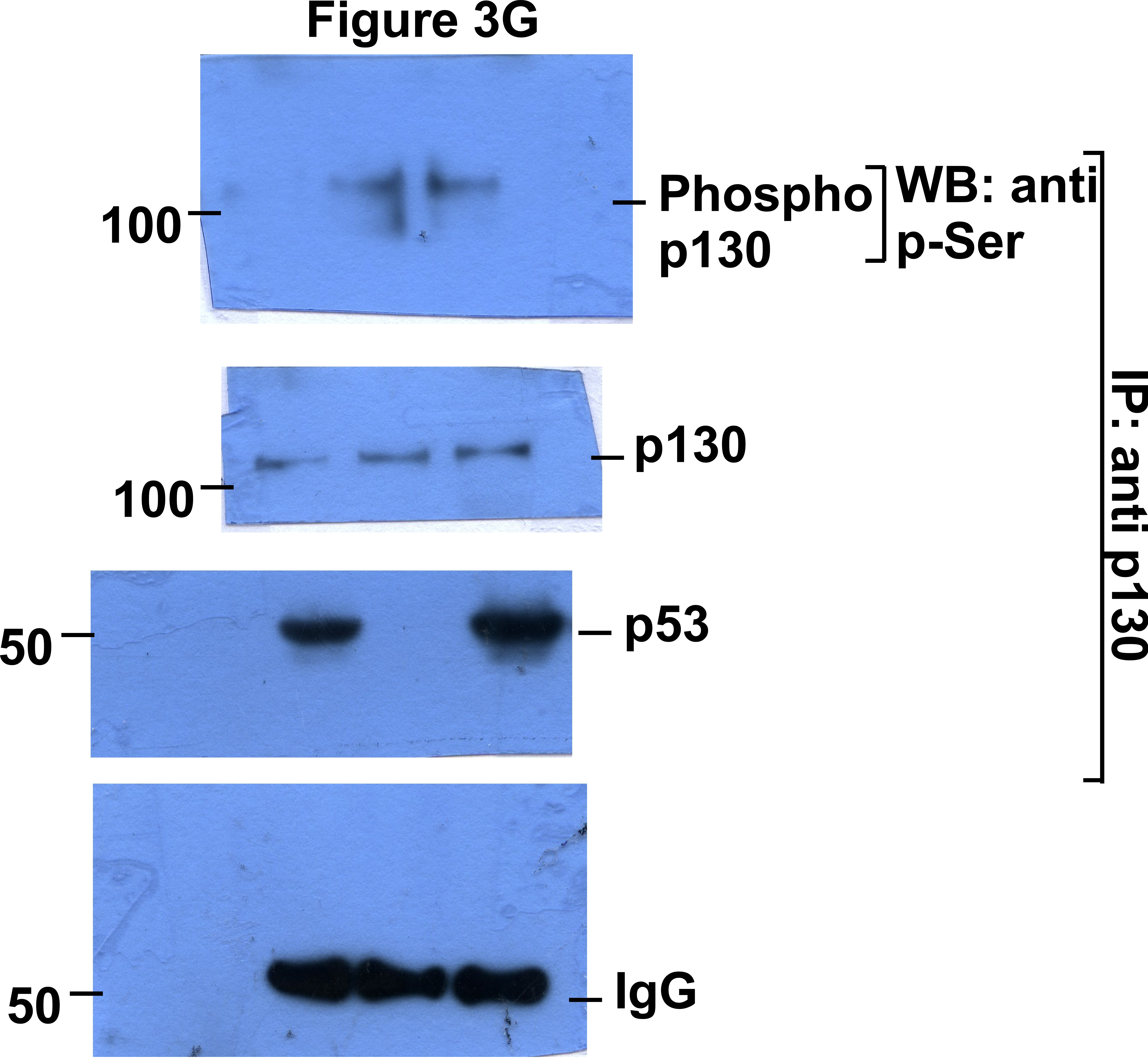

Supplement: Supplementary file 8 — Source data Fig. 3 [file 44318_2025_402_MOESM8_ESM.zip › SD Figure 3/3G/3G Western Replicate#1 (in publication).jpg]

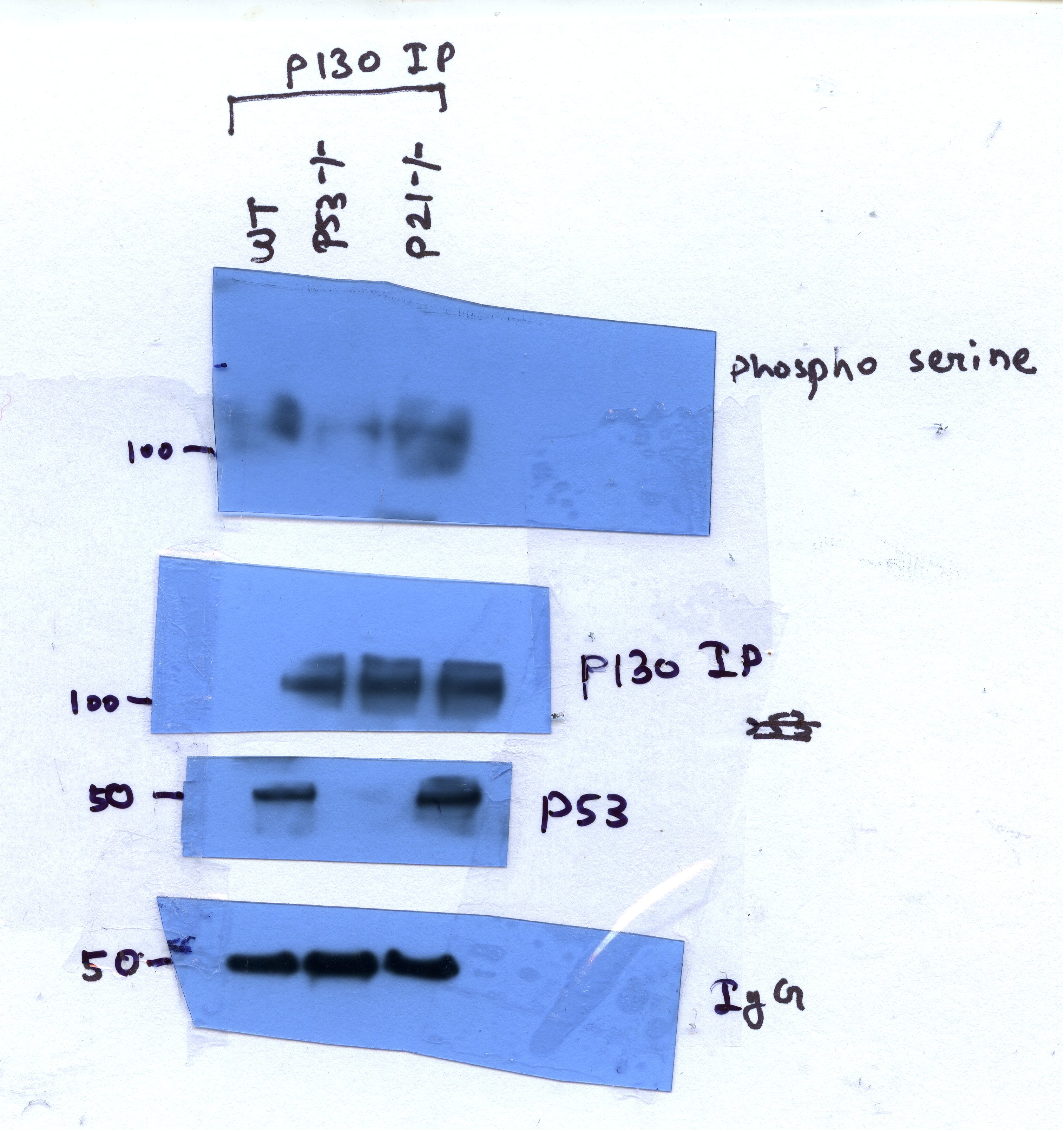

Supplement: Supplementary file 8 — Source data Fig. 3 [file 44318_2025_402_MOESM8_ESM.zip › SD Figure 3/3G/3G Western Replicate#2.jpg]

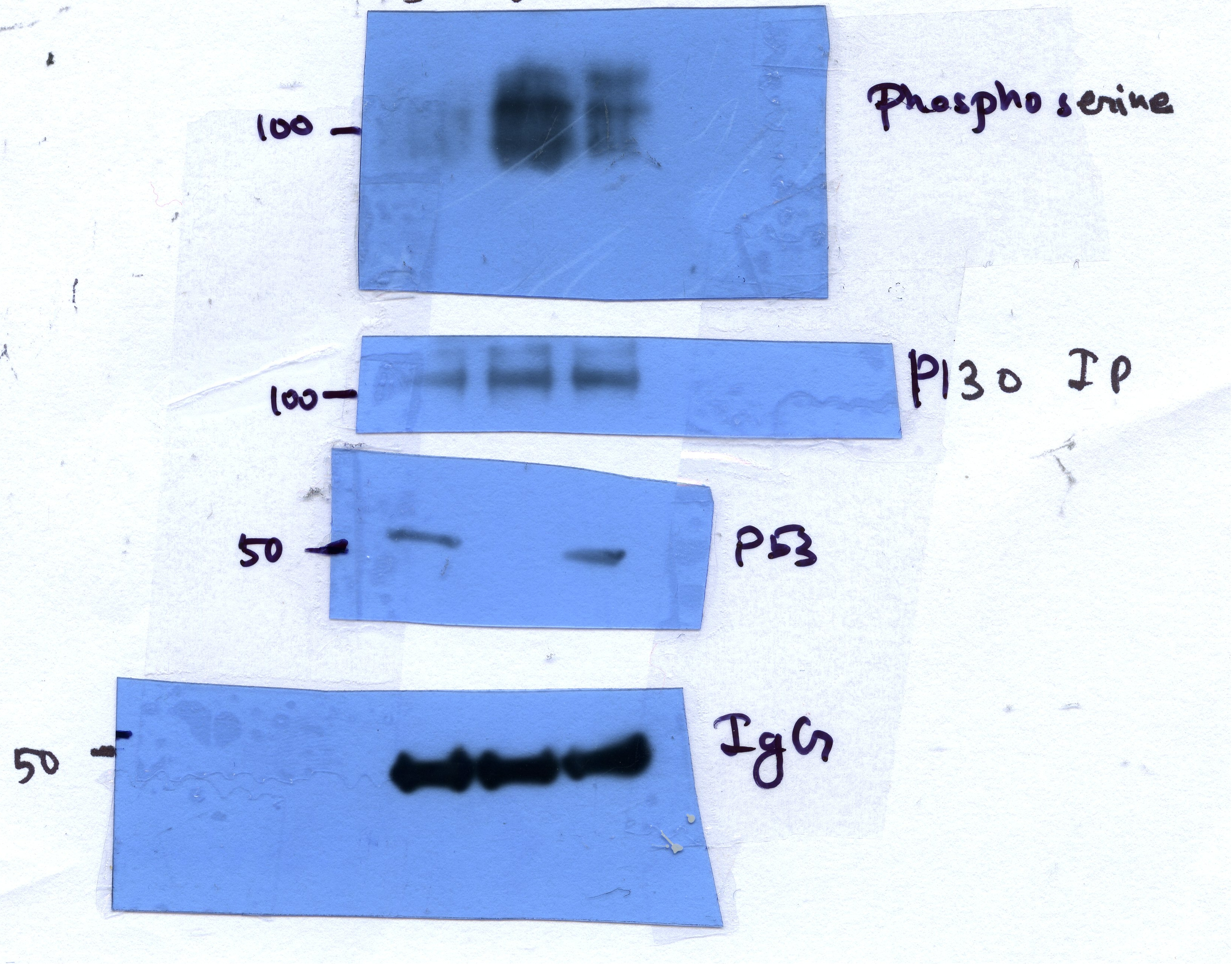

Supplement: Supplementary file 8 — Source data Fig. 3 [file 44318_2025_402_MOESM8_ESM.zip › SD Figure 3/3G/3G Western Replicate#3.jpg]

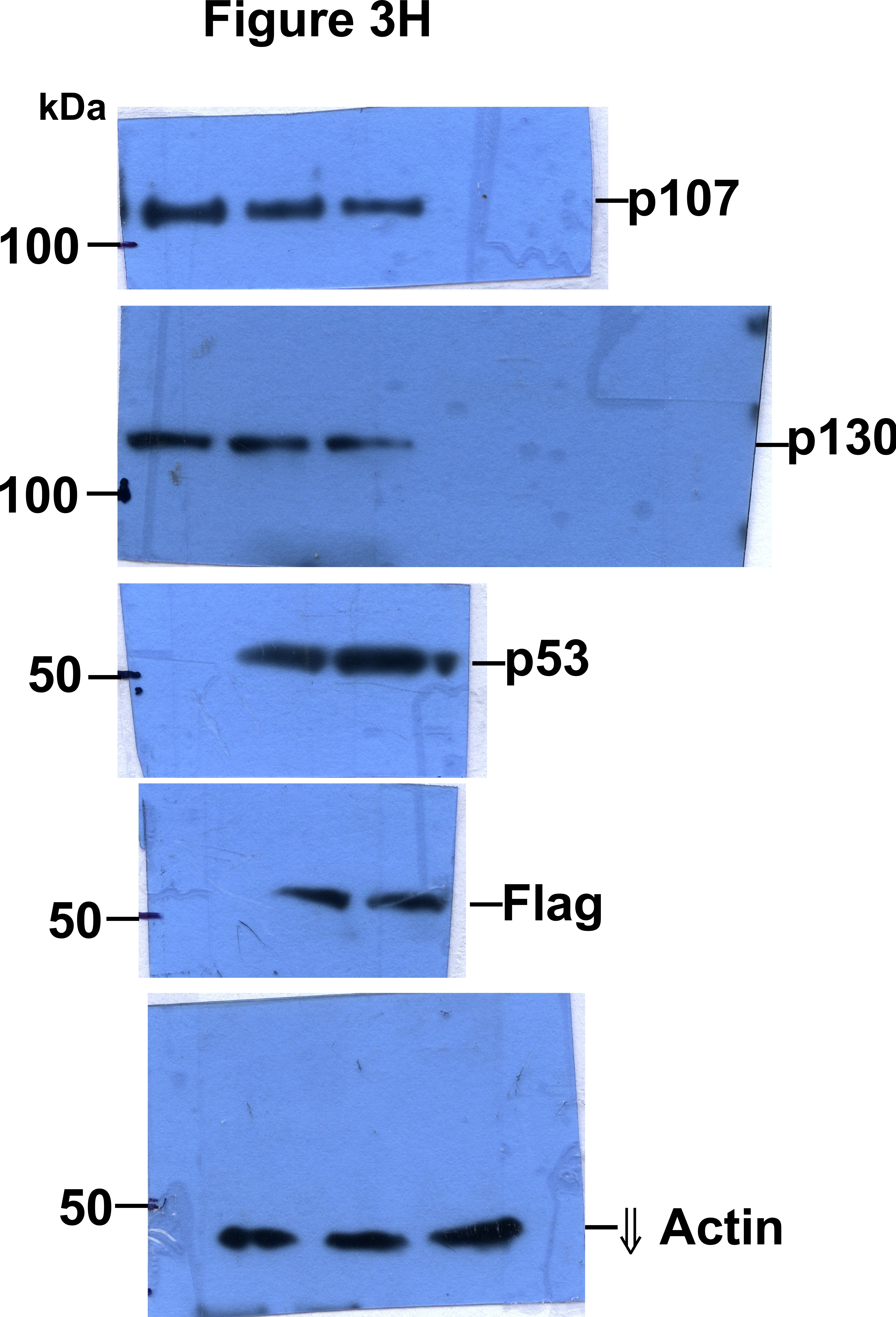

Supplement: Supplementary file 8 — Source data Fig. 3 [file 44318_2025_402_MOESM8_ESM.zip › SD Figure 3/3H/3H Western Replicate#1 (in publication).jpg]

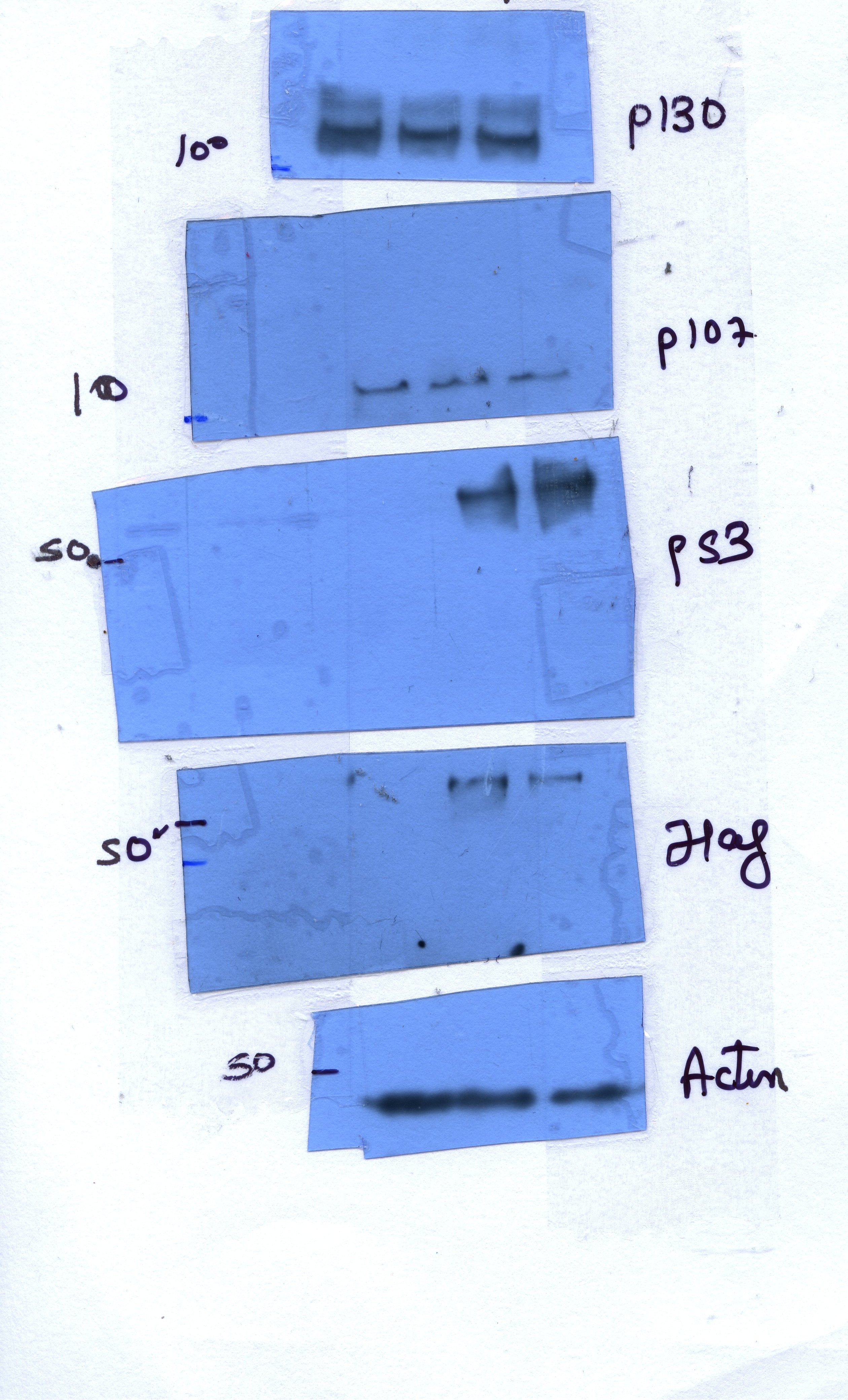

Supplement: Supplementary file 8 — Source data Fig. 3 [file 44318_2025_402_MOESM8_ESM.zip › SD Figure 3/3H/3H Western Replicate#2.jpg]

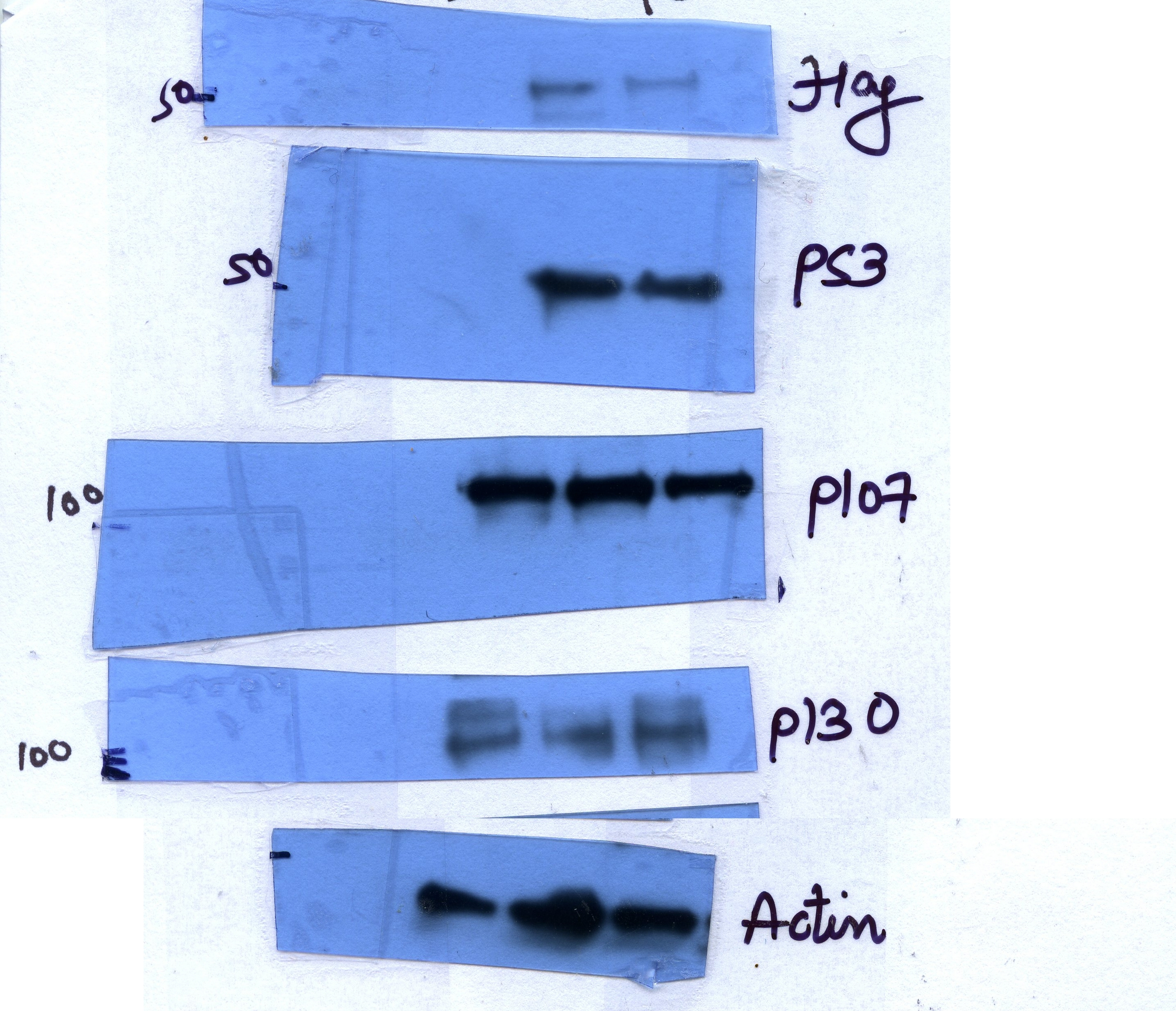

Supplement: Supplementary file 8 — Source data Fig. 3 [file 44318_2025_402_MOESM8_ESM.zip › SD Figure 3/3H/3H Western Replicate#3.jpg]

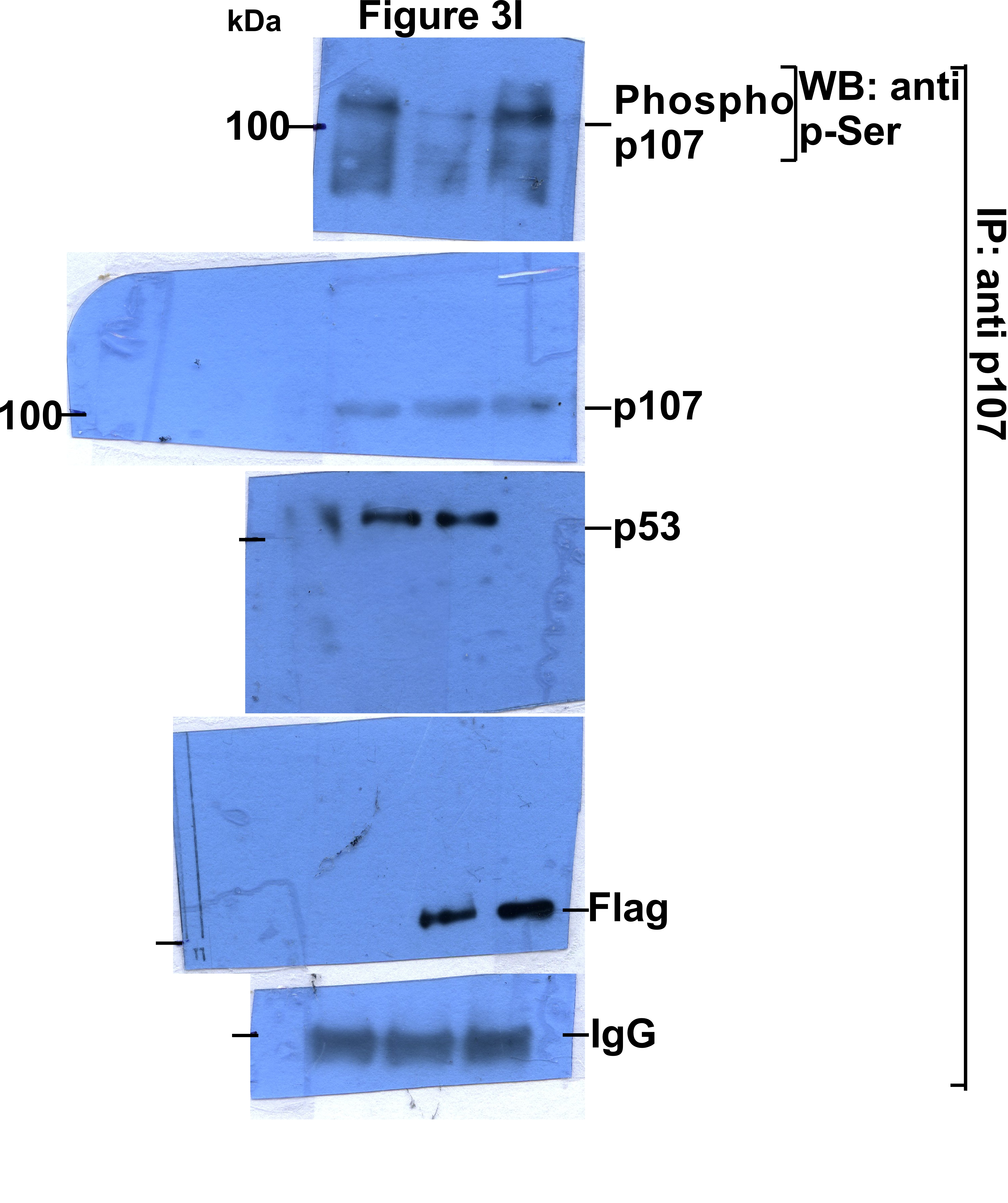

Supplement: Supplementary file 8 — Source data Fig. 3 [file 44318_2025_402_MOESM8_ESM.zip › SD Figure 3/3I/3I Western Replicate#1 (in publication).jpg]

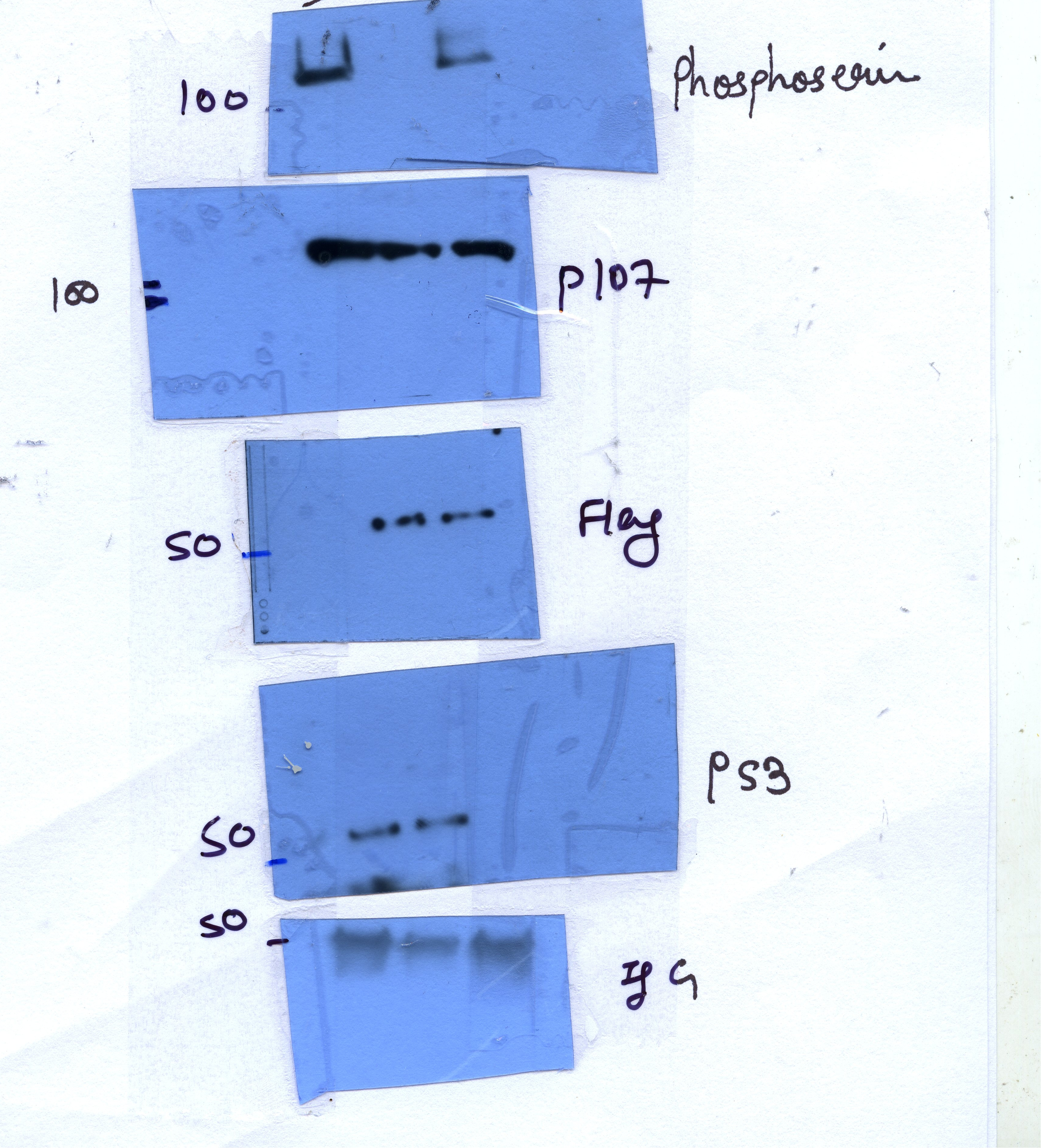

Supplement: Supplementary file 8 — Source data Fig. 3 [file 44318_2025_402_MOESM8_ESM.zip › SD Figure 3/3I/3I Western Replicate#2.jpg]

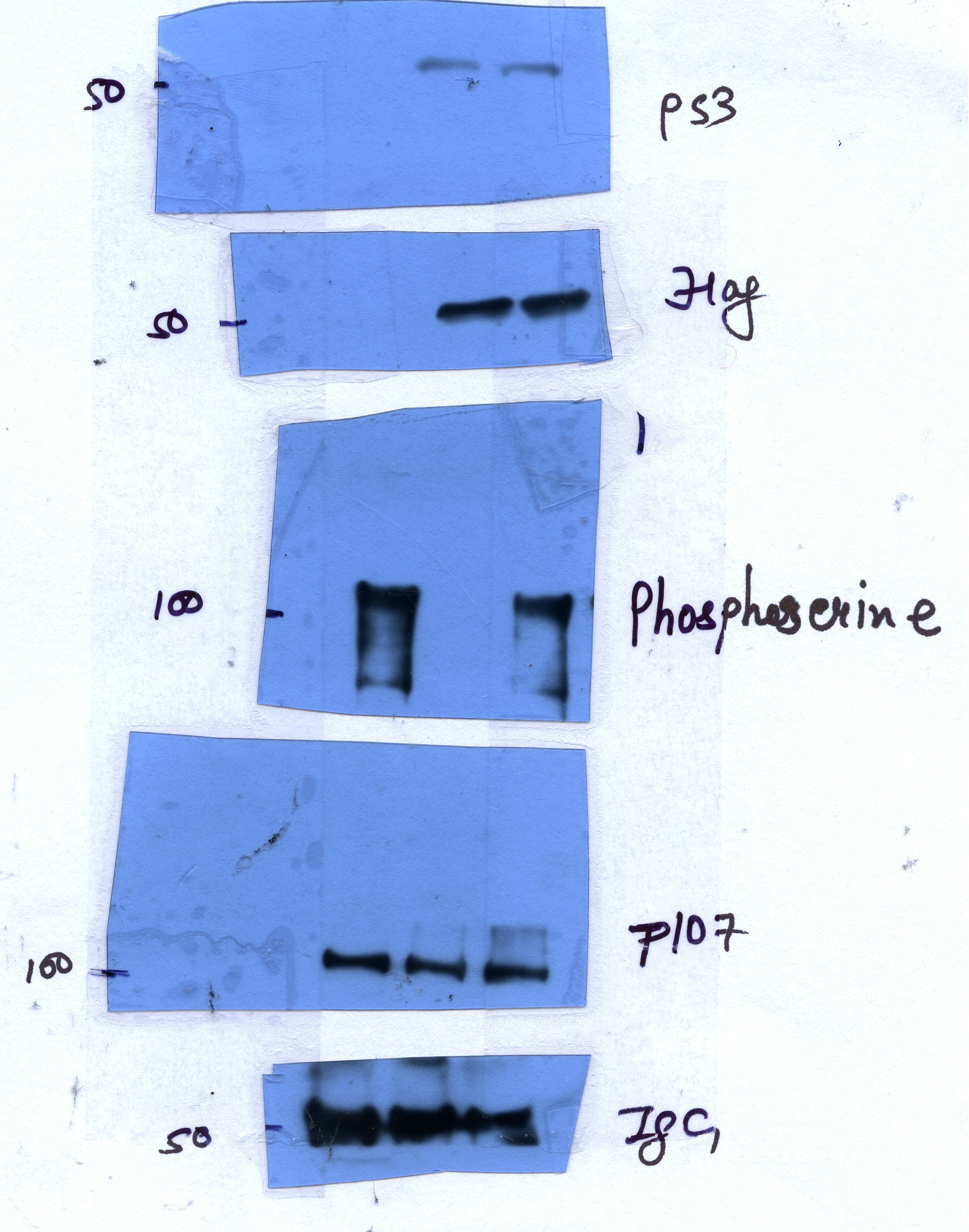

Supplement: Supplementary file 8 — Source data Fig. 3 [file 44318_2025_402_MOESM8_ESM.zip › SD Figure 3/3I/3I Western Replicate#3.jpg]

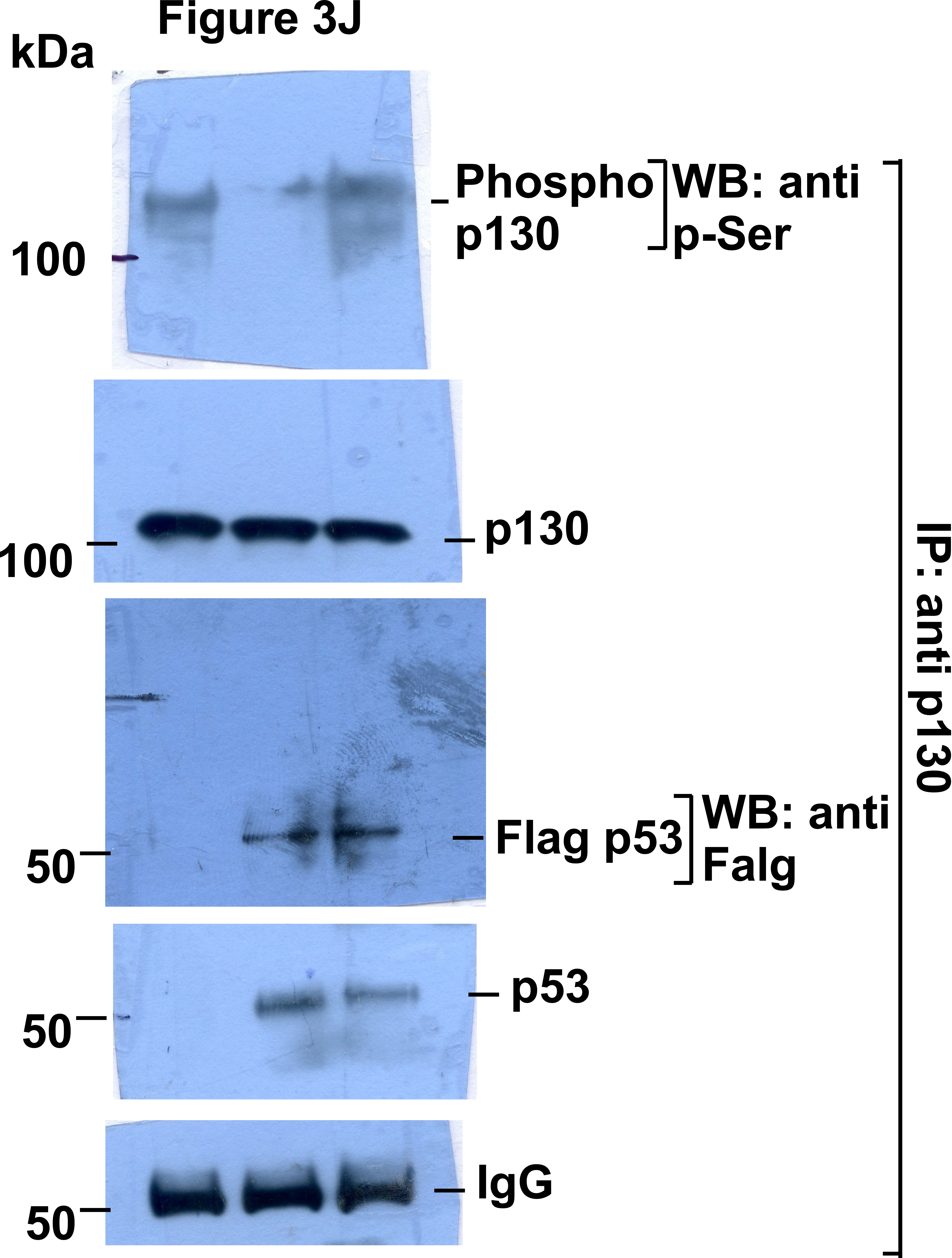

Supplement: Supplementary file 8 — Source data Fig. 3 [file 44318_2025_402_MOESM8_ESM.zip › SD Figure 3/3J/3J Western Replicate#1 (in publication).jpg]

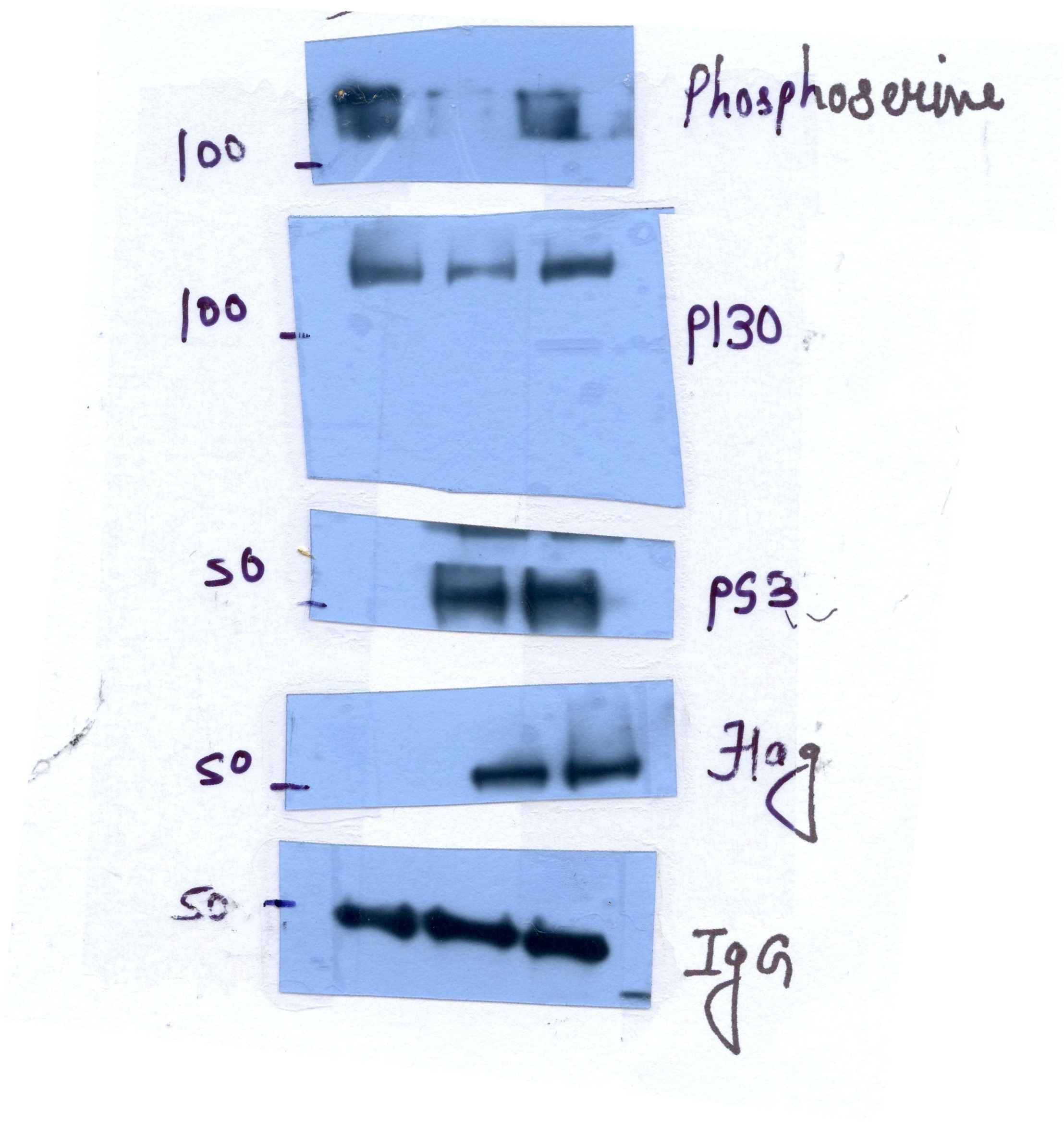

Supplement: Supplementary file 8 — Source data Fig. 3 [file 44318_2025_402_MOESM8_ESM.zip › SD Figure 3/3J/3J Western Replicate#2.jpg]

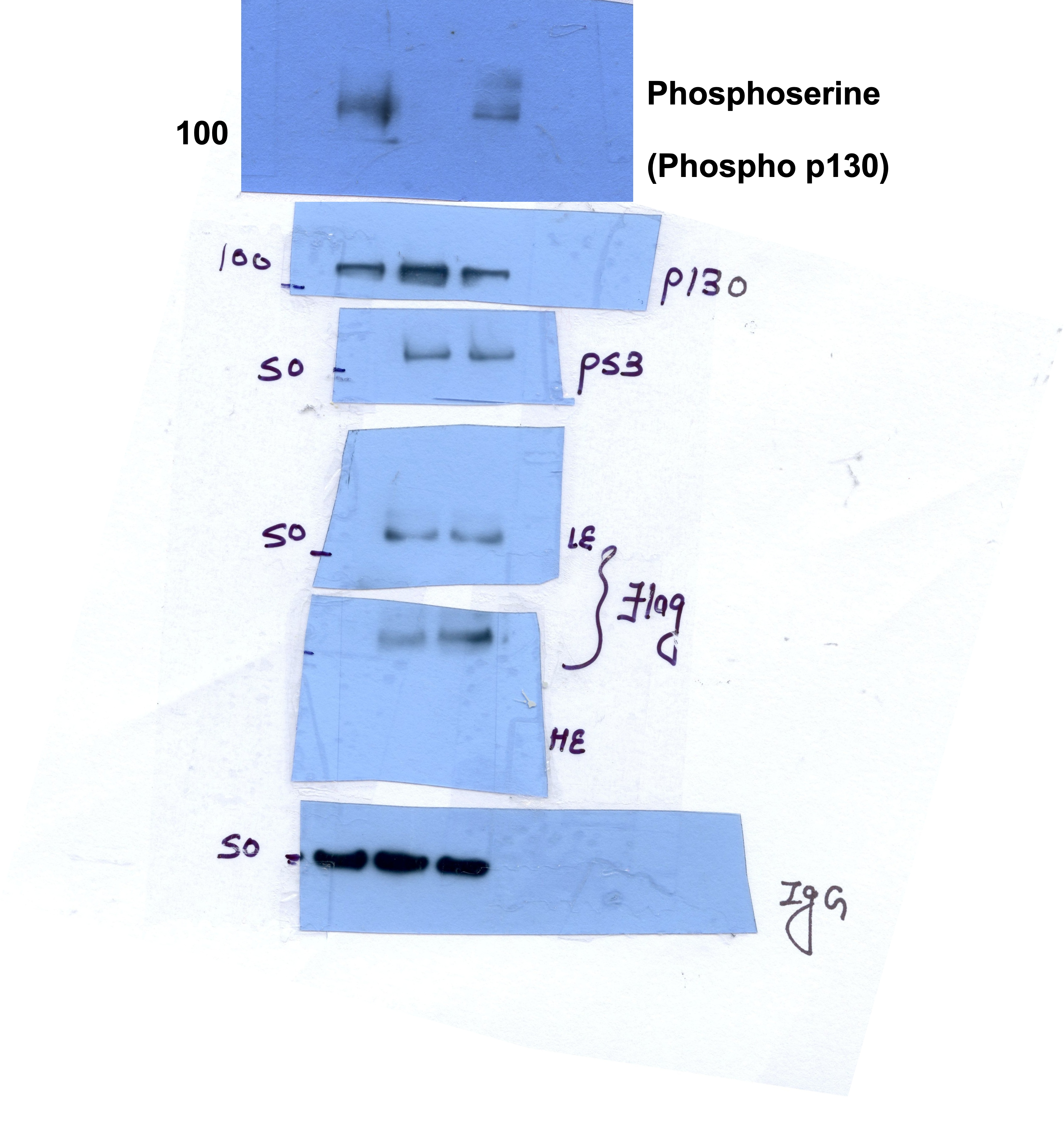

Supplement: Supplementary file 8 — Source data Fig. 3 [file 44318_2025_402_MOESM8_ESM.zip › SD Figure 3/3J/3J Western Replicate#3.jpg]

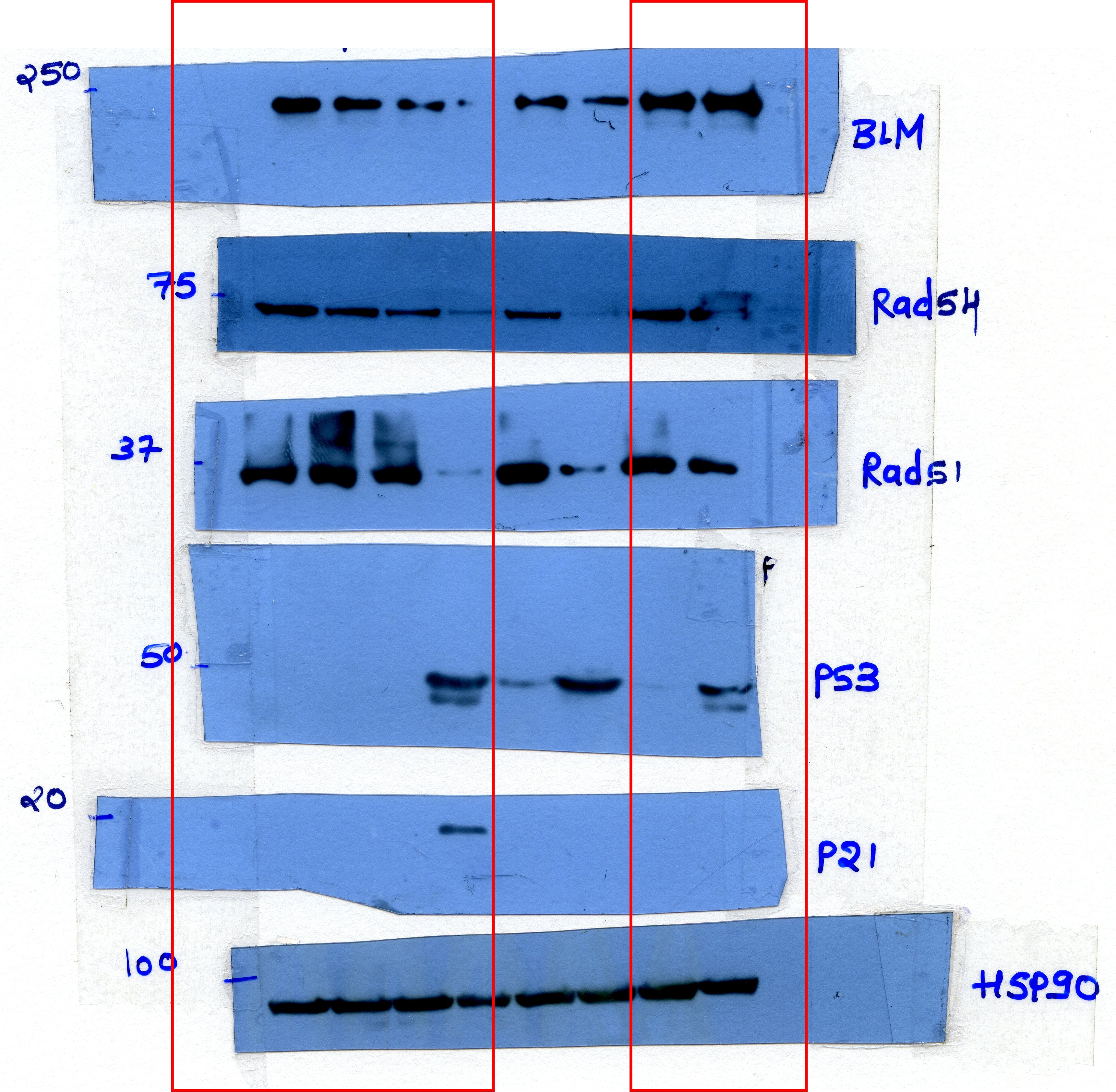

Supplement: Supplementary file 9 — Source data Fig. 4 [file 44318_2025_402_MOESM9_ESM.zip › SD Figure 4/4A/4A Western Replicate#1.jpg]

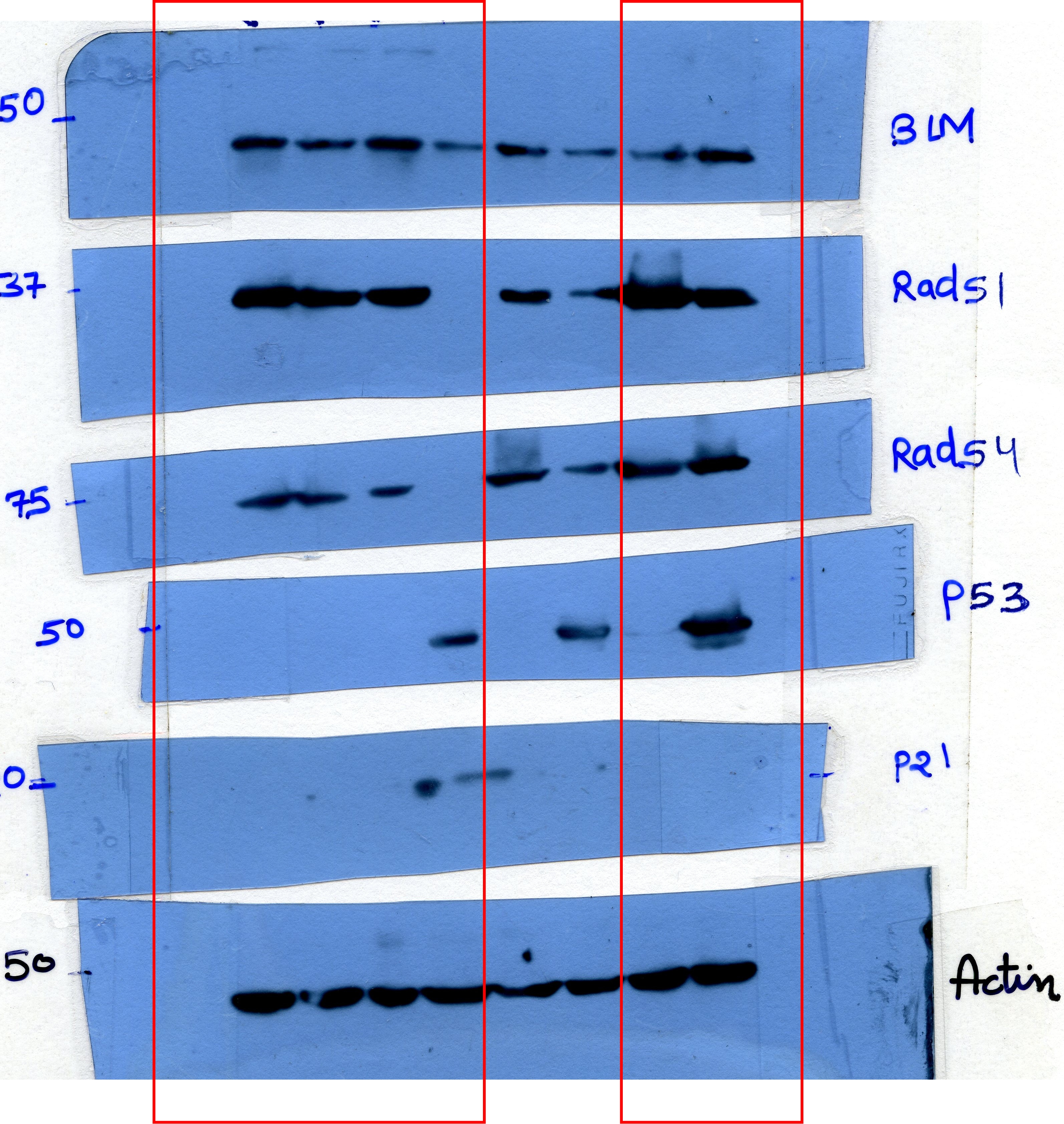

Supplement: Supplementary file 9 — Source data Fig. 4 [file 44318_2025_402_MOESM9_ESM.zip › SD Figure 4/4A/4A Western Replicate#2.jpg]

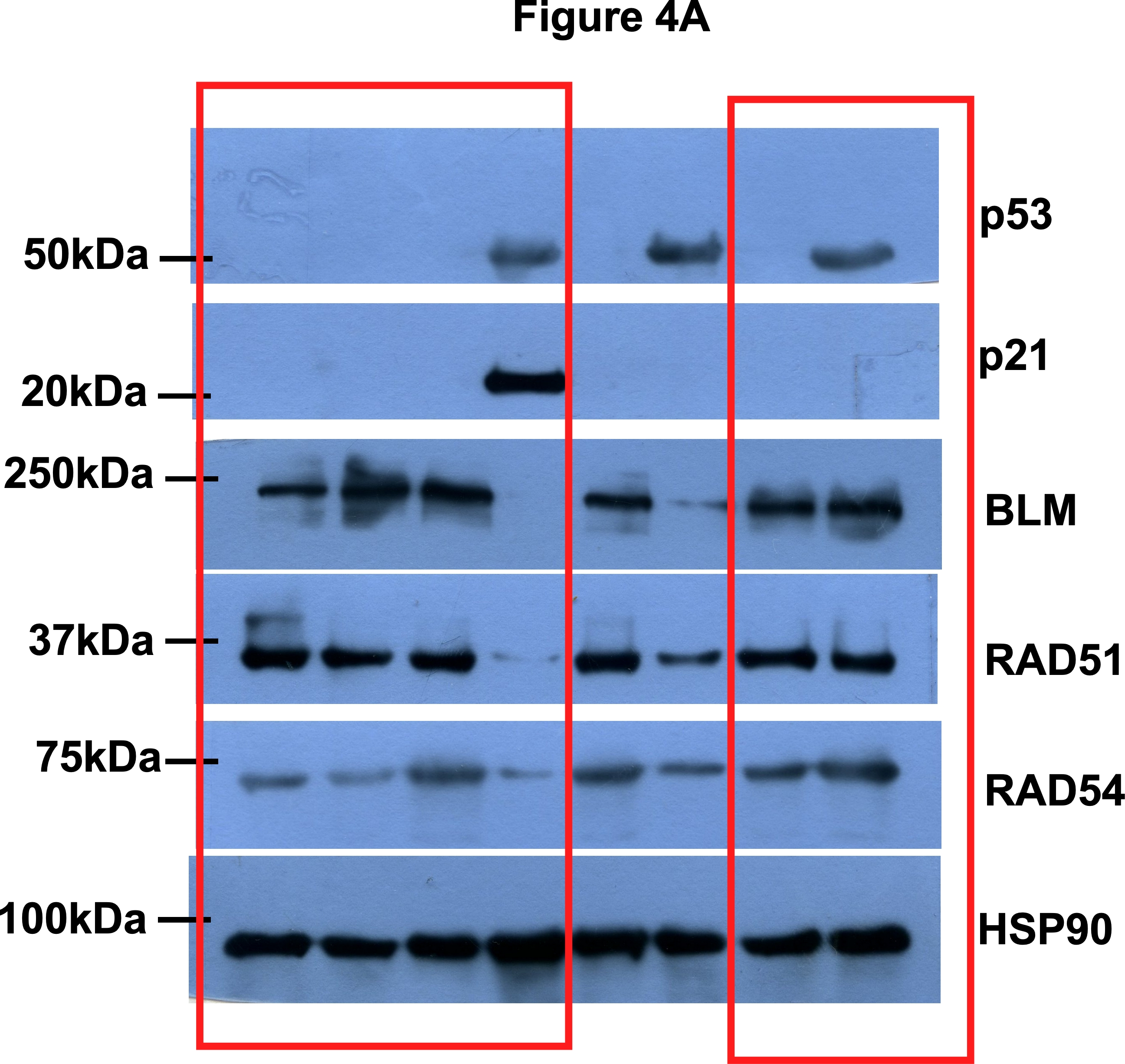

Supplement: Supplementary file 9 — Source data Fig. 4 [file 44318_2025_402_MOESM9_ESM.zip › SD Figure 4/4A/4A Western Replicate#3 (in publication).jpg]

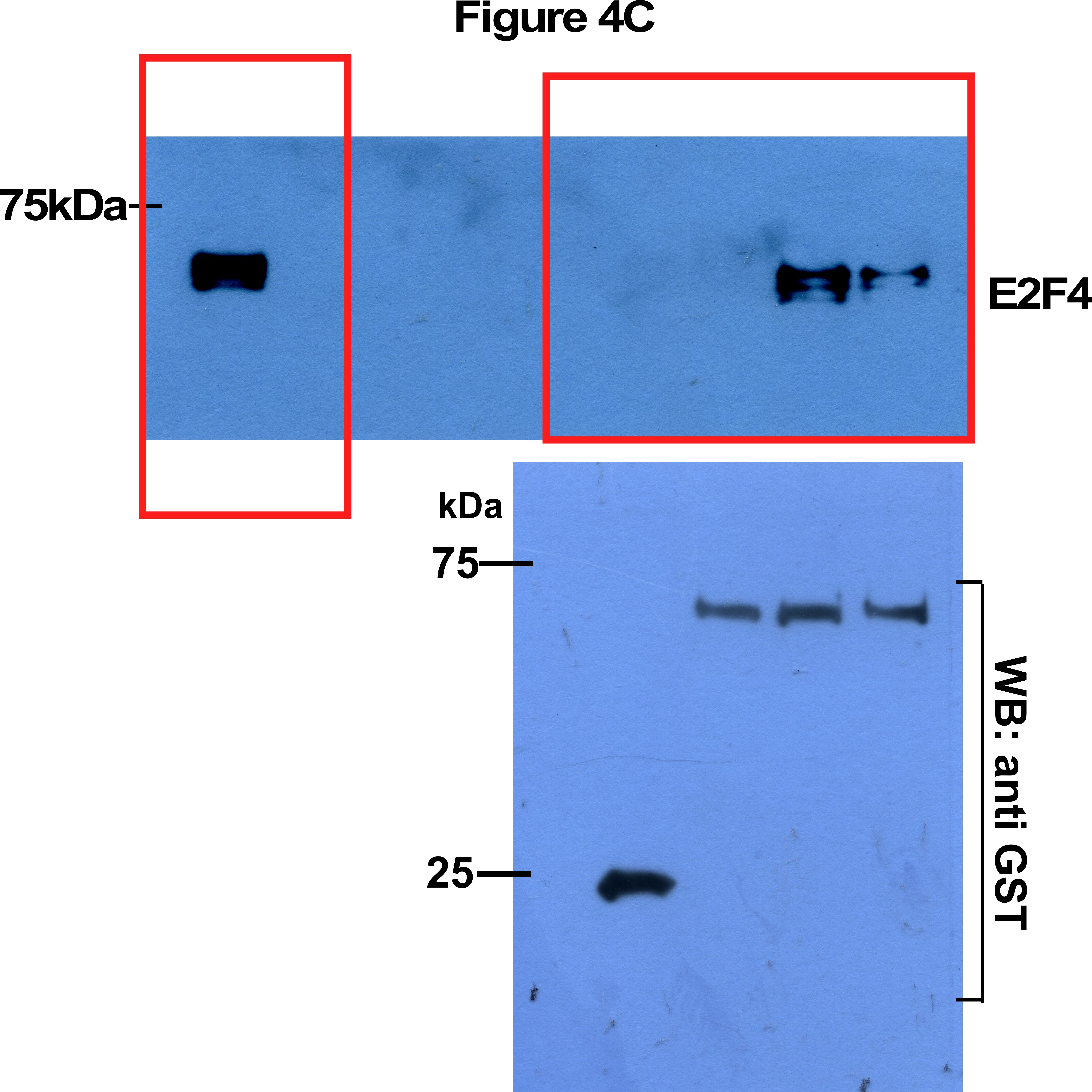

Supplement: Supplementary file 9 — Source data Fig. 4 [file 44318_2025_402_MOESM9_ESM.zip › SD Figure 4/4C/4C Western Replicate#1 (in publication).jpg]

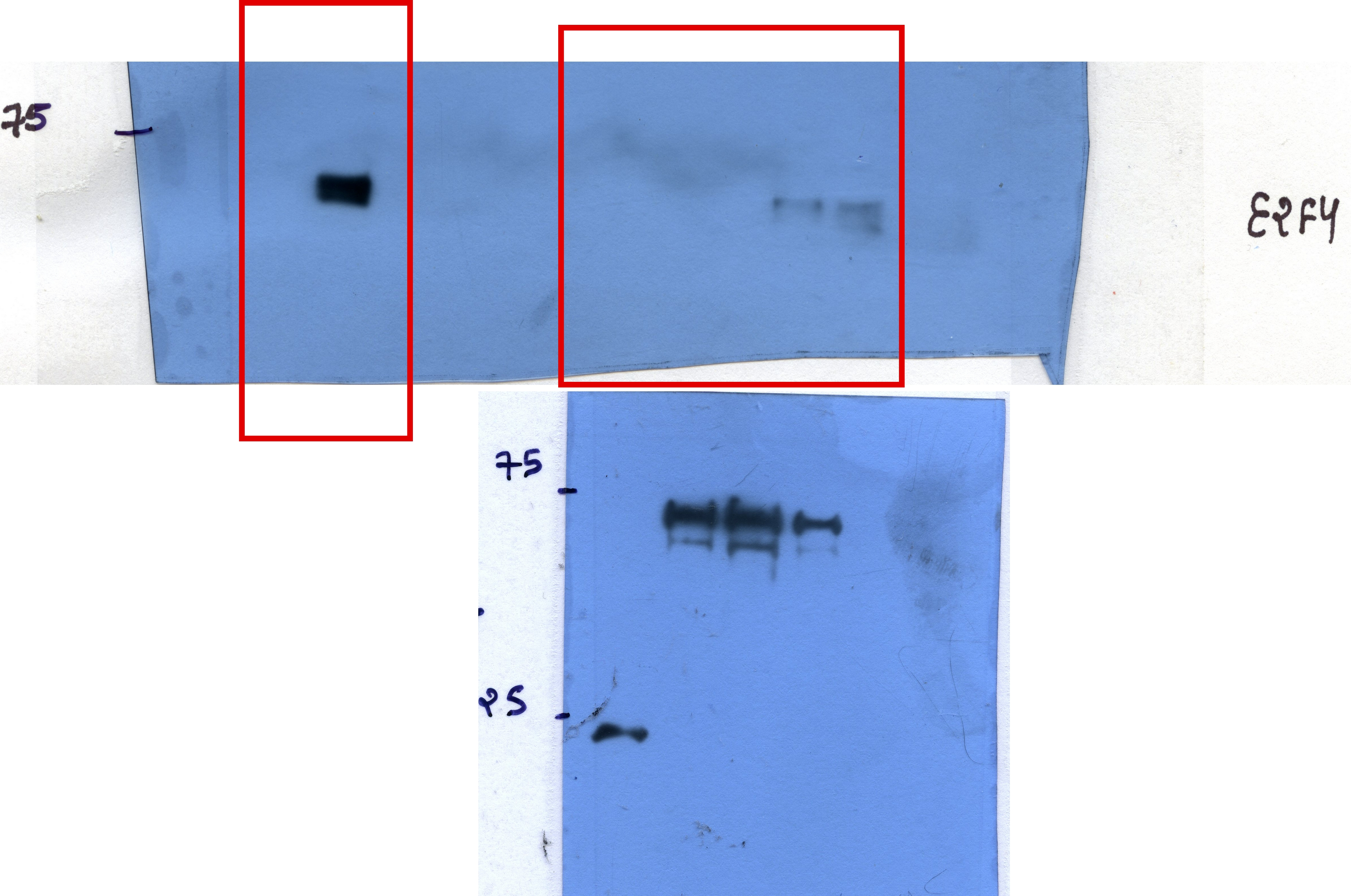

Supplement: Supplementary file 9 — Source data Fig. 4 [file 44318_2025_402_MOESM9_ESM.zip › SD Figure 4/4C/4C Western Replicate#2.jpg]

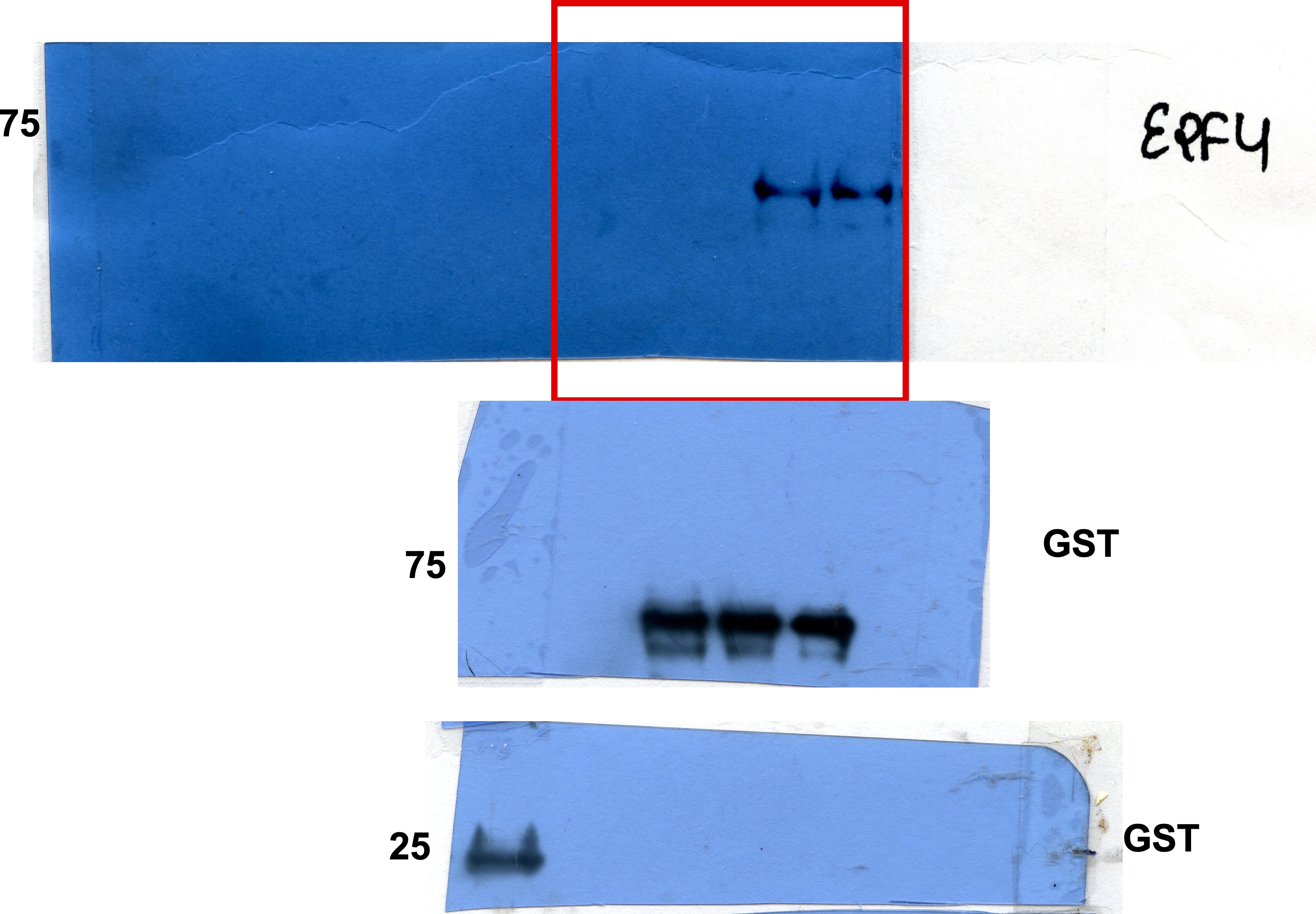

Supplement: Supplementary file 9 — Source data Fig. 4 [file 44318_2025_402_MOESM9_ESM.zip › SD Figure 4/4C/4C Western Replicate#3.jpg]

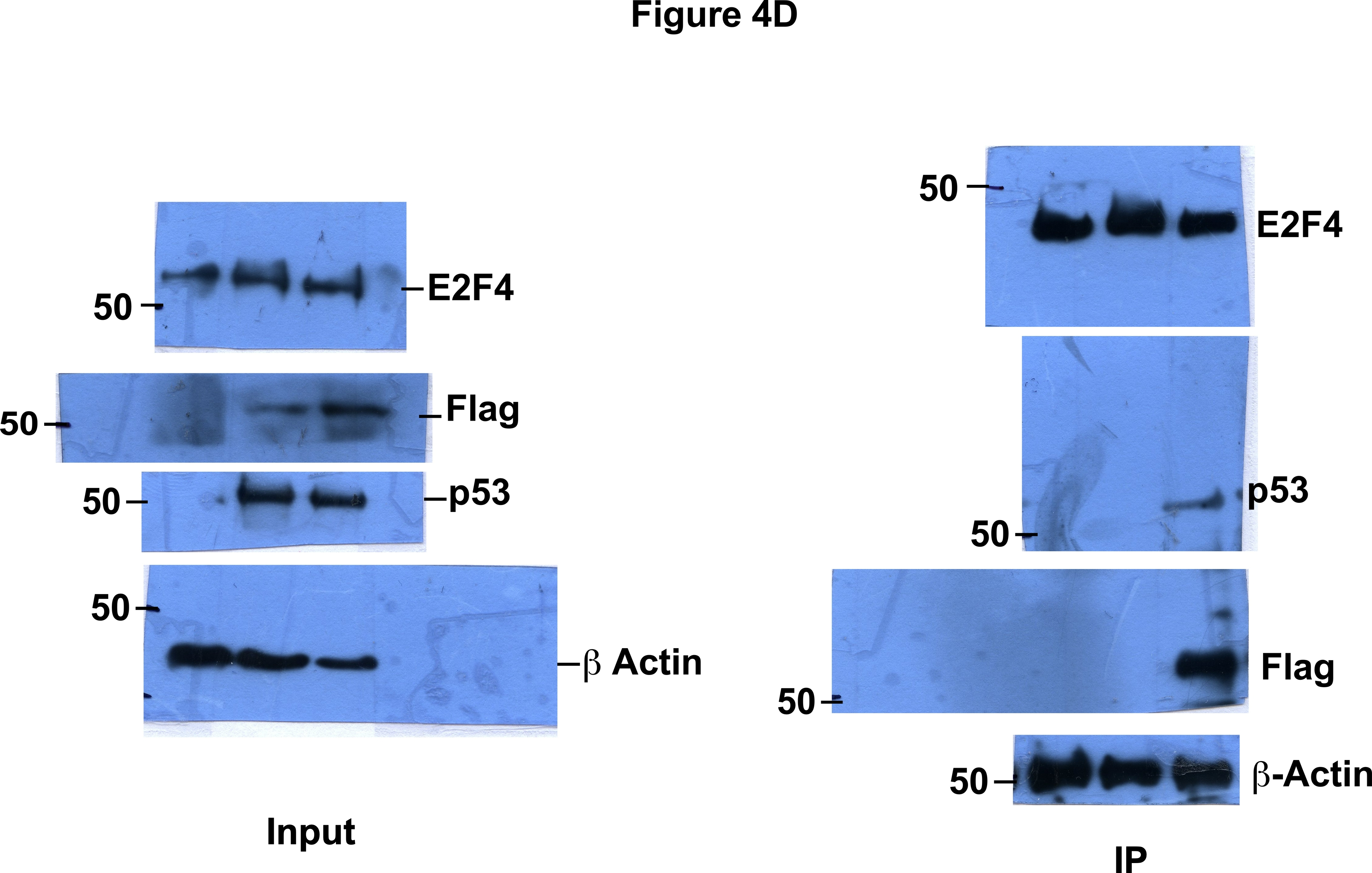

Supplement: Supplementary file 9 — Source data Fig. 4 [file 44318_2025_402_MOESM9_ESM.zip › SD Figure 4/4D/4D Western Replicate#1 (in publication).jpg]

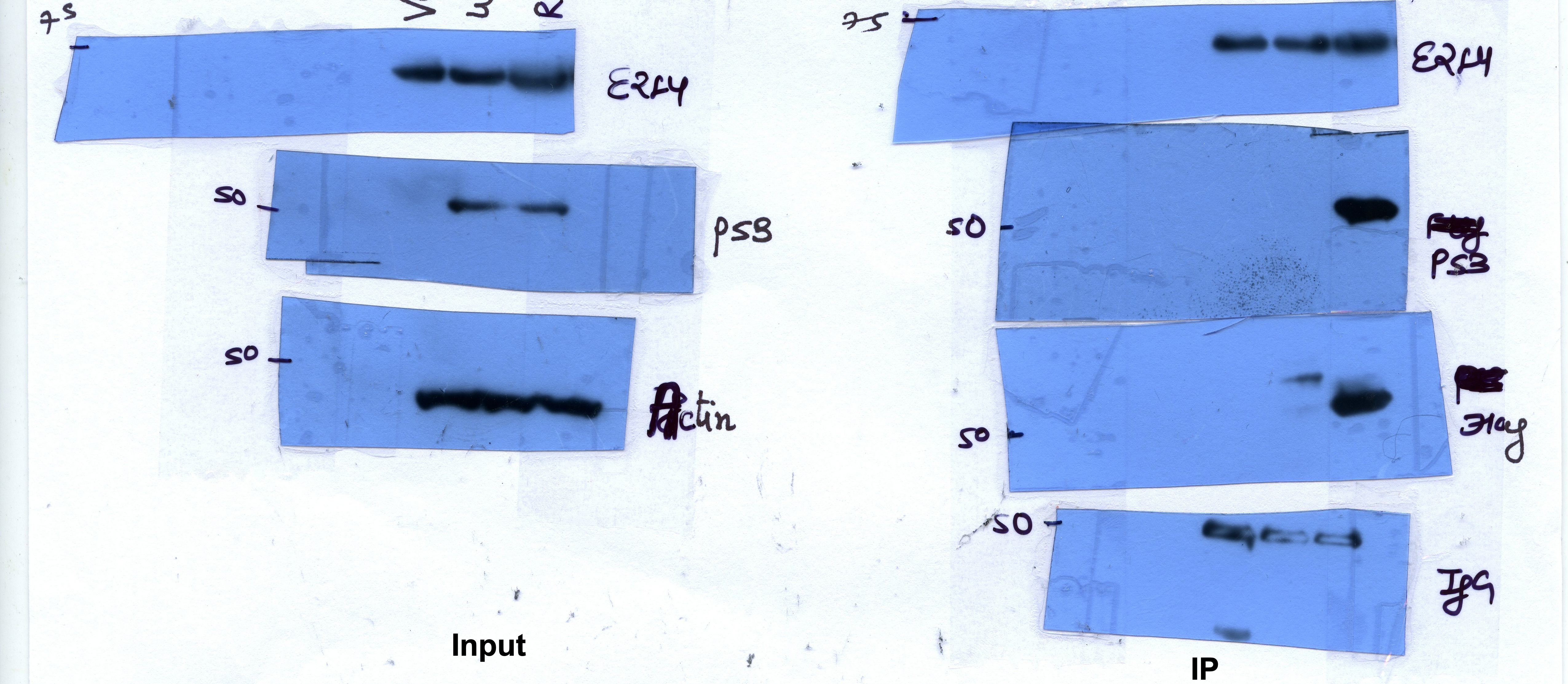

Supplement: Supplementary file 9 — Source data Fig. 4 [file 44318_2025_402_MOESM9_ESM.zip › SD Figure 4/4D/4D Western Replicate#2 .jpg]

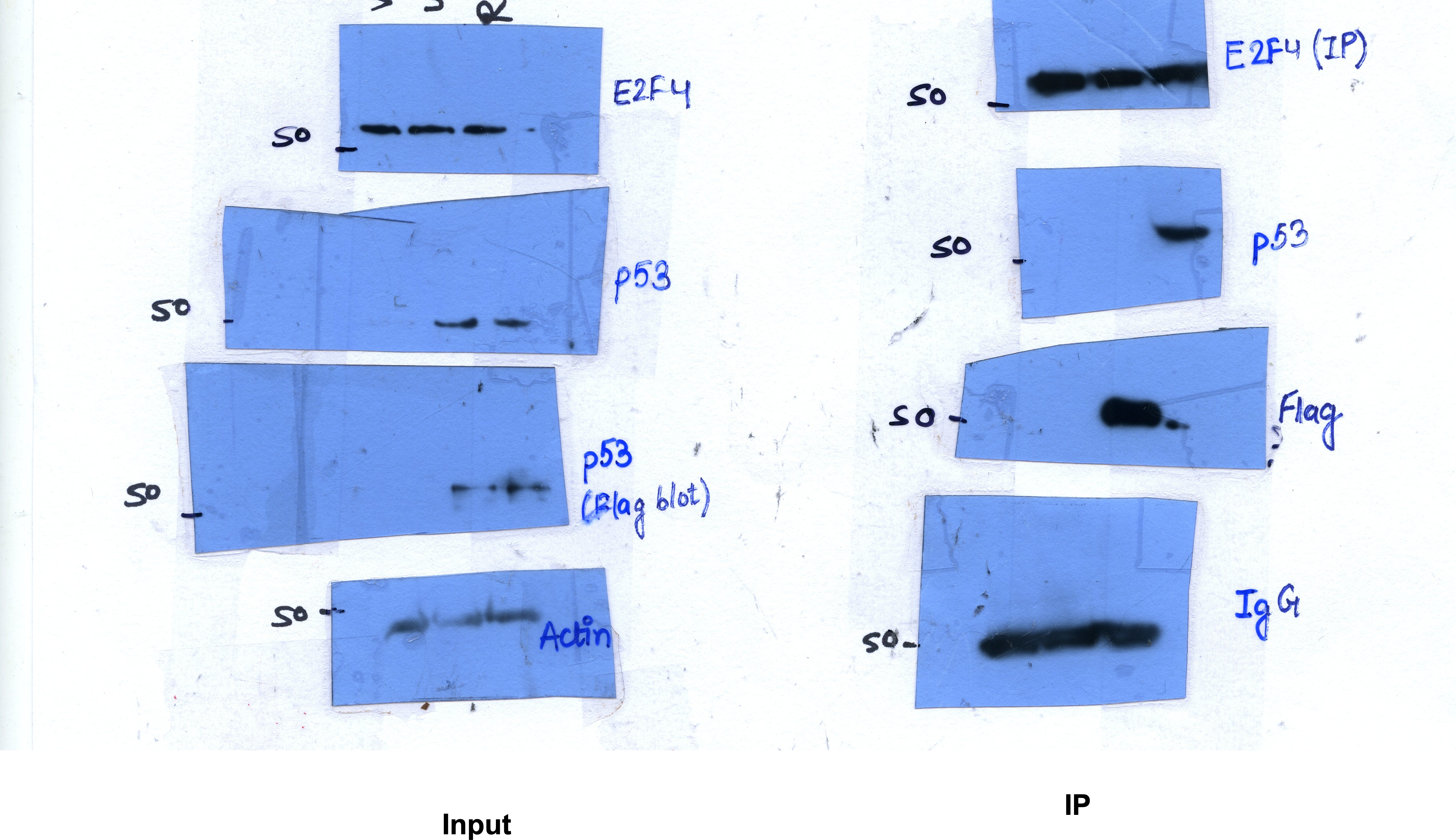

Supplement: Supplementary file 9 — Source data Fig. 4 [file 44318_2025_402_MOESM9_ESM.zip › SD Figure 4/4D/4D Western Replicate#3.jpg]

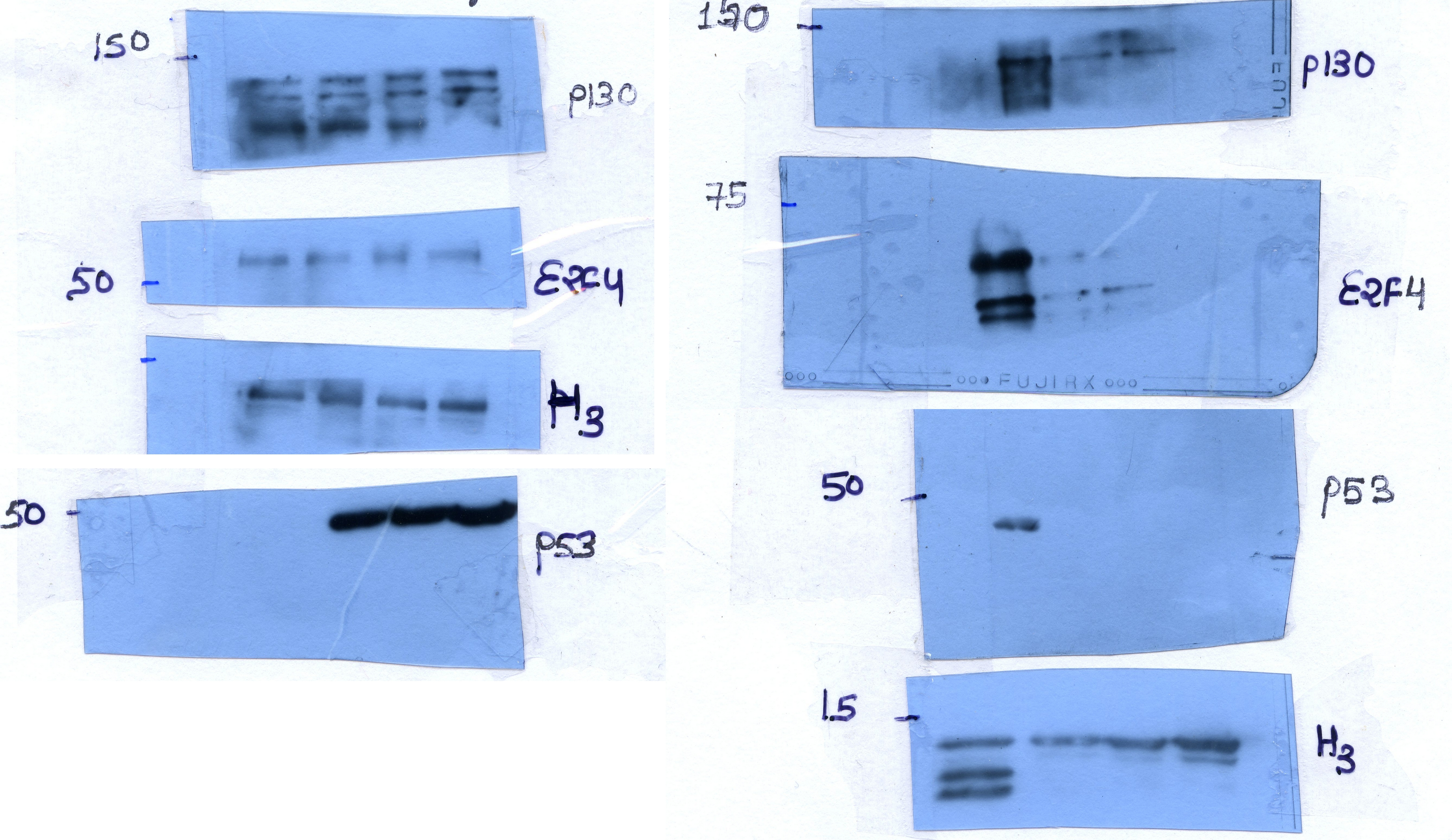

Supplement: Supplementary file 9 — Source data Fig. 4 [file 44318_2025_402_MOESM9_ESM.zip › SD Figure 4/4E/4E Western Replicate#1 .jpg]

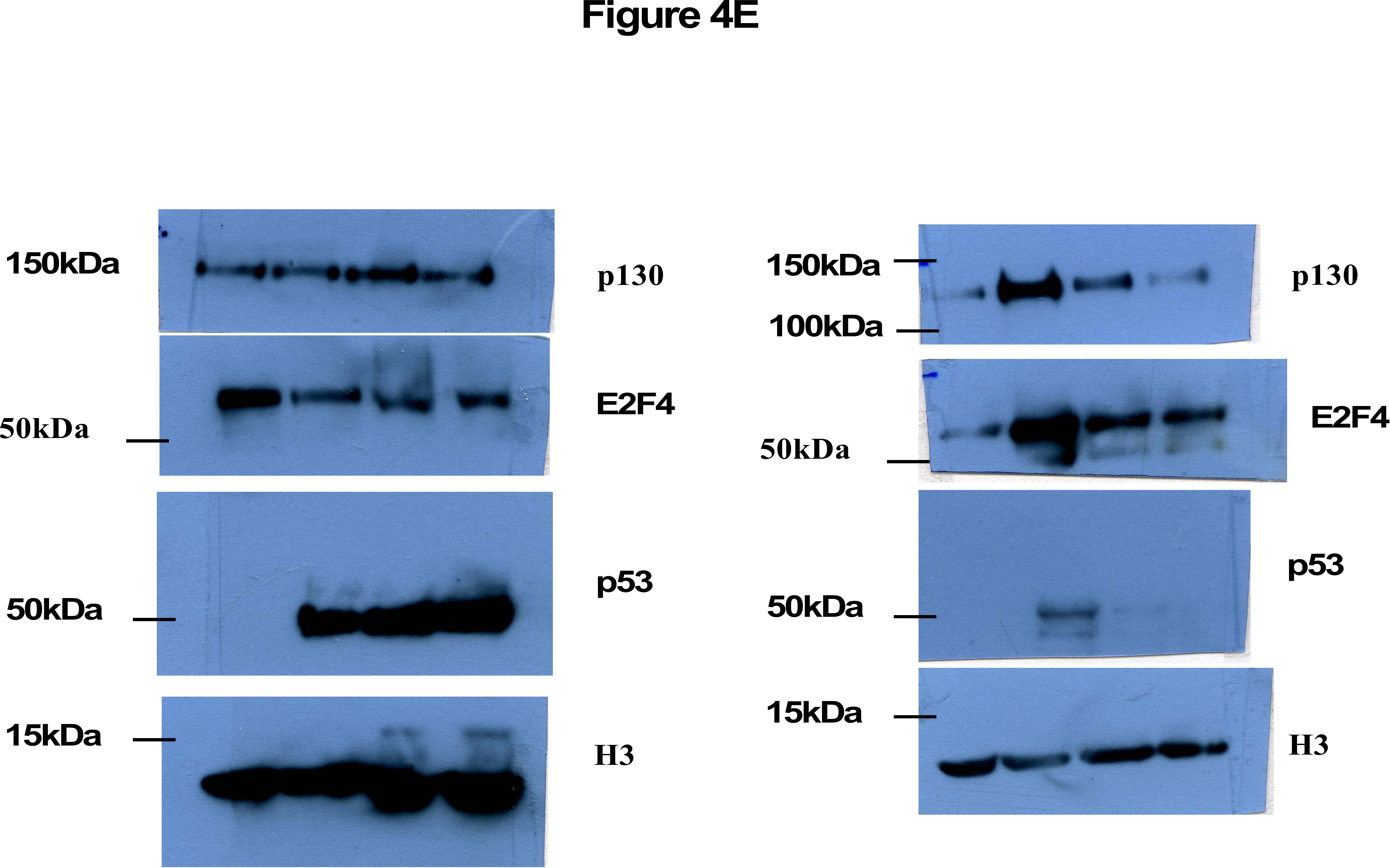

Supplement: Supplementary file 9 — Source data Fig. 4 [file 44318_2025_402_MOESM9_ESM.zip › SD Figure 4/4E/4E Western Replicate#2 (in publication).jpg]

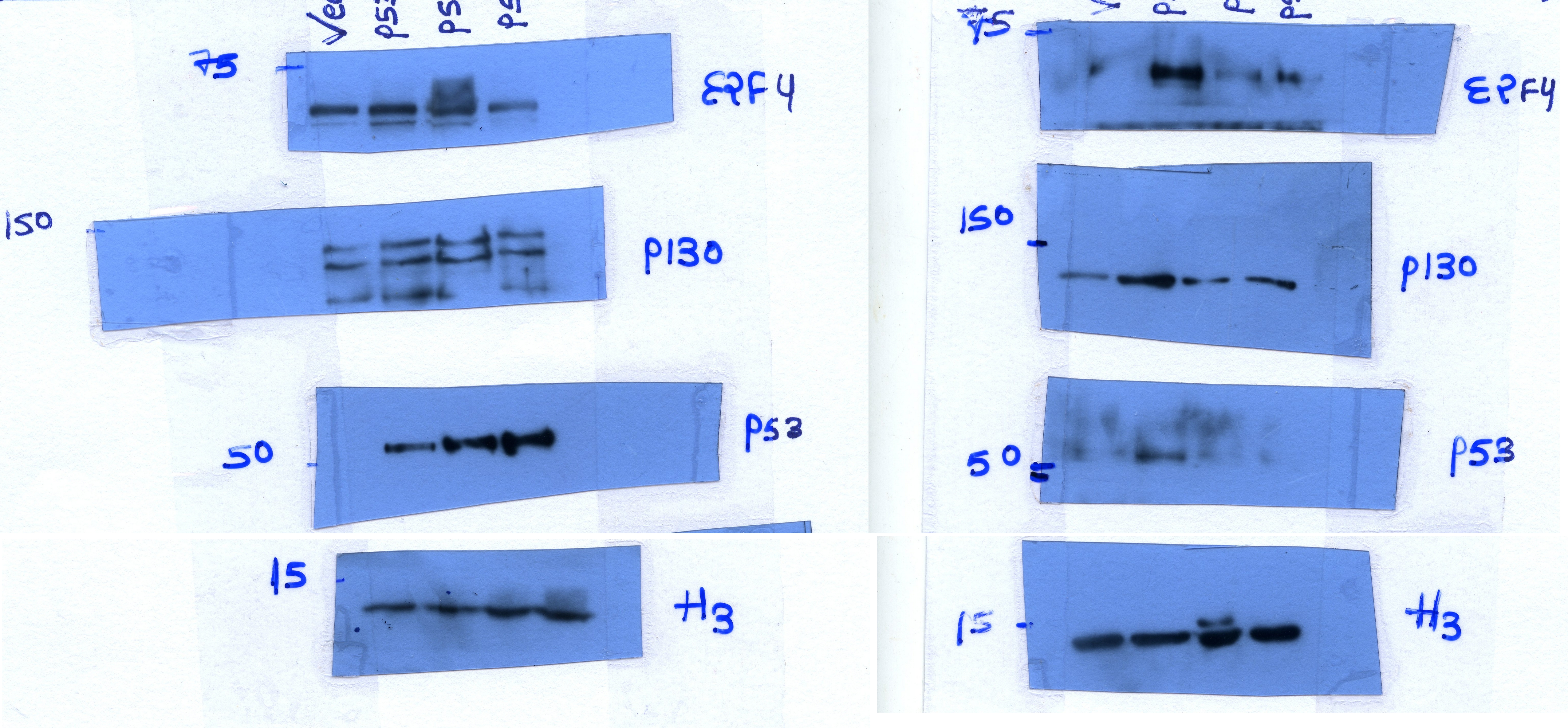

Supplement: Supplementary file 9 — Source data Fig. 4 [file 44318_2025_402_MOESM9_ESM.zip › SD Figure 4/4E/4E Western Replicate#3.jpg]

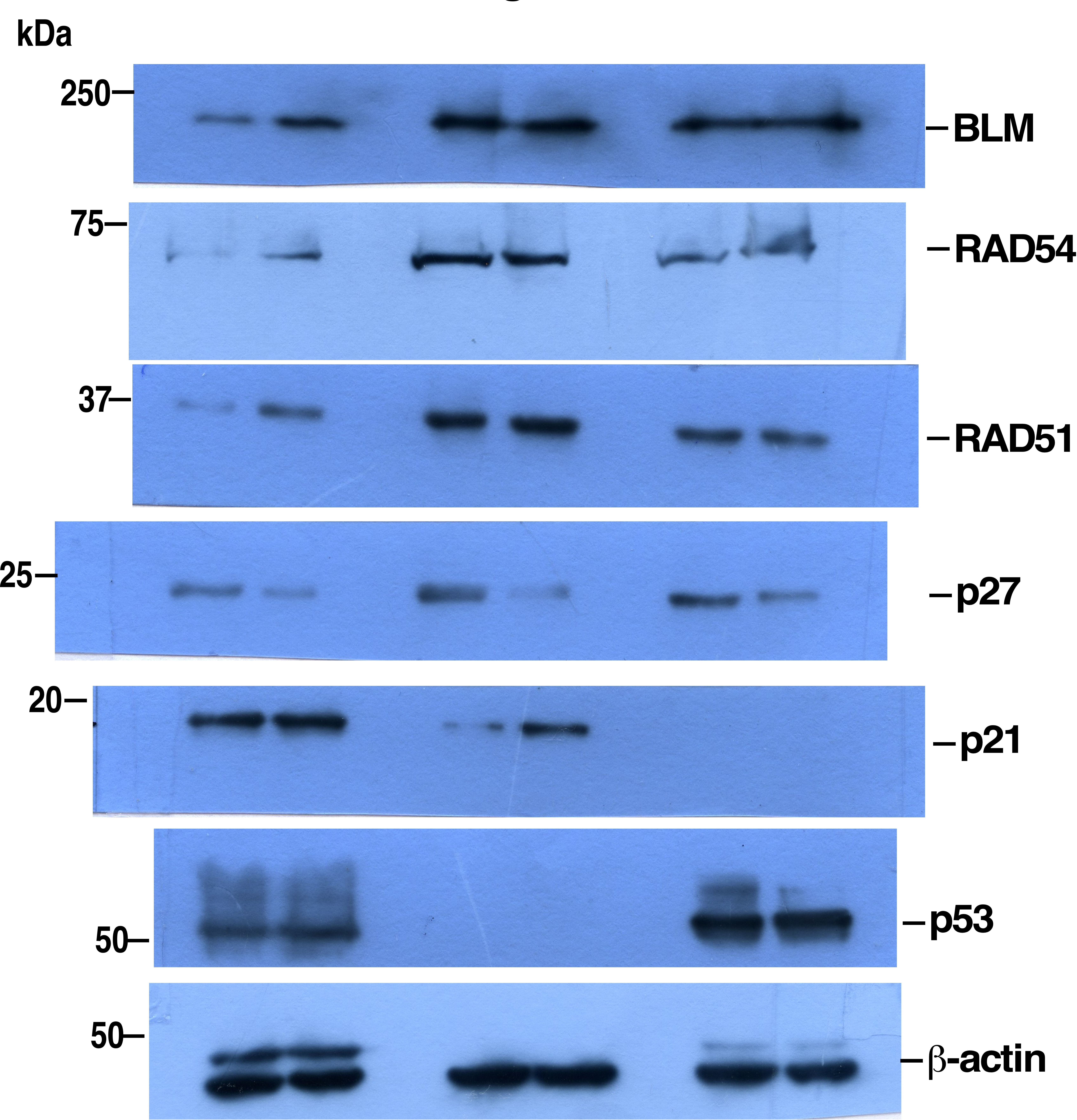

Supplement: Supplementary file 10 — Appendix Figure Source Data [file 44318_2025_402_MOESM10_ESM.zip › SD appendix figure/Figure S1/S1D/S1D Western Replicate#1 (in publication).jpg]

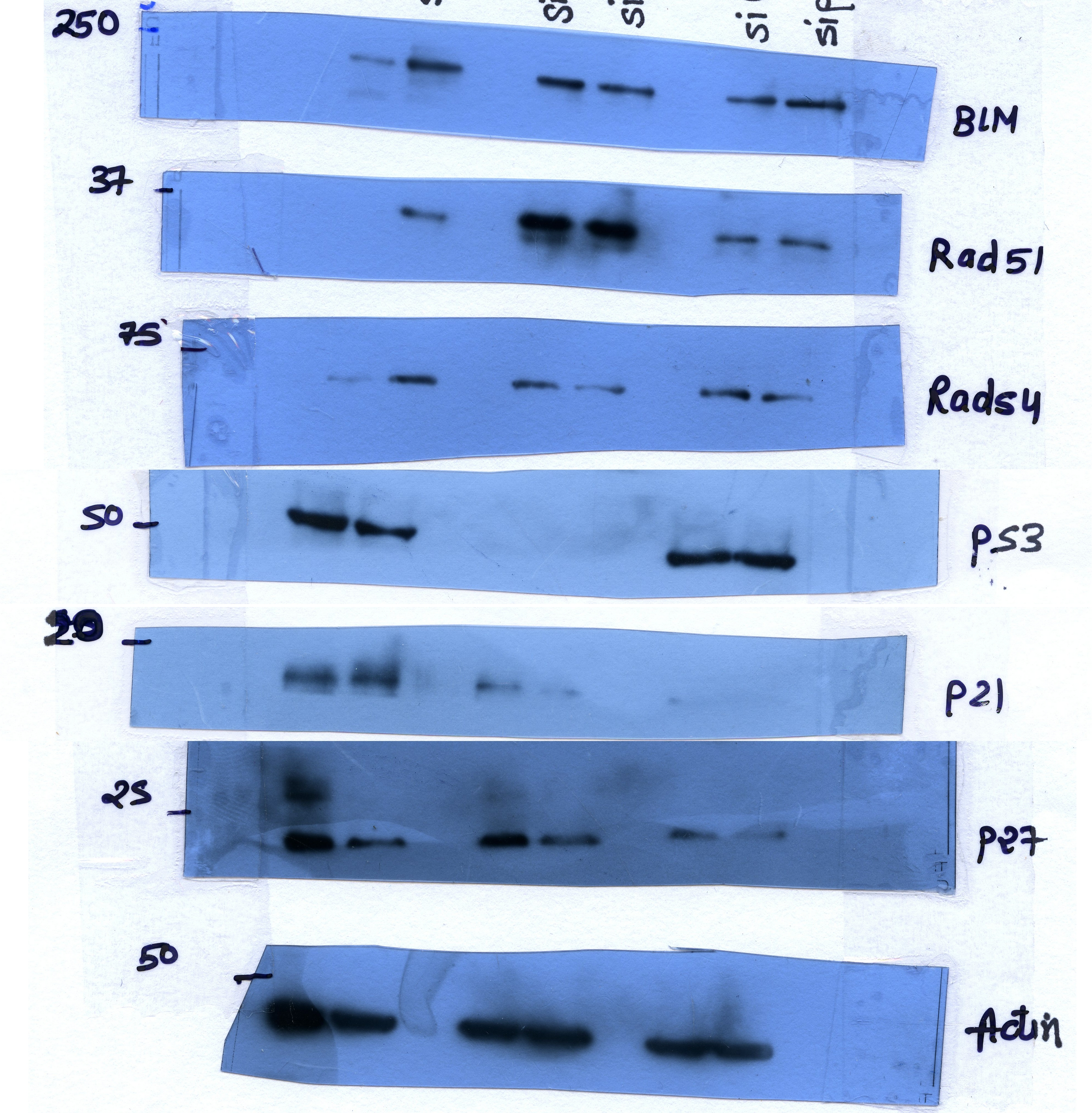

Supplement: Supplementary file 10 — Appendix Figure Source Data [file 44318_2025_402_MOESM10_ESM.zip › SD appendix figure/Figure S1/S1D/S1D Western Replicate#2.jpg]

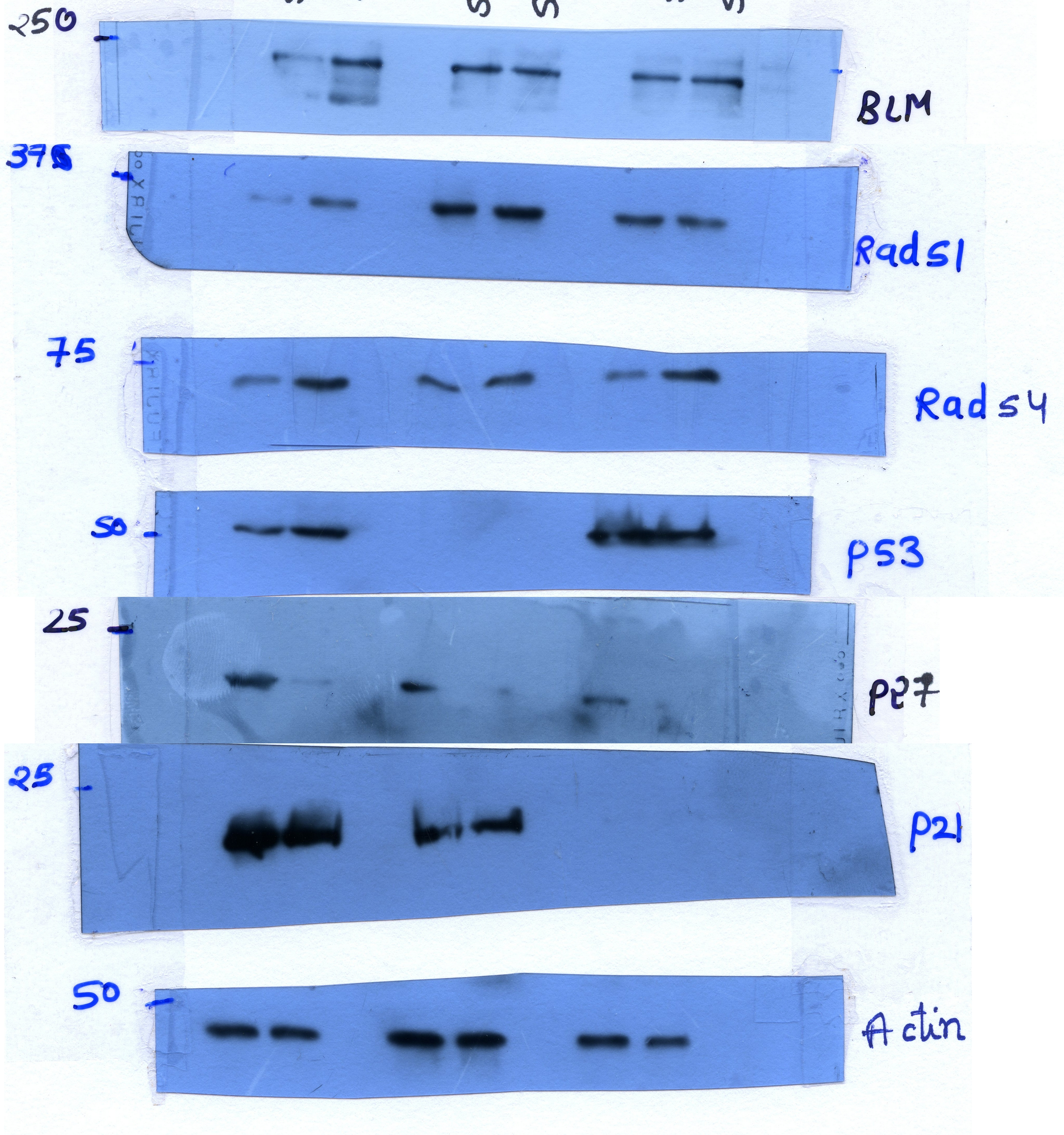

Supplement: Supplementary file 10 — Appendix Figure Source Data [file 44318_2025_402_MOESM10_ESM.zip › SD appendix figure/Figure S1/S1D/S1D Western Replicate#3.jpg]

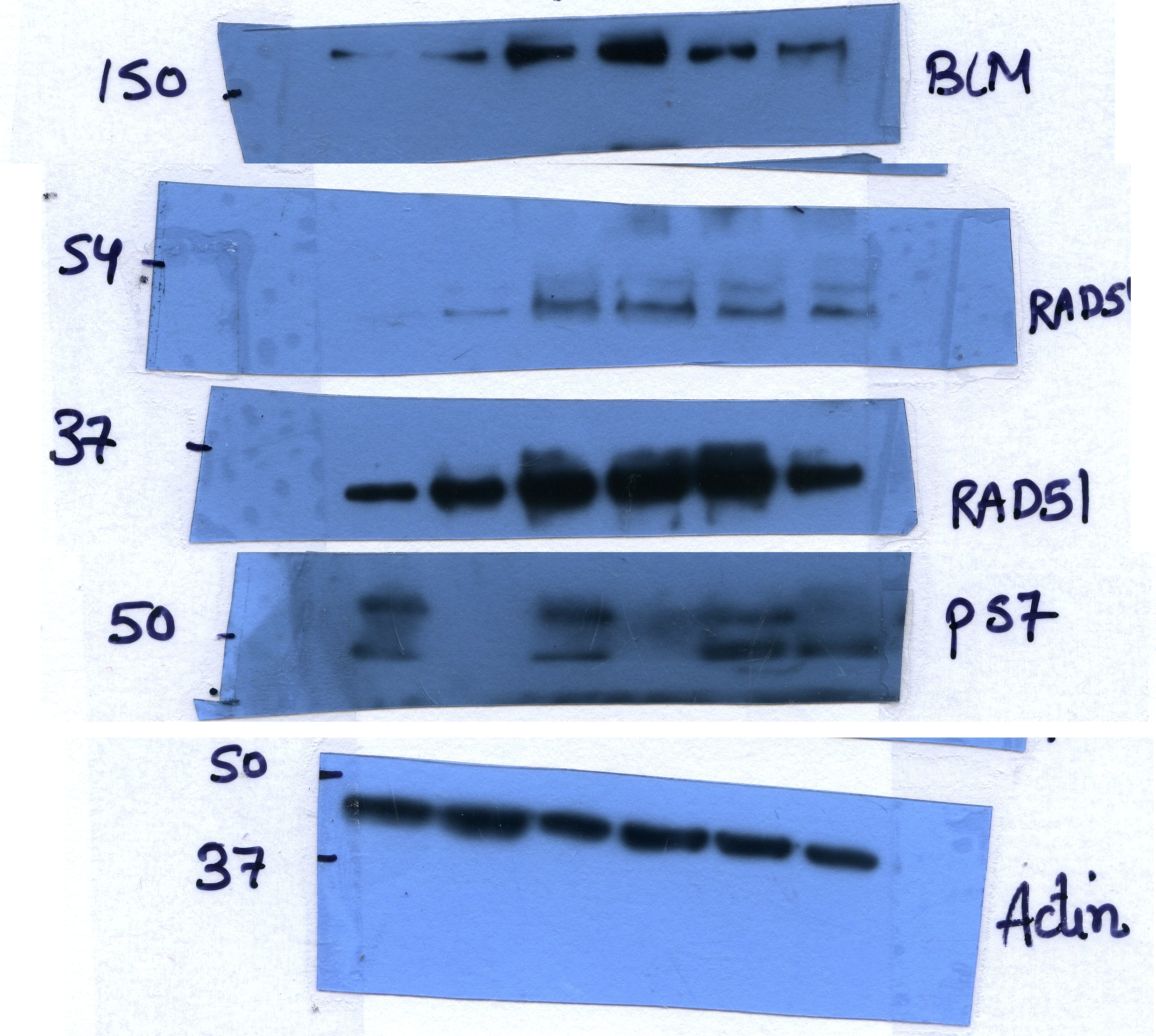

Supplement: Supplementary file 10 — Appendix Figure Source Data [file 44318_2025_402_MOESM10_ESM.zip › SD appendix figure/Figure S1/S1E/S1E Western Replicate#1.jpg]

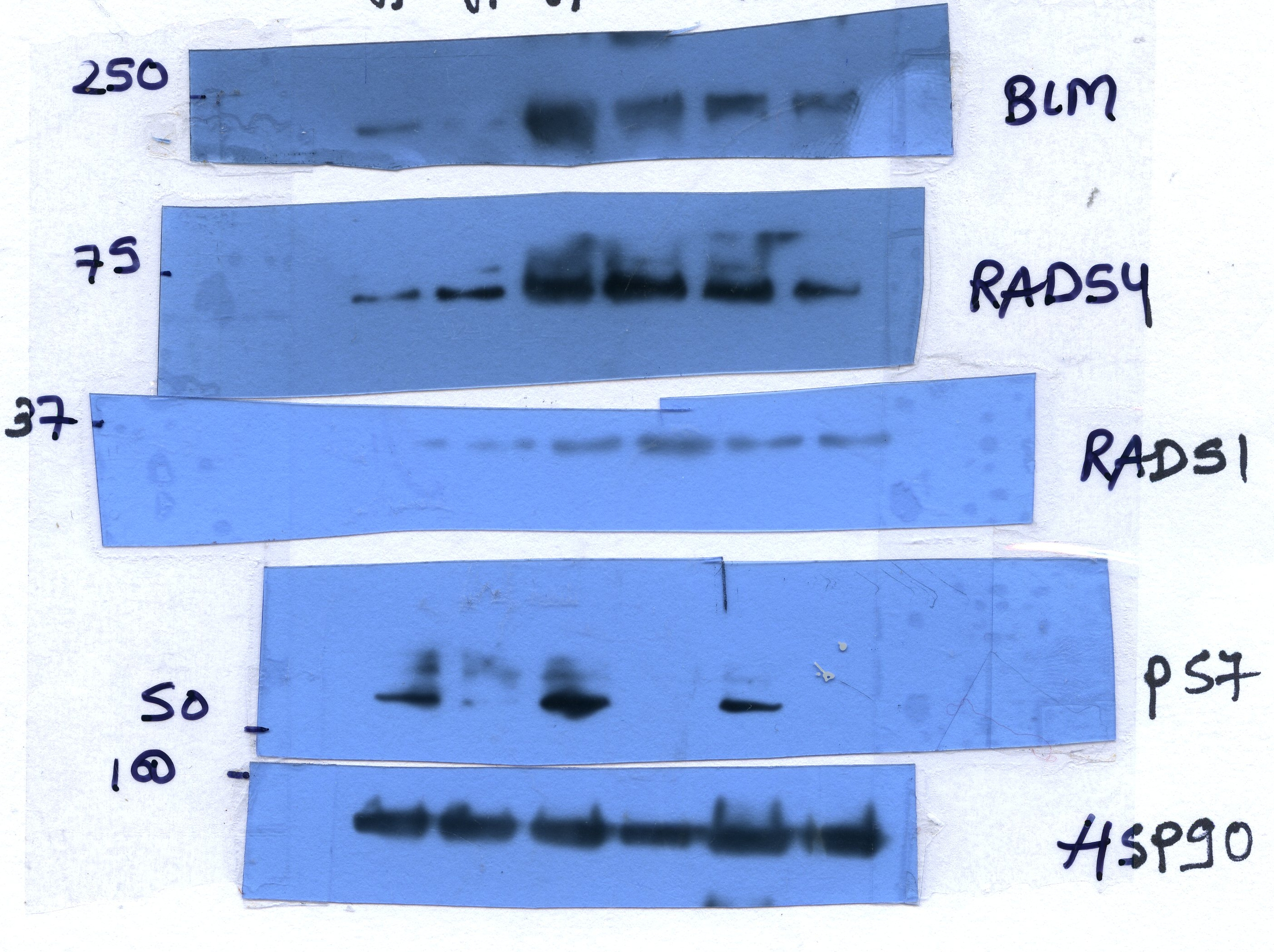

Supplement: Supplementary file 10 — Appendix Figure Source Data [file 44318_2025_402_MOESM10_ESM.zip › SD appendix figure/Figure S1/S1E/S1E Western Replicate#2.jpg]

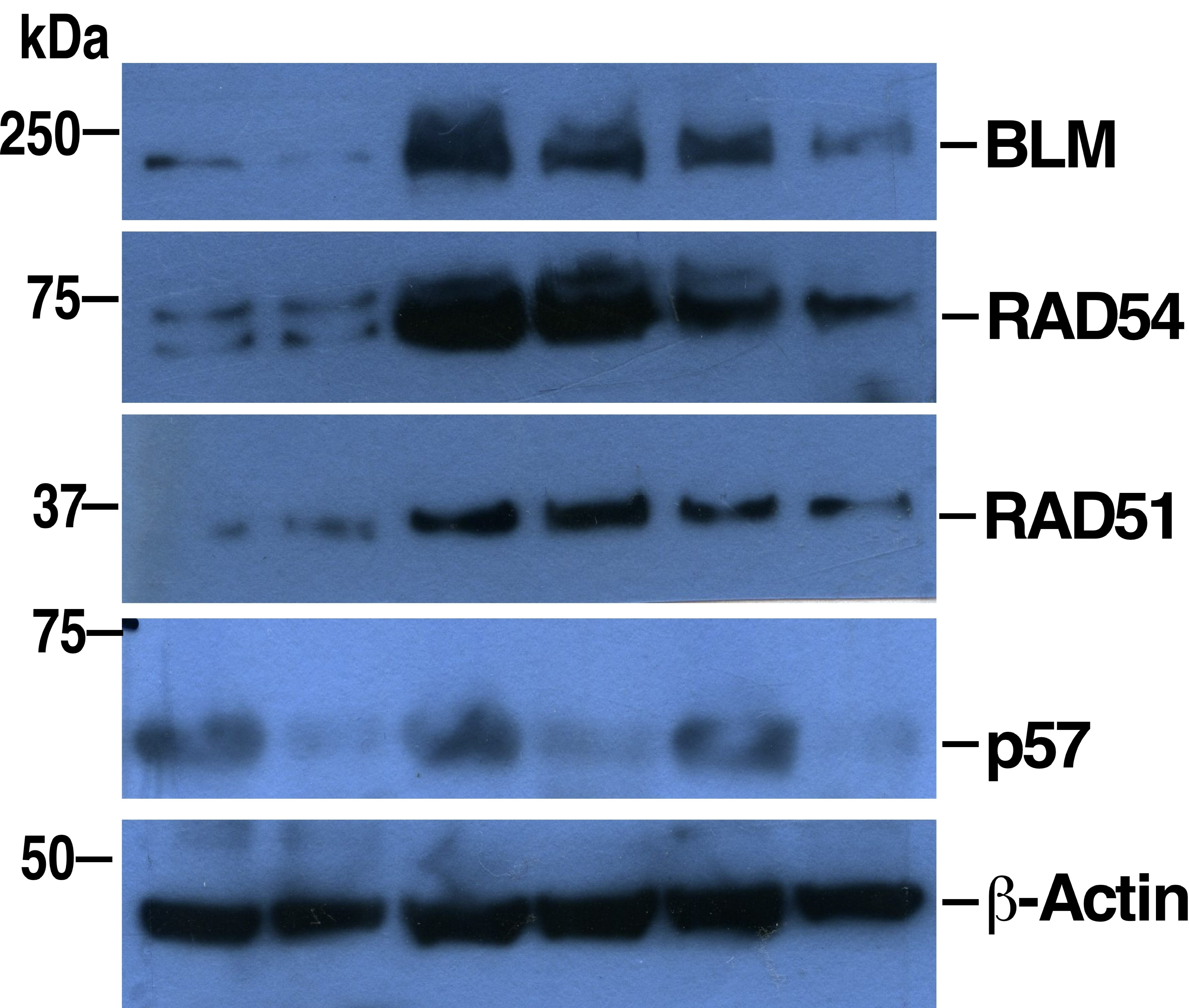

Supplement: Supplementary file 10 — Appendix Figure Source Data [file 44318_2025_402_MOESM10_ESM.zip › SD appendix figure/Figure S1/S1E/S1E Western Replicate#3 (in publication).jpg]

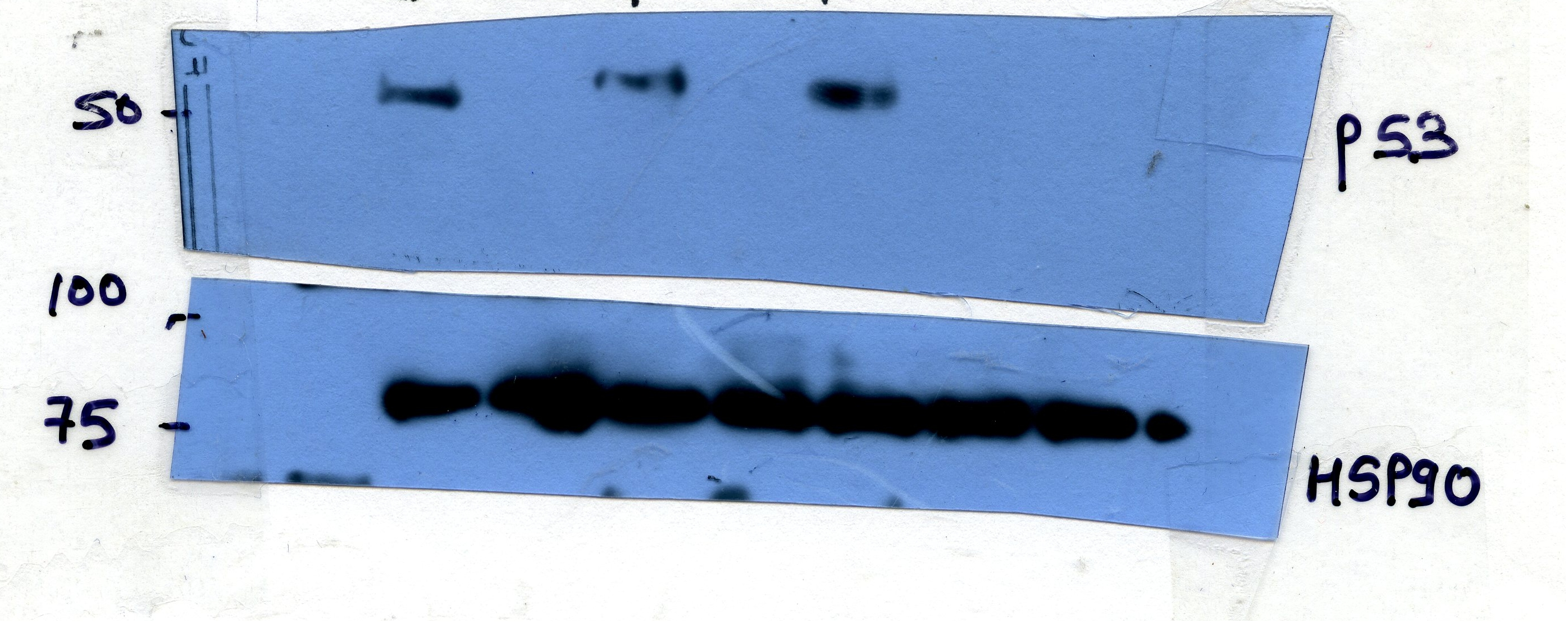

Supplement: Supplementary file 10 — Appendix Figure Source Data [file 44318_2025_402_MOESM10_ESM.zip › SD appendix figure/Figure S1/S1G/S1G Western Replicate#1.jpg]

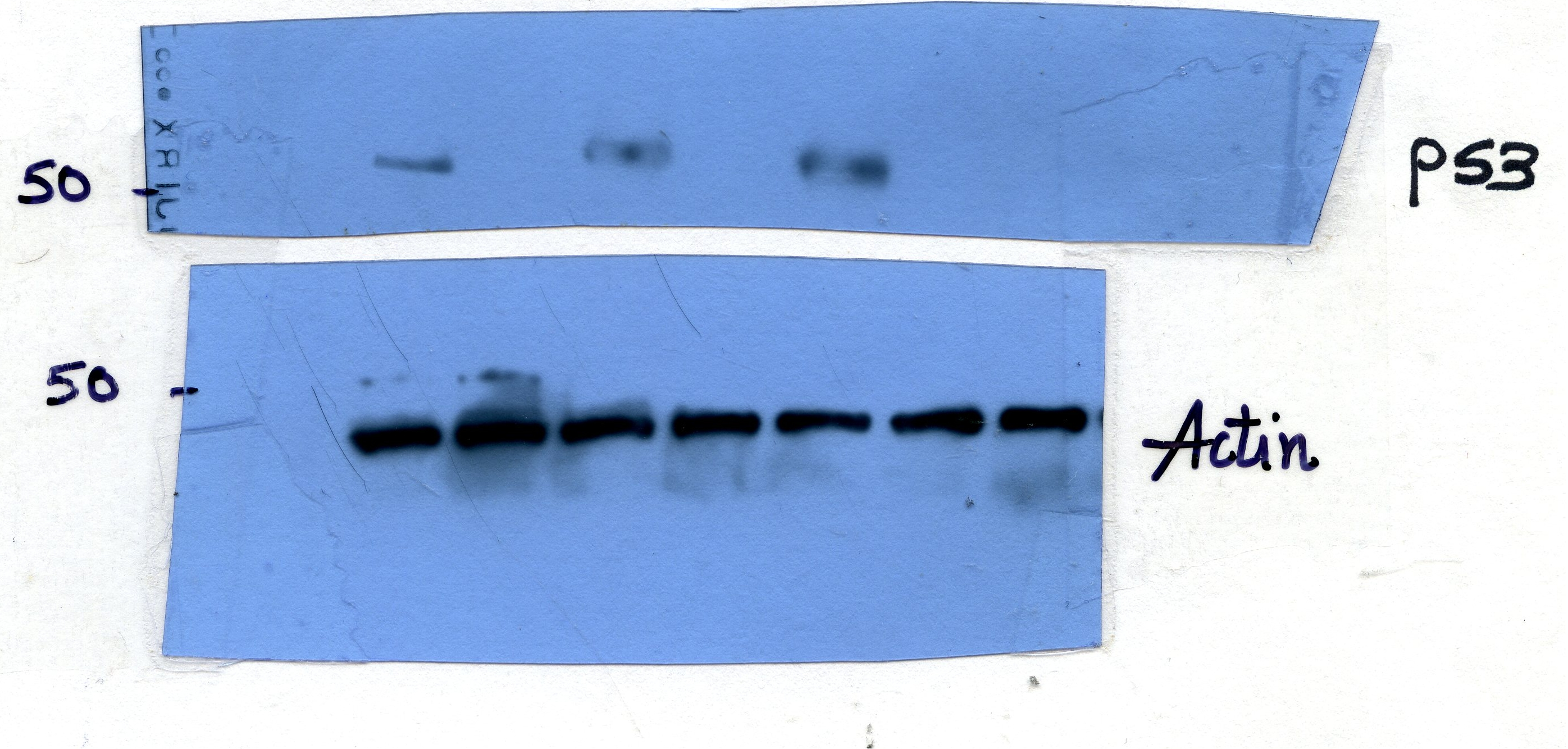

Supplement: Supplementary file 10 — Appendix Figure Source Data [file 44318_2025_402_MOESM10_ESM.zip › SD appendix figure/Figure S1/S1G/S1G Western Replicate#2.jpg]

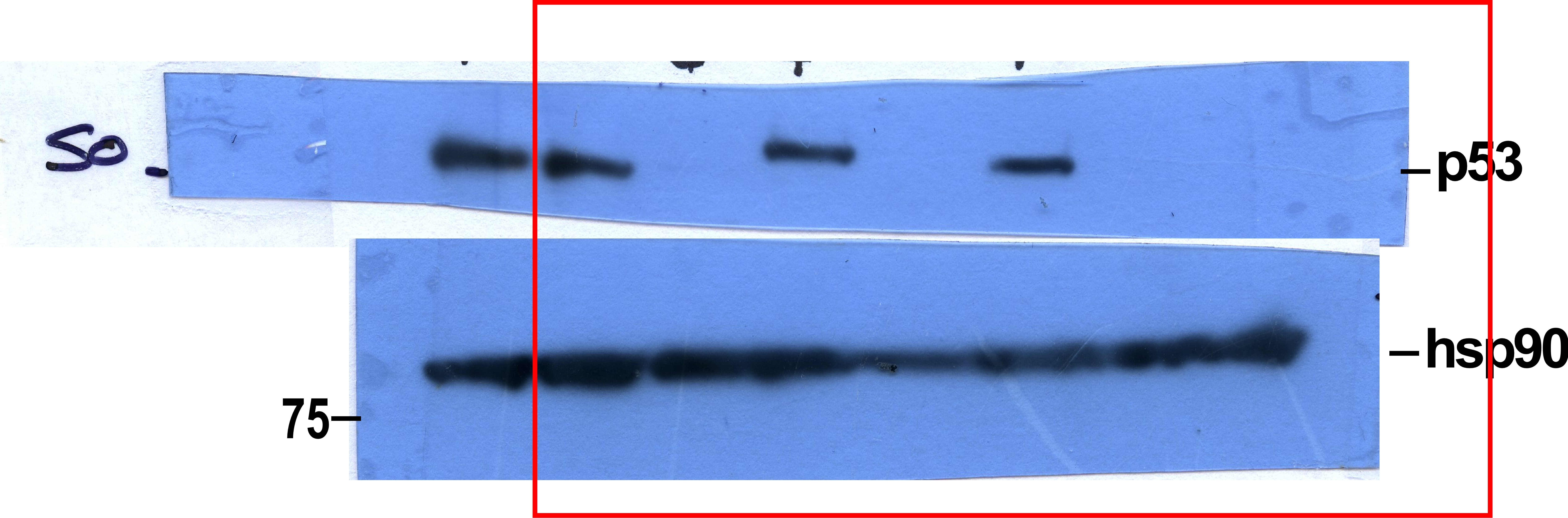

Supplement: Supplementary file 10 — Appendix Figure Source Data [file 44318_2025_402_MOESM10_ESM.zip › SD appendix figure/Figure S1/S1G/S1G Western Replicate#3 (in publication).jpg]

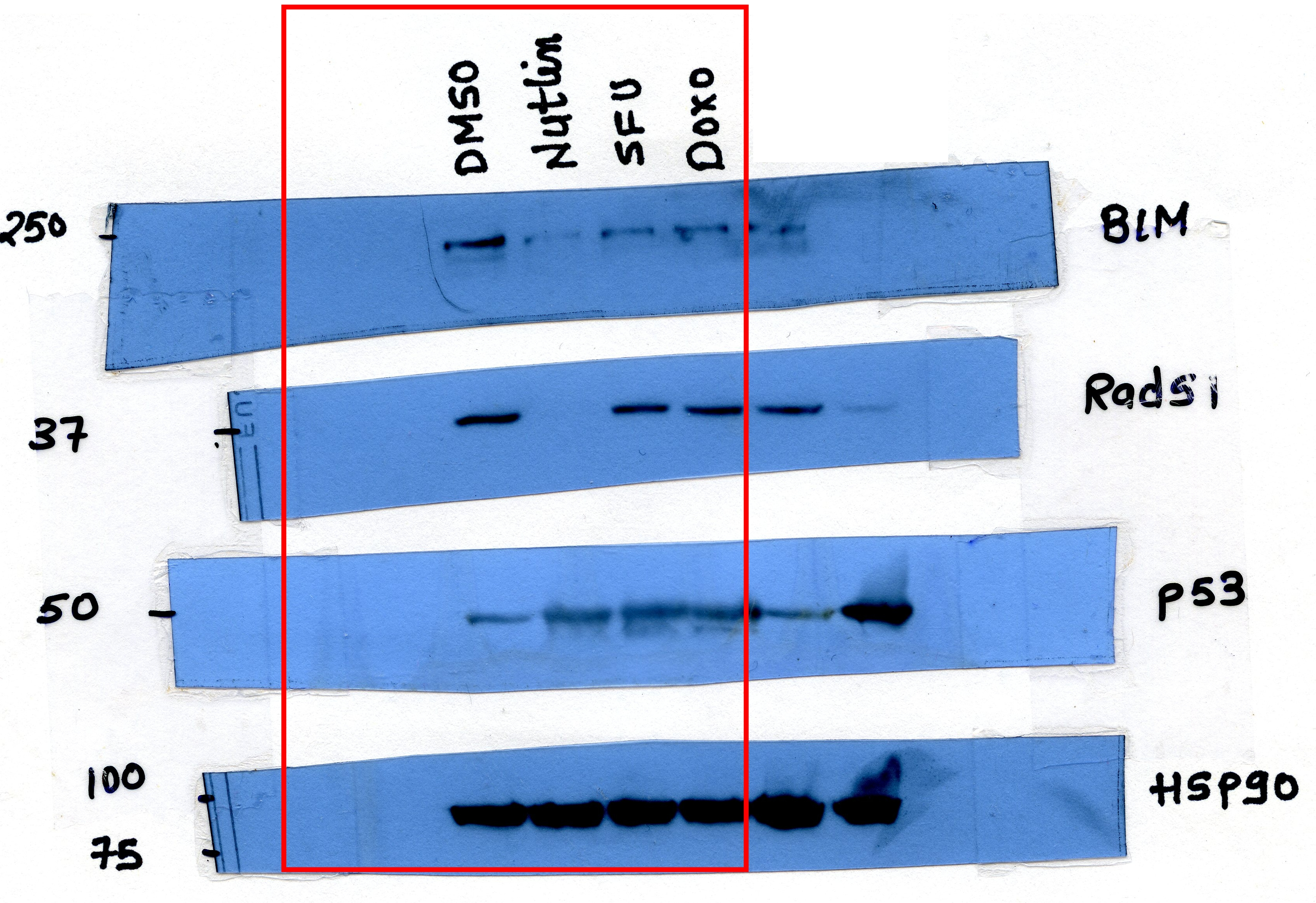

Supplement: Supplementary file 10 — Appendix Figure Source Data [file 44318_2025_402_MOESM10_ESM.zip › SD appendix figure/Figure S2/S2A/S2A Western Replicate#1.jpg]

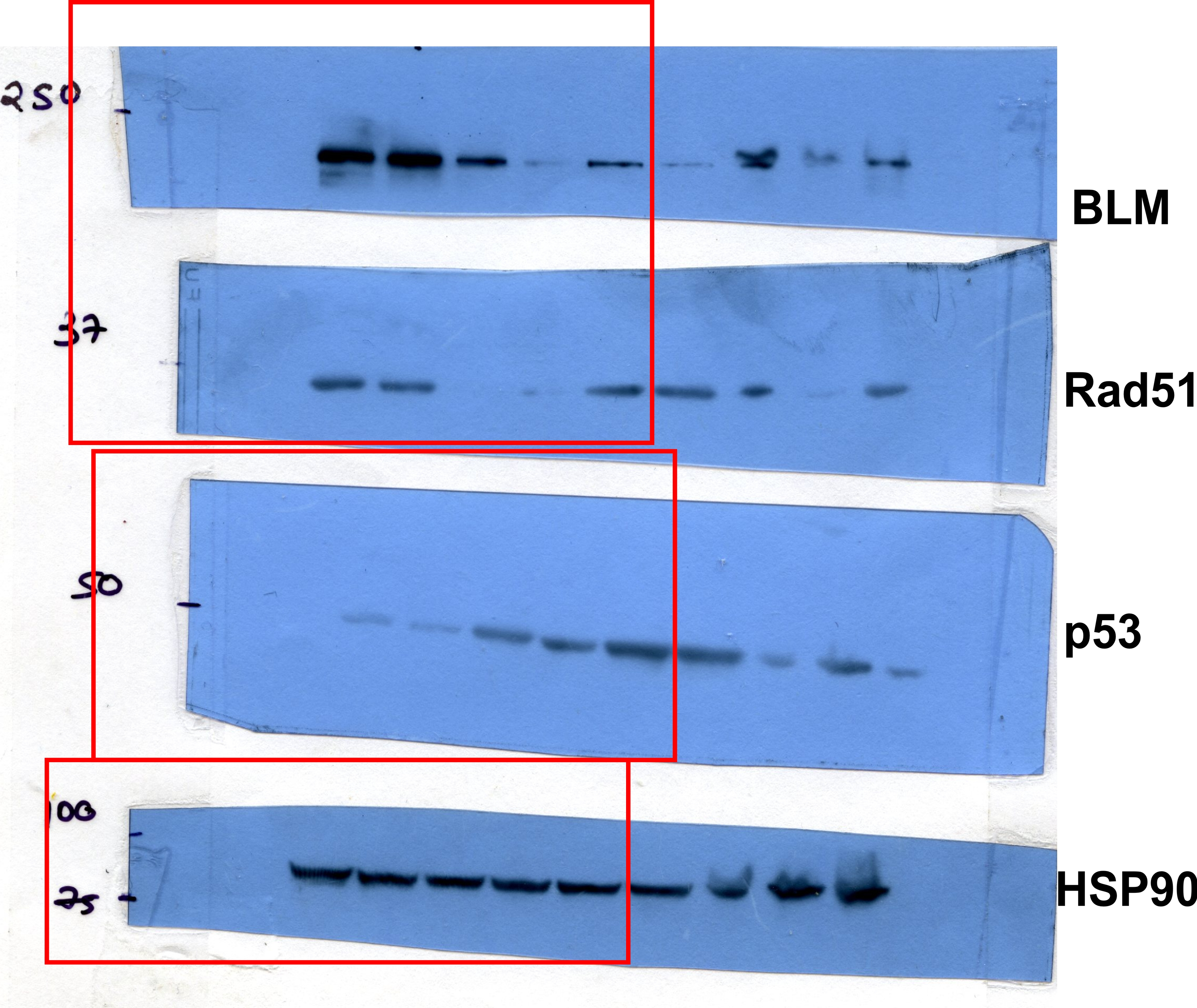

Supplement: Supplementary file 10 — Appendix Figure Source Data [file 44318_2025_402_MOESM10_ESM.zip › SD appendix figure/Figure S2/S2A/S2A Western Replicate#2 (in publication).jpg]

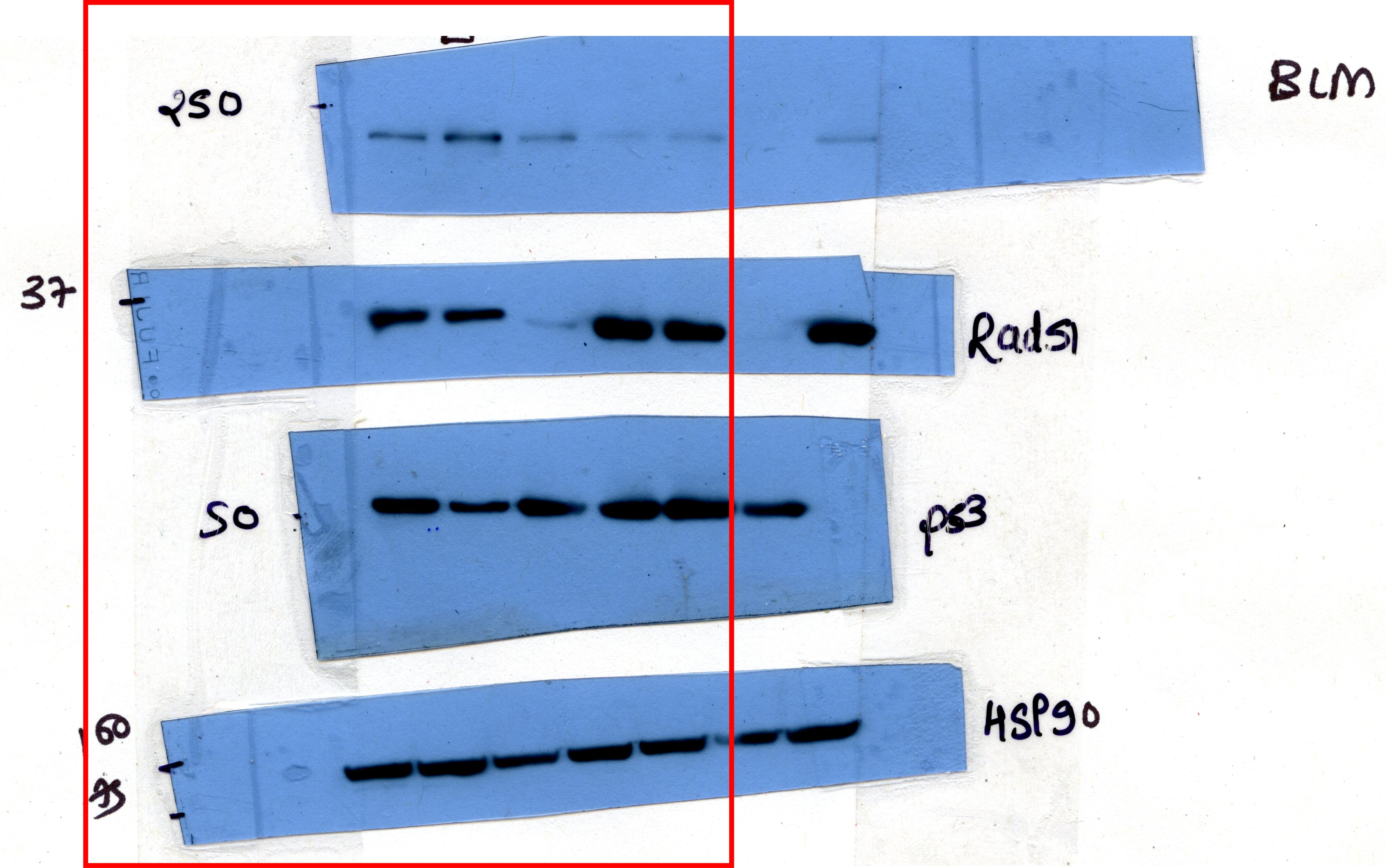

Supplement: Supplementary file 10 — Appendix Figure Source Data [file 44318_2025_402_MOESM10_ESM.zip › SD appendix figure/Figure S2/S2A/S2A Western Replicate#3.jpg]

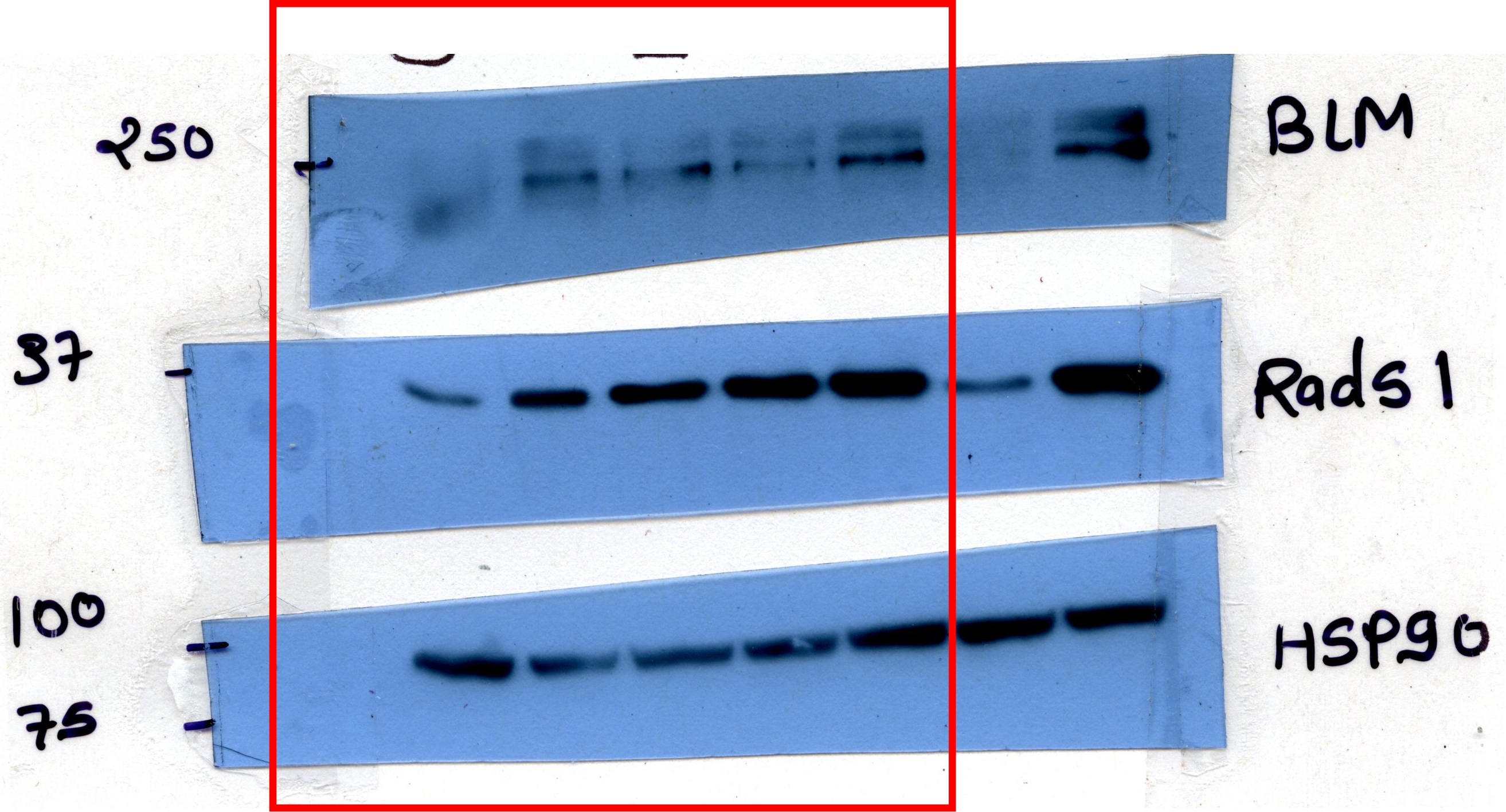

Supplement: Supplementary file 10 — Appendix Figure Source Data [file 44318_2025_402_MOESM10_ESM.zip › SD appendix figure/Figure S2/S2B/S2B Western Replicate#1.jpg]

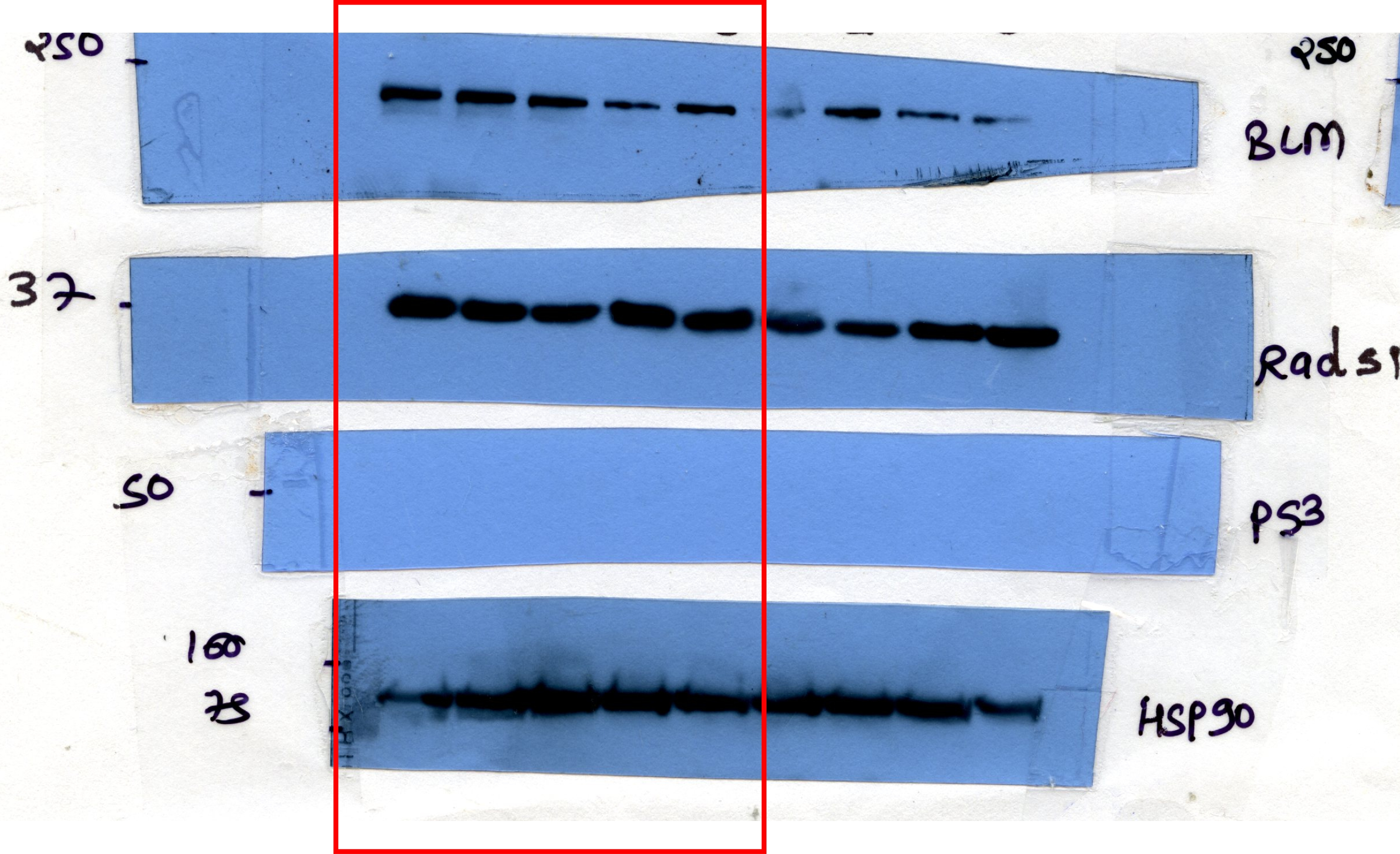

Supplement: Supplementary file 10 — Appendix Figure Source Data [file 44318_2025_402_MOESM10_ESM.zip › SD appendix figure/Figure S2/S2B/S2B Western Replicate#2.jpg]

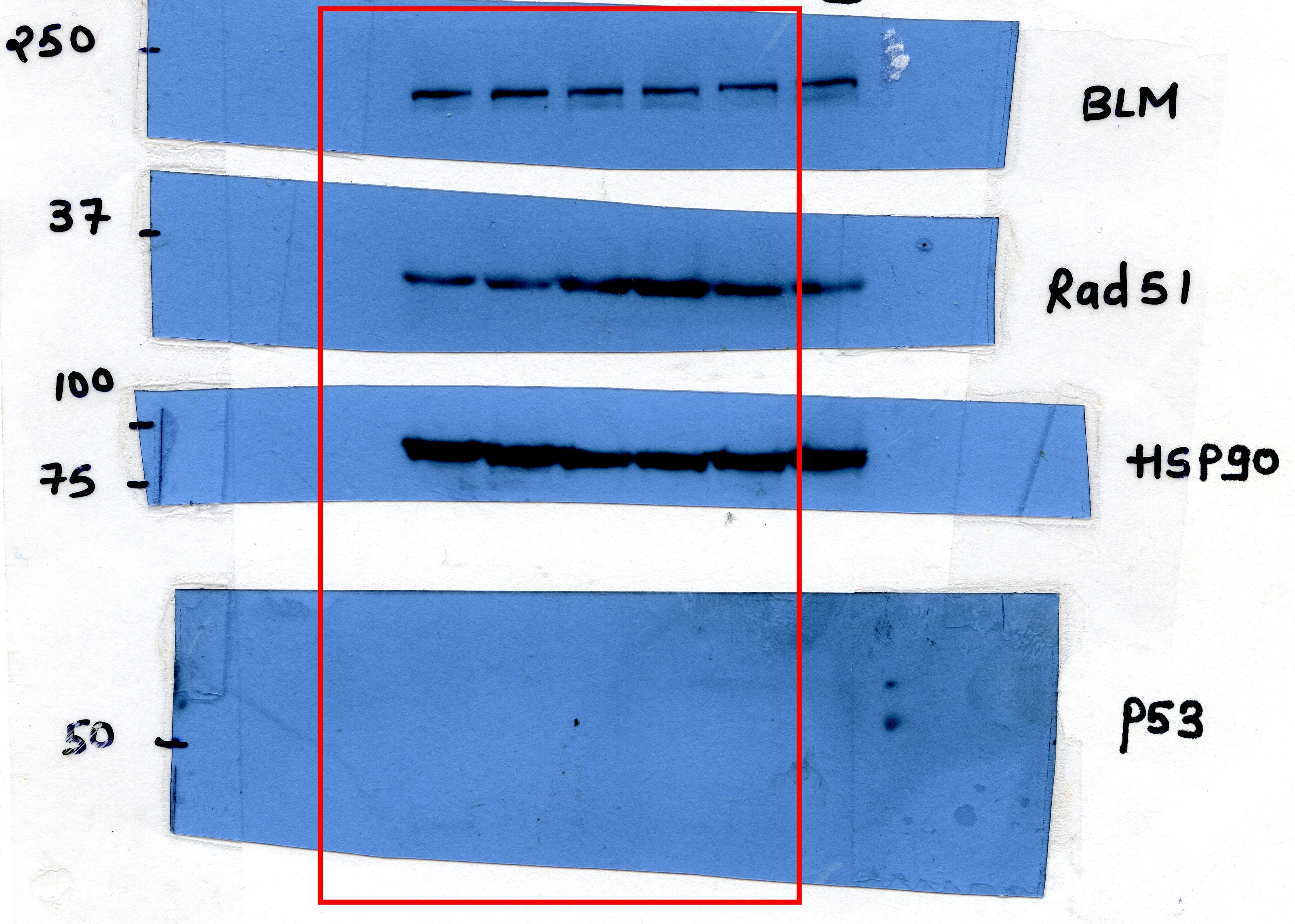

Supplement: Supplementary file 10 — Appendix Figure Source Data [file 44318_2025_402_MOESM10_ESM.zip › SD appendix figure/Figure S2/S2B/S2B Western Replicate#3 (in publication).jpg]

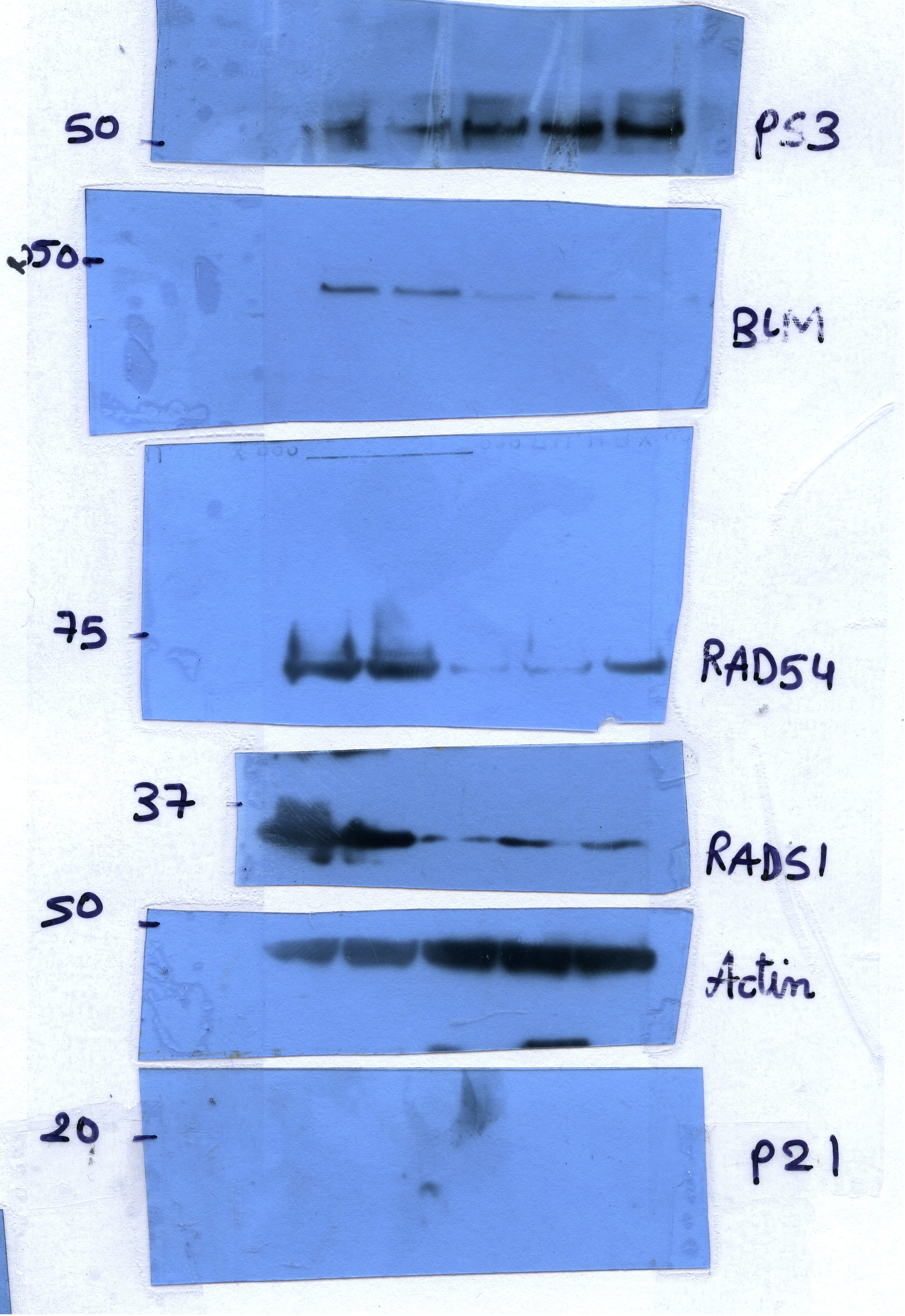

Supplement: Supplementary file 10 — Appendix Figure Source Data [file 44318_2025_402_MOESM10_ESM.zip › SD appendix figure/Figure S2/S2C/S2C Western Replicate#2.jpg]

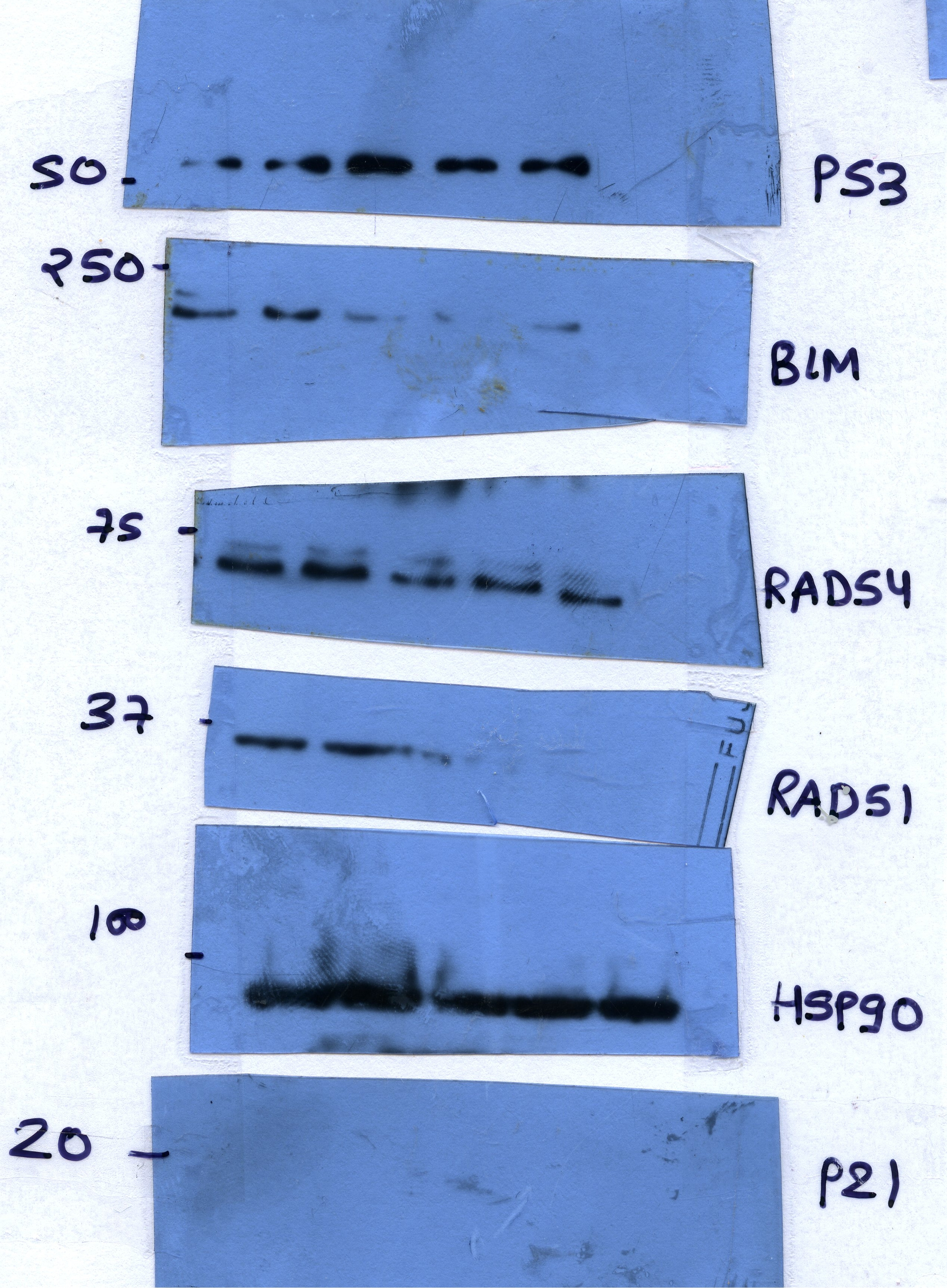

Supplement: Supplementary file 10 — Appendix Figure Source Data [file 44318_2025_402_MOESM10_ESM.zip › SD appendix figure/Figure S2/S2C/S2C Western Replicate#3.jpg]

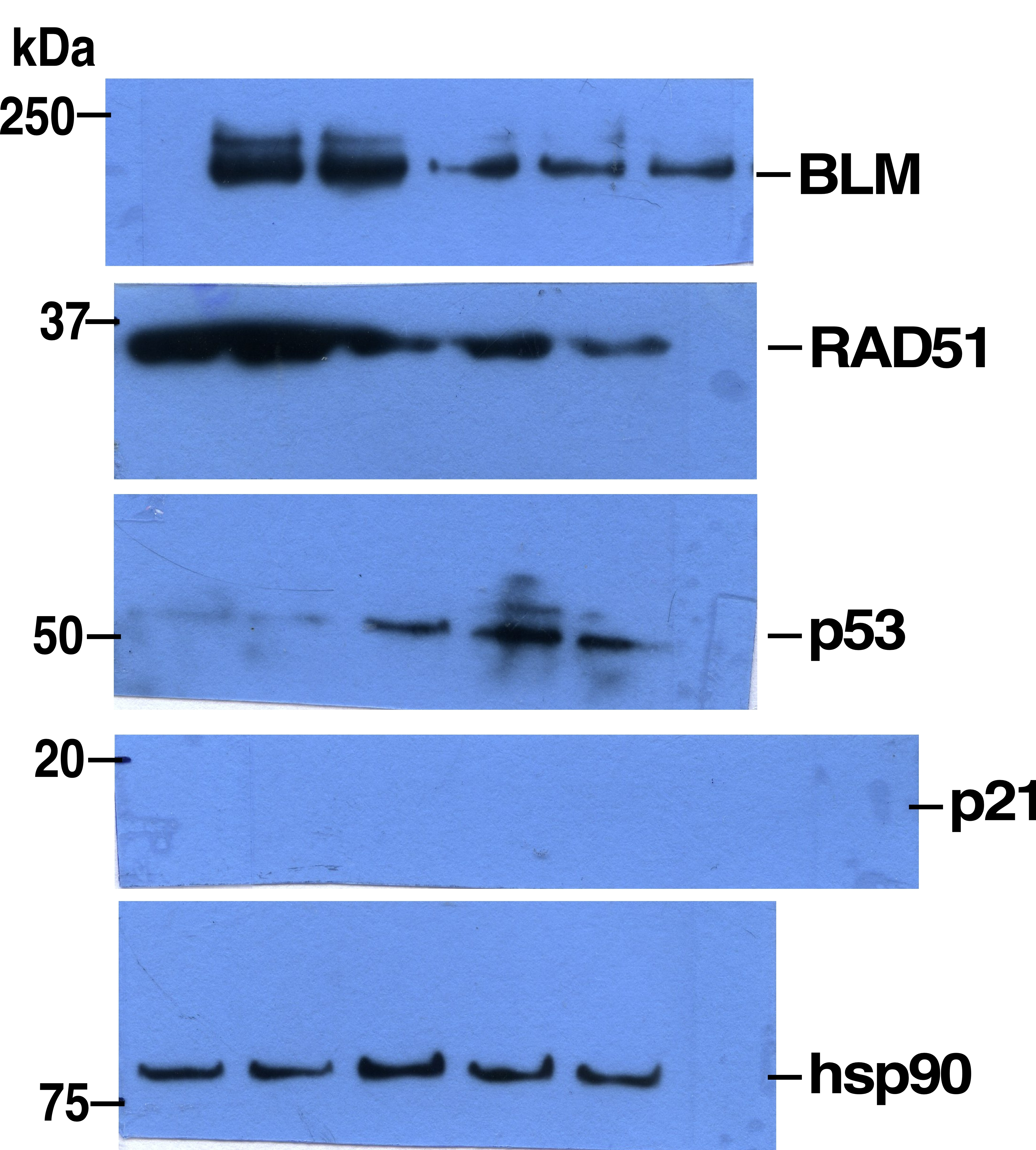

Supplement: Supplementary file 10 — Appendix Figure Source Data [file 44318_2025_402_MOESM10_ESM.zip › SD appendix figure/Figure S2/S2C/S2C Western Replicate#31 (in publication).jpg]

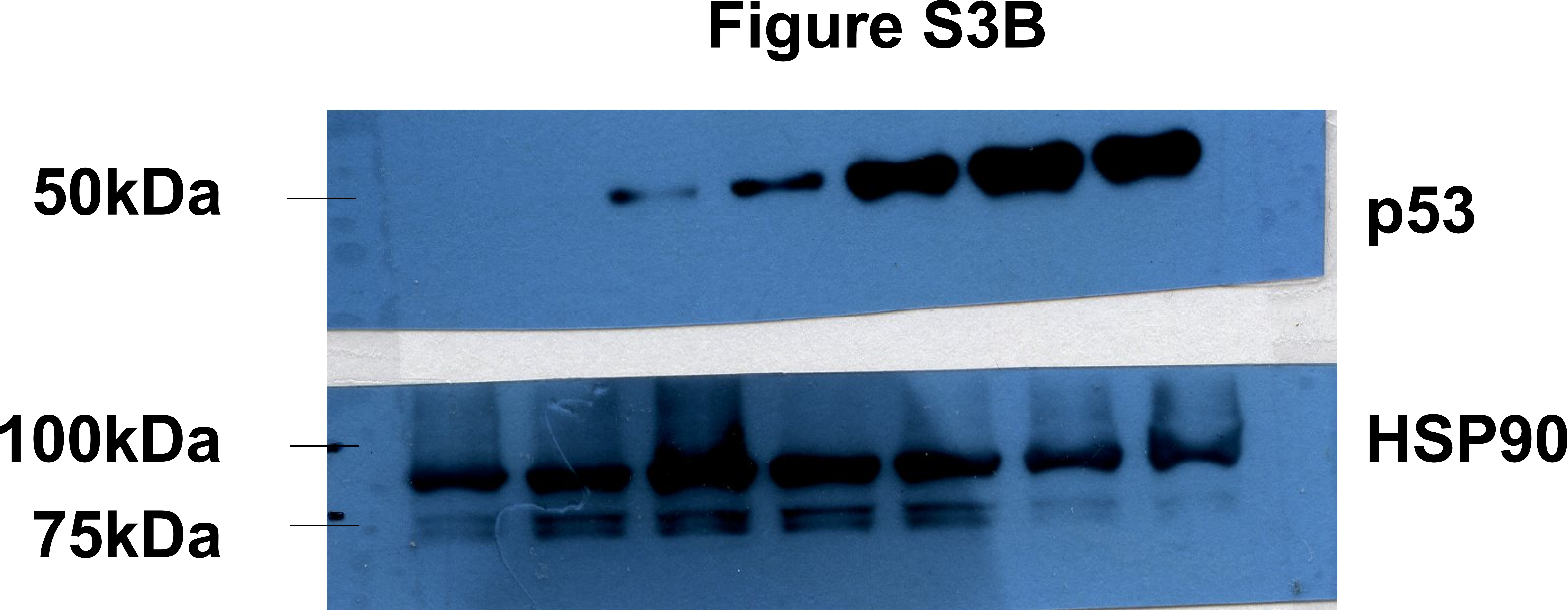

Supplement: Supplementary file 10 — Appendix Figure Source Data [file 44318_2025_402_MOESM10_ESM.zip › SD appendix figure/Figure S3/S3B/S3B Western Replicate#1 (in publication).jpg]

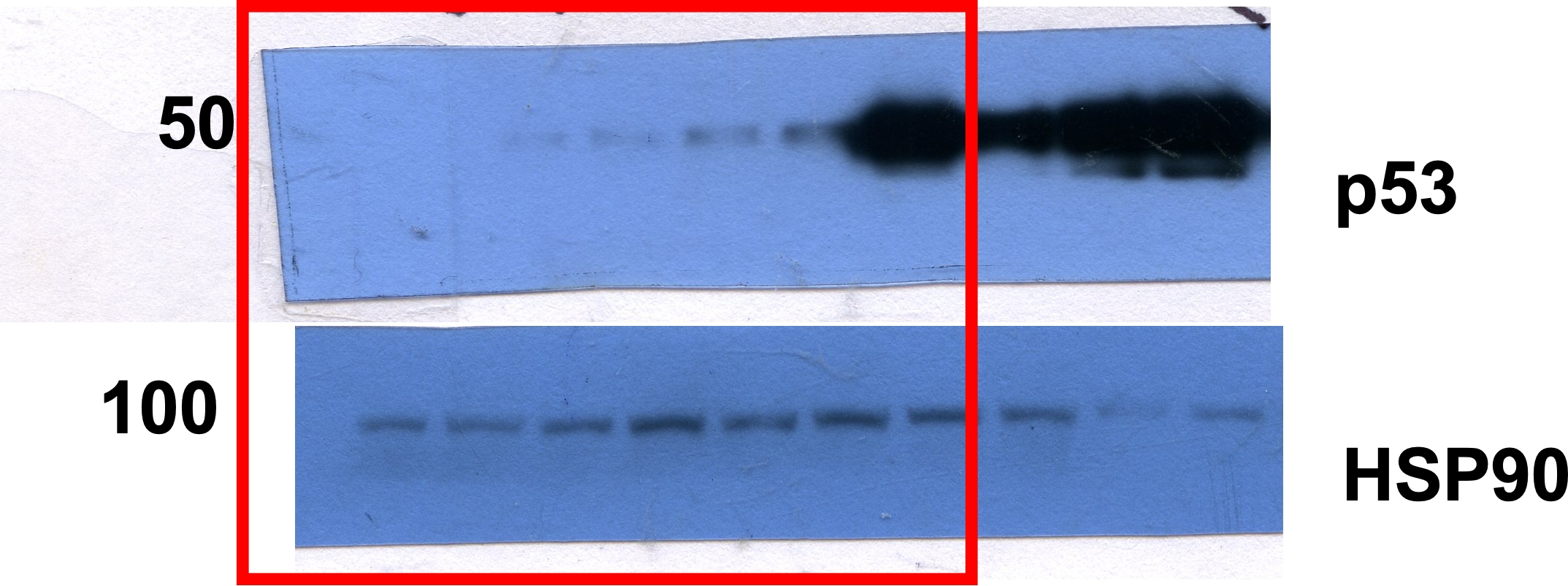

Supplement: Supplementary file 10 — Appendix Figure Source Data [file 44318_2025_402_MOESM10_ESM.zip › SD appendix figure/Figure S3/S3B/S3B Western Replicate#2.jpg]

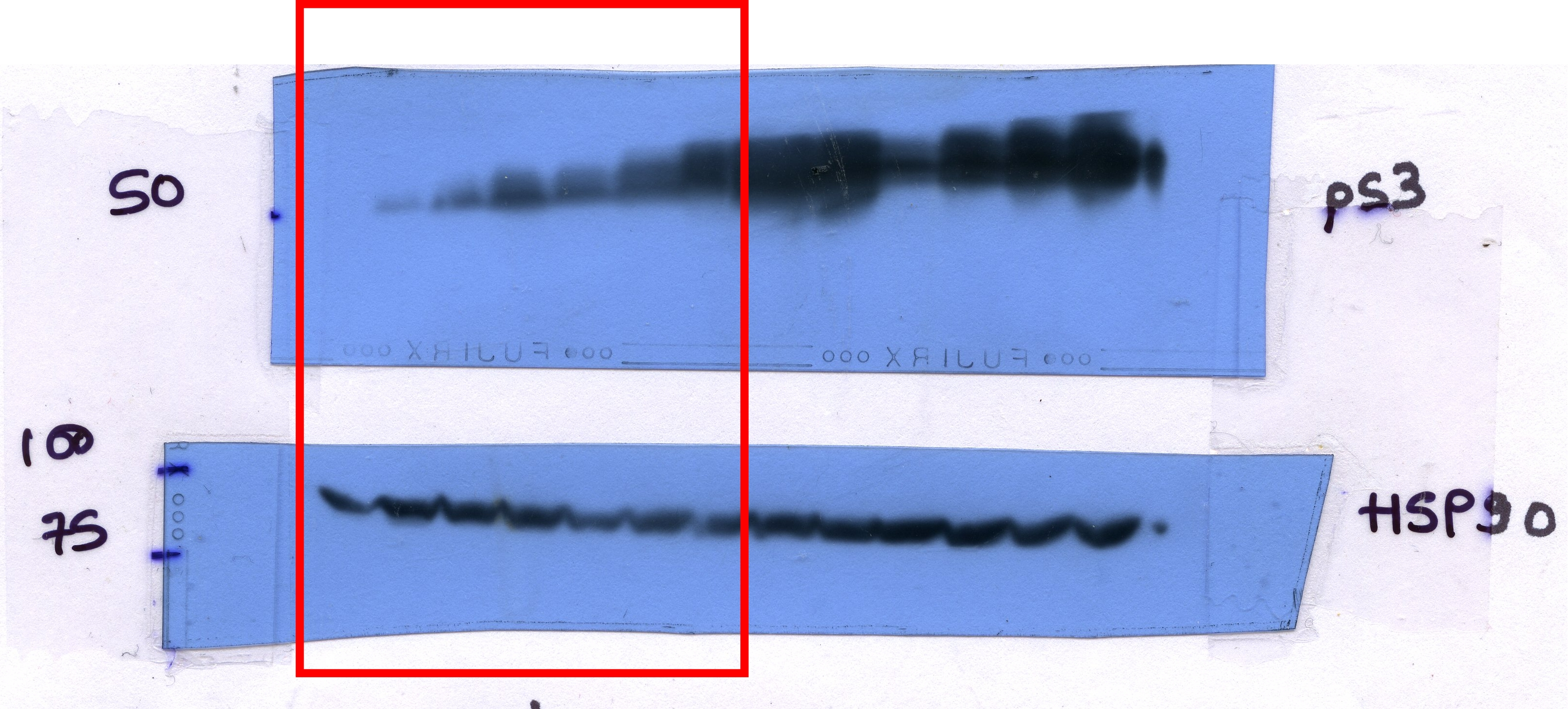

Supplement: Supplementary file 10 — Appendix Figure Source Data [file 44318_2025_402_MOESM10_ESM.zip › SD appendix figure/Figure S3/S3B/S3B Western Replicate#3.jpg]

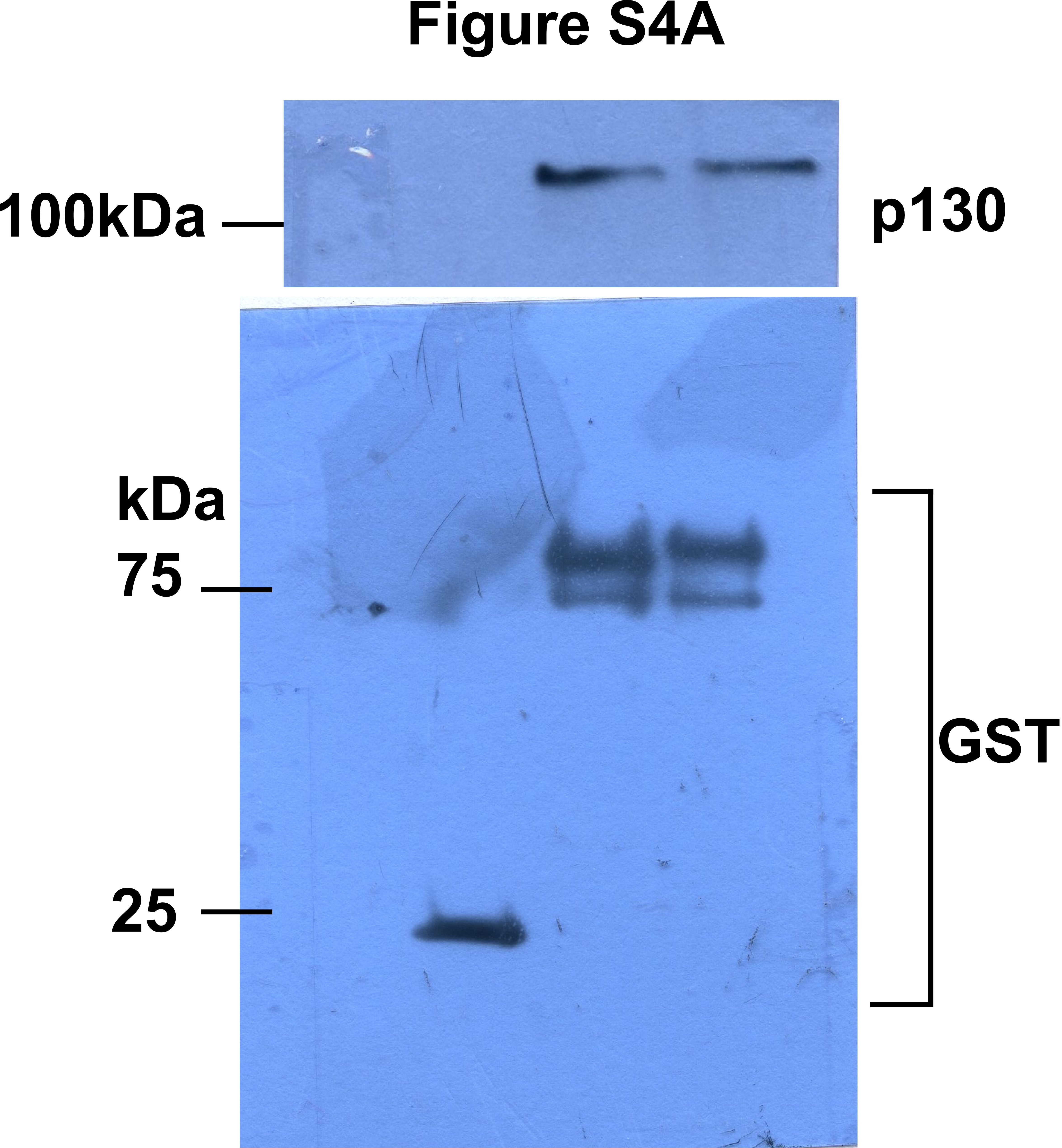

Supplement: Supplementary file 10 — Appendix Figure Source Data [file 44318_2025_402_MOESM10_ESM.zip › SD appendix figure/Figure S4/S4A/S4A Western Replicate#1 (in publication).jpg]

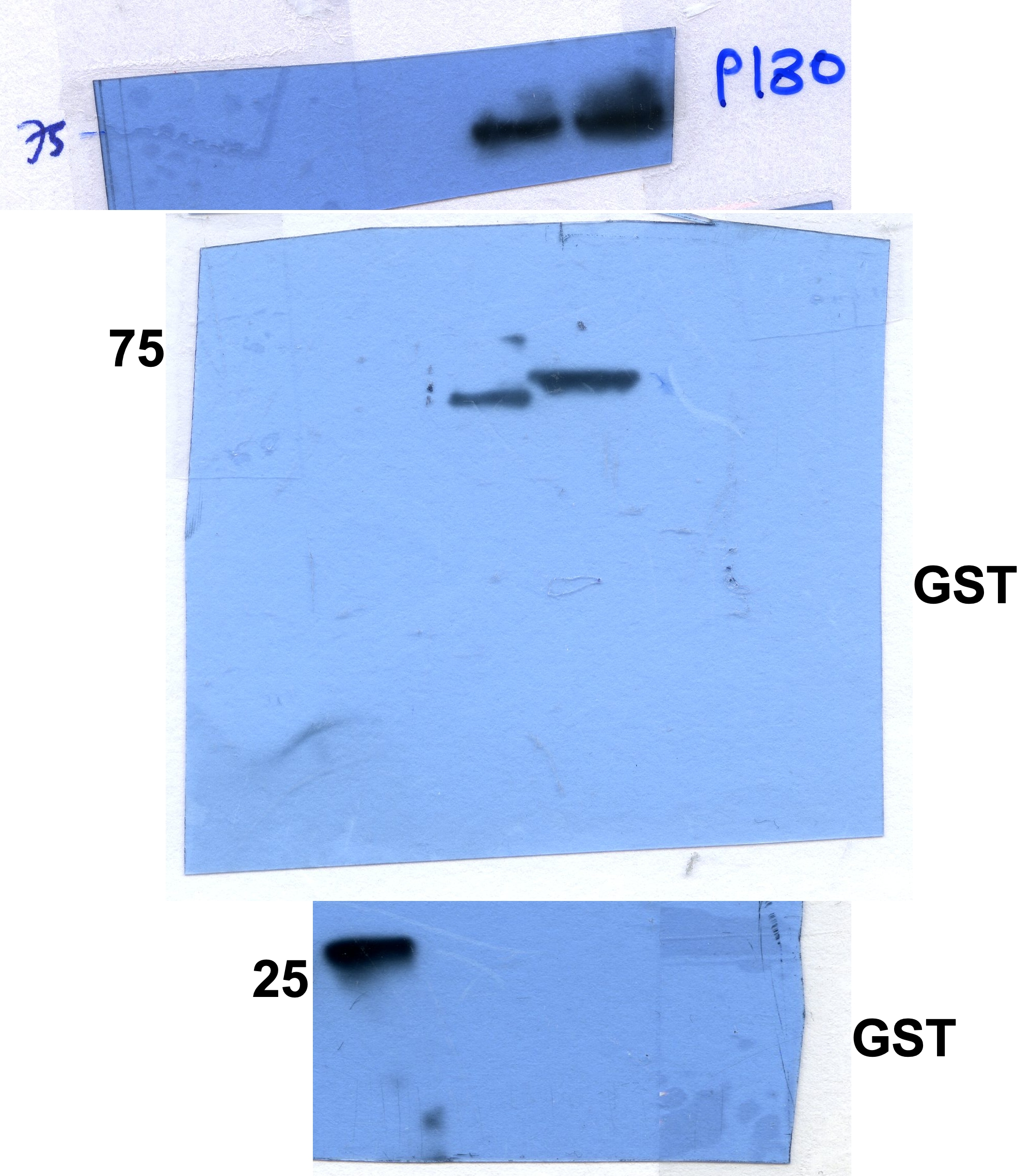

Supplement: Supplementary file 10 — Appendix Figure Source Data [file 44318_2025_402_MOESM10_ESM.zip › SD appendix figure/Figure S4/S4A/S4A Western Replicate#2.jpg]

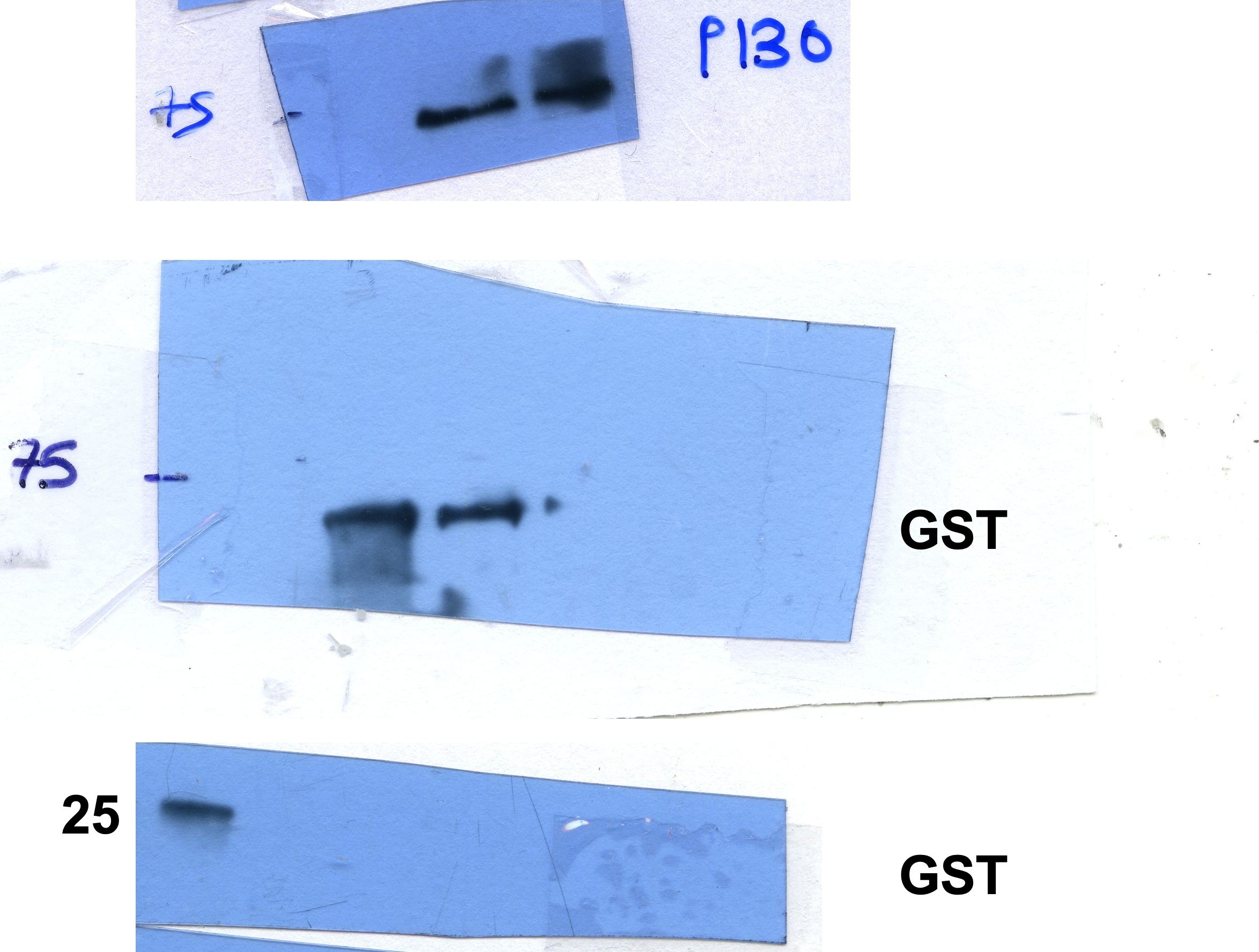

Supplement: Supplementary file 10 — Appendix Figure Source Data [file 44318_2025_402_MOESM10_ESM.zip › SD appendix figure/Figure S4/S4A/S4A Western Replicate#3.jpg]

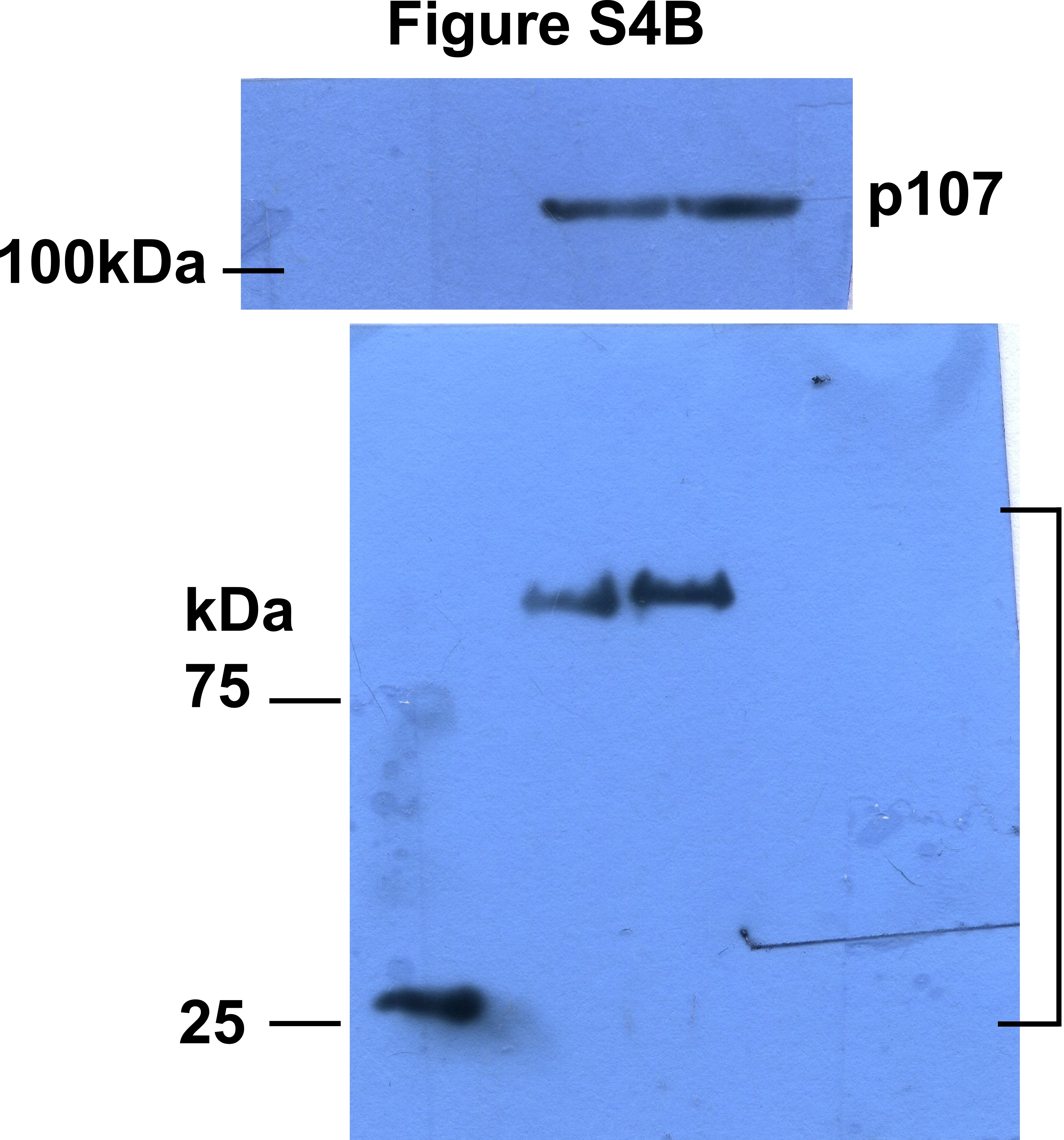

Supplement: Supplementary file 10 — Appendix Figure Source Data [file 44318_2025_402_MOESM10_ESM.zip › SD appendix figure/Figure S4/S4B/S4B Western Replicate#1 (in publication).jpg]

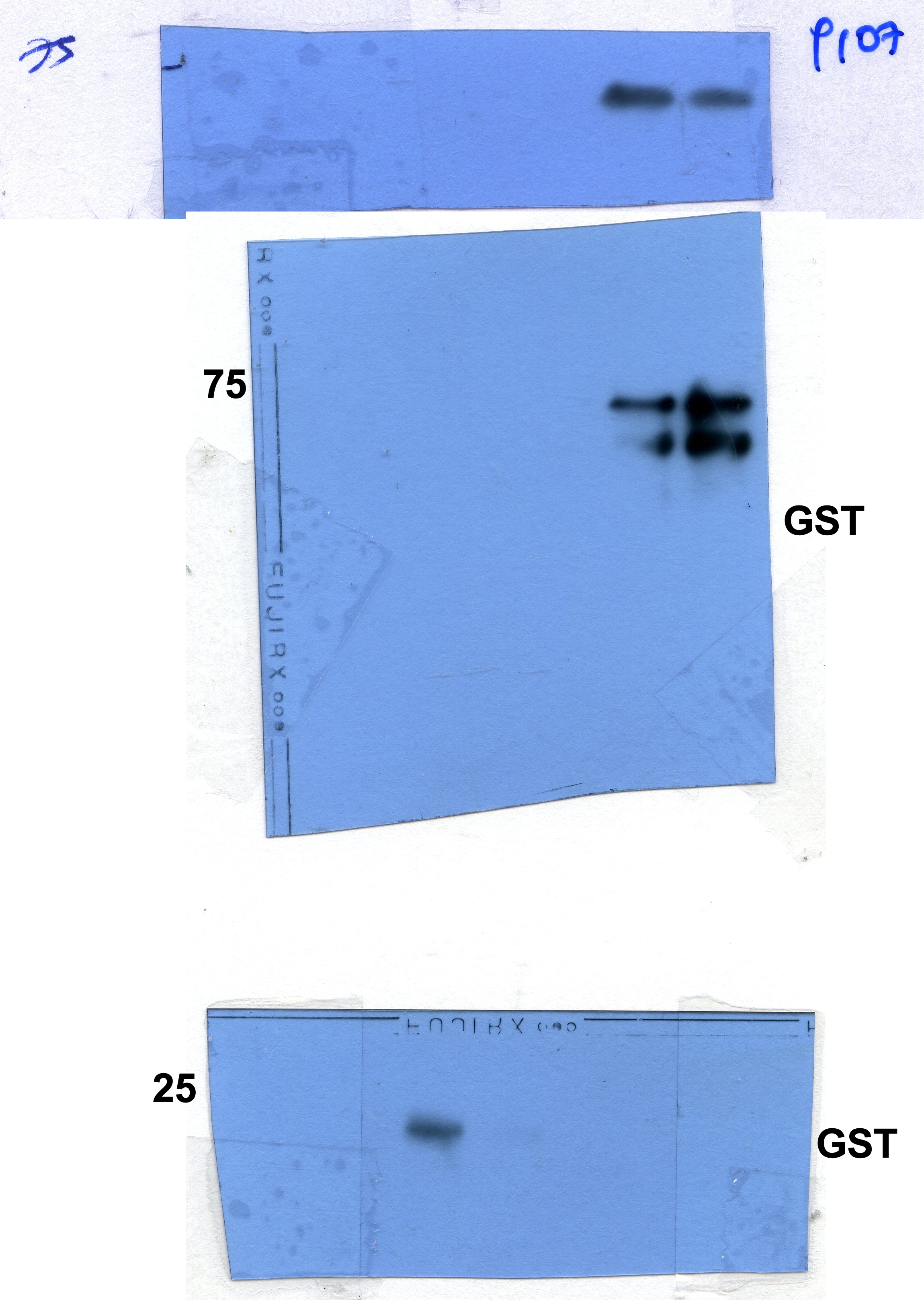

Supplement: Supplementary file 10 — Appendix Figure Source Data [file 44318_2025_402_MOESM10_ESM.zip › SD appendix figure/Figure S4/S4B/S4B Western Replicate#2.jpg]

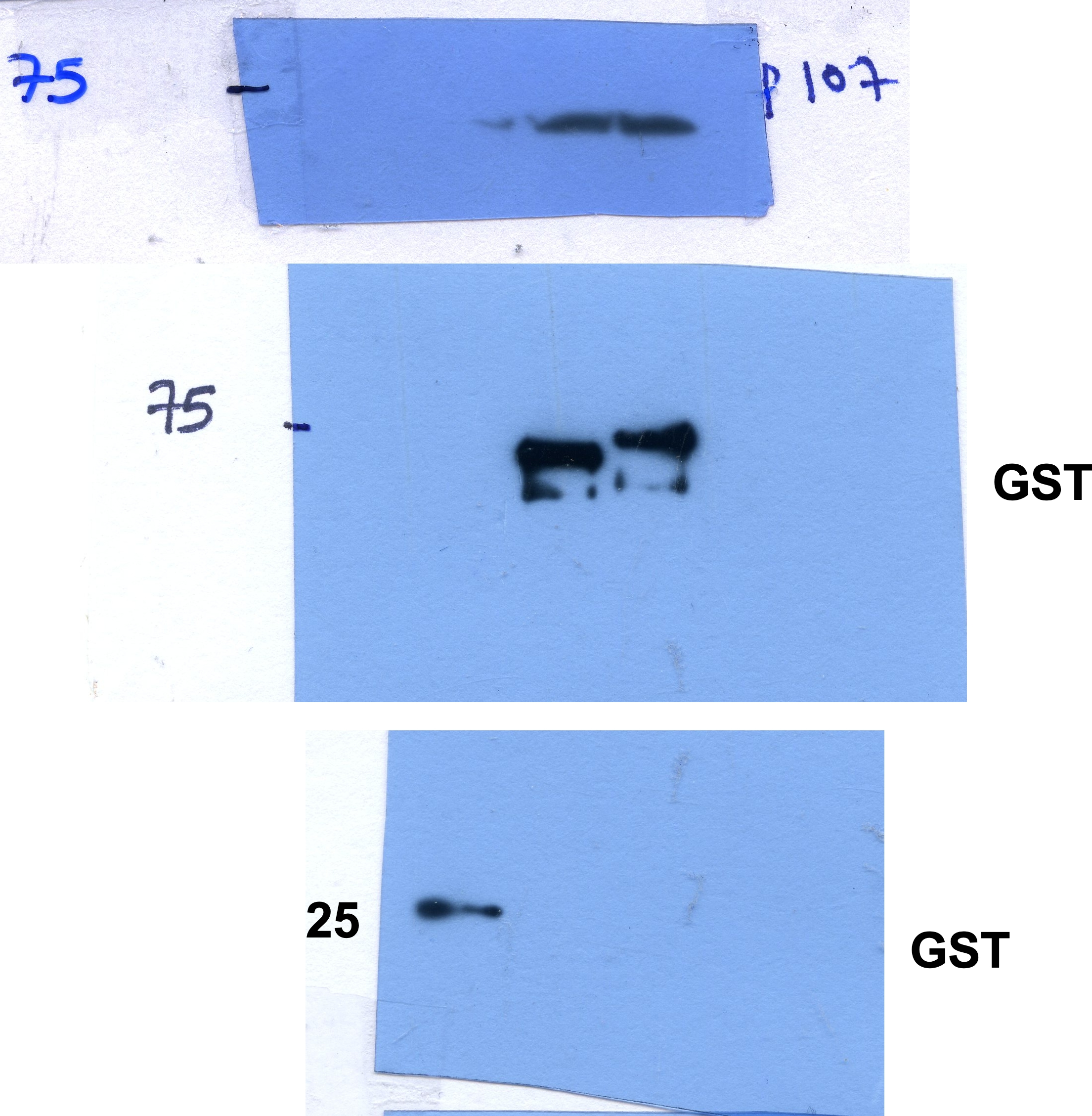

Supplement: Supplementary file 10 — Appendix Figure Source Data [file 44318_2025_402_MOESM10_ESM.zip › SD appendix figure/Figure S4/S4B/S4B Western Replicate#3.jpg]

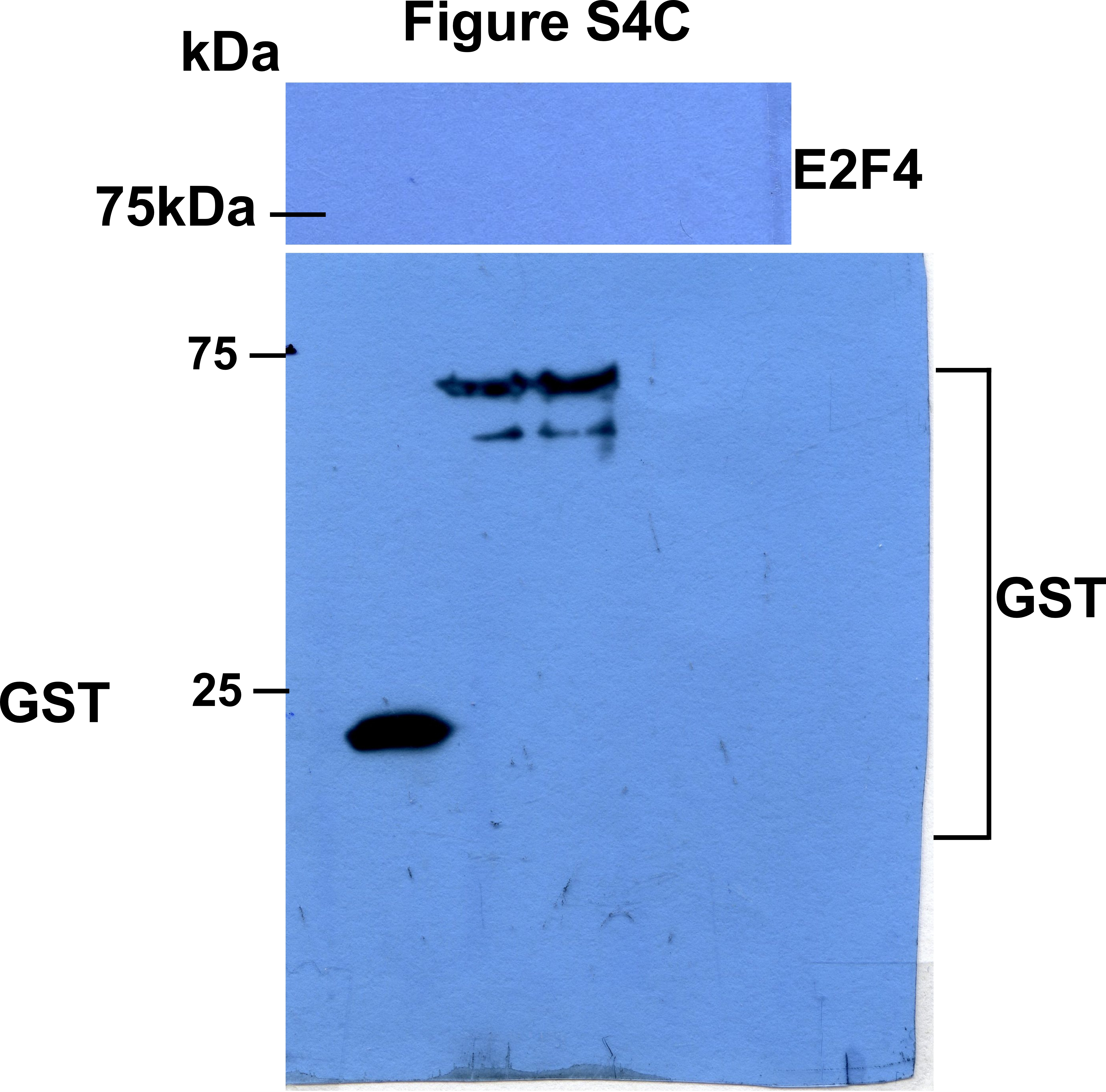

Supplement: Supplementary file 10 — Appendix Figure Source Data [file 44318_2025_402_MOESM10_ESM.zip › SD appendix figure/Figure S4/S4C/S4C Western Replicate#1 (in publication).jpg]

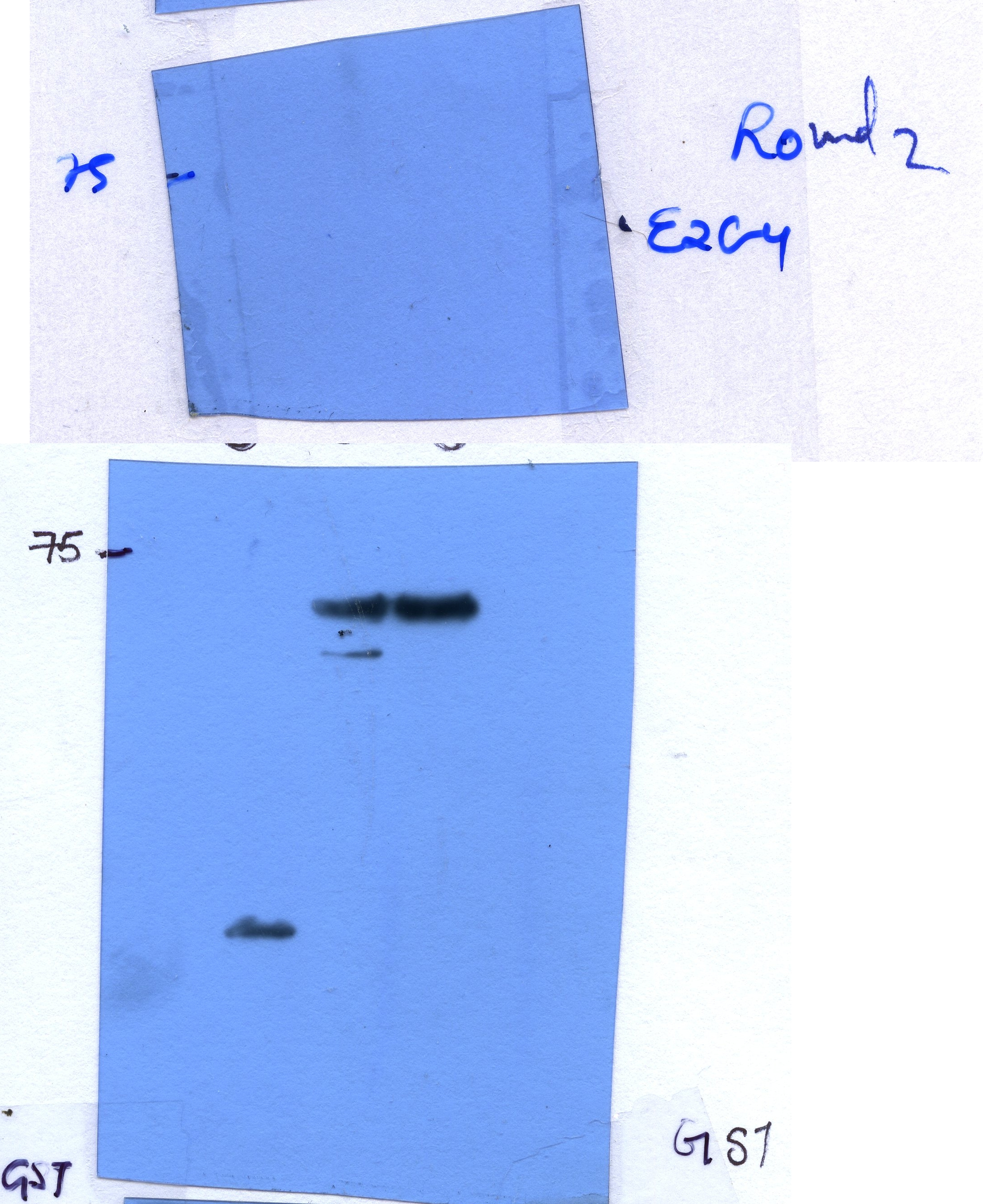

Supplement: Supplementary file 10 — Appendix Figure Source Data [file 44318_2025_402_MOESM10_ESM.zip › SD appendix figure/Figure S4/S4C/S4C Western Replicate#2.jpg]

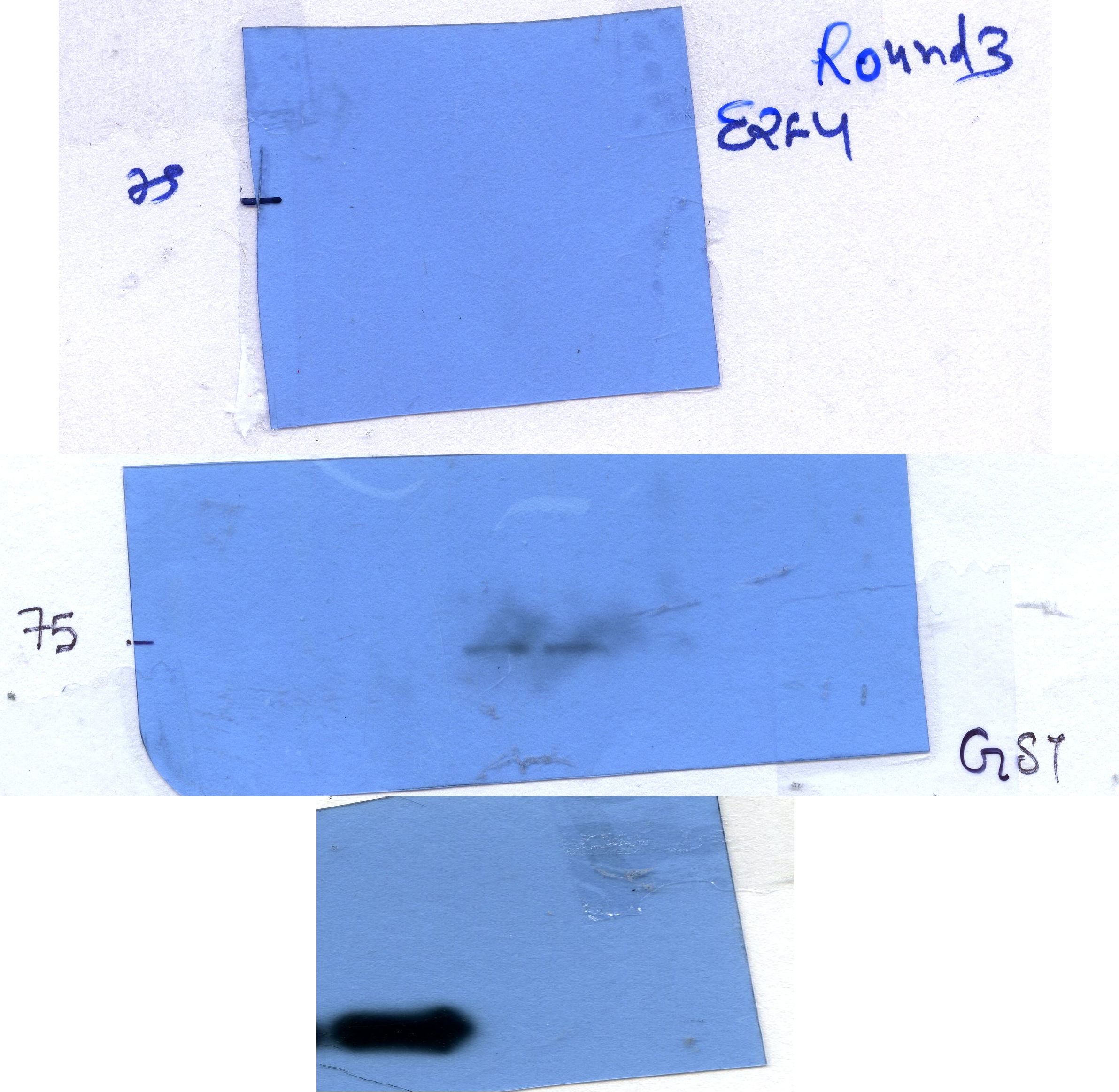

Supplement: Supplementary file 10 — Appendix Figure Source Data [file 44318_2025_402_MOESM10_ESM.zip › SD appendix figure/Figure S4/S4C/S4C Western Replicate#3.jpg]

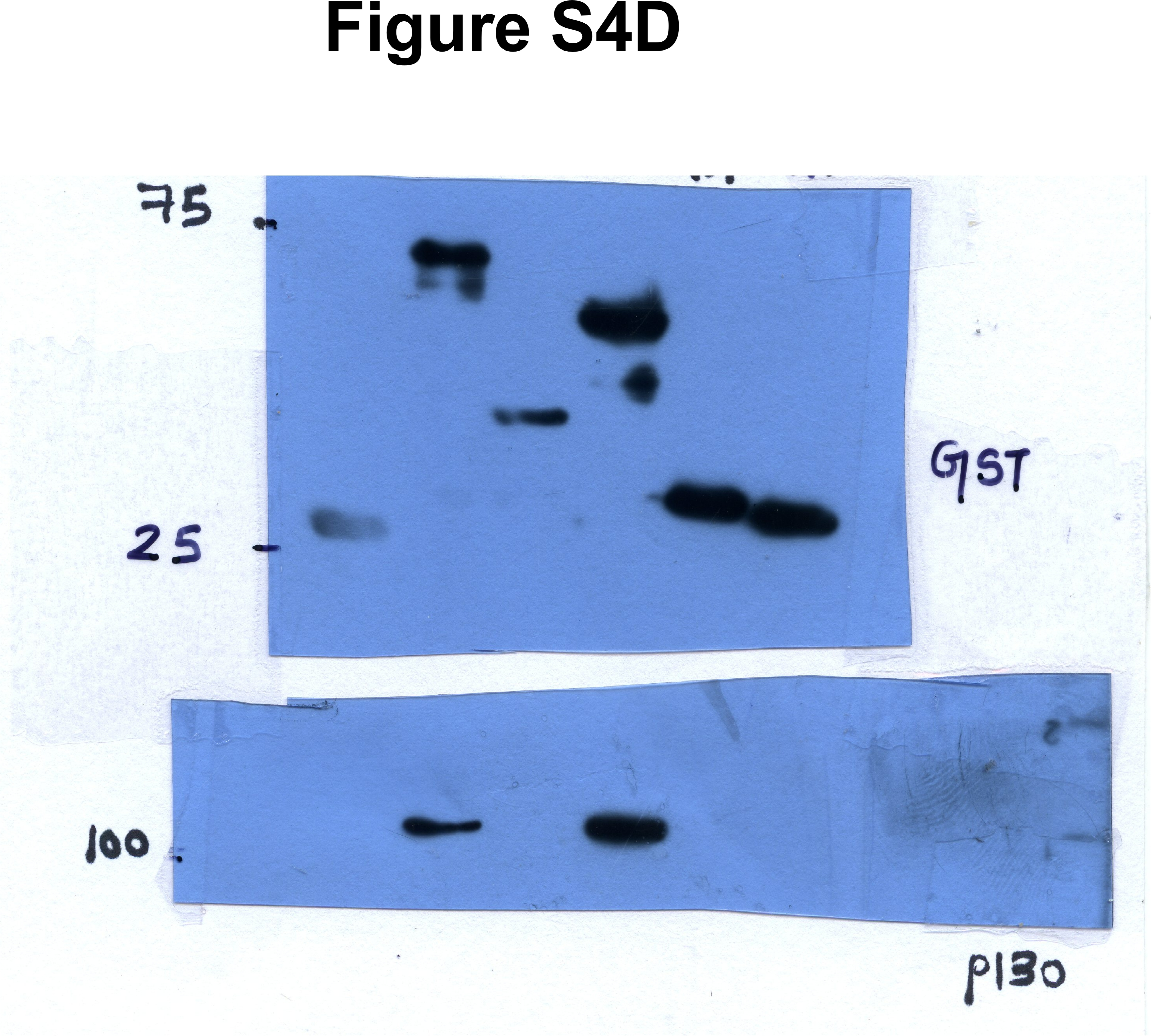

Supplement: Supplementary file 10 — Appendix Figure Source Data [file 44318_2025_402_MOESM10_ESM.zip › SD appendix figure/Figure S4/S4D/S4D Western Replicate#1 (in publication).jpg]

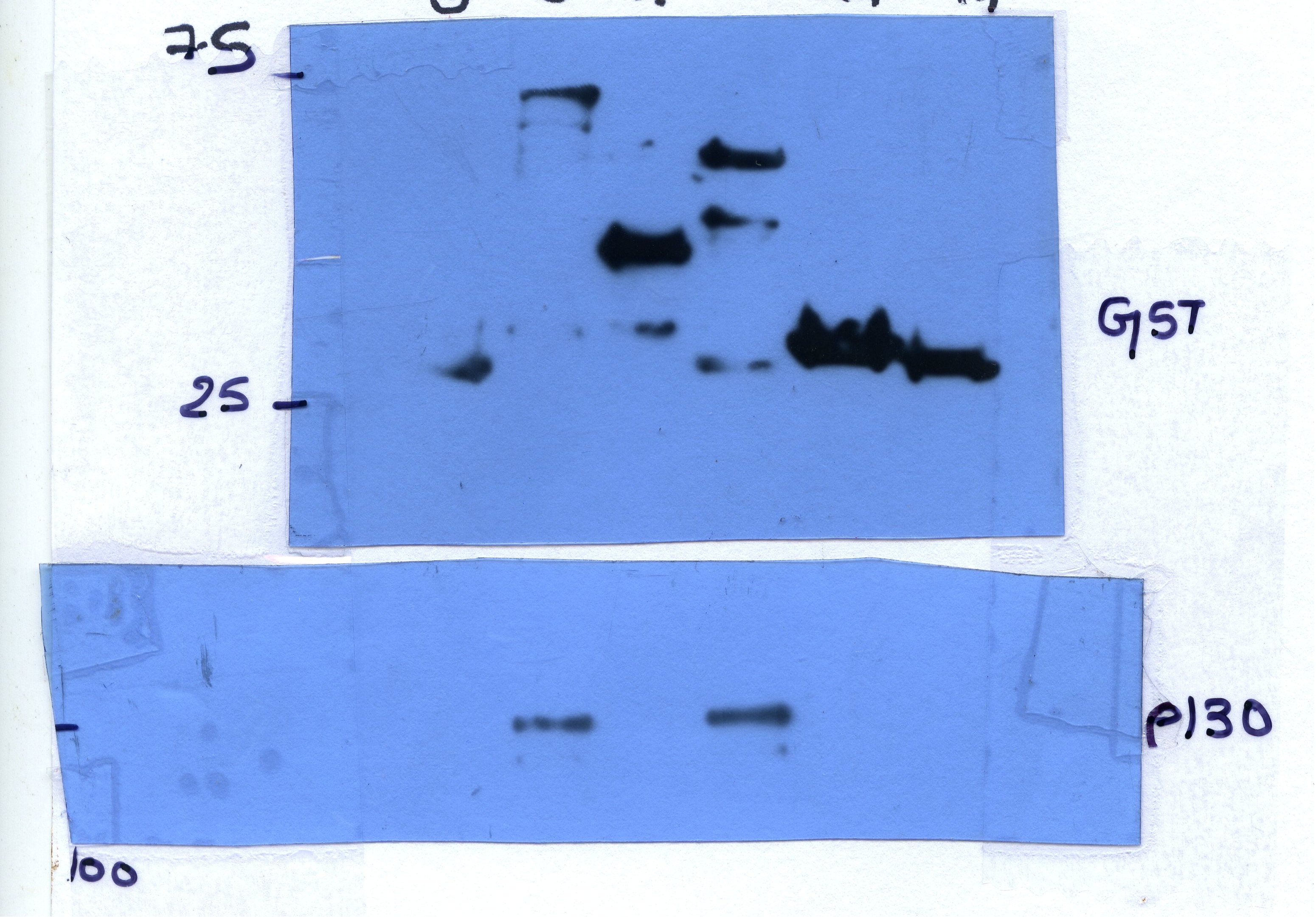

Supplement: Supplementary file 10 — Appendix Figure Source Data [file 44318_2025_402_MOESM10_ESM.zip › SD appendix figure/Figure S4/S4D/S4D Western Replicate#2.jpg]

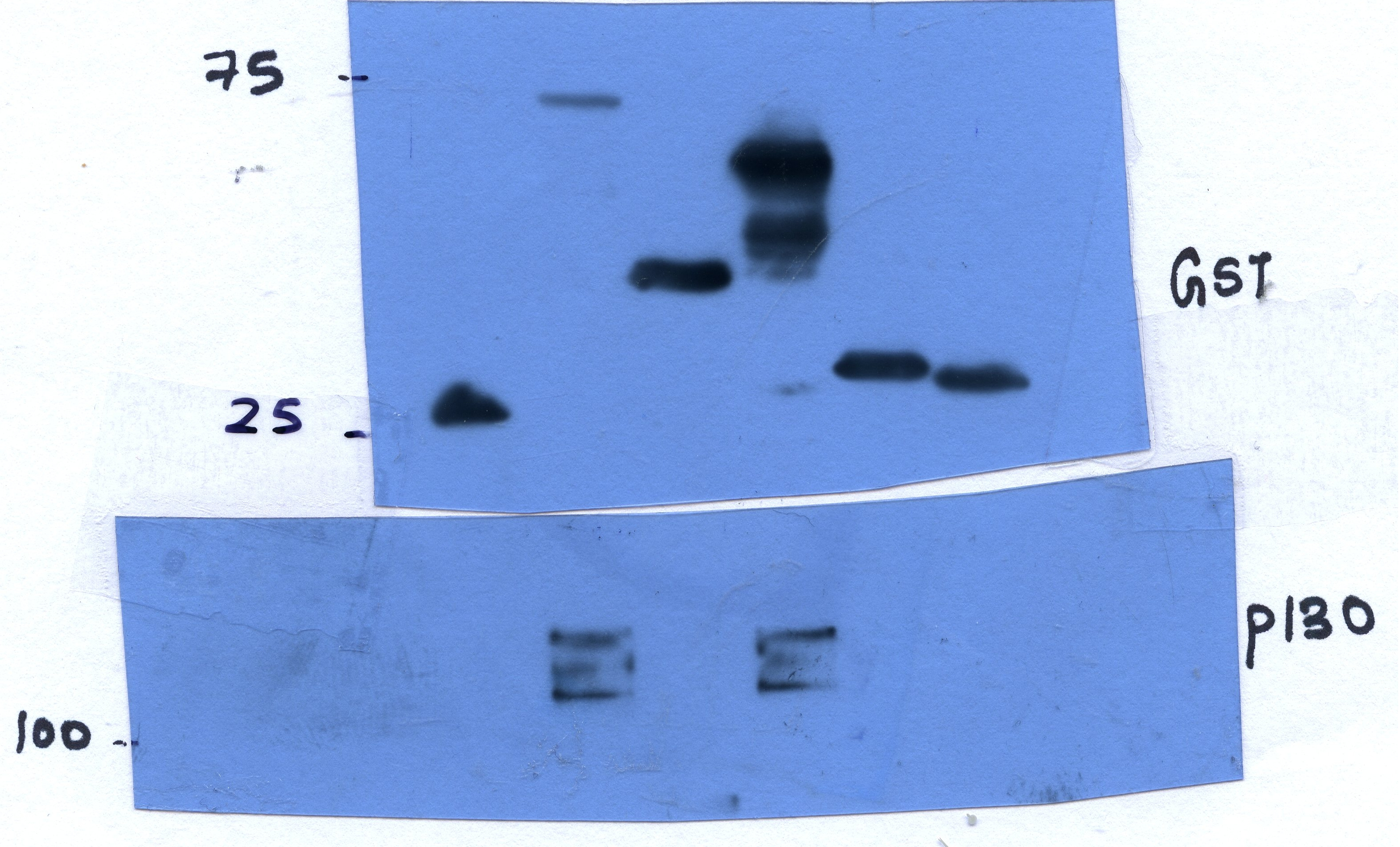

Supplement: Supplementary file 10 — Appendix Figure Source Data [file 44318_2025_402_MOESM10_ESM.zip › SD appendix figure/Figure S4/S4D/S4D Western Replicate#3.jpg]

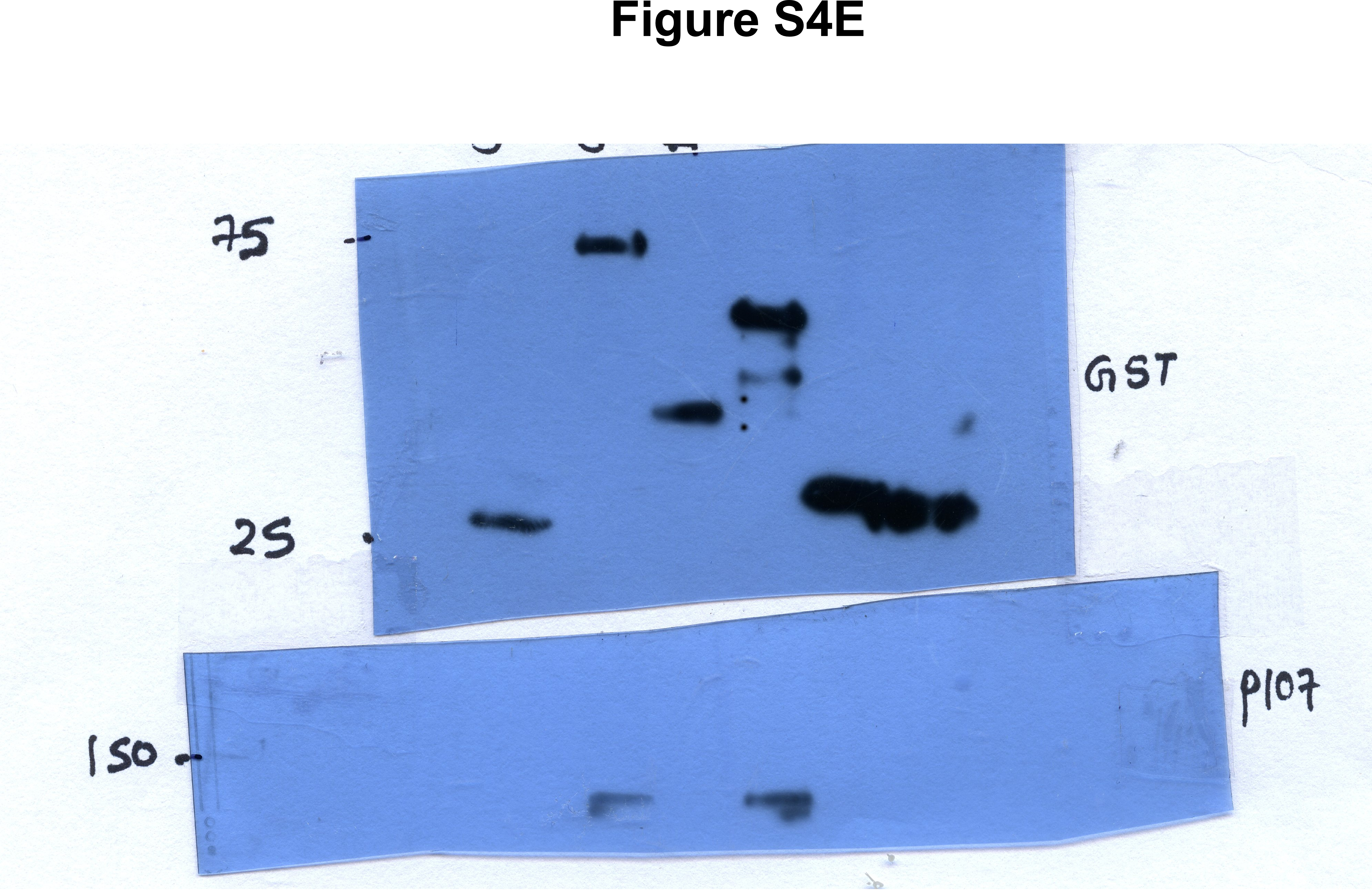

Supplement: Supplementary file 10 — Appendix Figure Source Data [file 44318_2025_402_MOESM10_ESM.zip › SD appendix figure/Figure S4/S4E/S4E Western Replicate#1 (in publication).jpg]
