# Supplementary figures and images for: p53 regulates DREAM complex-mediated repression in a p21-independent manner (part 2 of 2)
Source: EMBO J. 2025 Mar 4;44(8):2279–97. doi: 10.1038/s44318-025-00402-7 (PMC12000331; doi:10.1038/s44318-025-00402-7)

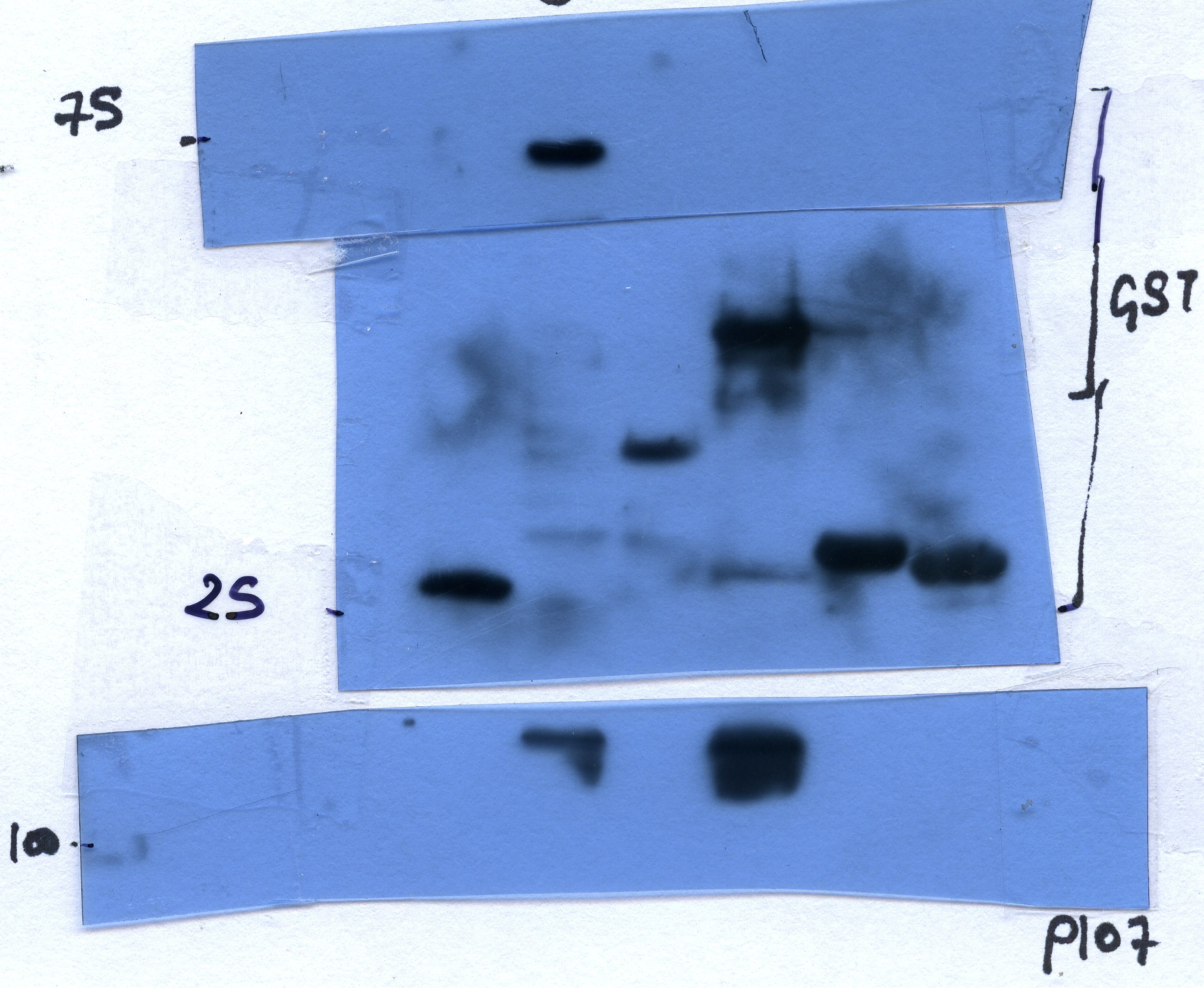

Supplement: Supplementary file 10 — Appendix Figure Source Data [file 44318_2025_402_MOESM10_ESM.zip › SD appendix figure/Figure S4/S4E/S4E Western Replicate#2.jpg]

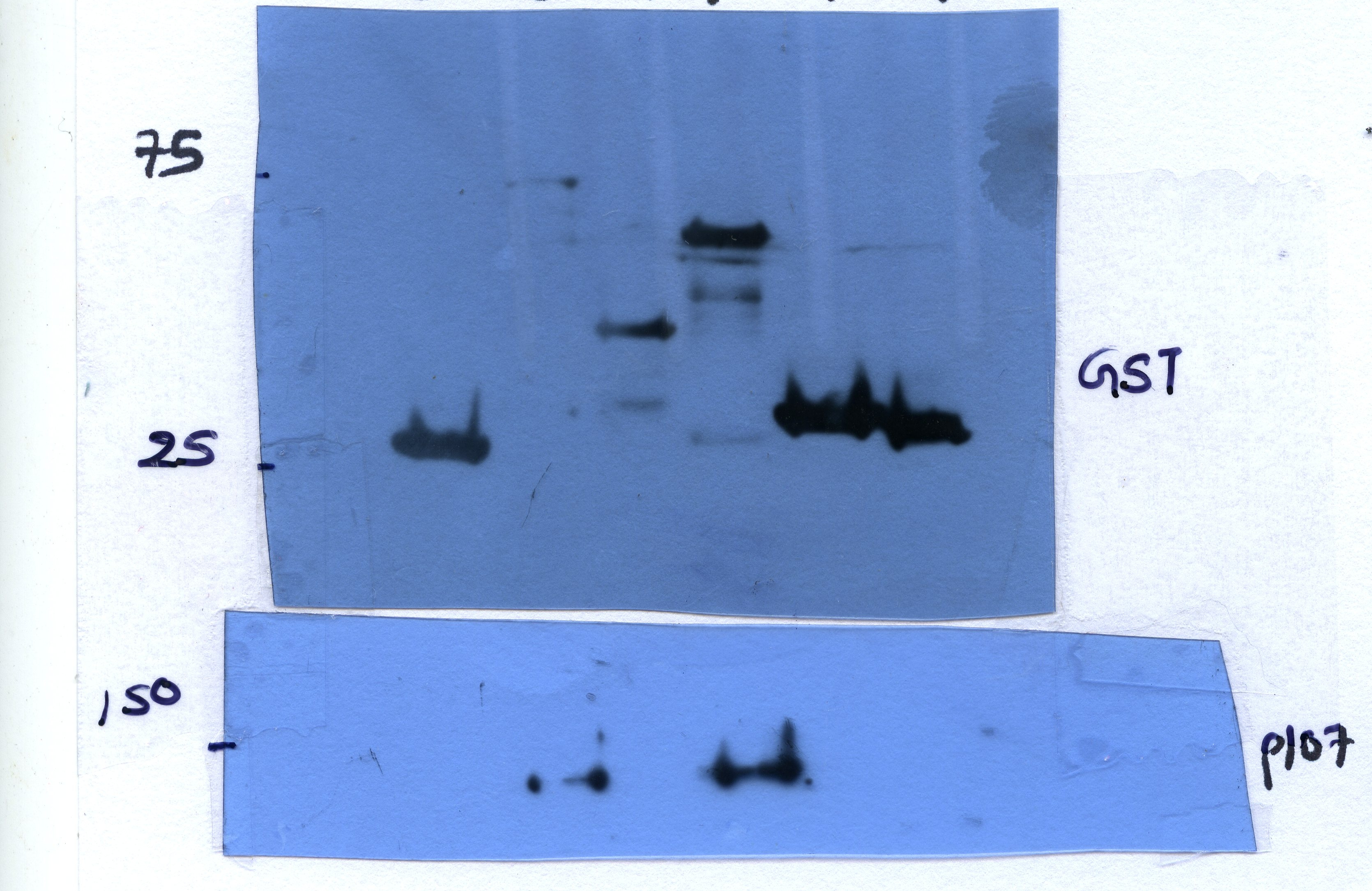

Supplement: Supplementary file 10 — Appendix Figure Source Data [file 44318_2025_402_MOESM10_ESM.zip › SD appendix figure/Figure S4/S4E/S4E Western Replicate#3.jpg]

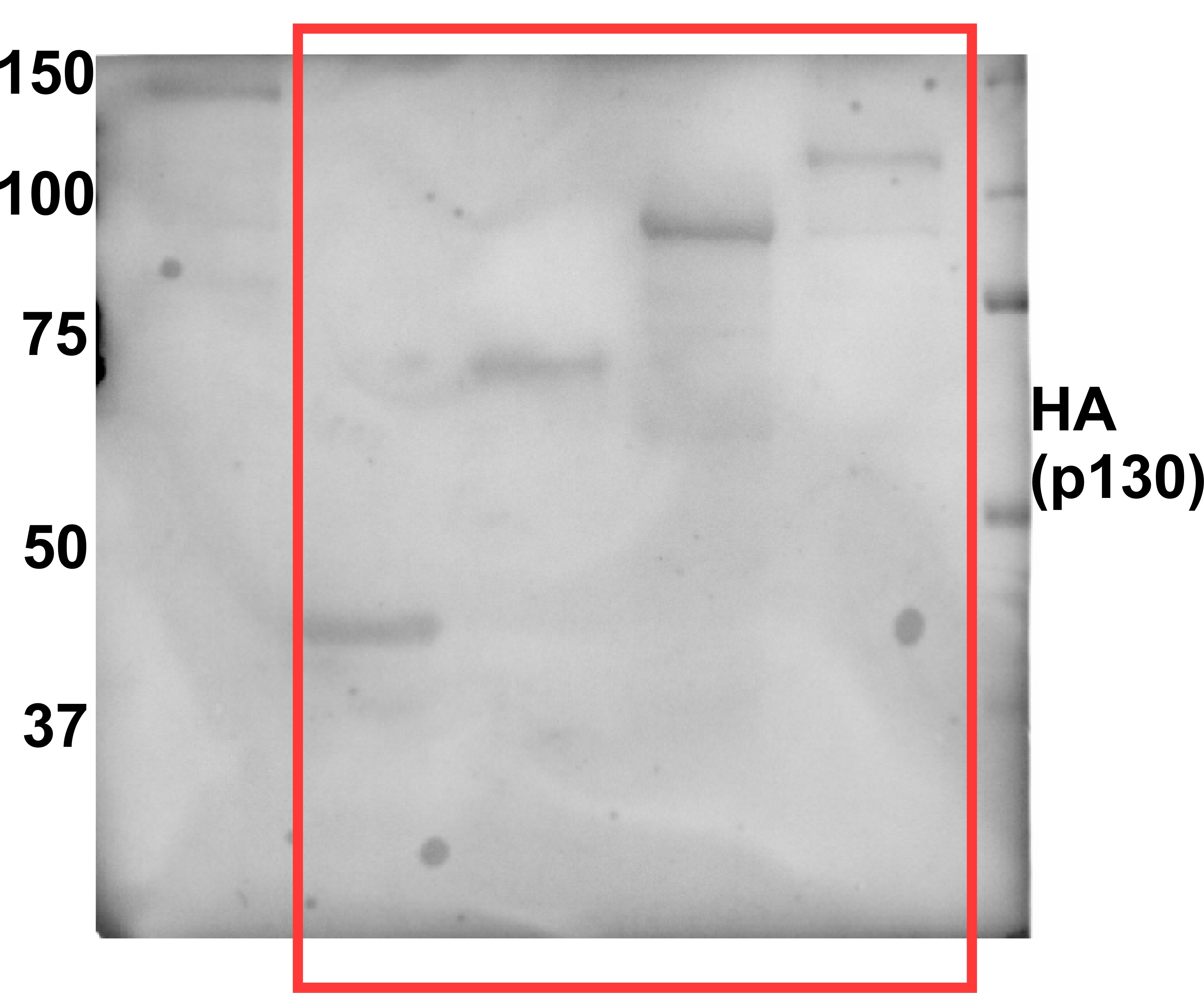

Supplement: Supplementary file 10 — Appendix Figure Source Data [file 44318_2025_402_MOESM10_ESM.zip › SD appendix figure/Figure S4/S4G/S4G Western.jpg]

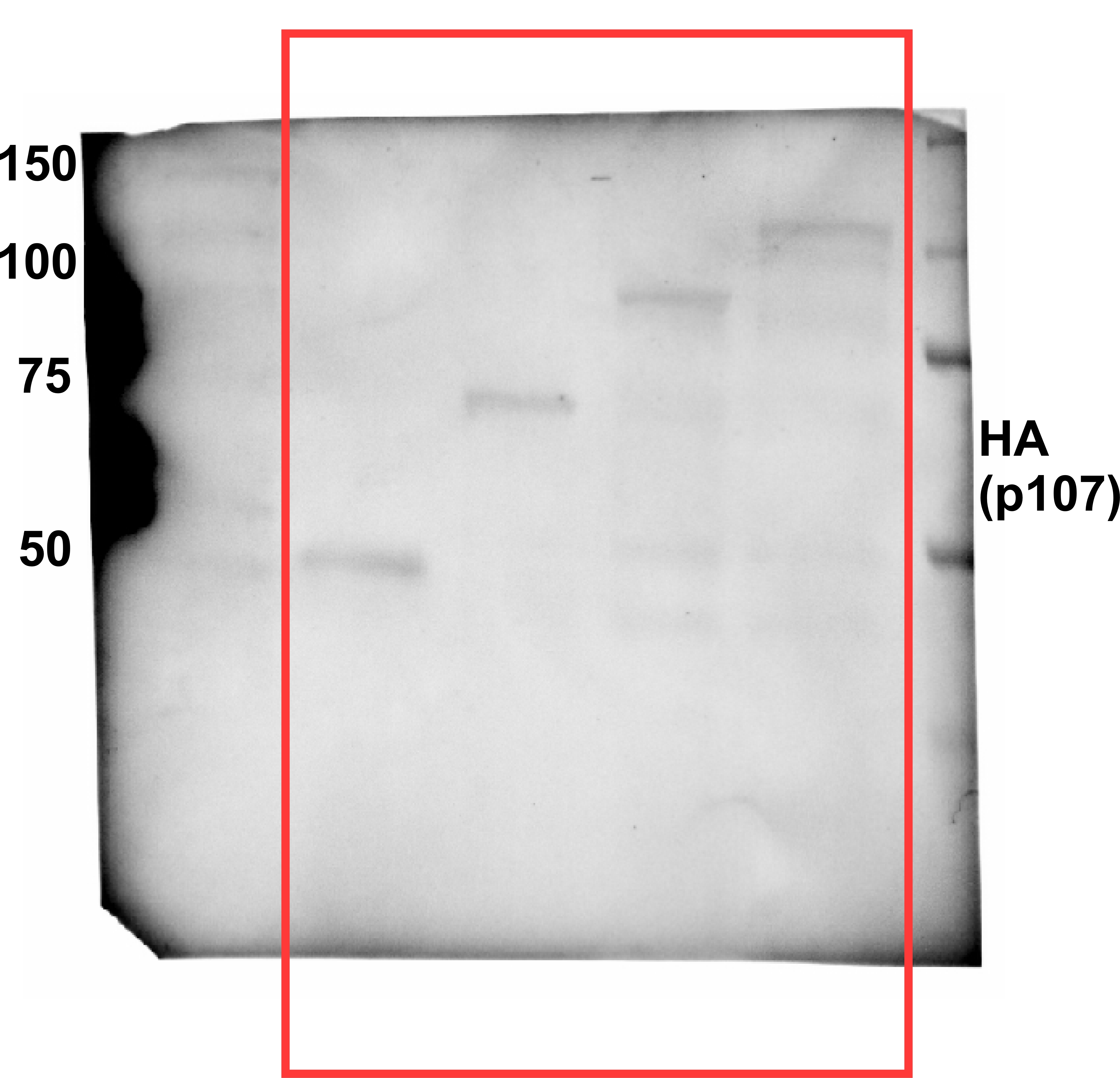

Supplement: Supplementary file 10 — Appendix Figure Source Data [file 44318_2025_402_MOESM10_ESM.zip › SD appendix figure/Figure S4/S4H/S4H Western.jpg]

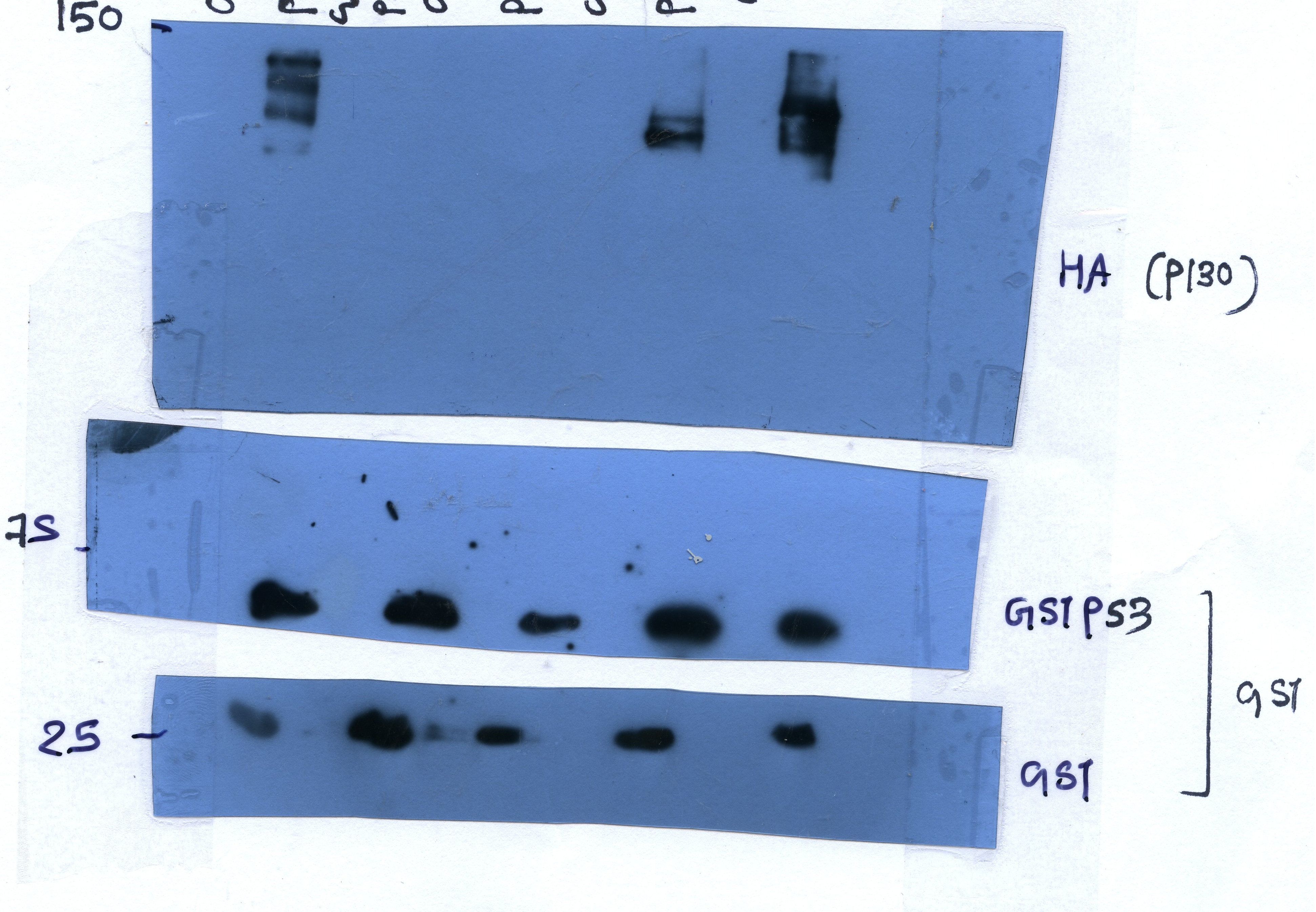

Supplement: Supplementary file 10 — Appendix Figure Source Data [file 44318_2025_402_MOESM10_ESM.zip › SD appendix figure/Figure S4/S4I/S4I Western Replicate#2.jpg]

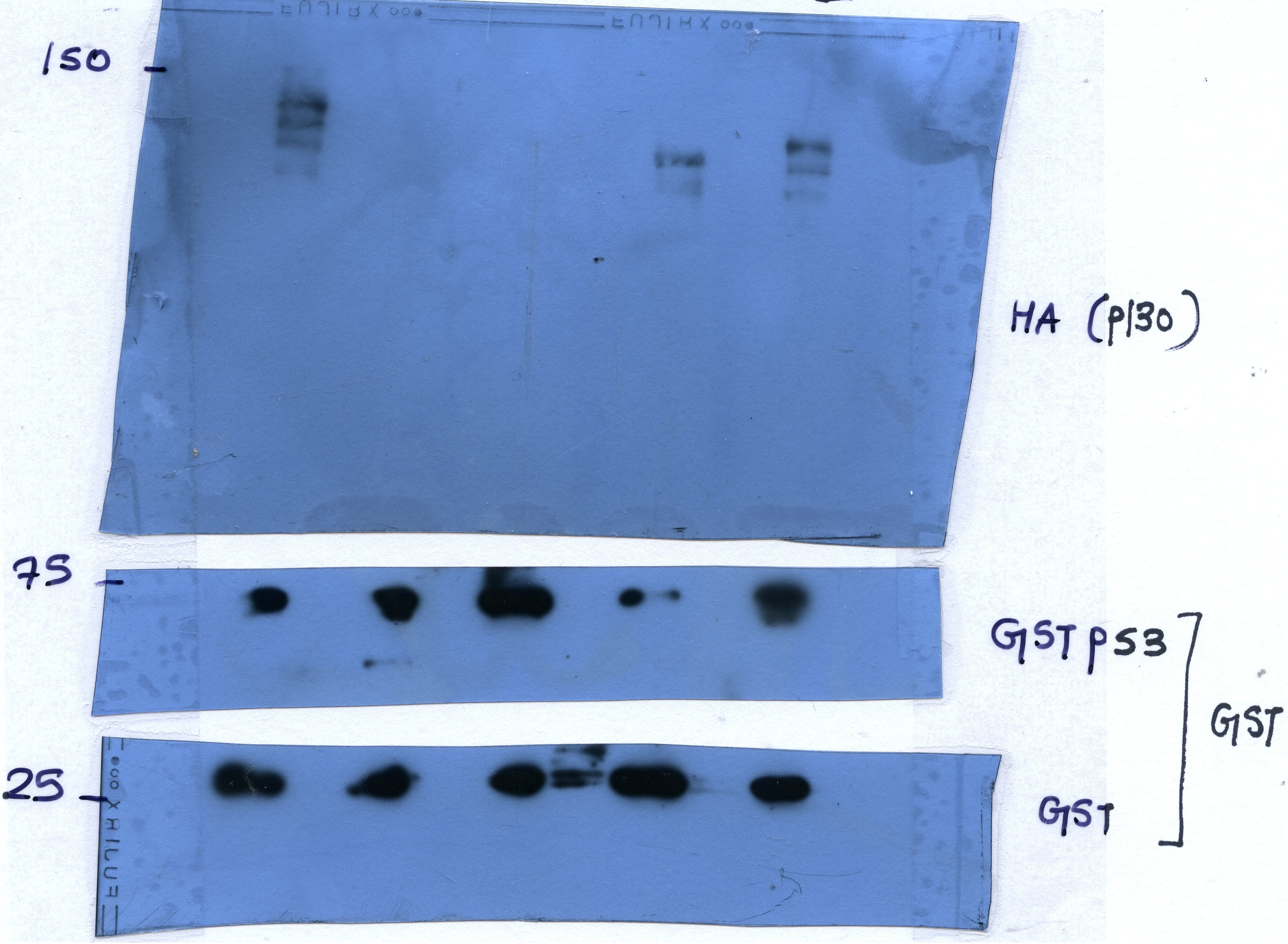

Supplement: Supplementary file 10 — Appendix Figure Source Data [file 44318_2025_402_MOESM10_ESM.zip › SD appendix figure/Figure S4/S4I/S4K Western Replicate#3.jpg]

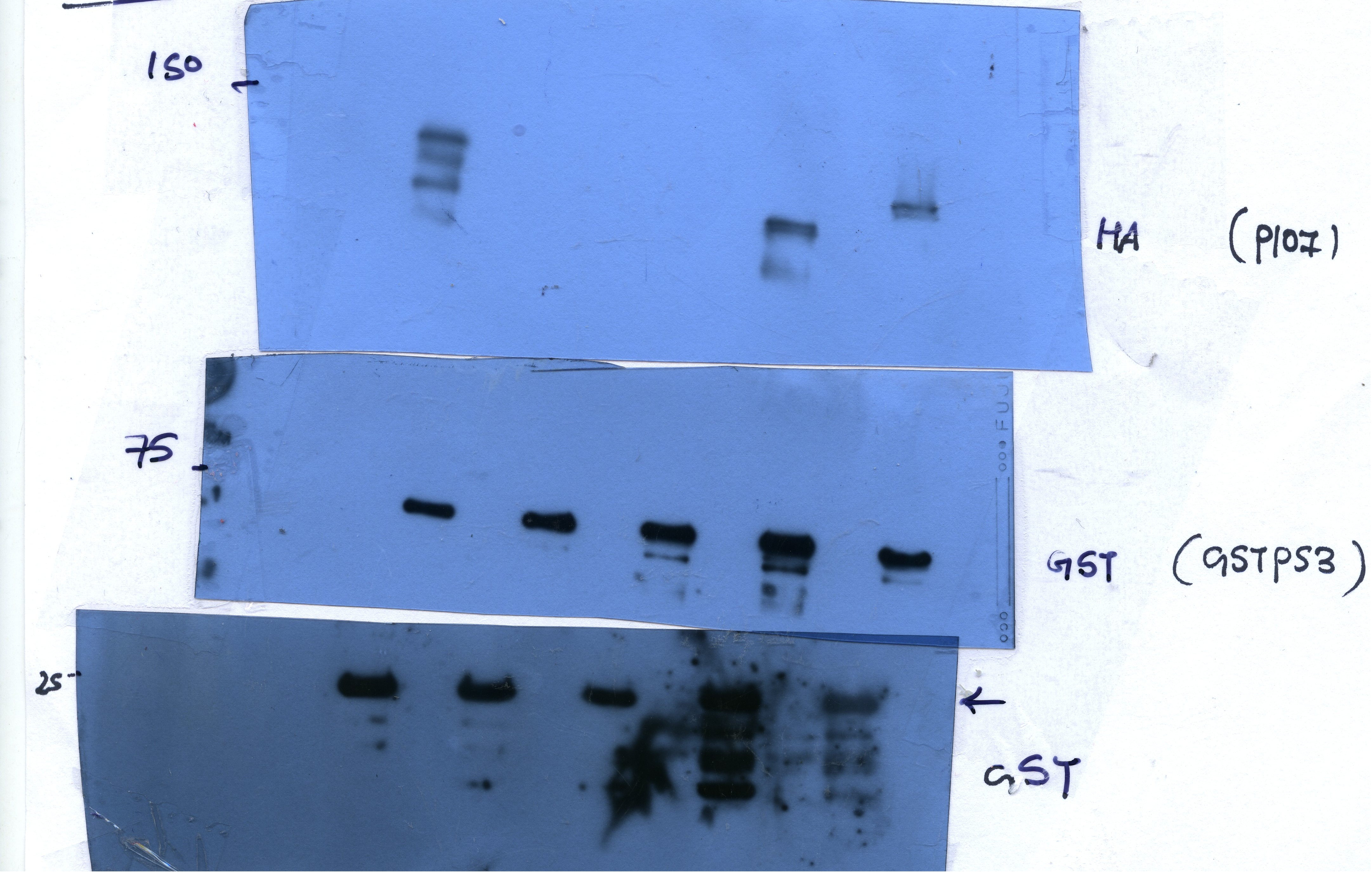

Supplement: Supplementary file 10 — Appendix Figure Source Data [file 44318_2025_402_MOESM10_ESM.zip › SD appendix figure/Figure S4/S4K/S4K Western Replicate#2.jpg]

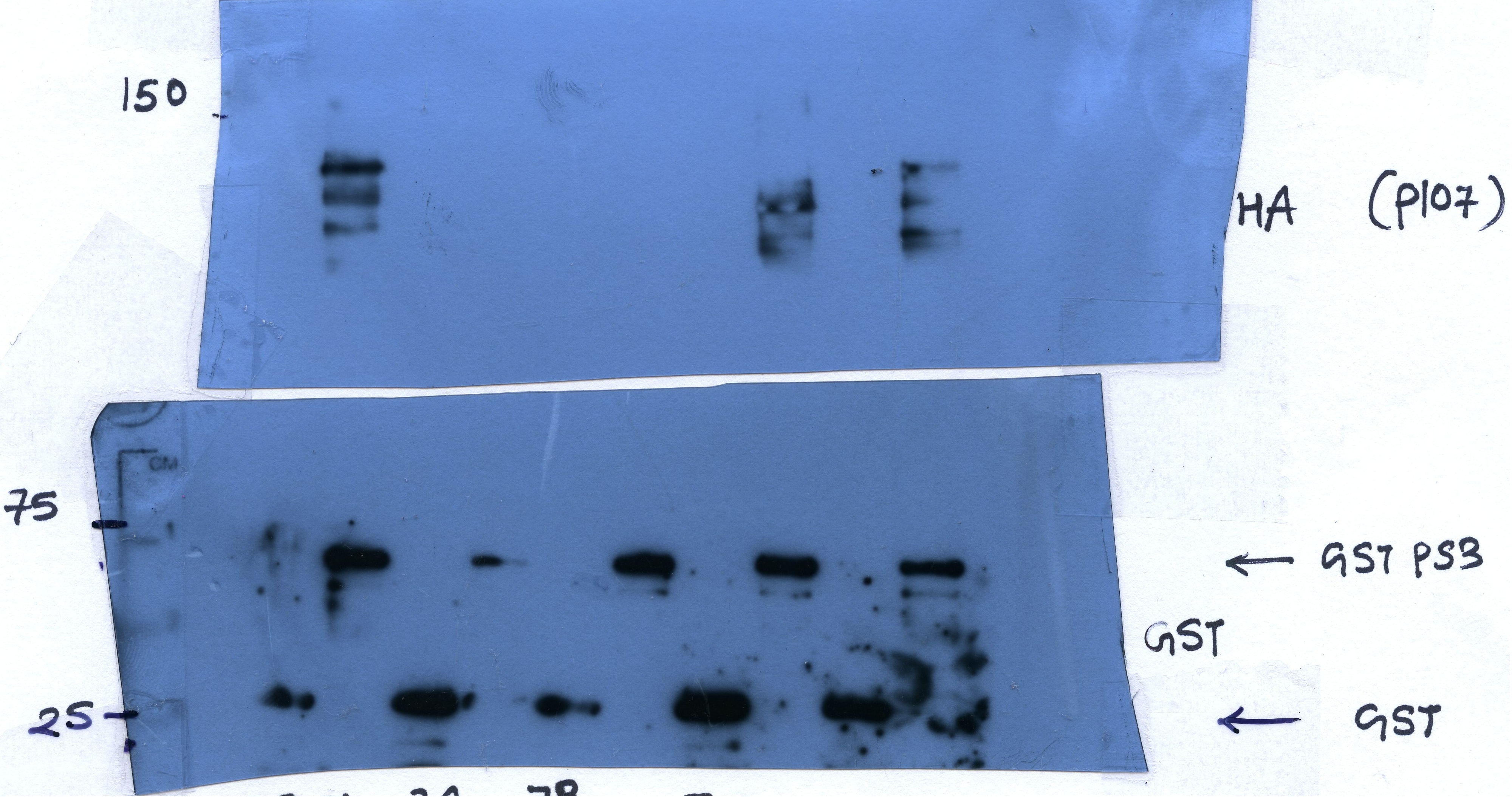

Supplement: Supplementary file 10 — Appendix Figure Source Data [file 44318_2025_402_MOESM10_ESM.zip › SD appendix figure/Figure S4/S4K/S4K Western Replicate#3.jpg]

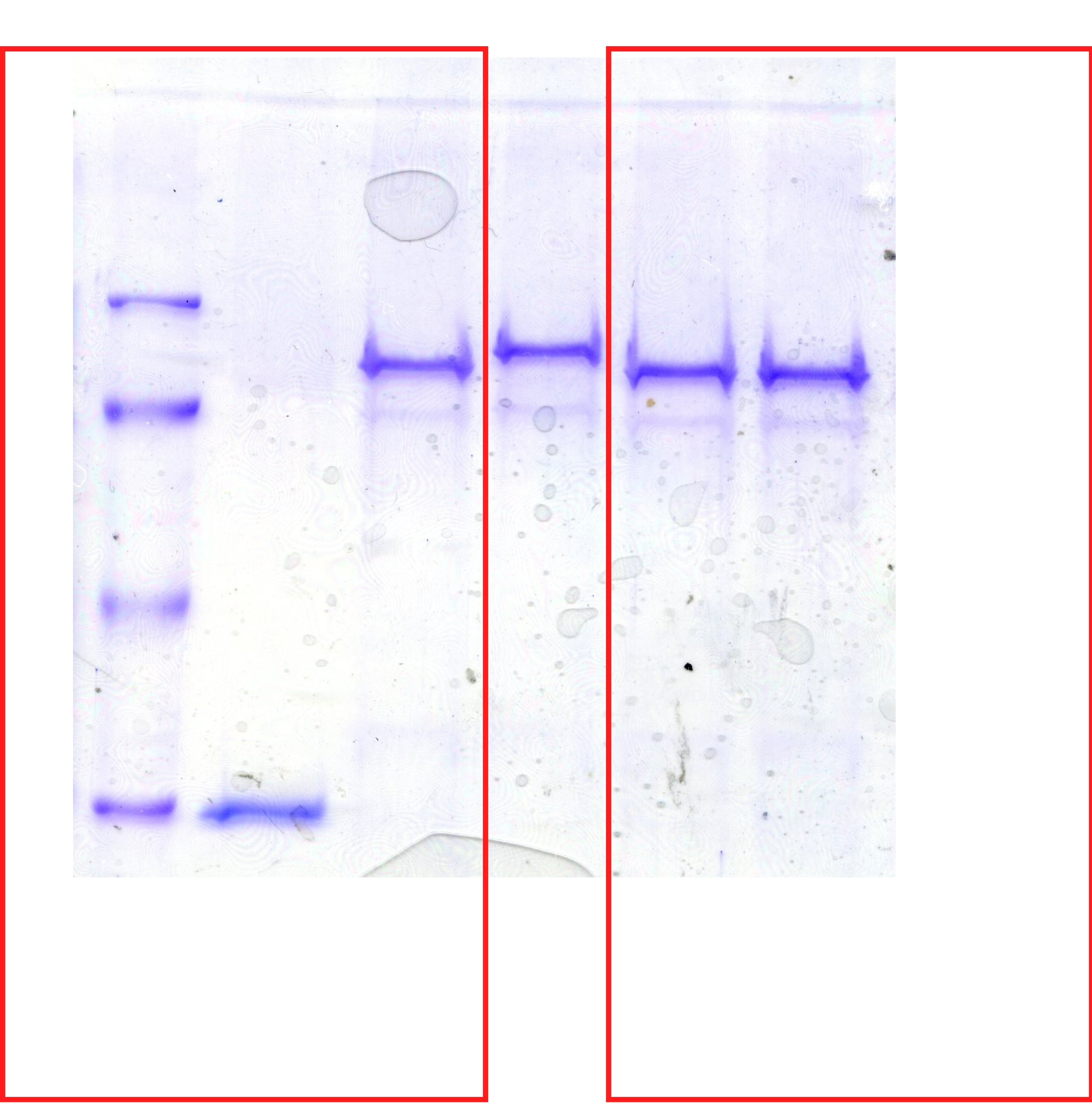

Supplement: Supplementary file 10 — Appendix Figure Source Data [file 44318_2025_402_MOESM10_ESM.zip › SD appendix figure/Figure S6/S6F/S6F Coomassie.jpg]
